# Equivalence of superspace groups

**DOI:** 10.1107/S0108767312041657

**Published:** 2012-11-14

**Authors:** Sander van Smaalen, Branton J. Campbell, Harold T. Stokes

**Affiliations:** a Laboratory of Crystallography, University of Bayreuth, Bayreuth, Germany; b Department of Physics and Astronomy, Brigham Young University, Provo, Utah 84602, USA

**Keywords:** symmetry, superspace groups, two-dimensionally modulated crystals, three-dimensionally modulated crystals

## Abstract

An algorithm is presented which determines the equivalence of two settings of a (3 + *d*)-dimensional superspace group (*d* = 1, 2, 3). The algorithm has been implemented as a web tool  on , providing the transformation of any user-given superspace group to the standard setting of this superspace group in . It is shown how the standard setting of a superspace group can be directly obtained by an appropriate transformation of the external-space lattice vectors (the basic structure unit cell) and a transformation of the internal-space lattice vectors (new modulation wavevectors are linear combinations of old modulation wavevectors plus a three-dimensional reciprocal-lattice vector). The need for non-standard settings in some cases and the desirability of employing standard settings of superspace groups in other cases are illustrated by an analysis of the symmetries of a series of compounds, comparing published and standard settings and the transformations between them. A compilation is provided of standard settings of compounds with two- and three-dimensional modulations. The problem of settings of superspace groups is discussed for incommensurate composite crystals and for chiral superspace groups.

## Abstract 2

The standard settings of (3 + *d*)-dimensional superspace groups are determined for a series of modulated compounds, especially concentrating on *d* = 2 and 3. The coordinate transformation in superspace is discussed in view of its implications in physical space.

## 1. Introduction

Symmetry is one of the most important concepts in the solid-state sciences. Knowledge of the symmetry of a crystalline compound allows the understanding of many aspects of its physical behavior, including degeneracies, the possibility of possessing non-linear properties and the anisotropy of the response to external fields. A change in symmetry at different temperatures, pressures or compositions is used as the key parameter for characterizing phase transitions of a compound. Symmetry is used for the description of phonon and electron bands and thus allows the interpretation of spectroscopic measurements on materials. Not least, symmetry restrictions on structural parameters are essential for successful refinements of crystal structures.

Theoretically, the classification of symmetry is solved. The 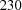 space groups give the 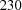 possibilities for the symmetry of a periodic structure (Hahn, 2002 ▶). Aperiodic crystals lack three-dimensional (3D) translational symmetry (Janssen *et al.*, 2006 ▶, 2007 ▶; van Smaalen, 2007 ▶). The structures of incommensurately modulated crystals are characterized by a three-dimensional lattice for the average structure together with 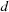 modulation waves (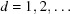) describing deviations from the lattice-periodic structure. Their symmetries are given by 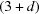-dimensional [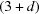
D] superspace groups (de Wolff *et al.*, 1981 ▶). The latter are space groups of 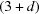
D space, which have to obey particular conditions in order to qualify as symmetry groups for the symmetries of aperiodic crystals. Incommensurate composite crystals are described by the same superspace groups as modulated crystals (van Smaalen, 2007 ▶), while quasicrystals require a slightly modified treatment (Janssen *et al.*, 2007 ▶; Steurer & Deloudi, 2009 ▶).

We have recently generated a complete list of superspace groups and their Bravais classes of dimensions 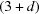 for 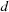 = 1, 2 and 3 (Stokes *et al.*, 2011*a* ▶). The list agrees with previous information on 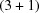
D superspace groups (Janssen *et al.*, 2006 ▶), but it contains numerous corrections for superspace groups of dimensions 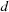 = 2 and 3 (Yamamoto, 2005 ▶) and even some corrections to the Bravais classes of dimensions 2 and 3 (Janssen *et al.*, 2006 ▶). The results of Stokes *et al.* (2011*a* ▶) are compiled in the form of the web-based data repository 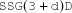 (Stokes *et al.*, 2011*b* ▶). 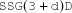 provides several types of information for each superspace group, including the Bravais class, the list of symmetry operators and reflection conditions in both standard and supercentered settings.

It is noticed that Stokes *et al.* (2011*a* ▶) have defined the standard settings and their symbols by a set of judiciously chosen rules, which, however, include subjective choices. The standard setting thus is defined as the setting included in the list of superspace groups on 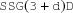.

The use of alternate settings of space groups is a well known feature for three-dimensional space groups. Volume A of the *International Tables for Crystallography* (Hahn, 2002 ▶) provides several settings for monoclinic space groups, thus showing, for example, that 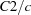, 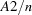 and 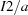 denote different settings of space group No. 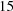.^1^ Alternate settings of three-dimensional space groups arise owing to different choices of the unit cell. Trivial transformations include a simple relabeling of the axes 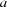, 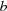 and 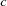. For monoclinic space groups this implies the freedom to select the unique axis as 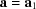, 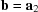 or 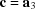. For the example of space group No. 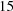 the transformation 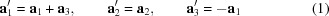
takes the setting 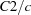 into 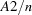, while both settings refer to a unique axis 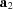. Applying the transformation of equation (1) to the setting 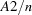 results in the third setting 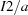. Other notorious pairs of equivalent settings include the 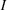 and 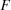 settings for space groups based on the 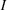-centered tetragonal lattice, primitive and centered hexagonal settings as well as obverse *versus* reverse settings for rhombohedral space groups, and the 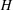-centered setting as an alternative to the primitive setting for trigonal space groups (Hahn, 2002 ▶).

Different diffraction experiments independently lead to any of the possible settings of the space group. It is then of high practical importance to find the transformation between these settings or to establish that two different space groups have indeed been obtained. The latter situation implies different compounds or different phases of one compound, while different settings of one space group imply that the same compound has been studied. In other experiments it is important that a previously defined orientation of a crystalline material is re-established, thus requiring the relation to be found between the newly found setting and a standard setting.

As a start, superspace groups for incommensurately mod­ulated compounds and incommensurate composite crystals exhibit the same variation of settings as three-dimensional space groups do, since 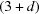
D superspace groups are based on a basic structure lattice and space group in three-dimensional space. In addition, superspace groups may appear in many more different settings, owing to the ambiguity in the choice of the modulation wavevectors characterizing the structure and the diffraction pattern. The equivalence of different settings of a superspace group is not always obvious. In some cases, establishing an actual equivalence can be a computationally prohibitive task unless appropriate algorithms are used. Here we present such an algorithm, which was used but not described in detail in our previous publication Stokes *et al.* (2011*a* ▶). It is available within 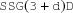 as a tool with which to determine the transformation between a user-provided setting of a 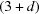
D superspace group and the standard setting defined by 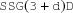 (Stokes *et al.*, 2011*b* ▶). It thus can be used to establish or disprove the equivalence of settings.

Coordinate transformations between different settings of three-dimensional space groups are discussed in Volume A of *International Tables for Crystallography* (Hahn, 2002 ▶). For 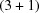
D superspace groups, typical transformations are presented in van Smaalen (2007 ▶). The possibility to combine two modulation wavevectors into an equivalent but different set of two wavevectors leads to new types of transformations for 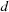 = 2 and 3.

One goal of this paper is to present an overview of typical coordinate transformations that may occur between settings of superspace groups. Particular attention is given to the relation between the formal description in 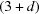
D space, as given on 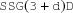 (Stokes *et al.*, 2011*a* ▶), and an experimentally related description in terms of a rotation and an origin shift in three dimensions (van Smaalen, 2007 ▶). Where available, we use substances published in the literature to illustrate important transformation types.

## 2. Equivalence of superspace groups

### 2.1. Definitions

The following definitions are used by Stokes *et al.* (2011*a* ▶), van Smaalen (2007 ▶) and Janssen *et al.* (1995 ▶). A 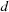-dimensionally modulated structure is characterized by 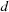 rationally independent modulation wavevectors 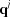 with components compiled in a 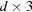 matrix 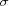 according to 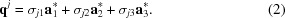
For an aperiodic structure at least one component in each row of 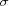 is an irrational number. The reciprocal vectors 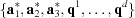 in physical space correspond to the reciprocal basis vectors 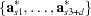 in superspace. The basis vectors of the direct lattice in superspace are 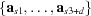 and the coordinates of a point in superspace are 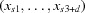.

Note that the 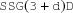 data repository, and also the web-based 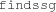 and 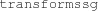 tools described herein, presently use the 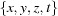 notation to indicate superspace coordinates, though the same notation is also commonly used to indicate physical- and internal-space coordinates.

An operator 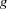 of a 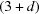
D superspace group 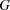 consists of a rotation 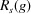 and a translation 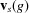 given in matrix form as 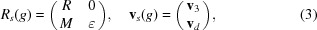
where 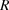 is a 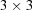 integer matrix and 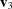 is a three-dimensional column vector, together defining the operator 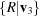 in physical space. 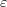 is a 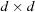 integer matrix, and 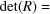 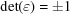. The 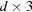 integer matrix 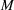 is defined as [equation (2)] 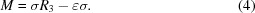


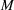
 has nonzero components only in the case that at least one of the modulation wavevectors incorporates nonzero rational components. Following Stokes *et al.* (2011*a* ▶), each operator 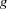 can be written as an augmented 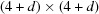 matrix 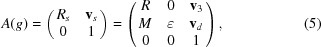
that simultaneously treats the point and translational parts of the operation. The action of operator 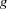 on a point in superspace then is given by the matrix product 
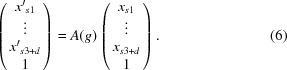
In superspace a coordinate transformation can be accomplished by the augmented affine transformation matrix 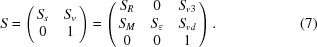
The components of 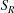, 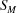 and 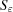 are required to be integers. Also, 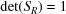 and 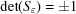. The transformation 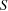 can be interpreted as a rotation 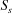 in superspace followed by a change of origin 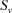. The effect of this transformation in physical space can be described in terms of a rotation of the reciprocal basis and the choice of an alternate set of modulation wavevectors, according to 
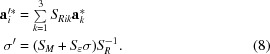
Two 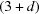
D superspace groups, 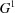 and 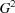, are equivalent if a single transformation 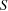 can be found, such that for every 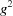 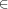 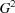, 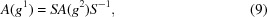
for some 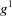 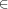 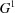. Note that these definitions imply that a primitive setting is used for the superspace group, where all lattice translations are represented by integers, even those which are centering translations in a conventional setting (Stokes *et al.*, 2011*a* ▶).

It is sufficient that the relation of equivalence [equation (9)] is tested for corresponding pairs of non-translational generators from the two superspace groups; the generators of the translation subgroup need not be considered. Furthermore, a transformation of the type of equation (9) can only be found between operators 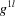 and 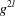 if [equation (3)] 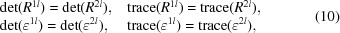
where 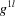 is the 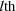 generator of 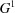 and 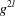 is the corresponding generator of 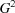. The appropriate pairs of generators are obtained by consideration of the basic structure space group implied by the superspace group. Other, trivial properties that need to be fulfilled for equivalence and that are easily tested include the number of operators in the point group of the superspace group, which must be equal for 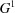 and 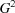.

### 2.2. The algorithm determining equivalence

The goal of testing for equivalence of two superspace groups is to find the augmented matrix 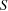 with which the operators of 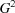 are transformed into corresponding operators of 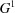, or to establish that a matrix 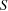 that solves equation (9) simultaneously for all pairs of generators does not exist. Equation (9) is quadratic in 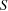 but can be recast in linear form as 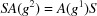. Given that a pairing has been established for the 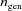 generators of 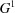 and 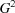, this results in 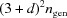 equations for 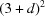 variables 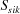, 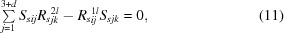
and 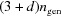 equations for additional 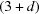 variables 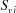, 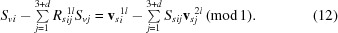
The translational parts of the operators 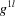 and 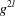 are only known up to a lattice translation, which is taken into account by the mod 1 in equation (12).

Employing the special structure of 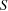 [equation (7)], the variables 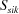 can be ordered in a column vector as 
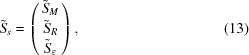
where, for example, 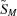 is obtained by juxtaposition of the columns of 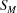 into a single column matrix. This procedure eliminates the 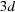 variables that are zero according to equation (7) and results in 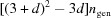 equations in 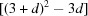 variables 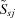, 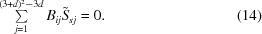
The 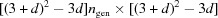 matrix 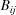 is obtained by rearrangement of equation (11), followed by linear row operations that bring it into row echelon form. In this form, the first nonzero element in each row occurs in a column where it is the only nonzero element. If 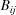 is such an element, then 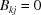 for all 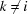 and 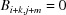 for all 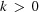 and 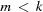. This equation relates the ‘dependent’ variable 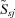 to ‘independent’ variables according to 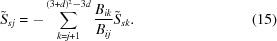
The number of independent equations is smaller than or equal to 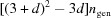. If the number of independent equations is larger than 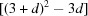, a solution does not exist for 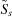, and the two superspace groups are shown to be inequivalent. Alternatively, the number of independent equations can be equal to 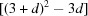, then defining a unique solution for 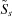. Finally, the number of independent equations can be smaller than 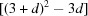, resulting in more than one solution to equation (15). Once values for the independent variables have been chosen, equation (15) can be used to compute the remaining variables of 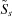 and 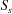. For each solution 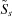 of equation (15), equation (12) may or may not provide a solution for the translational parts of the transformation.

The strategy for finding the transformation 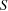 is now as follows. For each trial set of integers for the independent variables 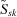, check that all dependent variables 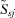 compute to have integer values and that 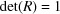 and 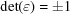. If not, discard the trial set. If so, use the values 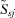 (both dependent and independent) in equation (12) and explore trial integer sets of variables 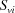 in search of a modulo 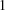 solution. If a solution is found, then equations (11) and (12) are both satisfied and the two sets of superspace-group operators, 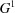 and 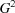, represent distinct but equivalent settings of the same superspace group. If no solution is found, then we can assume that the groups are not equivalent, provided that we have a robust algorithm that searches a sufficiently wide range of trial values for each independent variable as to guarantee a solution to equations (11) and (12) provided one exists. The method of choosing these variable exploration ranges is described in the supplementary material.^2^

The number of variables, and therefore the computational complexity of the search, increases with the dimension 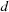 of the modulation. Furthermore, the goal of the proposed analysis is to determine which superspace group in the 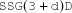 tables a user-given superspace is equivalent to. Since the number of superspace groups strongly increases with 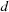, the number of candidate equivalencies that need to be tested increases dramatically with increasing dimension of the superspace, easily reaching several hundreds of groups in the worst case (orthorhombic symmetry). Thus we need an algorithm for evaluating the possible equivalence of two superspace groups that is not only robust but also efficient. The efficiency of the algorithm boils down to finding the most restrictive number of trial sets of integers for which equivalence needs to be tested (see the supplementary material).

An algorithm based on these rules has been implemented in the software 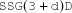 (Stokes *et al.*, 2011*a* ▶). For any user-given set of superspace operators, the web tool 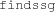 determines the complete list of operators (modulo lattice translations) of the superspace group that they generate, as well as a minimal list of generators, identifies the equivalent superspace group in the 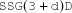 tables and provides the coordinate transformation 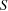 to the standard setting [equation (7)].

## 3. Alternate settings of (3 + 1)-dimensional superspace groups

### 3.1. The basic structure space group

An important reason for the occurrence of non-standard settings of superspace groups is the common use of different standard settings for superspace groups and three-dimensional space groups. Structural analysis of modulated crystals often proceeds by the initial determination from the main reflections of the periodic basic structure along with its three-dimensional space group (the basic structure space group, BSG). Subsequently, satellite reflections are considered and modulation functions and the superspace group are determined. For other substances the incommensurate phase is the result of a phase transition, so that the three-dimensional space group of the unmodulated structure at ambient conditions is known independently. This space group, or one of its subgroups, is preserved as the BSG of the incommensurate phase.^3^

In all these cases the BSG is specified before the symmetry of the modulation is considered. It is then a matter of chance that the superspace group thus obtained will or will not be in its standard setting. These points can be illustrated by space group No. 62 with standard setting 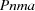 (Hahn, 2002 ▶). In this setting, TaSe
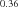
Te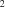 is modulated with 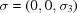 and 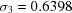 [equation (2); van der Lee *et al.* (1994) ▶], so that the 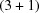
D superspace-group symbol is 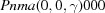, which is the standard setting for superspace group No. 62.1.9.1 in 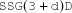.

Thiourea has a lattice-periodic structure with space group 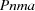 at ambient conditions. Below 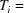 202 K it develops an incommensurate modulation with 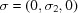 in the 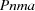 setting (Gao *et al.*, 1988 ▶; Zuñiga *et al.*, 1989 ▶). Combining the BSG and modulation wavevector leads to the 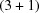
D superspace group 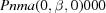. 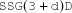 shows that this is an alternate setting of superspace group No. 62.1.9.3, for which the standard symbol is 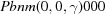. The augmented matrix 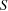 that transforms coordinates from the original (unprimed) to the standard (primed) settings [equation (6)] is given in 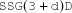 as 
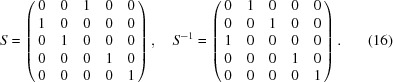
According to equation (8) the new basis vectors of the basic structure are obtained as the upper-left 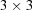 part of the transpose of 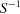. Inspection of equation (16) shows that the basis vectors of the basic structure in the standard (primed) setting 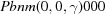 are obtained by a transformation of the basis vectors in the original (unprimed) setting 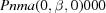 as 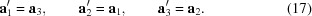
The fourth row of 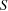 shows that the modulation wavevector remains the same, but its components with respect to the transformed basic structure reciprocal basis vectors are obtained by equation (8), 
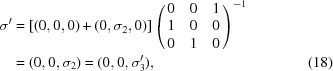
in accordance with the standard setting of superspace group No. 62.1.9.3.

### 3.2. Choice of the modulation wavevector

A second source of variation of settings is the freedom in the choice of the modulation wavevector. Given a modulation with modulation wavevector 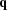, any reciprocal vector 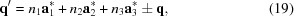
where 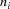 are integers is an appropriate choice for the modulation wavevector. A common choice is to select 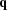 within the first Brillouin zone of the basic structure, *i.e.* to choose the shortest possible vector [equation (19)]. This choice does not necessarily correspond to the standard setting of the superspace group.

Transformations that change settings have been extensively discussed in van Smaalen (2007 ▶). The principles are illustrated by the symmetries of *A* 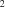 *BX* 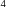 ferroelectric compounds with the 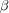-K
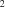
SO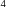 structure type and orthorhombic symmetry according to space group No. 62 (Hahn, 2002 ▶). Basic structures have been described in the standard setting 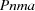 for some compounds, but the most frequently employed settings are 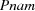 and 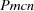 (Cummins, 1990 ▶).

K
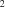
SeO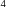 develops an incommensurate modulation below 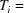 129.5 K with 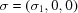 in the 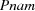 setting (Yamada & Ikeda, 1984 ▶). The incommensurate component is 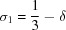 with 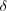 equal to a small positive number that depends on temperature. The superspace group is 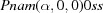. 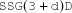 shows that this is an alternate setting of superspace group No. 62.1.9.6 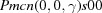, involving a transformation of basis vectors and the selection of an alternative modulation wavevector according to 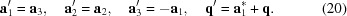
With respect to the transformed reciprocal basis vectors, the components of the modulation wavevector are [equation (8)] 
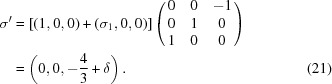
The transformed modulation wavevector has a negative component and a length larger than 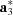, which might be considered an unfavorable situation. 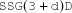 contains the tool 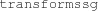, with which any user-specified transformation can be applied to the reciprocal basis vectors and modulation wavevectors. Employing this tool with [equation (20)] 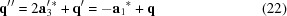
shows that a modulation wavevector with components [equation (8)] 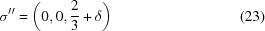
again represents the standard setting of superspace group No. 62.1.9.6 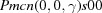. Alternatively, the transformation 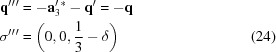
leads to a non-standard setting of superspace group No. 62.1.9.6.

The analysis of symmetry alone does not consider numerical values of lattice parameters or modulation wavevectors. Therefore, 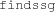 does not employ this information. Accordingly, it is impossible to give preference to one of the transformations of equation (20) or equation (22). Instead, the tool 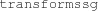 can be used for transformation to the desired values.

Rb
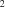
ZnCl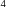 is incommensurately modulated below 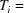 375 K with 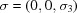 in the 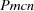 setting (Hogervorst, 1986 ▶). The incommensurate component is 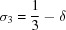 with 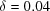 at room temperature. The superspace group is 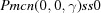. 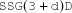 shows that this is another alternate setting of superspace group No. 62.1.9.6 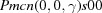. The transformation now only involves the choice of a different modulation wavevector: 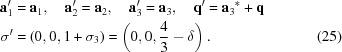
Like in the previous example, the transformation given by 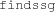 does not lead to the setting with the shortest possible modulation wavevector for the case of Rb
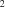
ZnCl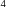. Employing 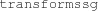 shows that the standard setting of superspace group No. 62.1.9.6 can also be obtained by the transformation of modulation wavevector 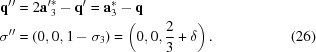
As discussed in van Smaalen (2007 ▶), replacement of 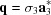 by 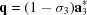 may change the apparently intrinsic translational component along the fourth coordinate for symmetry operators that possess a nonzero intrinsic translational component in the direction corresponding to the incommensurate component of the modulation wavevector. In the present example that is 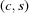 [mirror operation with intrinsic translation 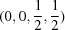] being replaced by 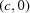 [mirror operation with intrinsic translation 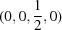]. These two settings of the superspace group correlate with different normal-mode descriptions of the same phase transition, for which it has been established that the modulation wavevector with 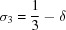 describes a distortion in terms of a 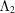 soft optical phonon, while 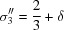 leads to the preferred description of the distortion in terms of a 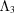 soft optical phonon (Axe *et al.*, 1986 ▶). It is well known that a change of setting will sometimes change the irreducible representation that contributes to a distortion without changing the physical distortion itself.

### 3.3. The supercentered setting

Aperiodic crystals are characterized by 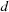 modulation wavevectors, each of which possesses at least one irrational component. According to symmetry, the values of these components can be viewed as variable rather than having specific irrational values. The other components are either zero or may assume rational values as allowed by the point symmetry. It is easily checked against the list of Bravais classes of 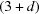
D superspace groups that the allowed rational components of modulation wavevectors are 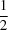 and 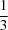 in the standard superspace-group settings, as well as nonzero integers in the case of BSGs based on a centered lattice of the basic structure. The modulation wavevector is usually separated into a rational part, with zeros and rational numbers as components, and an irrational part, with zeros and the variable components, according to 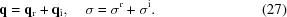



A modulation wavevector with nonzero rational components may naturally occur when a diffraction pattern of a modulated crystal is indexed, first determining the unit cell – centered if required – of the basic lattice, and then selecting an appropriate modulation wavevector, *e.g.* the shortest possible vector. However, rational components of 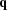 imply that the lattice in superspace is a centered lattice with the special feature that centering vectors contain nonzero components both along the fourth coordinate and along at least one of the three physical coordinates. Employing centered unit cells for centered lattices is common practice. It has several advantages in crystallographic analysis, facilitating the description of reflection conditions and the analysis of point symmetry. For superspace groups it has been denoted as the supercentered setting as opposed to the BSG setting, where a modulation wavevector with rational components is combined with the standard centered setting of the BSG (Stokes *et al.*, 2011*a* ▶). 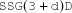 provides symmetry operators for both the BSG and supercentered settings (Stokes *et al.*, 2011*b* ▶).

**Figure 1:**
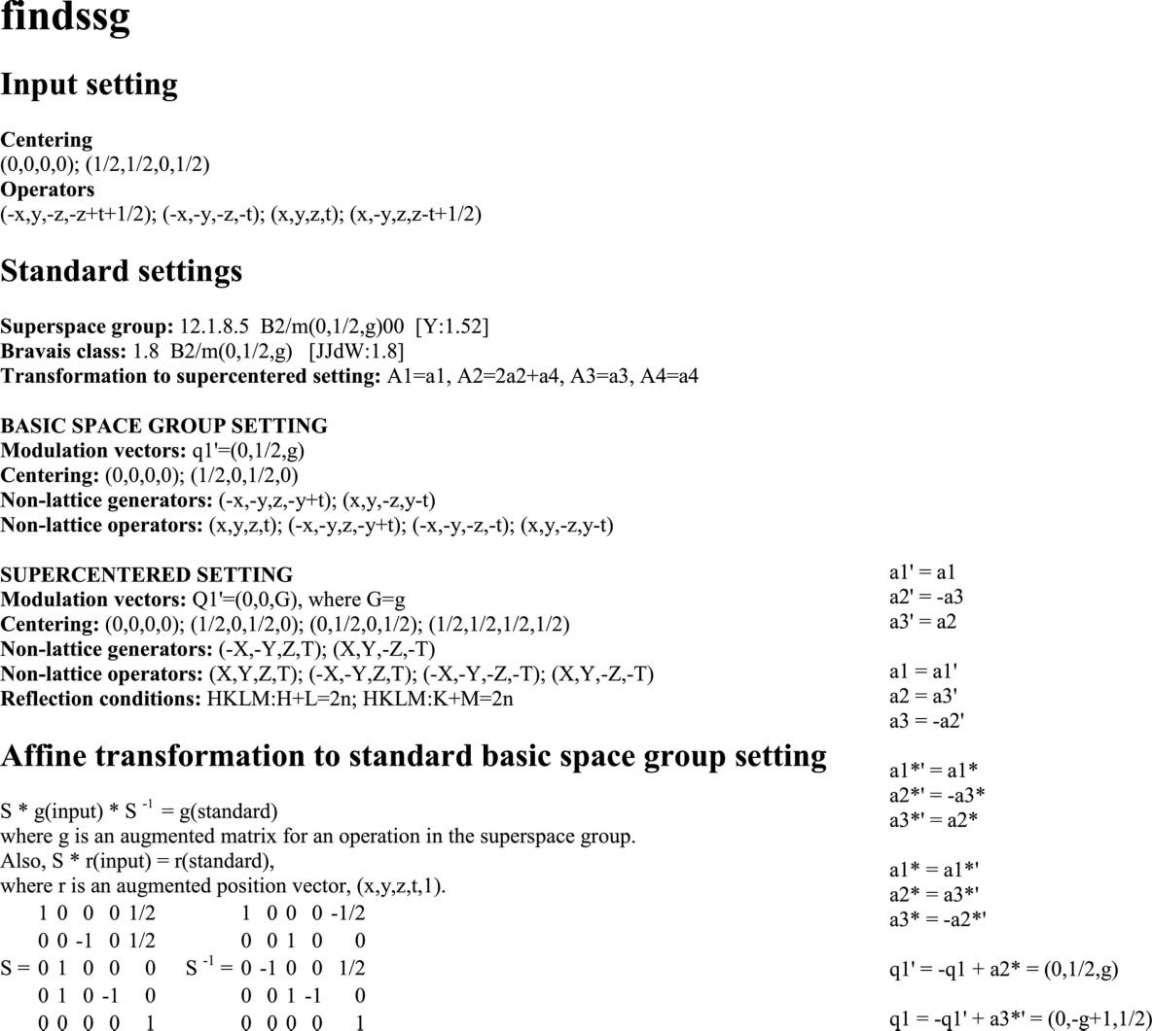
Output of 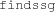 on 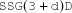, showing the equivalence of superspace group 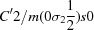 to superspace group No. 12.1.8.5 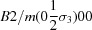.

As an example, consider blue bronze K
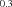
MoO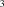. (Despite the decimal subscript in the usual form of the chemical formula, it possesses a fully ordered crystal structure with two formula units K
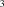
Mo
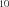
O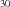 per unit cell.) Blue bronze develops an incommensurate charge-density wave (CDW) below 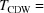 183 K with a modulation wavevector 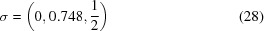
at 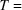 100 K in the setting of the high-temperature space group. Schutte & de Boer (1993 ▶) have determined the crystal structure of the incommensurate phase. With the modulation wavevector of equation (28), they obtained the 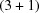
D superspace group 
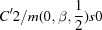
. This mixed setting contains the 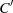 center 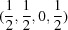, which possesses a nonzero component along the fourth coordinate (van Smaalen, 2007 ▶), and which is different from the BSG setting comprising the 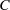 center 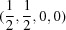. 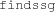 shows that 
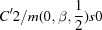
 is an alternate setting of superspace group No. 12.1.8.5 
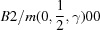
 (Table 1 ▶ and Fig. 1 ▶). In physical space, the transformation from the setting of Schutte & de Boer (1993 ▶) to the standard BSG setting involves a permutation of the unit-cell axes and the choice of a new modulation wavevector according to 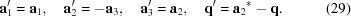
Furthermore, an origin shift of 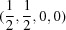 is required in order to bring the origin onto the operator 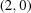 instead of 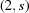. The components of 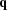 with respect to the transformed (primed) reciprocal basis vectors follow from equation (8) as [compare equation (21)] 
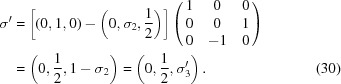
The standard supercentered setting is obtained from the standard BSG setting 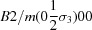 by the transformation of superspace basis vectors as given in 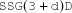, and corresponds to the following transformation of the physical-space basis vectors and modulation wavevector (capital letters indicate the supercentered setting): 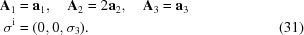
Observe that the modulation wavevector is purely irrational in the supercentered setting, as expected [equation (27)].

## 4. A plethora of settings

### 4.1. (3 + 2)-Dimensional superspace groups

#### 4.1.1. General features

Different settings of 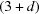
D superspace groups are obtained when different settings of the BSG are chosen (§3.1). For 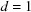, further settings result from the ambiguity in the choice of the modulation wavevector: all modulation wavevectors that differ by a reciprocal-lattice vector of the basic structure are equally valid [equation (19)] and may define different settings of a superspace group (§3.2). For 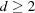, additional coordinate transformations involve the replacement of the modulation wavevectors by linear combinations of them.

Generalizing equation (7) shows that any set of reciprocal vectors (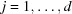) 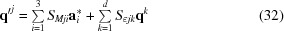
is an appropriate choice for the set of 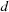 modulation wavevectors, where 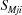 and 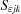 are integers and 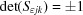. Such linear combinations of the modulation vectors of the standard setting are often necessary to make the description of experimental diffraction data simpler and more intuitive, but can also have the opposite effect if applied arbitrarily.

In analyzing superspace groups with 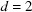, it is useful to distinguish between 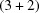
D and 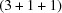
D superspace groups, where the latter refer to incommensurate crystals with two independent modulation waves, while the 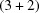
D superspace groups refer to crystals with two symmetry-related modulation waves, such as those in Bravais class No. 2.57 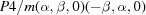.

As an example of a 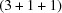
D superspace group, consider 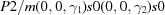. 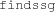 shows that this is an alternate setting of superspace group No. 10.2.5.6 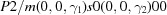. The transformation which brings the original setting into the standard BSG setting is 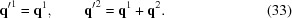
The difference between the two settings is that the operator 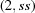 in one setting is equivalent to the operator 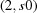 in the standard setting. The transformation modifies the intrinsic translation of a twofold axis along one of the superspace coordinates. This is a feature specific to transformations of the type of equation (33), while the modification of the modulation wavevector by a reciprocal-lattice vector of the basic structure [equation (19)] can only affect the intrinsic translations of symmetry operators that are screw axes or glide planes in three dimensions. More complicated linear combinations of modulation wavevectors may be required, as in the transformation between 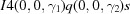 and the standard setting 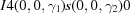 of superspace group No. 79.2.62.3 [equation (32)]: 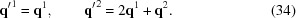
The same concept can be applied for reducing the number of rational components of the modulation wavevectors. 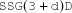 shows that superspace group 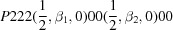 is an alternate setting of superspace group No. 16.2.19.3 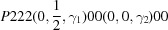 with 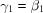 and 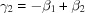. The transformation between these settings involves a linear combination of the two modulation wavevectors as well as a change of the setting of the BSG according to 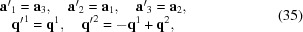
where again the primed vectors refer to the standard BSG setting.

#### 4.1.2. NbSe_3_

Several of the features discussed here are illustrated by the example of NbSe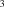. NbSe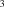 develops an incommensurate CDW below 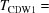 145 K. A second, independent CDW develops below 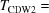 59 K, then resulting in an incommensurately modulated structure with two independent modulation waves, and with symmetry given by the 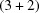
D superspace group No. 11.2.6.4 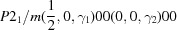 from 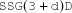. Inspection of the list of superspace groups shows that No. 11.2.6.4 is the only superspace group in its Bravais class that has BSG 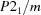. This implies that any possible combinations of nonzero intrinsic translations along the fourth and fifth coordinate axes are equivalent to the setting 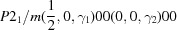 by a suitable transformation.

The modulated, low-temperature crystal structure of NbSe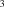 has been described in superspace group 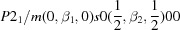 (unique 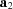 axis) with (van Smaalen *et al.*, 1992 ▶) 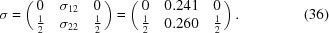
This setting naturally arises for the following choices:

(i) The BSG is equal to the space group of the periodic structure at ambient conditions, which has a unique axis 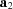 that is the preferred setting for monoclinic three-dimensional space groups.

(ii) The choice of axes 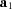 and 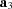 is that of the previously determined periodic crystal structure at ambient conditions.

(iii) Modulation wavevectors are chosen within the first Brillouin zone.

(iv) The first modulation wavevector is that of the first CDW and the second modulation wavevector applies to the second CDW.

All four choices need to be adapted, in order to arrive at the standard setting of this superspace group:

(i) The standard setting of the superspace group has incommensurate components of the modulation wavevectors along 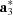, thus requiring a reordering of the basic structure axes.

(ii) Transforming the second modulation wavevector into a wavevector with one nonzero rational component requires a basic structure monoclinic unit cell that involves linear combinations of the axes 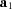 and 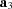 [compare to equation (1)]. Notice that this transformation does not affect the symbol of the BSG.

(iii) The transformation of 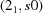 into 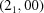 requires the transformation 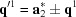 [equation (32)].

(iv) The standard setting requires interchanging the two modulation wavevectors.

**Figure 2:**
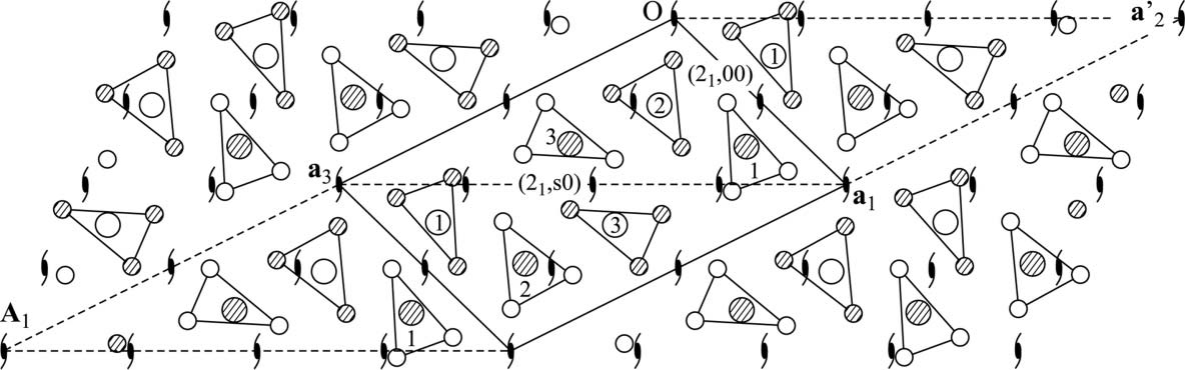
Projection of the basic structure of NbSe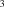. All atoms are in mirror planes. Hatched and open circles are atoms at 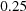 and 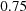 of the projected coordinate, respectively. Small circles are Se; large circles are Nb, with numbers indicating Nb1, Nb2 and Nb3 atoms. Symmetry operators 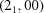 and 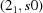 alternate in the supercentered setting. Unit cells are indicated for the published BSG setting (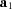, 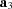; solid lines), the standard BSG setting [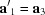, 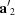; dashed lines; see equation (37)] and the supercentered setting [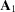, 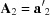; dashed lines; see equation (41)].

Altogether, the transformation from the published setting to the standard BSG setting of superspace group No. 11.2.6.4 is achieved by 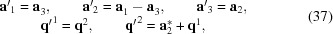
which implies a transformation of reciprocal basis vectors of the basic structure as 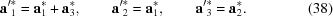
The components of the modulation wavevectors with respect to the transformed reciprocal basis vectors follow from equation (8) or by inspection of equations (37) and (38): 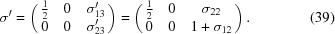
The tool 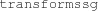 can be used to demonstrate that an alternate transformation, defined by a different choice of the second modulation wavevector, also leads to the standard BSG setting of superspace group No. 11.2.6.4:
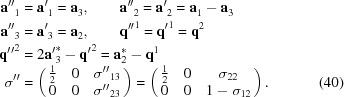
Choices (i), (ii) and (iv) are arbitrary – there does not appear to be a compelling reason to adhere to the standard setting except to establish the equivalence of different crystal structures. The choice (iii) of the modulation wavevector is related to the important question about the real wavevectors of the CDWs, which is not obvious because the incommensurate components of the modulation wavevectors can either be 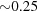 or 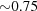, depending on the setting. This is most easily analyzed with the help of the supercentered setting, which follows from the standard BSG setting by the transformation 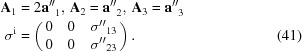
Structural analysis has shown that the first CDW (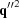 in the standard setting) is located on a pair of chains of niobium atoms, denoted as the Nb3 atoms, while the second CDW (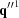 = 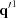) is located on a pair of chains of Nb1 atoms (Fig. 2 ▶). 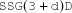 provides the explicit form of the symmetry operators in the supercentered setting. Employing these operators, one finds that the double chain of Nb1 atoms centered on 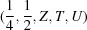 of the supercentered unit cell is located on the screw axis 
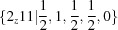
.^4^ This is a screw operator 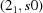 as it is generated by the combination of the screw 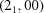 and the centering translation 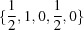. The pair of chains of Nb3 atoms is related by the operator 
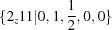
, which is a screw operator (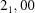). We judge that 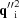 = 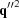 = 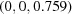 is the real wavevector of the Nb3 modulation, because an additional phase shift is not involved on application of this symmetry. On the other hand, the second wave with 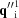 = (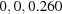) [equations (39) and (40)] implies symmetry for the pair of Nb1 chains involving a phase shift of one half. The real wavevector thus is 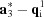 = 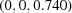, resulting in the setting 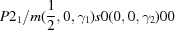 of superspace group No. 11.2.6.4. 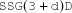 shows that the standard setting can be restored by a shift of the origin of 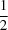 along 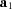. With this final transformation, the symmetry of NbSe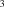 is described in the standard setting, and the components of the modulation wavevectors show that both CDWs are waves with wave­vectors of 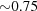 on their respective double chains of niobium atoms.

#### 4.1.3. Centerings in internal space

Table 2 ▶ compiles superspace groups for a series of compounds with two-dimensional modulations. Symbols for the superspace groups from the original publications encompass a disparate set of notations, including symbols based on the online database of 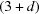
D superspace groups (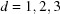) of Yamamoto (2005 ▶), as in the case of Ca
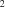
CoSi
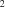
O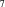, symbols based on Janner *et al.* (1983 ▶), as in the case of Mo
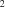
S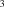, and symbols derived from these notations, such as replacing 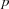 by 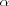 in the case of Sm
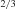
Cr
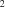
S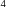, as well as other *ad hoc* symbols.

While for several compounds a permutation is required of the basis vectors of the basic structure unit cell in order to transform the published setting into the standard setting, other, less trivial transformations occur too. 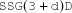 shows that the symmetry of tetrathiafulvalene tetracyanoquinodimethane (TTF TCNQ), 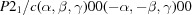, is based on a supercentered lattice, where the centering exclusively involves the two internal coordinates. The supercentered setting has modulation wavevectors 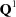 = 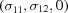 and 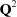 = 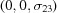 with 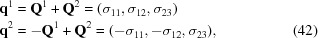
resulting in the reflection condition and corresponding centering translation: 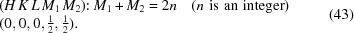
Monoclinic symmetry is the lowest symmetry where this kind of superspace lattice centering can occur. For Ca
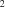
CoSi
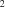
O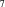, on the other hand, the superspace group 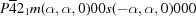 does not possess a centered lattice, despite the seemingly simpler modulation wavevectors 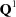 = 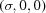 and 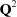 = 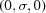, which are related to 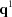 and 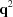 as in equation (42). The reason is that 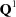 and 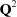 are related by symmetry in the same way as 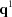 and 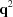 are, and the would-be supercentering does not have an advantage over the primitive lattice from the point of view of symmetry. Examples of supercentered lattices with higher symmetries are given for three-dimensional modulations in §4.2.

A peculiar feature of the modulation of TTF TCNQ is that one of the unrestricted components is not experimentally distinguishable from zero (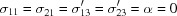). The explanation probably lies in the optimal phase relations between the CDWs on neighboring stacks of TTF or TCNQ molecules, as it is governed by the physics of CDW formation. However, in this case the phase relation is not reflected in the symmetry of the crystal structure. Similar observations can be made for Mo
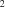
S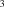 (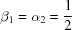 and 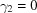), (Bi,Pb)
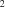
(Sr,Bi,Pb,Ca)
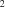
CuO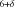 (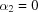) and LaSe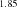 (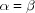). For these compounds, the special values of the components of the modulation wavevectors are reminiscent of the higher symmetries at high temperatures [monoclinic for Mo
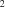
S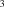 and orthorhombic for the high-*T*_c_ superconductor (Bi,Pb)
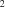
(Sr,Bi,Pb,Ca)
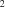
CuO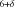] or the higher symmetry of a hypothetical basic structure (Laue symmetry 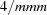 for LaSe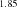).

**Figure 3:**
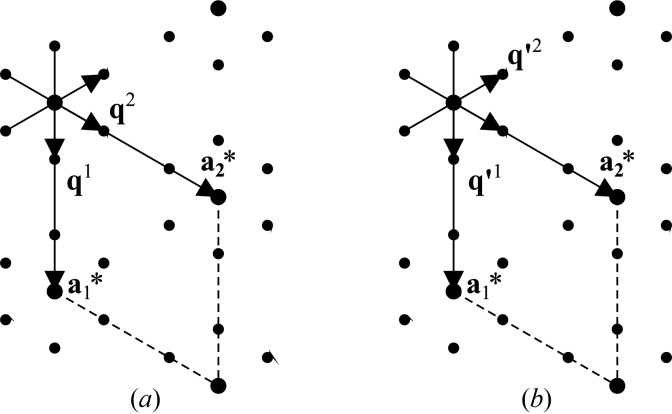
Reciprocal plane parallel to 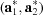 of a hexagonal lattice. (*a*) Indexing of satellite reflections with two modulation wavevectors enclosing an angle of 60°. (*b*) Preferred indexing with modulation wavevectors enclosing an angle of 120°. Notice that 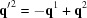.

The lattice type (primitive, centered BSG or supercentered) is the same for all superspace groups belonging to a Bravais class. Likewise, the choice of modulation wavevectors should be the same for all superspace groups within a single Bravais class: the Bravais class is defined by the point symmetry of the lattice together with the modulation wavevectors. A further requirement on the modulation wavevectors is that they must transform according to the three-dimensional point symmetry of the superspace group. These requirements become important for the selection of modulation wavevectors in the case of trigonal and hexagonal Bravais classes of 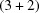
D superspace groups. Since all these Bravais classes contain superspace groups with acentric trigonal symmetry (Table 3 ▶), it is necessary to choose a pair of modulation wavevectors that enclose an angle of 120° and not 60° (Fig. 3 ▶). With the exception of the recent study on 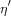-Cu
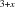
Si, this condition has not been obeyed in studies of the compounds with trigonal or hexagonal symmetries listed in Table 2 ▶, where the angle between 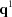 and 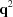 was chosen as 60°. While not wrong in these cases, it is highly preferable to describe these structures using an angle of 120° between the modulation wavevectors so as to be consistent with the settings of their Bravais classes.

### 4.2. (3 + 3)-Dimensional superspace groups

Different settings of 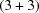
D superspace groups are obtained by means of the same degrees of freedom that apply to 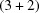
D superspace groups. That is, the setting of a 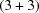
D superspace group depends on the choice of basic structure basis vectors and on the freedom in the choice of modulation wavevectors, including the possibility to replace the modulation wavevectors by linear combinations of them [equation (32)].

#### 4.2.1. Supercentered setting of (TaSe_4_)_2_I

(TaSe
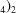
I has a periodic structure with space group 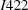 at ambient conditions. A CDW develops below 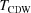 = 263 K. It is expressed in the diffraction by the presence of eight incommensurate satellite reflections around each main reflection, which can be indexed as first-order satellite reflections according to the four modulation wavevectors 
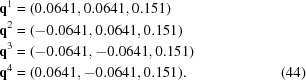
van Smaalen *et al.* (2001 ▶) incorrectly reported this as a four-dimensional modulation (Table 4 ▶), but then continued to show that the phase transition is accompanied by a lowering of the point symmetry and the formation of a multiply twinned crystal with a one-dimensional incommensurate modulation in each domain. Nevertheless, for the purpose of illustrating a fundamental issue of symmetry, we will proceed as though all modulation wavevectors would originate in a single domain, where the number of symmetry-equivalent modulation wavevectors is larger than the dimension of the modulation. The modulation in equation (44) is actually three-dimensional, because 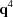 = 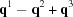. Despite this relationship between the modulation wavevectors, the tetragonal symmetry requires that modulation wavefunctions are symmetric in the four arguments 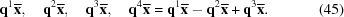
This symmetry becomes obvious in the supercentered setting, where 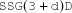 shows that there are two symmetry-equivalent modulation wavevectors, 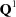 and 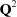, in addition to a third wavevector, 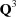, parallel to the tetragonal axis: 
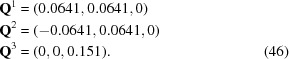
The four pairs of satellite reflections as well as the four equivalent arguments of the modulation wavefunctions then follow as all four equivalent linear combinations of 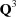 with 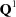 or 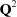: 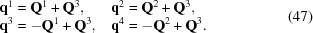
In accordance with the centering of the superspace lattice, diffracted satellite reflections do not appear at 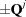 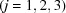, and modulation functions do not contain harmonics involving arguments 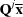, but only contain linear combinations like 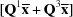 [equation (47)].

#### Superspace symmetry with BSG 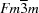


4.2.2.

All known compounds with a three-dimensional modulation possess cubic symmetry. Wustite, Fe
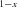
O, is based on an 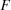-centered cubic lattice with BSG 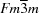 and the simple modulation with 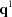 = 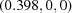 (Table 4 ▶). The superspace group is symmorphic and centerings other than the 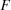-centering, 
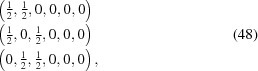
of the BSG do not occur.

Three compounds have been reported with symmetry according to superspace group No. 225.3.215.7 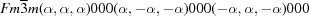. They have the same BSG as wustite but different modulation wavevectors. As with (TaSe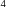)
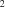
I (§4.2.1), four symmetry-equivalent modulation wavevectors exist. The supercentered setting clearly reveals the three-dimensional nature of the modulation with 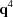 = 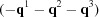 (Table 4 ▶), and 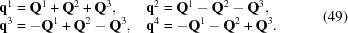
For Bi
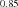
Mo
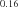
O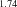 the modulation wavevectors of the supercentered setting are 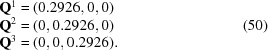
The centering translations of the supercentered setting combine the 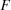-center of the basic structure [equation (48)] with a so-called ‘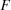-center’ among the internal superspace coordinates, the latter being defined as 
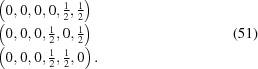
This can be compared with superspace group No. 225.3.212.5, based on modulation wavevectors of the type 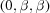, where the supercentered setting again involves modulation wavevectors of the type 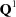 = 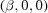 [equation (50)], but now combines the 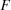-center of the basic structure [equation (48)] with an ‘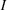-center’ among the internal superspace dimensions with centering translation 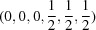.

Interestingly, replacing three-valent molybdenum atoms by five-valent niobium or tantalum atoms leads to a similar, but different structure involving mirror planes with nonzero intrinsic translational components along the internal superspace dimensions (Table 4 ▶).

#### 4.2.3. Modulation in the *I*-centered lattice of V_6_Ni_16_Si_7_

V
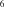
Ni
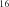
Si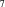 is a three-dimensionally modulated crystal with symmetry based on the cubic 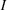-centered lattice and BSG 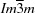 (Table 4 ▶). Withers *et al.* (1990 ▶) report an indexing of the electron diffraction based on the modulation wavevectors 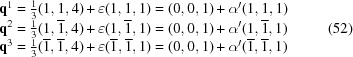
where 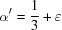 and 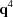 = 
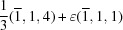
 = 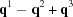.

Withers *et al.* (1990 ▶) also report the observed reflection conditions, but then provide an analysis based on the theory of irreducible representations (normal-mode analysis). Yamamoto (1993 ▶) has assigned to V
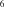
Ni
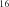
Si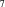 the 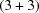
D superspace group with the tentative symbol 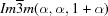.


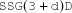
 shows that such a superspace group does not exist. Since symmetry operators are not provided by Withers *et al.* (1990 ▶) or Yamamoto (1993 ▶), we could not use the 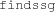 tool on 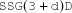 for computing the transformation to the standard setting. However, 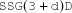 does show that the only possible modulation wavevectors for three-dimensional modulations with BSG 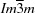 are 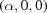, 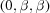 and 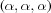 (Bravais classes 3.208, 3.211 and 3.214, respectively). Indeed, the modulation wavevectors can be rewritten as 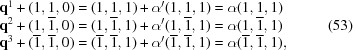
where 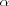 = 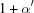 = 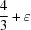. Notice that we cannot add the basic structure reciprocal vector 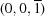 to the modulation wavevectors [equation (19)], because this is a forbidden reciprocal vector for the 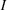-centered lattice. Instead, we have added the vector 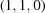 to 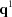 in order to arrive at a reciprocal vector along the diagonal of the cubic unit cell. Of course, this goes at the expense of a considerably increased length for the modulation wavevectors. Nevertheless, a description that respects the symmetry of the problem requires these long modulation wavevectors. With the new indexing, the non-symmorphic superspace group 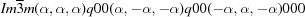 is obtained, which corresponds to No. 229.3.214.8 in 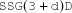 (Table 4 ▶).

## 5. Incommensurate composite crystals

Incommensurate composite crystals comprise two or more subsystems, each of which has an incommensurately modulated structure. The basic structures of the subsystems are mutually incommensurate, but for all known compounds, any pair of subsystems share a common reciprocal-lattice plane of their basic structures. The third reciprocal basis vector of one subsystem then acts as modulation wavevector for the other subsystem, and the other way around. The symmetry of a composite crystal is given by a 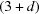
D superspace group, while the symmetry of each subsystem is also given by a 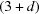
D superspace group. These so-called subsystem superspace groups often are different (inequivalent) groups according to the definition of superspace group employed in de Wolff *et al.* (1981 ▶) and Stokes *et al.* (2011*a* ▶).

**Figure 4:**
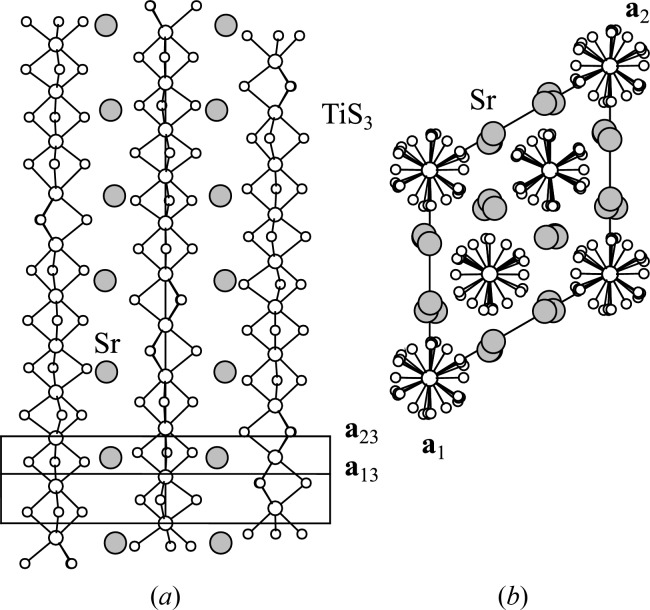
Crystal structure of [Sr]
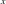
[TiS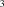]. (*a*) Projection showing the two types of columns with mutually incommensurate periodicities 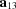 for the 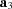 axis of the first subsystem (TiS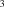) and 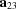 for the 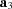 axis of the second subsystem (TiS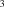). (*b*) Projection along the mutually incommensurate direction showing the common basal plane of the hexagonal lattice. Large circles denote metal atoms, small circles represent sulfur atoms. Reprinted from Figs. 1.4(*c*) and 1.4(*d*) in van Smaalen (2007 ▶) by permission of Oxford University Press (http://www.oup.com).

The various aspects of the structures and symmetries of composite crystals are illustrated by the example of [Sr]
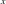
[TiS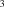] (Onoda *et al.*, 1993 ▶), where square brackets indicate the subsystems. The seemingly non-stoichiometric composition with 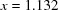 reflects the incommensurate ratio of the volumes of the basic structure unit cells of subsystem 1 (TiS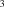) and subsystem 2 (Sr). [Sr]
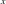
[TiS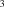] is a composite crystal of the columnar type, where chains of Sr atoms and columns of TiS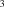 are alternatingly arranged on a two-dimensional hexagonal lattice (Fig. 4 ▶). The basic structure reciprocal lattices share the basis vectors in the basal plane, while the third direction (parallel to the chains) is the incommensurate direction: 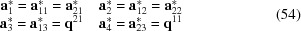
where 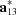, for example, denotes the third reciprocal basis vector of the first subsystem and 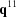 is the first (and in this example only) modulation wavevector of the first subsystem with 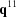 = 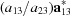.

An indexing of all reflections with four integers is obtained with the four reciprocal basis vectors 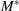 = 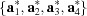. Along with its modulation wavevector, the reciprocal basis vectors of subsystem 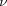 (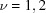) are obtained from the four reciprocal vectors 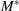 by a 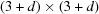 integer matrix 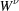 (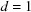 in the present example) according to 
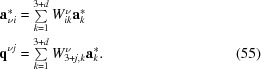
The matrices 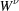 extract the basic structure reciprocal basis vectors and modulation wavevectors of subsystem 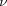 from the basis vectors used for indexing. In this sense, 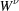 represents a coordinate transformation in superspace between the arbitrarily chosen superspace representation 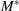 and the natural subsystem superspace, which is specific to each subsystem. Operators of the subsystem superspace group follow as (van Smaalen, 1991 ▶) 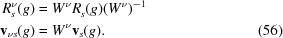
Because reciprocal basis vectors of one subsystem act as modulation wavevectors of the other subsystem, 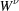 must be a coordinate transformation that mixes the first three dimensions and the additional dimensions for at least some of the subsystems. This coordinate transformation is a forbidden transformation when establishing the equivalence of superspace groups (Stokes *et al.*, 2011*a* ▶). Therefore, the subsystem superspace groups are generally inequivalent, unless they are equivalent by chance, as is the case for the mineral levyclaudite which possesses triclinic symmetry (Evain *et al.*, 2006 ▶).

For [Sr]
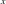
[TiS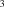] equation (54) shows that 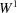 is the identity matrix. This choice of 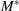 has become a *de facto* standard for composite crystals. It implies a setting where the symmetry of [Sr]
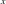
[TiS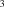] and the symmetry of the first subsystem are described by the same superspace group. Onoda *et al.* (1993 ▶) give the superspace group 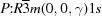, which is found to be an alternate symbol for superspace group No. 166.1.22.2, 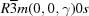 on 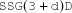. Apart from the 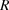-centering of the hexagonal basic structure unit cell, other centerings in superspace do not exist for this lattice.

Equation (54) leads for the second subsystem to 
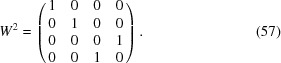
The 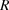-centering of the original setting transforms by 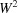 [equation (56)] into the superspace centering vectors 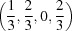 and 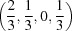, which represent an 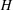-type centering of the BSG and which has been denoted as the 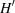-centering of the superspace lattice (van Smaalen, 2007 ▶). 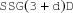 shows that the transformation by 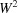 [equation (57)] leads to the supercentered setting of the 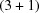
D superspace group No. 163.1.23.1, 
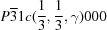
.

**Figure 5:**
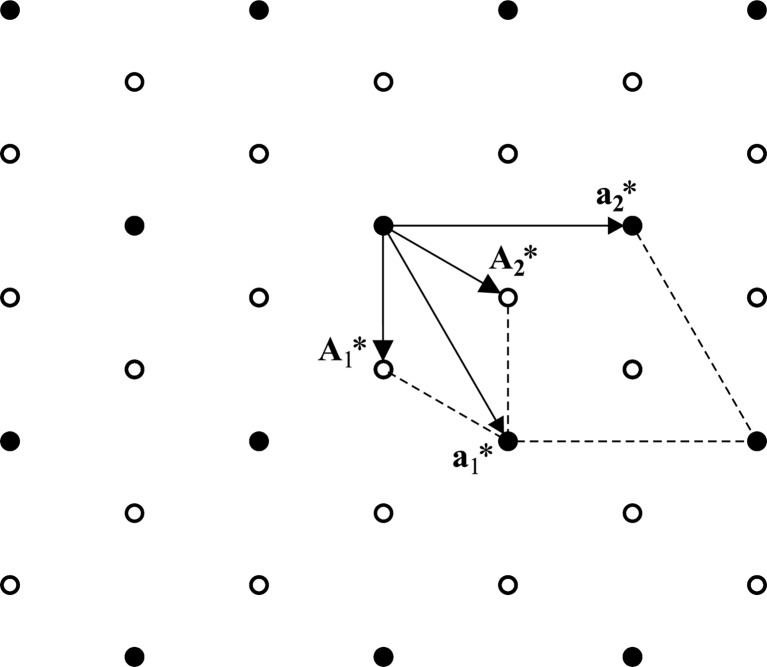
Basal plane of the reciprocal lattice of [Sr]
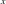
[TiS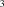]. 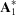 and 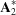 are the reciprocal basis vectors of the BSG setting of the rhombohedral lattice. Filled circles represent Bragg reflections and open circles are the positions of extinct Bragg reflections. 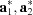 are the reciprocal-lattice vectors of the trigonal unit cell in the standard (primitive) BSG setting. Extinct Bragg reflections (open circles) only apply to this lattice if the supercentered setting (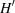 setting) is used.

The subsystem superspace groups of [Sr]
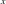
[TiS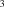] turn out to be inequivalent 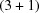
D superspace groups, although they are of course equivalent as 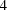
D space groups as governed by the coordinate transformation 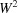. The case of [Sr]
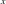
[TiS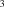] is special as it combines different Bravais lattices of the BSG for the subsystems. The rhombohedral lattice with an 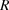-centering and the primitive trigonal space group described with an 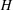-centered unit cell can be considered as different centerings of the hexagonal unit cell, which share a common reciprocal-lattice plane perpendicular to the trigonal axis (Fig. 5 ▶).

Other incommensurate composite crystals, including misfit layer sulfides (Wiegers, 1996 ▶), misfit layer cobalt oxides (Isobe *et al.*, 2007 ▶) and urea inclusion compounds (van Smaalen & Harris, 1996 ▶), also exhibit a pairing of two inequivalent superspace groups. A detailed analysis of this feature is outside the scope of the present overview and will not be discussed further here.

Standard settings and alternate settings of superspace groups occur for incommensurate composite crystals by means of the same kinds of coordinate transformations as have been discussed for modulated crystals. One difference is the stronger inclination for employing non-standard settings in the case of composite crystals, because the set 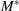 of reciprocal basis vectors is preferably chosen to contain reciprocal basis vectors of the basic structures of the subsystems. For example, for the case of [PbS]
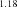
[TiS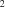] and isostructural [Ca
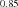
OH]
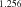
[CoO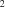] (van Smaalen *et al.*, 1991 ▶; Isobe *et al.*, 2007 ▶), 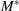 has been chosen as 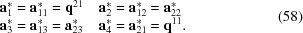
This results in a mixed setting of the 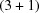
D superspace group as 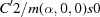 with 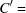 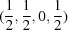. 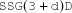 shows that this is an alternate setting of superspace group No. 12.1.7.4 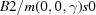. Apart from the trivial transformation of the setting of the BSG, the transformation toward the standard BSG setting involves the choice of an alternate modulation wavevector: 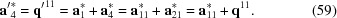
The interpretation of 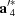 as a reciprocal basis vector of the second subsystem is lost in this representation [equation (59)]. Therefore, the mixed setting (*i.e.* not BSG setting or supercentered setting) with centering translation 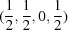 is preferred over the standard setting in the case of these composite crystals.

## 6. Chiral superspace groups

Chiral space groups are space groups that may be the symmetry of crystals containing chiral molecules. They are of particular importance in the life sciences, because all proteins and nucleotides are molecules of this type (Lovelace *et al.*, 2008 ▶).

Chiral space groups are those space groups of which the point group contains rotations only (Blow, 2002 ▶). Chiral superspace groups are then defined as the superspace groups for which the three-dimensional point group of the BSG contains rotations only (Souvignier, 2003 ▶). A list of 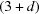
D superspace groups 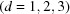 has been generated with this criterion and is available on 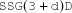. It is noticed that the fraction of superspace groups that is chiral strongly decreases on increasing superspace dimension 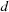 (Table 5 ▶). We did not find a compelling theoretical reason for this feature. But we do observe that the number of ways to combine the intrinsic translations of the BSG with the intrinsic translations along the additional superspace dimensions, or with supercentering translations, increases with 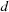. So it appears that the intrinsic translations of chiral BSG operations are more restricted in the combinations in which they can participate.

Superspace groups are defined on the basis of equivalence relations that only allow coordinate transformations that preserve the handedness of the coordinate axes in three-dimensional space [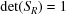; equation (7)], *i.e.* that preserve chirality. This definition leads to pairs of enantiomorphic superspace groups in cases where the BSG is an enantiomorphic space group, like the 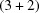
D superspace groups No. 76.2.60.2 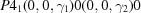 and No. 78.2.60.2 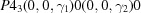. Intrinsic translations along the additional superspace dimensions do not give rise to enantiomorphic superspace groups. For example, superspace group No. 75.2.60.4 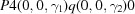 is not enantiomorphic (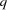 stands for the fractional translation 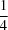). Instead of being an enantiomorph, 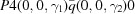 is an alternative setting of No. 75.2.60.4, and is transformed into the standard setting by the choice of a different modulation wavevector: 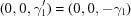 [equation (19)].

## 7. Conclusions

The computational complexity of finding the transformation between two settings of a 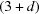
D superspace group is surprisingly high, especially for 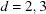. Here an efficient algorithm is presented, which either establishes two superspace groups to be different superspace groups or determines them to be different settings of the same superspace group and then provides the transformation between these settings. The algorithm has been implemented as an internet-based utility called ‘
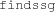
’, which identifies any user-given 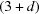
D superspace group (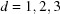) based on the superspace-group operators provided, and displays the transformation to the standard setting of this superspace group in the 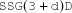 tables.

The algorithm considers coordinate transformations in superspace. It is shown that in general such a transformation corresponds to one, or a combination, of the following three types of transformations in physical space:

(i) A transformation of the basic structure unit cell.

(ii) Adding any reciprocal-lattice vector of the basic structure to the modulation wavevector [equation (19)].

(iii) Replacing originally chosen modulation wavevectors by linear combinations of the same [only for 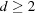; equation (32)].

These transformations are illustrated by the analysis of the symmetries of a series of compounds with 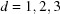, comparing published and standard settings and discussing the transformations between them. It is argued that non-standard settings are needed in some cases, while standard settings of superspace groups are desirable in other cases. A compilation is provided of standard settings of compounds with two- and three-dimensional modulations (Tables 2 ▶ and 4 ▶). It appears that several *ad hoc* notations have been used in the literature for 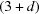
D superspace groups, especially for 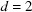 and 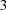.

For 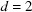 superspace groups with trigonal/hexagonal symmetry, an angle of 120° between the two modulation wavevectors is preferred and is the only correct choice for acentric trigonal cases (Table 3 ▶). This is the standard setting for all relevant Bravais classes in 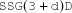, in contrast to the use of a 60° angle in most published structures (§4.1.3 and Table 2 ▶).

The problem of superspace-group settings, including the choice of origin, is subtle. Therefore, we strongly advise authors to explicitly document for each structure the list of symmetry operators (or at least the generators) of the superspace group, along with the explicit form of the modulation wavevectors as in equation (2). It would also be useful to include the number and symbol of the standard setting on 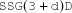 for each structure, because this will make it easier to check the equivalences of structures and symmetries in future studies.

## Supplementary Material

Output of SSG(3+d)D for different settings of superspace groups. DOI: 10.1107/S0108767312041657/pc5018sup1.zip

Supplementary material file. DOI: 10.1107/S0108767312041657/pc5018sup2.pdf

## Figures and Tables

**Table 1:** Symmetry operators of superspace group No. 12.1.8.5 in mixed, BSG and supercentered settings The second column refers to the mixed setting chosen by Schutte & de Boer (1993 ▶) [who incorrectly give the symbol 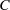 for the centering translation 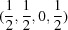] with the corresponding supercentered setting in the third column, featuring the tentative symbols 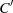 and 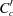 for the centerings according to Table 3.9 in van Smaalen (2007 ▶). The fourth and fifth columns give the standard BSG and standard supercentered settings as provided by 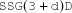. The notation of symmetry operators follows 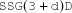, where 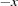 has been replaced by 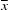 and similar.

Symmetry operator	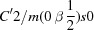	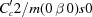	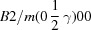	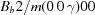
Centering	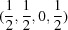	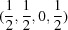	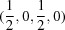	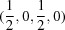
		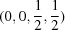		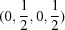
		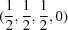		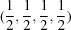
Identity	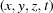	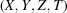	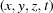	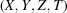
Twofold rotation	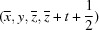	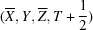	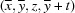	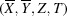
Inversion	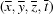	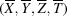	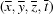	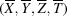
Mirror	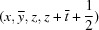	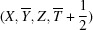	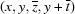	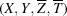

**Table 2:** Superspace groups for incommensurate compounds with two-dimensional modulations Given are the published modulation wavevectors and superspace-group symbols, the number and symbol of the standard BSG setting of the superspace group in 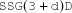, and the transformation of the published basic structure unit cell to the standard BSG setting as well as the modulation wavevectors of the latter.

			Published	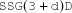	Superspace-group symbols for	Standard BSG setting	
Compound	Note	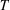 (K)	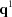 / 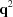	No.	published/standard BSG settings	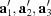	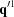 / 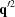
Mo 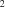 S 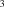	(*a*)	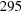	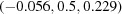	2.2.1.1	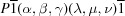	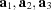	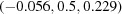
			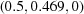		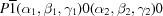		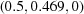
(Bi,Pb)_2_(Sr,Bi,Pb,Ca)_2_-	(*b*)	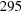	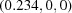	9.2.4.1	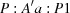	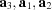	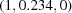
CuO 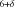			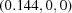		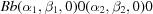		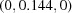
NbSe 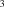	(*c*)	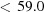	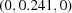	11.2.6.4	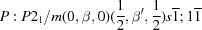	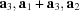	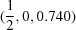
			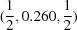		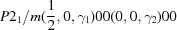		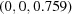
TTF TCNQ	(*d*)	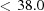	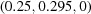	14.2.16.6	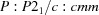	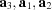	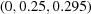
			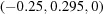		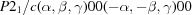		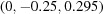
(PO 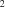 ) 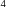 (WO 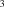 ) 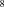	(*e*)	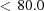	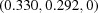	19.2.50.3	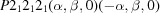	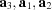	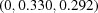
			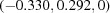		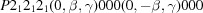		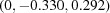
Sm 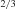 Cr 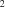 S 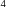	(*f*)	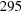	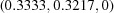	62.2.50.22	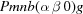 m¨*g*	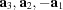	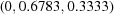
			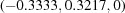		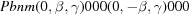		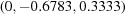
GdS 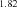	(*g*)	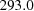	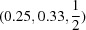	85.2.58.2	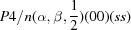	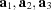	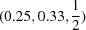
			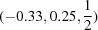		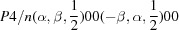		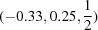
LaSe 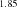	(*h*)	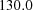	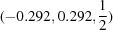	85.2.58.2	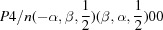	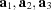	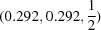
			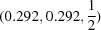		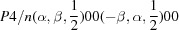		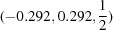
Ba 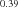 Sr 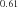 Nb 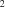 O 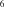	(*i*)	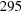	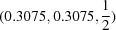	100.2.69.13	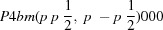	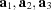	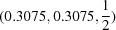
			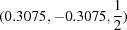		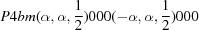		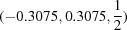
Sr 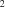 TiSi 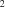 O 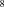	(*j*)	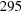	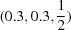	100.2.69.14	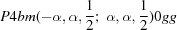	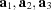	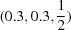
			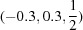		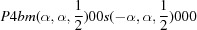		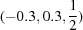
Ca 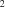 CoSi 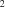 O 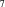	(*k*)	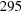	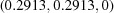	113.2.68.6	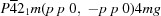	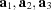	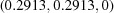
			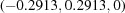		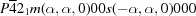		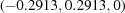
(Sr 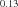 Ca 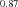 ) 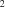 CoSi 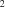 O 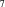	(*l*)	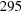	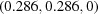	113.2.68.6	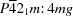	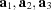	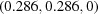
			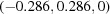		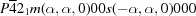		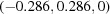
CaNdGa 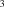 O 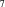	(*m*)	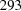	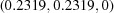	113.2.68.6	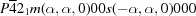	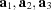	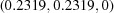
			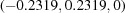		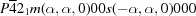		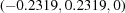
CaLaGa 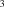 O 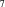	(*n*)	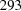	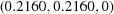	113.2.68.6	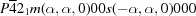	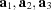	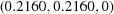
			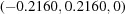		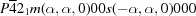		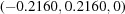
Ni 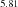 SnTe 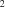	(*o*)	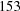	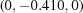	139.2.67.7	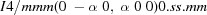	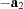 , 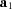 , 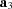	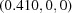
			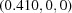		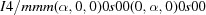		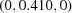
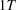 -TaS 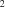	(*p*)	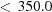	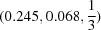	147.2.72.1	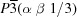	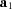 , 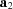 , 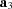	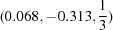
			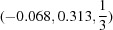		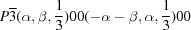		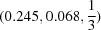
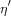 -Cu 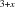 Si	(*q*)	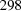	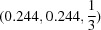	162.2.76.3	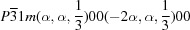	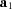 , 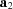 , 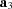	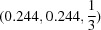
			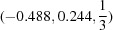		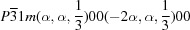		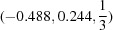
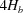 -TaSe 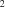	(*r*)	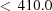	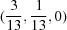	176.2.80.1	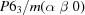	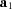 , 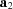 , 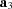	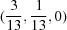
			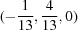		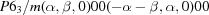		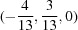
Ag 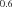 NbS 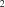	(*s*)	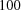	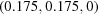	186.2.83.4	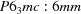	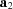 , 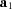 , 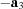	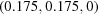
			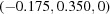		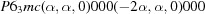		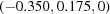
Au 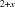 Cd 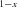	(*t*)	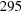	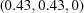	194.2.83.4	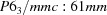	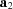 , 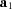 , 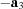	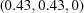
			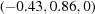		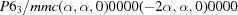		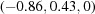
Cu 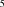 Sb	(*u*)	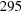	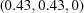	194.2.83.4	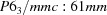	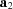 , 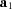 , 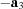	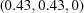
			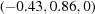		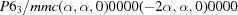		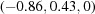

*Notes*: (*a*) Schutte *et al.* (1993 ▶). (*b*) Gao *et al.* (1990 ▶). (*c*) van Smaalen *et al.* (1992 ▶). (*d*) TTF (tetrathiafulvalene) and TCNQ (tetracyanoquinodimethane), Bouveret & Megtert (1989 ▶). (*e*) Ludecke *et al.* (2001 ▶); an extensive review of basic structures and CDW transitions of the phosphate bronzes (PO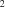)
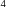
(WO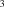)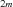 (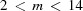) is given by Roussel *et al.* (2000 ▶). (*f*) Lafond *et al.* (1996 ▶). (*g*) Tamazyan *et al.* (2003 ▶). (*h*) Graf & Doert (2009 ▶). (*i*) Woike *et al.* (2003 ▶). (*j*) Höche *et al.* (2003 ▶). (*k*) Hagiya *et al.* (1993 ▶). (*l*) Bagautdinov *et al.* (2000 ▶). (*m*) Wei *et al.* (2011 ▶). (*n*) Wei *et al.* (2012 ▶). (*o*) Isaeva *et al.* (2007 ▶). (*p*) Yamamoto *et al.* (1990 ▶) and Spijkerman *et al.* (1997 ▶). (*q*) Palatinus *et al.* (2011 ▶). (*r*) Ludecke *et al.* (1999 ▶). (*s*) van der Lee *et al.* (1991 ▶). (*t*) Yamamoto (1983 ▶). (*u*) Motai *et al.* (1993 ▶).

**Table 3:** Selected 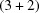
D superspace groups with acentric trigonal symmetry Other superspace groups exist that differ in the intrinsic translations.

No.	Superspace-group symbol
143.2.72.1	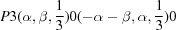
149.2.76.3	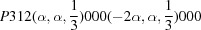
150.2.78.1	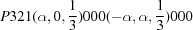
143.2.80.4	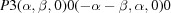
149.2.82.6	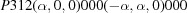
150.2.82.4	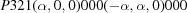
149.2.83.7	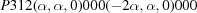
150.2.83.5	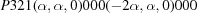

**Table 4:** Superspace groups for incommensurate compounds with three-dimensional modulations Given are the published modulation wavevectors and superspace-group symbol, the number and symbol of the standard BSG setting of the superspace group in 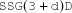 and the modulation wavevectors in the standard BSG setting. The published and standard basic structure unit cells are equal to each other: 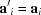 (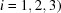.

				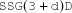	Superspace-group symbols for	Standard BSG setting
Compound	Note	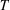 (K)	Published 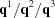	No.	published/standard BSG settings	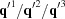
(TaSe 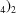 I	(*a*)	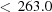	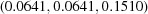	97.3.179.24	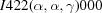	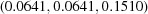
			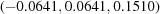		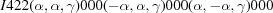	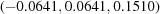
			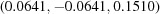			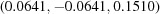
Fe 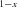 O	(*b*)	295	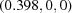	225.3.209.1	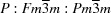	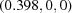
			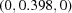		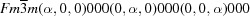	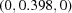
			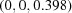			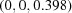
None	(*c*)		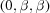	225.3.212.5	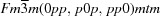	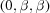
			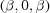		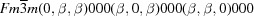	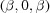
			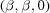			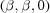
Cu 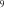 BiS 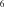	(*d*)	295	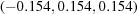	225.3.215.7	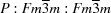	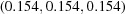
			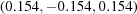		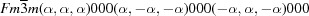	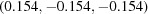
			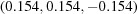			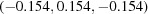
Bi 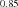 Cr 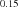 O 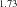	(*e*)	295	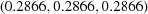	225.3.215.7	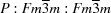	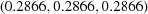
			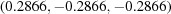		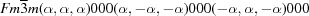	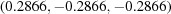
			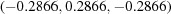			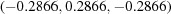
Bi 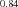 Mo 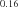 O 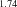	(*f*)	295	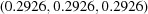	225.3.215.7	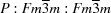	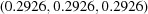
			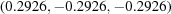		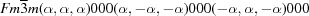	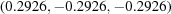
			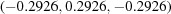			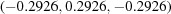
Bi 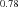 Nb 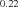 O 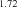	(*g*)	295	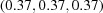	225.3.215.8	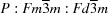	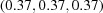
			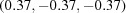		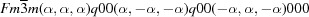	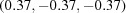
			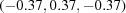			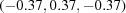
Bi 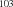 Ta 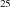 O 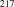	(*h*)	295	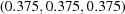	225.3.215.8	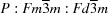	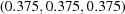
			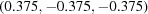		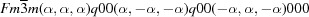	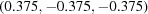
			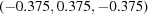			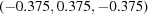
BaBi 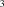 O 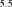	(*i*)	295	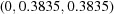	229.3.211.5	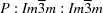	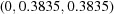
			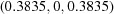		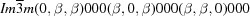	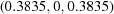
			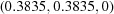			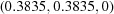
V 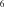 Ni 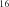 Si 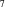	(*j*)	295	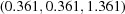	229.3.214.8	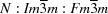	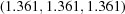
			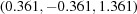		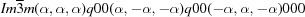	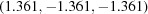
			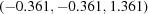			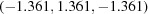

*Notes*: (*a*) van Smaalen *et al.* (2001 ▶) incorrectly reported a 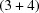
D superspace group 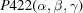. (*b*) Wustite, 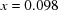; Yamamoto (1982 ▶). (*c*) Provided for purposes of comparison with the other groups with BSG 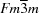; ‘published’ setting is the symbol from Yamamoto (2005 ▶). (*d*) Ohmasa *et al.* (1995 ▶). (*e*) Esmaeilzadeh *et al.* (2001 ▶) only discuss the supercentered setting. They give two more compositions: Bi
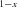
Cr
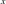
O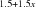 with 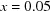 (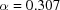) and 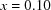 (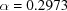). (*f*) Valldor *et al.* (2000 ▶). (*g*) Withers *et al.* (1999 ▶): Bi
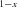
Nb
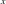
O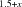 (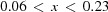). (*h*) Ling *et al.* (1998 ▶): Bi
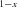
Ta
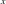
O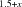 (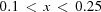). (*i*) Esmaeilzadeh *et al.* (2000 ▶). (*j*) Withers *et al.* (1990 ▶) and Yamamoto (1993 ▶).

**Table 5:** Number of chiral superspace groups in comparison to the number of superspace groups

	Dimension of space or superspace			
Classification	3	3 + 1	3 + 2	3 + 3
Bravais classes	14	24	83	215
Superspace groups	230	775	3338	12584
Chiral superspace groups	65	135	368	1019
Fraction that is chiral	0.283	0.174	0.110	0.081

## Footnotes

1 The mathematically correct designation is that of equivalent space groups providing different settings of space-group type No. .

2 Supplementary material for this paper is available from the IUCr electronic archives (Reference: PC5018). Services for accessing this material are described at the back of the journal.

3 The modulated phase often appears as a twinned crystal if the BSG is a subgroup of the three-dimensional space group of the periodic phase (van Smaalen, 2007 ▶).

4 The symbol  indicates an operator with a twofold rotation  of the BSG along  combined with a unit  matrix , and followed by a superspace translation .
