# Supplementary material for: Equivalence of superspace groups
Source: Acta Crystallogr A. 2012 Nov 14;69(Pt 1):75–90. doi: 10.1107/S0108767312041657 (PMC3553647; doi:10.1107/S0108767312041657)
Supplement: Supplementary file 1 [file a-69-00075-sup1.zip › ssg1d_c2_m_k03moo3.pdf]

12.1.8.5 B2/m(0 ½ σ_3)00

Superspace group: **12.1.8.5 B2/m(0,1/2,g)00 [Y:1.52]**
Bravais class: **1.8 B2/m(0,1/2,g) [JJdW:1.8]**
Transformation to supercentered setting: **A1=a1, A2=2a2+a4, A3=a3, A4=a4**

BASIC SPACE GROUP SETTING
Modulation vectors: **q1=(0,1/2,g)**
Centering: **(0,0,0,0); (1/2,0,1/2,0)**
Non-lattice generators: **(-x,-y,z,-y+t); (x,y,-z,y-t)**
Non-lattice operators: **(x,y,z,t); (-x,-y,z,-y+t); (-x,-y,-z,-t); (x,y,-z,y-t)**

SUPERCENTERED SETTING
Modulation vectors: **Q1=(0,0,G), where G=g**
Centering: **(0,0,0,0); (1/2,0,1/2,0); (0,1/2,0,1/2); (1/2,1/2,1/2,1/2)**
Non-lattice generators: **(-X,-Y,Z,T); (X,Y,-Z,-T)**
Non-lattice operators: **(X,Y,Z,T); (-X,-Y,Z,T); (-X,-Y,-Z,-T); (X,Y,-Z,-T)**
Reflection conditions: **HKLM:H+L=2n; HKLM:K+M=2n**

This is the superspace group of the incommensurately modulated CDW state of blue bronze $K_{0.3}MoO_3$.

findssg B2/m(0 ½ σ_3)00

The operators of the standard BSG setting have been given as input to findssg. The S matrix is the identiy matrix, as it should be.

Input setting

Centering
(0,0,0,0); (1/2,0,1/2,0)
Operators
(-x,-y,z,-y+t); (x,y,-z,y-t); (x,y,z,t); (-x,-y,-z,-t)

Standard settings

Superspace group: 12.1.8.5 B2/m(0,1/2,g)00 [Y:1.52]
Bravais class: 1.8 B2/m(0,1/2,g) [JJdW:1.8]
Transformation to supercentered setting: A1=a1, A2=2a2+a4, A3=a3, A4=a4

BASIC SPACE GROUP SETTING
Modulation vectors: q1'=(0,1/2,g)
Centering: (0,0,0,0); (1/2,0,1/2,0)
Non-lattice generators: (-x,-y,z,-y+t); (x,y,-z,y-t)
Non-lattice operators: (x,y,z,t); (-x,-y,z,-y+t); (-x,-y,-z,-t); (x,y,-z,y-t)

SUPERCENTERED SETTING
Modulation vectors: Q1'=(0,0,G), where G=g
Centering: (0,0,0,0); (1/2,0,1/2,0); (0,1/2,0,1/2); (1/2,1/2,1/2,1/2)
Non-lattice generators: (-X,-Y,Z,T); (X,Y,-Z,-T)
Non-lattice operators: (X,Y,Z,T); (-X,-Y,Z,T); (-X,-Y,-Z,-T); (X,Y,-Z,-T)
Reflection conditions: HKLM:H+L=2n; HKLM:K+M=2n

Affine transformation to standard basic space group setting

S * g(input) * S $^{-1}$ = g(standard),
where g is an augmented matrix for an operation in the superspace group.
Also, S * r(input) = r(standard),
where r is an augmented position vector, (x,y,z,t,1).

$$S = \begin{pmatrix} 1&0&0&0&0\\ 0&1&0&0&0\\ 0&0&1&0&0\\ 0&0&0&1&0\\ 0&0&0&0&1 \end{pmatrix} \quad S^{-1} = \begin{pmatrix} 1&0&0&0&0\\ 0&1&0&0&0\\ 0&0&1&0&0\\ 0&0&0&1&0\\ 0&0&0&0&1 \end{pmatrix}$$

a1' = a1
a2' = a2

a3' = a3

a1 = a1'
a2 = a2'
a3 = a3'

a1*' = a1*
a2*' = a2*
a3*' = a3*

a1* = a1*'
a2* = a2*'
a3* = a3*'

q1' = q1 = (0,1/2,g)

q1 = q1' = (0,1/2,g)

findssg B2/m(0 ½ σ_3)s0

The operators of an alternate BSG setting have been given as input to findssg (2,s instead of 2,0 operator). The transformation is a pure origin shift.

Input setting

Centering
(0,0,0,0); (1/2,0,1/2,0)
Operators
(-x,-y,z,-y+t+1/2); (x,y,-z,y-t); (x,y,z,t); (-x,-y,-z,-t+1/2)

Standard settings

Superspace group: 12.1.8.5 B2/m(0,1/2,g)00 [Y:1.52]
Bravais class: 1.8 B2/m(0,1/2,g) [JJdW:1.8]
Transformation to supercentered setting: A1=a1, A2=2a2+a4, A3=a3, A4=a4

BASIC SPACE GROUP SETTING
Modulation vectors: q1'=(0,1/2,g)
Centering: (0,0,0,0); (1/2,0,1/2,0)
Non-lattice generators: (-x,-y,z,-y+t); (x,y,-z,y-t)
Non-lattice operators: (x,y,z,t); (-x,-y,z,-y+t); (-x,-y,-z,-t); (x,y,-z,y-t)

SUPERCENTERED SETTING
Modulation vectors: Q1'=(0,0,G), where G=g
Centering: (0,0,0,0); (1/2,0,1/2,0); (0,1/2,0,1/2); (1/2,1/2,1/2,1/2)
Non-lattice generators: (-X,-Y,Z,T); (X,Y,-Z,-T)
Non-lattice operators: (X,Y,Z,T); (-X,-Y,Z,T); (-X,-Y,-Z,-T); (X,Y,-Z,-T)
Reflection conditions: HKLM:H+L=2n; HKLM:K+M=2n

Affine transformation to standard basic space group setting

$S * g(\text{input}) * S^{-1} = g(\text{standard})$,
where g is an augmented matrix for an operation in the superspace group.
Also, S * r(input) = r(standard),
where r is an augmented position vector, (x,y,z,t,1).

$$S = \begin{pmatrix} 1 & 0 & 0 & 0 & 1/2 \\ 0 & 1 & 0 & 0 & 1/2 \\ 0 & 0 & 1 & 0 & 0 \\ 0 & 0 & 0 & 1 & 3/4 \\ 0 & 0 & 0 & 0 & 1 \end{pmatrix} \quad S^{-1} = \begin{pmatrix} 1 & 0 & 0 & 0 & -1/2 \\ 0 & 1 & 0 & 0 & -1/2 \\ 0 & 0 & 1 & 0 & 0 \\ 0 & 0 & 0 & 1 & -3/4 \\ 0 & 0 & 0 & 0 & 1 \end{pmatrix}$$

a1' = a1
a2' = a2

a3' = a3

a1 = a1'
a2 = a2'
a3 = a3'

a1*' = a1*
a2*' = a2*
a3*' = a3*

a1* = a1*'
a2* = a2*'
a3* = a3*'

q1' = q1 = (0,1/2,g)

q1 = q1' = (0,1/2,g)

findssg B'2/m(0 ½ σ3)00

The operators of a mixed setting with B' centering translation have been given as input to findssg.

Input setting

Centering
(0,0,0,0); (1/2,0,1/2,1/2)
Operators
(-x,-y,z,-y+t); (-x,-y,-z,-t); (x,y,z,t); (x,y,-z,y-t)

Standard settings

Superspace group: 12.1.8.5 B2/m(0,1/2,g)00 [Y:1.52]
Bravais class: 1.8 B2/m(0,1/2,g) [JJdW:1.8]
Transformation to supercentered setting: A1=a1, A2=2a2+a4, A3=a3, A4=a4

BASIC SPACE GROUP SETTING
Modulation vectors: q1'=(0,1/2,g)
Centering: (0,0,0,0); (1/2,0,1/2,0)
Non-lattice generators: (-x,-y,z,-y+t); (x,y,-z,y-t)
Non-lattice operators: (x,y,z,t); (-x,-y,z,-y+t); (-x,-y,-z,-t); (x,y,-z,y-t)

SUPERCENTERED SETTING
Modulation vectors: Q1'=(0,0,G), where G=g
Centering: (0,0,0,0); (1/2,0,1/2,0); (0,1/2,0,1/2); (1/2,1/2,1/2,1/2)
Non-lattice generators: (-X,-Y,Z,T); (X,Y,-Z,-T)
Non-lattice operators: (X,Y,Z,T); (-X,-Y,Z,T); (-X,-Y,-Z,-T); (X,Y,-Z,-T)
Reflection conditions: HKLM:H+L=2n; HKLM:K+M=2n

Affine transformation to standard basic space group setting

$S * g(input) * S^{-1} = g(standard)$,
where g is an augmented matrix for an operation in the superspace group.
Also, S * r(input) = r(standard),
where r is an augmented position vector, (x,y,z,t,1).

$$S = \begin{matrix} 1 & 0 & 0 & 0 & 0 \\ 0 & 1 & 0 & 0 & 0 \\ 0 & 0 & 1 & 0 & 0 \\ 0 & 0 & -1 & 1 & 0 \\ 0 & 0 & 0 & 0 & 1 \end{matrix} \quad S^{-1} = \begin{matrix} 1 & 0 & 0 & 0 & 0 \\ 0 & 1 & 0 & 0 & 0 \\ 0 & 0 & 1 & 0 & 0 \\ 0 & 0 & 1 & 1 & 0 \\ 0 & 0 & 0 & 0 & 1 \end{matrix}$$

a1' = a1
a2' = a2

a3' = a3

a1 = a1'
a2 = a2'
a3 = a3'

a1*' = a1*
a2*' = a2*
a3*' = a3*

a1* = a1*'
a2* = a2*'
a3* = a3*'

q1' = q1 - a3* = (0,1/2,g)

q1 = q1' + a3*' = (0,1/2,g+1)

findssg B'2/m(0 ½ σ_3)s0

Input setting

Centering
(0,0,0,0); (1/2,0,1/2,1/2)
Operators
(-x,-y,z,-y+t+1/2); (-x,-y,-z,-t); (x,y,z,t); (x,y,-z,y-t+1/2)

Standard settings

Superspace group: 12.1.8.5 B2/m(0,1/2,g)00 [Y:1.52]
Bravais class: 1.8 B2/m(0,1/2,g) [JJdW:1.8]
Transformation to supercentered setting: A1=a1, A2=2a2+a4, A3=a3, A4=a4

BASIC SPACE GROUP SETTING
Modulation vectors: q1'=(0,1/2,g)
Centering: (0,0,0,0); (1/2,0,1/2,0)
Non-lattice generators: (-x,-y,z,-y+t); (x,y,-z,y-t)
Non-lattice operators: (x,y,z,t); (-x,-y,z,-y+t); (-x,-y,-z,-t); (x,y,-z,y-t)

SUPERCENTERED SETTING
Modulation vectors: Q1'=(0,0,G), where G=g
Centering: (0,0,0,0); (1/2,0,1/2,0); (0,1/2,0,1/2); (1/2,1/2,1/2,1/2)
Non-lattice generators: (-X,-Y,Z,T); (X,Y,-Z,-T)
Non-lattice operators: (X,Y,Z,T); (-X,-Y,Z,T); (-X,-Y,-Z,-T); (X,Y,-Z,-T)
Reflection conditions: HKLM:H+L=2n; HKLM:K+M=2n

Affine transformation to standard basic space group setting

S * g(input) * S $^{-1}$ = g(standard),
where g is an augmented matrix for an operation in the superspace group.
Also, S * r(input) = r(standard),
where r is an augmented position vector, (x,y,z,t,1).

$$S = \begin{pmatrix} 1 & 0 & 0 & 0 & 1/2 \\ 0 & 1 & 0 & 0 & 1/2 \\ 0 & 0 & 1 & 0 & 0 \\ 0 & 0 & -1 & 1 & 0 \\ 0 & 0 & 0 & 0 & 1 \end{pmatrix} \quad S^{-1} = \begin{pmatrix} 1 & 0 & 0 & 0 & -1/2 \\ 0 & 1 & 0 & 0 & -1/2 \\ 0 & 0 & 1 & 0 & 0 \\ 0 & 0 & 1 & 1 & 0 \\ 0 & 0 & 0 & 0 & 1 \end{pmatrix}$$

a1' = a1
a2' = a2
a3' = a3

a1 = a1'

a2 = a2'
a3 = a3'

a1*' = a1*
a2*' = a2*
a3*' = a3*

a1* = a1*'
a2* = a2*'
a3* = a3*'

q1' = q1 - a3* = (0,1/2,g)

q1 = q1' + a3*' = (0,1/2,g+1)

findssg C'2/m(0 σ_2 ½)00

This is the published setting of blue bronze K0.3MoO3.

Input setting

Centering
(0,0,0,0); (1/2,1/2,0,1/2)
Operators
(-x,y,-z,-z+t); (-x,-y,-z,-t); (x,y,z,t); (x,-y,z,z-t)

Standard settings

Superspace group: 12.1.8.5 B2/m(0,1/2,g)00 [Y:1.52]
Bravais class: 1.8 B2/m(0,1/2,g) [JJdW:1.8]
Transformation to supercentered setting: A1=a1, A2=2a2+a4, A3=a3, A4=a4

BASIC SPACE GROUP SETTING
Modulation vectors: q1'=(0,1/2,g)
Centering: (0,0,0,0); (1/2,0,1/2,0)
Non-lattice generators: (-x,-y,z,-y+t); (x,y,-z,y-t)
Non-lattice operators: (x,y,z,t); (-x,-y,z,-y+t); (-x,-y,-z,-t); (x,y,-z,y-t)

SUPERCENTERED SETTING
Modulation vectors: Q1'=(0,0,G), where G=g
Centering: (0,0,0,0); (1/2,0,1/2,0); (0,1/2,0,1/2); (1/2,1/2,1/2,1/2)
Non-lattice generators: (-X,-Y,Z,T); (X,Y,-Z,-T)
Non-lattice operators: (X,Y,Z,T); (-X,-Y,Z,T); (-X,-Y,-Z,-T); (X,Y,-Z,-T)
Reflection conditions: HKLM:H+L=2n; HKLM:K+M=2n

Affine transformation to standard basic space group setting

S * g(input) * S $^{-1}$ = g(standard),
where g is an augmented matrix for an operation in the superspace group.
Also, S * r(input) = r(standard),
where r is an augmented position vector, (x,y,z,t,1).

$$S = \begin{pmatrix} 1 & 0 & 0 & 0 & 0 \\ 0 & 0 & -1 & 0 & 0 \\ 0 & 1 & 0 & 0 & 0 \\ 0 & 1 & 0 & -1 & 0 \\ 0 & 0 & 0 & 0 & 1 \end{pmatrix} \quad S^{-1} = \begin{pmatrix} 1 & 0 & 0 & 0 & 0 \\ 0 & 0 & 1 & 0 & 0 \\ 0 & -1 & 0 & 0 & 0 \\ 0 & 0 & 1 & -1 & 0 \\ 0 & 0 & 0 & 0 & 1 \end{pmatrix}$$

a1' = a1
a2' = -a3
a3' = a2

a1 = a1'
a2 = a3'
a3 = -a2'

a1*' = a1*
a2*' = -a3*
a3*' = a2*

a1* = a1*'
a2* = a3*'
a3* = -a2*'

q1' = -q1 + a2* = (0,1/2,g)

q1 = -q1' + a3*' = (0,-g+1,1/2)

findssg $C_c2/m(0\ \sigma_2\ 0)00$

Input setting

Centering
(0,0,0,0); (1/2,1/2,0,1/2); (0,0,1/2,1/2); (1/2,1/2,1/2,0)
Operators
(-x,y,-z,t); (-x,-y,-z,-t); (x,y,z,t); (x,-y,z,-t)

Standard settings

Superspace group: 12.1.8.5 B2/m(0,1/2,g)00 [Y:1.52]
Bravais class: 1.8 B2/m(0,1/2,g) [JJdW:1.8]
Transformation to supercentered setting: A1=a1, A2=2a2+a4, A3=a3, A4=a4

BASIC SPACE GROUP SETTING
Modulation vectors: q1'=(0,1/2,g)
Centering: (0,0,0,0); (1/2,0,1/2,0)
Non-lattice generators: (-x,-y,z,-y+t); (x,y,-z,y-t)
Non-lattice operators: (x,y,z,t); (-x,-y,z,-y+t); (-x,-y,-z,-t); (x,y,-z,y-t)

SUPERCENTERED SETTING
Modulation vectors: Q1'=(0,0,G), where G=g
Centering: (0,0,0,0); (1/2,0,1/2,0); (0,1/2,0,1/2); (1/2,1/2,1/2,1/2)
Non-lattice generators: (-X,-Y,Z,T); (X,Y,-Z,-T)
Non-lattice operators: (X,Y,Z,T); (-X,-Y,Z,T); (-X,-Y,-Z,-T); (X,Y,-Z,-T)
Reflection conditions: HKLM:H+L=2n; HKLM:K+M=2n

Affine transformation to standard basic space group setting

S * g(input) * S $^{-1}$ = g(standard),
where g is an augmented matrix for an operation in the superspace group.
Also, S * r(input) = r(standard),
where r is an augmented position vector, (x,y,z,t,1).

$$S = \begin{matrix} -1 & 0 & 0 & 0 & 0 \\ 0 & 0 & 2 & 0 & 0 \\ 0 & 1 & 0 & 0 & 0 \\ 0 & 1 & 1 & -1 & 0 \\ 0 & 0 & 0 & 0 & 1 \end{matrix} \qquad S^{-1} = \begin{matrix} -1 & 0 & 0 & 0 & 0 \\ 0 & 0 & 1 & 0 & 0 \\ 0 & 1/2 & 0 & 0 & 0 \\ 0 & 1/2 & 1 & -1 & 0 \\ 0 & 0 & 0 & 0 & 1 \end{matrix}$$

a1' = -a1
a2' = 1/2 a3
a3' = a2

a1 = -a1'

a2 = a3'
a3 = 2 a2'

a1*' = -a1*
a2*' = 2 a3*
a3*' = a2*

a1* = -a1*'
a2* = a3*'
a3* = 1/2 a2*'

q1' = -q1 + a2* + a3* = (0,1/2,g)

q1 = -q1' + 1/2 a2*' + a3*' = (0,-g+1,0)

findssg C'2/m(0 σ_2 ½)s0

Input setting

Centering
(0,0,0,0); (1/2,1/2,0,1/2)
Operators
(-x,y,-z,-z+t+1/2); (-x,-y,-z,-t); (x,y,z,t); (x,-y,z,z-t+1/2)

Standard settings

Superspace group: 12.1.8.5 B2/m(0,1/2,g)00 [Y:1.52]
Bravais class: 1.8 B2/m(0,1/2,g) [JJdW:1.8]
Transformation to supercentered setting: A1=a1, A2=2a2+a4, A3=a3, A4=a4

BASIC SPACE GROUP SETTING
Modulation vectors: q1'=(0,1/2,g)
Centering: (0,0,0,0); (1/2,0,1/2,0)
Non-lattice generators: (-x,-y,z,-y+t); (x,y,-z,y-t)
Non-lattice operators: (x,y,z,t); (-x,-y,z,-y+t); (-x,-y,-z,-t); (x,y,-z,y-t)

SUPERCENTERED SETTING
Modulation vectors: Q1'=(0,0,G), where G=g
Centering: (0,0,0,0); (1/2,0,1/2,0); (0,1/2,0,1/2); (1/2,1/2,1/2,1/2)
Non-lattice generators: (-X,-Y,Z,T); (X,Y,-Z,-T)
Non-lattice operators: (X,Y,Z,T); (-X,-Y,Z,T); (-X,-Y,-Z,-T); (X,Y,-Z,-T)
Reflection conditions: HKLM:H+L=2n; HKLM:K+M=2n

Affine transformation to standard basic space group setting

S * g(input) * S $^{-1}$ = g(standard),
where g is an augmented matrix for an operation in the superspace group.
Also, S * r(input) = r(standard),
where r is an augmented position vector, (x,y,z,t,1).

$$S = \begin{array}{ccccc} 1 & 0 & 0 & 0 & 1/2 \\ 0 & 0 & -1 & 0 & 1/2 \\ 0 & 1 & 0 & 0 & 0 \\ 0 & 1 & 0 & -1 & 0 \\ 0 & 0 & 0 & 0 & 1 \end{array} \qquad S^{-1} = \begin{array}{ccccc} 1 & 0 & 0 & 0 & -1/2 \\ 0 & 0 & 1 & 0 & 0 \\ 0 & -1 & 0 & 0 & 1/2 \\ 0 & 0 & 1 & -1 & 0 \\ 0 & 0 & 0 & 0 & 1 \end{array}$$

a1' = a1
a2' = -a3
a3' = a2

a1 = a1'

a2 = a3'
a3 = -a2'

a1*' = a1*
a2*' = -a3*
a3*' = a2*

a1* = a1*'
a2* = a3*'
a3* = -a2*'

q1' = -q1 + a2* = (0,1/2,g)

q1 = -q1' + a3*' = (0,-g+1,1/2)

findssg $C_c2/m(0\ \sigma_2\ 0)s0$

Input setting

Centering
(0,0,0,0); (1/2,1/2,0,1/2); (0,0,1/2,1/2); (1/2,1/2,1/2,0)
Operators
(-x,y,-z,t+1/2); (-x,-y,-z,-t); (x,y,z,t); (x,-y,z,-t+1/2)

Standard settings

Superspace group: 12.1.8.5 B2/m(0,1/2,g)00 [Y:1.52]
Bravais class: 1.8 B2/m(0,1/2,g) [JJdW:1.8]
Transformation to supercentered setting: A1=a1, A2=2a2+a4, A3=a3, A4=a4

BASIC SPACE GROUP SETTING
Modulation vectors: q1'=(0,1/2,g)
Centering: (0,0,0,0); (1/2,0,1/2,0)
Non-lattice generators: (-x,-y,z,-y+t); (x,y,-z,y-t)
Non-lattice operators: (x,y,z,t); (-x,-y,z,-y+t); (-x,-y,-z,-t); (x,y,-z,y-t)

SUPERCENTERED SETTING
Modulation vectors: Q1'=(0,0,G), where G=g
Centering: (0,0,0,0); (1/2,0,1/2,0); (0,1/2,0,1/2); (1/2,1/2,1/2,1/2)
Non-lattice generators: (-X,-Y,Z,T); (X,Y,-Z,-T)
Non-lattice operators: (X,Y,Z,T); (-X,-Y,Z,T); (-X,-Y,-Z,-T); (X,Y,-Z,-T)
Reflection conditions: HKLM:H+L=2n; HKLM:K+M=2n

Affine transformation to standard basic space group setting

$S * g(\text{input}) * S^{-1} = g(\text{standard})$,
where g is an augmented matrix for an operation in the superspace group.
Also, S * r(input) = r(standard),
where r is an augmented position vector, (x,y,z,t,1).

$$S = \begin{pmatrix} -1 & 0 & 0 & 0 & 1/2 \\ 0 & 0 & 2 & 0 & 1/2 \\ 0 & 1 & 0 & 0 & 0 \\ 0 & 1 & 1 & -1 & 0 \\ 0 & 0 & 0 & 0 & 1 \end{pmatrix} \quad S^{-1} = \begin{pmatrix} -1 & 0 & 0 & 0 & 1/2 \\ 0 & 0 & 1 & 0 & 0 \\ 0 & 1/2 & 0 & 0 & -1/4 \\ 0 & 1/2 & 1 & -1 & -1/4 \\ 0 & 0 & 0 & 0 & 1 \end{pmatrix}$$

a1' = -a1
a2' = 1/2 a3
a3' = a2

a1 = -a1'

a2 = a3'
a3 = 2 a2'

a1*' = -a1*
a2*' = 2 a3*
a3*' = a2*

a1* = -a1*'
a2* = a3*'
a3* = 1/2 a2*'

q1' = -q1 + a2* + a3* = (0,1/2,g)

q1 = -q1' + 1/2 a2*' + a3*' = (0,-g+1,0)
