# Supplementary material for: Equivalence of superspace groups
Source: Acta Crystallogr A. 2012 Nov 14;69(Pt 1):75–90. doi: 10.1107/S0108767312041657 (PMC3553647; doi:10.1107/S0108767312041657)
Supplement: Supplementary file 1 [file a-69-00075-sup1.zip › ssg1d_cp2m_pbtis3_misfit.pdf]

12.1.7.4 B2/m(0,0,g)s0

Superspace group: 12.1.7.4 B2/m(0,0,g)s0 [Y:1.50]
Bravais class: 1.7 B2/m(0,0,g) [JJdW:1.7]
Transformation to supercentered setting: none
Modulation vectors: q1=(0,0,g)
Centering: (0,0,0,0); (1/2,0,1/2,0)
Non-lattice generators: (-x,-y,z,t+1/2); (x,y,-z,-t+1/2)
Non-lattice operators: (x,y,z,t); (-x,-y,z,t+1/2); (-x,-y,-z,-t); (x,y,-z,-t+1/2)
Reflection conditions: hklm:h+l=2n; 00lm:m=2n

This is the superspace group of the composite crystal, misfit layer compound [PbS]1.18[TiS2] See: S. van Smaalen, A. Meetsma, G. A. Wiegers and J. L. De Boer, Acta Crystallogr. B 47, 314-325 (1191). The same symmetry applies to isostructural (Ca0.85OH)1.1564CoO2. See: M. Isobe, M. Onoda, M. Shizuya, M. Tanaka and E. Takayama-Muromachi, JACS 129, 14586 (2007).
The published setting is C'2/m(a,0,0)s-1. with C' = (1/2, 1/2, 0, 1/2).

-----

findssg B2/m(0,0,g)s0

Generators of the standard BSG setting have been given as input to findssg.

Input setting

Centering
(0,0,0,0); (1/2,0,1/2,0)
Operators
(-x,-y,z,t+1/2); (x,y,-z,-t+1/2); (x,y,z,t); (-x,-y,-z,-t)

Standard settings

Superspace group: 12.1.7.4 B2/m(0,0,g)s0 [Y:1.50]
Bravais class: 1.7 B2/m(0,0,g) [JJdW:1.7]
Transformation to supercentered setting: none
Modulation vectors: q1'=(0,0,g)
Centering: (0,0,0,0); (1/2,0,1/2,0)
Non-lattice generators: (-x,-y,z,t+1/2); (x,y,-z,-t+1/2)
Non-lattice operators: (x,y,z,t); (-x,-y,z,t+1/2); (-x,-y,-z,-t); (x,y,-z,-t+1/2)
Reflection conditions: hklm:h+l=2n; 00lm:m=2n

Affine transformation to standard basic space group setting

$S * g(input) * S^{-1} = g(standard)$,
where g is an augmented matrix for an operation in the superspace group.
Also, S * r(input) = r(standard),
where r is an augmented position vector, (x,y,z,t,1).

$$S = \begin{pmatrix} 1 & 0 & 0 & 0 & 0 \\ 0 & 1 & 0 & 0 & 0 \\ 0 & 0 & 1 & 0 & 0 \\ 0 & 0 & 0 & 1 & 0 \\ 0 & 0 & 0 & 0 & 1 \end{pmatrix} \quad S^{-1} = \begin{pmatrix} 1 & 0 & 0 & 0 & 0 \\ 0 & 1 & 0 & 0 & 0 \\ 0 & 0 & 1 & 0 & 0 \\ 0 & 0 & 0 & 1 & 0 \\ 0 & 0 & 0 & 0 & 1 \end{pmatrix}$$

a1' = a1
a2' = a2
a3' = a3

a1 = a1'
a2 = a2'
a3 = a3'

a1*' = a1*
a2*' = a2*
a3*' = a3*

a1* = a1*'
a2* = a2*'
a3* = a3*'

q1' = q1 = (0,0,g)

q1 = q1' = (0,0,g)

findssg C'2/m(a,0,0)s0

Generators of the published setting have been given as input to findssg.

Input setting

Centering
(0,0,0,0); (1/2,1/2,0,1/2)
Operators
(x,-y,-z,t+1/2); (-x,-y,-z,-t); (x,y,z,t); (-x,y,z,-t+1/2)

Standard settings

Superspace group: 12.1.7.4 B2/m(0,0,g)s0 [Y:1.50]
Bravais class: 1.7 B2/m(0,0,g) [JJdW:1.7]
Transformation to supercentered setting: none
Modulation vectors: q1'=(0,0,g)
Centering: (0,0,0,0); (1/2,0,1/2,0)
Non-lattice generators: (-x,-y,z,t+1/2); (x,y,-z,-t+1/2)
Non-lattice operators: (x,y,z,t); (-x,-y,z,t+1/2); (-x,-y,-z,-t); (x,y,-z,-t+1/2)
Reflection conditions: hklm:h+l=2n; 00lm:m=2n

Affine transformation to standard basic space group setting

$S * g(\text{input}) * S^{-1} = g(\text{standard})$,
where g is an augmented matrix for an operation in the superspace group.
Also, $S * r(\text{input}) = r(\text{standard})$,
where r is an augmented position vector, (x,y,z,t,1).

$$S = \begin{pmatrix} 0 & 1 & 0 & 0 & 0 \\ 0 & 0 & 1 & 0 & 0 \\ 1 & 0 & 0 & 0 & 0 \\ 1 & 0 & 0 & 1 & 0 \\ 0 & 0 & 0 & 0 & 1 \end{pmatrix} \quad S^{-1} = \begin{pmatrix} 0 & 0 & 1 & 0 & 0 \\ 1 & 0 & 0 & 0 & 0 \\ 0 & 1 & 0 & 0 & 0 \\ 0 & 0 & -1 & 1 & 0 \\ 0 & 0 & 0 & 0 & 1 \end{pmatrix}$$

a1' = a2
a2' = a3
a3' = a1

a1 = a3'
a2 = a1'
a3 = a2'

a1*' = a2*
a2*' = a3*
a3*' = a1*

a1* = a3*'
a2* = a1*'

a3* = a2*'

q1' = q1 + a1* = (0,0,g)

q1 = q1' - a3*' = (g-1,0,0)
