# Supplementary material for: Equivalence of superspace groups
Source: Acta Crystallogr A. 2012 Nov 14;69(Pt 1):75–90. doi: 10.1107/S0108767312041657 (PMC3553647; doi:10.1107/S0108767312041657)
Supplement: Supplementary file 1 [file a-69-00075-sup1.zip › ssg1d_p31c_srxtis3.pdf]

163.1.23.1 P-31c(1/3,1/3,g)000

Superspace group: 163.1.23.1 P-31c(1/3,1/3,g)000 [Y:1.708]
Bravais class: 1.23 P-31m(1/3,1/3,g) [JJdW:1.23]
Transformation to supercentered setting: A1=2a1+a2+a4, A2=-a1+a2, A3=a3, A4=a4

BASIC SPACE GROUP SETTING
Modulation vectors: q1=(1/3,1/3,g)
Centering: (0,0,0,0)
Non-lattice generators: (y,-x+y,-z,y-t); (x,y,z,t); (y,x,z+1/2,t)
Non-lattice operators: (x,y,z,t); (-y,x-y,z,-y+t); (-x+y,-x,z,-x+t); (x,x-y,-z+1/2,x-t); (-x+y,y,-z+1/2,y-t); (-y,-x,-z+1/2,-t); (-x,-y,-z,-t); (y,-x+y,-z,y-t); (x-y,x,-z,x-t); (-x,-x+y,z+1/2,-x+t); (x-y,-y,z+1/2,-y+t); (y,x,z+1/2,t)

SUPERCENTERED SETTING
Modulation vectors: Q1=(0,0,G), where G=g
Centering: (0,0,0,0); (1/3,2/3,0,2/3); (2/3,1/3,0,1/3)
Non-lattice generators: (Y,-X+Y,-Z,-T); (X,Y,Z,T); (X,X-Y,Z+1/2,T)
Non-lattice operators: (X,Y,Z,T); (-Y,X-Y,Z,T); (-X+Y,-X,Z,T); (X-Y,-Y,-Z+1/2,-T); (Y,X,-Z+1/2,-T); (-X,-X+Y,-Z+1/2,-T); (-X,-Y,-Z,-T); (Y,-X+Y,-Z,-T); (X-Y,X,-Z,-T); (-X+Y,Y,Z+1/2,T); (-Y,-X,Z+1/2,T); (X,X-Y,Z+1/2,T)
Reflection conditions: HKLM:H-K-M=3n; H-HLM:L=2n; H0LM:L=2n; 0KLM:L=2n
-----

This is the superspace group of the [Sr] subsystem of the incommensurate composite crystal [Sr]x[TiS3] with x = 1.132 or similar. See:
M. Onoda, M. Saeki, A. Yamamoto and K. Kato, Acta Crystallogr. B 49, 929-936 (1993).

The supercentered setting is the H' centering.
-----

findssg P-31c(1/3,1/3,g)000

Generators of the standard BSG setting have been given as input to findssg.

Input setting

Centering
none
Operators
(y,-x+y,-z,y-t); (x,y,z,t); (y,x,z+1/2,t); (-x+y,-x,z,-x+t); (x,x-y,-z+1/2,x-t); (-x+y,y,-z+1/2,y-t); (-x,-x+y,z+1/2,-x+t); (x-y,x,-z,x-t); (-x,-y,-z,-t); (x-y,-y,z+1/2,-y+t); (-y,-x,-z+1/2,-t); (-y,x-y,z,-y+t)

Standard settings

Superspace group: 163.1.23.1 P-31c(1/3,1/3,g)000 [Y:1.708]
Bravais class: 1.23 P-31m(1/3,1/3,g) [JJdW:1.23]
Transformation to supercentered setting: A1=2a1+a2+a4, A2=-a1+a2, A3=a3, A4=a4

BASIC SPACE GROUP SETTING
Modulation vectors: q1'=(1/3,1/3,g)
Centering: (0,0,0,0)
Non-lattice generators: (y,-x+y,-z,y-t); (x,y,z,t); (y,x,z+1/2,t)
Non-lattice operators: (x,y,z,t); (-y,x-y,z,-y+t); (-x+y,-x,z,-x+t); (x,x-y,-z+1/2,x-t); (-x+y,y,-z+1/2,y-t); (-y,-x,-z+1/2,-t); (-x,-y,-z,-t); (y,-x+y,-z,y-t); (x-y,x,-z,x-t); (-x,-x+y,z+1/2,-x+t); (x-y,-y,z+1/2,-y+t); (y,x,z+1/2,t)

SUPERCENTERED SETTING
Modulation vectors: Q1'=(0,0,G), where G=g
Centering: (0,0,0,0); (1/3,2/3,0,2/3); (2/3,1/3,0,1/3)
Non-lattice generators: (Y,-X+Y,-Z,-T); (X,Y,Z,T); (X,X-Y,Z+1/2,T)
Non-lattice operators: (X,Y,Z,T); (-Y,X-Y,Z,T); (-X+Y,-X,Z,T); (X-Y,-Y,-Z+1/2,-T); (Y,X,-Z+1/2,-T); (-X,-X+Y,-Z+1/2,-T); (-X,-Y,-Z,-T); (Y,-X+Y,-Z,-T); (X-Y,X,-Z,-T); (-X+Y,Y,Z+1/2,T); (-Y,-X,Z+1/2,T); (X,X-Y,Z+1/2,T)
Reflection conditions: HKLM:H-K-M=3n; H-HLM:L=2n; H0LM:L=2n; 0KLM:L=2n

Affine transformation to standard basic space group setting

$S * g(input) * S^{-1} = g(standard)$,
where g is an augmented matrix for an operation in the superspace group.
Also, S * r(input) = r(standard),
where r is an augmented position vector, (x,y,z,t,1).

$$S = \begin{matrix} 1 & 0 & 0 & 0 & 0 \\ 0 & 1 & 0 & 0 & 0 \\ 0 & 0 & 1 & 0 & 0 \\ 0 & 0 & 0 & 1 & 0 \\ 0 & 0 & 0 & 0 & 1 \end{matrix} \quad S^{-1} = \begin{matrix} 1 & 0 & 0 & 0 & 0 \\ 0 & 1 & 0 & 0 & 0 \\ 0 & 0 & 1 & 0 & 0 \\ 0 & 0 & 0 & 1 & 0 \\ 0 & 0 & 0 & 0 & 1 \end{matrix}$$

a1' = a1
a2' = a2
a3' = a3

a1 = a1'
a2 = a2'
a3 = a3'

a1*' = a1*
a2*' = a2*
a3*' = a3*

a1* = a1*'
a2* = a2*'
a3* = a3*'

q1' = q1 = (1/3,1/3,g)

q1 = q1' = (1/3,1/3,g)

findssg H'-31c(0,0,g)000

Generators of the standard supercentered setting have been given as input to findssg.

Input setting

Centering
(0,0,0,0); (1/3,2/3,0,2/3); (2/3,1/3,0,1/3)
Operators
(y,-x+y,-z,-t); (x,y,z,t); (x,x-y,z+1/2,t); (-x+y,-x,z,t); (x-y,-y,-z+1/2,-t); (y,x,-z+1/2,-t); (-x+y,y,z+1/2,t); (x-y,x,-z,-t); (-x,-y,-z,-t); (-y,-x,z+1/2,t); (-x,-x+y,-z+1/2,-t); (-y,x-y,z,t)

Standard settings

Superspace group: 163.1.23.1 P-31c(1/3,1/3,g)000 [Y:1.708]
Bravais class: 1.23 P-31m(1/3,1/3,g) [JJdW:1.23]
Transformation to supercentered setting: A1=2a1+a2+a4, A2=-a1+a2, A3=a3, A4=a4

BASIC SPACE GROUP SETTING
Modulation vectors: q1'=(1/3,1/3,g)
Centering: (0,0,0,0)
Non-lattice generators: (y,-x+y,-z,y-t); (x,y,z,t); (y,x,z+1/2,t)
Non-lattice operators: (x,y,z,t); (-y,x-y,z,-y+t); (-x+y,-x,z,-x+t); (x,x-y,-z+1/2,x-t); (-x+y,y,-z+1/2,y-t); (-y,-x,-z+1/2,-t); (-x,-y,-z,-t); (y,-x+y,-z,y-t); (x-y,x,-z,x-t); (-x,-x+y,z+1/2,-x+t); (x-y,-y,z+1/2,-y+t); (y,x,z+1/2,t)

SUPERCENTERED SETTING
Modulation vectors: Q1'=(0,0,G), where G=g
Centering: (0,0,0,0); (1/3,2/3,0,2/3); (2/3,1/3,0,1/3)
Non-lattice generators: (Y,-X+Y,-Z,-T); (X,Y,Z,T); (X,X-Y,Z+1/2,T)
Non-lattice operators: (X,Y,Z,T); (-Y,X-Y,Z,T); (-X+Y,-X,Z,T); (X-Y,-Y,-Z+1/2,-T); (Y,X,-Z+1/2,-T); (-X,-X+Y,-Z+1/2,-T); (-X,-Y,-Z,-T); (Y,-X+Y,-Z,-T); (X-Y,X,-Z,-T); (-X+Y,Y,Z+1/2,T); (-Y,-X,Z+1/2,T); (X,X-Y,Z+1/2,T)
Reflection conditions: HKLM:H-K-M=3n; H-HLM:L=2n; H0LM:L=2n; 0KLM:L=2n

Affine transformation to standard basic space group setting

S * g(input) * S $^{-1}$ = g(standard),
where g is an augmented matrix for an operation in the superspace group.
Also, S * r(input) = r(standard),
where r is an augmented position vector, (x,y,z,t,1).

$$S = \begin{pmatrix} 2 & -1 & 0 & 0 & 0 \\ 1 & 1 & 0 & 0 & 0 \\ 0 & 0 & 1 & 0 & 0 \\ 1 & 0 & 0 & 1 & 0 \\ 0 & 0 & 0 & 0 & 1 \end{pmatrix} \quad S^{-1} = \begin{pmatrix} 1/3 & 1/3 & 0 & 0 & 0 \\ -1/3 & 2/3 & 0 & 0 & 0 \\ 0 & 0 & 1 & 0 & 0 \\ -1/3 & -1/3 & 0 & 1 & 0 \\ 0 & 0 & 0 & 0 & 1 \end{pmatrix}$$

$a_1' = 1/3\, a_1 - 1/3\, a_2$
$a_2' = 1/3\, a_1 + 2/3\, a_2$
$a_3' = a_3$

$a_1 = 2\, a_1' + a_2'$
$a_2 = -a_1' + a_2'$
$a_3 = a_3'$

$a_1^{*\prime} = 2\, a_1^* - a_2^*$
$a_2^{*\prime} = a_1^* + a_2^*$
$a_3^{*\prime} = a_3^*$

$a_1^* = 1/3\, a_1^{*\prime} + 1/3\, a_2^{*\prime}$
$a_2^* = -1/3\, a_1^{*\prime} + 2/3\, a_2^{*\prime}$
$a_3^* = a_3^{*\prime}$

$q_1' = q_1 + a_1^* = (1/3,1/3,g)$

$q_1 = q_1' - 1/3\, a_1^{*\prime} - 1/3\, a_2^{*\prime} = (0,0,g)$
