# Supplementary material for: Equivalence of superspace groups
Source: Acta Crystallogr A. 2012 Nov 14;69(Pt 1):75–90. doi: 10.1107/S0108767312041657 (PMC3553647; doi:10.1107/S0108767312041657)
Supplement: Supplementary file 1 [file a-69-00075-sup1.zip › ssg1d_pnma_a2bx4.pdf]

Pnma(00g)000 **$TaSe_{0.36}Te_2$**

findssg

Input setting

Centering
none
Operators
(x+1/2,y,-z+1/2,-t); (x,-y+1/2,z,t); (-x+1/2,y+1/2,z+1/2,t); (x,y,z,t); (x+1/2,-y+1/2,-z+1/2,-t); (-x,y+1/2,-z,-t); (-x+1/2,-y,z+1/2,t); (-x,-y,-z,-t)

Standard settings

Superspace group: 62.1.9.1 Pnma(0,0,g)000 [Y:1.354]
Bravais class: 1.9 Pmmm(0,0,g) [JJdW:1.9]
Transformation to supercentered setting: none
Modulation vectors: q1'=(0,0,g)
Centering: (0,0,0,0)
Non-lattice generators: (-x+1/2,y+1/2,z+1/2,t); (x,-y+1/2,z,t); (x+1/2,y,-z+1/2,-t)
Non-lattice operators: (x,y,z,t); (x+1/2,-y+1/2,-z+1/2,-t); (-x,y+1/2,-z,-t); (-x+1/2,-y,z+1/2,t); (-x,-y,-z,-t); (-x+1/2,y+1/2,z+1/2,t); (x,-y+1/2,z,t); (x+1/2,y,-z+1/2,-t)
Reflection conditions: 0klm:k+l=2n; hk00:h=2n

Affine transformation to standard basic space group setting

$S * g(input) * S^{-1} = g(standard)$,
where g is an augmented matrix for an operation in the superspace group.
Also, S * r(input) = r(standard),
where r is an augmented position vector, (x,y,z,t,1).

$$S = \begin{pmatrix} 1&0&0&0&0\\ 0&1&0&0&0\\ 0&0&1&0&0\\ 0&0&0&1&0\\ 0&0&0&0&1 \end{pmatrix} \quad S^{-1} = \begin{pmatrix} 1&0&0&0&0\\ 0&1&0&0&0\\ 0&0&1&0&0\\ 0&0&0&1&0\\ 0&0&0&0&1 \end{pmatrix}$$

a1' = a1
a2' = a2
a3' = a3

a1 = a1'
a2 = a2'
a3 = a3'

a1*' = a1*
a2*' = a2*
a3*' = a3*

a1* = a1*'
a2* = a2*'
a3* = a3*'

q1' = q1 = (0,0,g)

q1 = q1' = (0,0,g)

Pnma(00g)s00

findssg

Input setting

Centering
none
Operators
(x+1/2,y,-z+1/2,-t+1/2); (x,-y+1/2,z,t); (-x+1/2,y+1/2,z+1/2,t+1/2);
(x,y,z,t); (x+1/2,-y+1/2,-z+1/2,-t+1/2); (-x,y+1/2,-z,-t); (-x+1/2,-y,z+1/2,t+1/2); (-x,-y,-z,-t)

Standard settings

Superspace group: 62.1.9.1 Pnma(0,0,g)000 [Y:1.354]
Bravais class: 1.9 Pmmm(0,0,g) [JJdW:1.9]
Transformation to supercentered setting: none
Modulation vectors: q1'=(0,0,g)
Centering: (0,0,0,0)
Non-lattice generators: (-x+1/2,y+1/2,z+1/2,t); (x,-y+1/2,z,t); (x+1/2,y,-z+1/2,-t)
Non-lattice operators: (x,y,z,t); (x+1/2,-y+1/2,-z+1/2,-t); (-x,y+1/2,-z,-t); (-x+1/2,-y,z+1/2,t); (-x,-y,-z,-t); (-x+1/2,y+1/2,z+1/2,t); (x,-y+1/2,z,t); (x+1/2,y,-z+1/2,-t)
Reflection conditions: 0klm:k+l=2n; hk00:h=2n

Affine transformation to standard basic space group setting

$S * g(input) * S^{-1} = g(standard)$,
where g is an augmented matrix for an operation in the superspace group.
Also, S * r(input) = r(standard),
where r is an augmented position vector, (x,y,z,t,1).

$$S = \begin{pmatrix} 1 & 0 & 0 & 0 & 0 \\ 0 & 1 & 0 & 0 & 0 \\ 0 & 0 & 1 & 0 & 0 \\ 0 & 0 & 1 & 1 & 0 \\ 0 & 0 & 0 & 0 & 1 \end{pmatrix} \quad S^{-1} = \begin{pmatrix} 1 & 0 & 0 & 0 & 0 \\ 0 & 1 & 0 & 0 & 0 \\ 0 & 0 & 1 & 0 & 0 \\ 0 & 0 & -1 & 1 & 0 \\ 0 & 0 & 0 & 0 & 1 \end{pmatrix}$$

a1' = a1
a2' = a2
a3' = a3

a1 = a1'
a2 = a2'
a3 = a3'

a1*' = a1*
a2*' = a2*
a3*' = a3*

a1* = a1*'
a2* = a2*'
a3* = a3*'

q1' = q1 + a3* = (0,0,g)

q1 = q1' - a3*' = (0,0,g-1)

Pnma(0b0)000 **Thiourea**

findssg

Input setting

Centering
none
Operators
(x+1/2,y,-z+1/2,t); (x,-y+1/2,z,-t); (-x+1/2,y+1/2,z+1/2,t); (x,y,z,t); (x+1/2,-y+1/2,-z+1/2,-t); (-x,y+1/2,-z,t); (-x+1/2,-y,z+1/2,-t); (-x,-y,-z,-t)

Standard settings

Superspace group: 62.1.9.3 Pbnm(0,0,g)000 [Y:1.350]
Bravais class: 1.9 Pmmm(0,0,g) [JJdW:1.9]
Transformation to supercentered setting: none
Modulation vectors: q1'=(0,0,g)
Centering: (0,0,0,0)
Non-lattice generators: (-x+1/2,y+1/2,z,t); (x+1/2,-y+1/2,z+1/2,t); (x,y,-z+1/2,-t)
Non-lattice operators: (x,y,z,t); (-x+1/2,y+1/2,-z+1/2,-t); (-x,-y,z+1/2,t); (x+1/2,-y+1/2,-z,-t); (-x,-y,-z,-t); (x+1/2,-y+1/2,z+1/2,t); (x,y,-z+1/2,-t); (-x+1/2,y+1/2,z,t)
Reflection conditions: h0lm:h+l=2n; 0klm:k=2n

Affine transformation to standard basic space group setting

$S * g(input) * S^{-1} = g(standard)$,
where g is an augmented matrix for an operation in the superspace group.
Also, S * r(input) = r(standard),
where r is an augmented position vector, (x,y,z,t,1).

$$S = \begin{pmatrix} 0&0&1&0&0\\ 1&0&0&0&0\\ 0&1&0&0&0\\ 0&0&0&1&0\\ 0&0&0&0&1 \end{pmatrix} \quad S^{-1} = \begin{pmatrix} 0&1&0&0&0\\ 0&0&1&0&0\\ 1&0&0&0&0\\ 0&0&0&1&0\\ 0&0&0&0&1 \end{pmatrix}$$

a1' = a3
a2' = a1
a3' = a2

a1 = a2'
a2 = a3'
a3 = a1'

a1*' = a3*
a2*' = a1*
a3*' = a2*

a1* = a2*'
a2* = a3*'
a3* = a1*'

q1' = q1 = (0,0,g)

q1 = q1' = (0,g,0)

Pnma(0b0)s00

findssg

Input setting

Centering
none
Operators
(x+1/2,y,-z+1/2,t); (x,-y+1/2,z,-t+1/2); (-x+1/2,y+1/2,z+1/2,t+1/2); (x,y,z,t); (x+1/2,-y+1/2,-z+1/2,-t+1/2); (-x,y+1/2,-z,t+1/2); (-x+1/2,-y,z+1/2,-t); (-x,-y,-z,-t)

Standard settings

Superspace group: 62.1.9.3 Pbnm(0,0,g)000 [Y:1.350]
Bravais class: 1.9 Pmmm(0,0,g) [JJdW:1.9]
Transformation to supercentered setting: none
Modulation vectors: q1'=(0,0,g)
Centering: (0,0,0,0)
Non-lattice generators: (-x+1/2,y+1/2,z,t); (x+1/2,-y+1/2,z+1/2,t); (x,y,-z+1/2,-t)
Non-lattice operators: (x,y,z,t); (-x+1/2,y+1/2,-z+1/2,-t); (-x,-y,z+1/2,t); (x+1/2,-y+1/2,-z,-t); (-x,-y,-z,-t); (x+1/2,-y+1/2,z+1/2,t); (x,y,-z+1/2,-t); (-x+1/2,y+1/2,z,t)
Reflection conditions: h0lm:h+l=2n; 0klm:k=2n

Affine transformation to standard basic space group setting

S * g(input) * S $^{-1}$ = g(standard),
where g is an augmented matrix for an operation in the superspace group.
Also, S * r(input) = r(standard),
where r is an augmented position vector, (x,y,z,t,1).

$$S = \begin{matrix} 0 & 0 & 1 & 0 & 0 \\ 1 & 0 & 0 & 0 & 0 \\ 0 & 1 & 0 & 0 & 0 \\ 0 & 1 & 0 & 1 & 0 \\ 0 & 0 & 0 & 0 & 1 \end{matrix} \quad S^{-1} = \begin{matrix} 0 & 1 & 0 & 0 & 0 \\ 0 & 0 & 1 & 0 & 0 \\ 1 & 0 & 0 & 0 & 0 \\ 0 & 0 & -1 & 1 & 0 \\ 0 & 0 & 0 & 0 & 1 \end{matrix}$$

a1' = a3
a2' = a1
a3' = a2

a1 = a2'
a2 = a3'
a3 = a1'

a1*' = a3*
a2*' = a1*
a3*' = a2*

a1* = a2*'
a2* = a3*'

a3* = a1*'

q1' = q1 + a2* = (0,0,g)

q1 = q1' - a3*' = (0,g-1,0)

Pnma(a00)000

findssg

Input setting

Centering
none
Operators
(x+1/2,y,-z+1/2,t); (x,-y+1/2,z,t); (-x+1/2,y+1/2,z+1/2,-t); (x,y,z,t); (x+1/2,-y+1/2,-z+1/2,t); (-x,y+1/2,-z,-t); (-x+1/2,-y,z+1/2,-t); (-x,-y,-z,-t)

Standard settings

Superspace group: 62.1.9.5 Pmcn(0,0,g)000 [Y:1.356]
Bravais class: 1.9 Pmmm(0,0,g) [JJdW:1.9]
Transformation to supercentered setting: none
Modulation vectors: q1'=(0,0,g)
Centering: (0,0,0,0)
Non-lattice generators: (-x+1/2,y,z,t); (x,-y+1/2,z+1/2,t); (x+1/2,y+1/2,-z+1/2,-t)
Non-lattice operators: (x,y,z,t); (-x+1/2,-y+1/2,z+1/2,t); (x+1/2,-y,-z,-t); (-x,y+1/2,-z+1/2,-t); (-x,-y,-z,-t); (x+1/2,y+1/2,-z+1/2,-t); (-x+1/2,y,z,t); (x,-y+1/2,z+1/2,t)
Reflection conditions: h0lm:l=2n; hk00:h+k=2n

Affine transformation to standard basic space group setting

$S * g(input) * S^{-1} = g(standard)$,
where g is an augmented matrix for an operation in the superspace group.
Also, S * r(input) = r(standard),
where r is an augmented position vector, (x,y,z,t,1).

$$S = \begin{matrix} 0 & 1 & 0 & 0 & 0 \\ 0 & 0 & 1 & 0 & 0 \\ 1 & 0 & 0 & 0 & 0 \\ 0 & 0 & 0 & 1 & 0 \\ 0 & 0 & 0 & 0 & 1 \end{matrix} \qquad S^{-1} = \begin{matrix} 0 & 0 & 1 & 0 & 0 \\ 1 & 0 & 0 & 0 & 0 \\ 0 & 1 & 0 & 0 & 0 \\ 0 & 0 & 0 & 1 & 0 \\ 0 & 0 & 0 & 0 & 1 \end{matrix}$$

a1' = a2
a2' = a3
a3' = a1

a1 = a3'
a2 = a1'
a3 = a2'

a1*' = a2*
a2*' = a3*
a3*' = a1*

a1* = a3*'
a2* = a1*'
a3* = a2*'

q1' = q1 = (0,0,g)

q1 = q1' = (g,0,0)

Pnma(a00)00s

findssg

Input setting

Centering
none
Operators
(x+1/2,y,-z+1/2,t+1/2); (x,-y+1/2,z,t); (-x+1/2,y+1/2,z+1/2,-t+1/2); (x,y,z,t); (x+1/2,-y+1/2,-z+1/2,t+1/2); (-x,y+1/2,-z,-t); (-x+1/2,-y,z+1/2,-t+1/2); (-x,-y,-z,-t)

Standard settings

Superspace group: 62.1.9.5 Pmcn(0,0,g)000 [Y:1.356]
Bravais class: 1.9 Pmmm(0,0,g) [JJdW:1.9]
Transformation to supercentered setting: none
Modulation vectors: q1'=(0,0,g)
Centering: (0,0,0,0)
Non-lattice generators: (-x+1/2,y,z,t); (x,-y+1/2,z+1/2,t); (x+1/2,y+1/2,-z+1/2,-t)
Non-lattice operators: (x,y,z,t); (-x+1/2,-y+1/2,z+1/2,t); (x+1/2,-y,-z,-t); (-x,y+1/2,-z+1/2,-t); (-x,-y,-z,-t); (x+1/2,y+1/2,-z+1/2,-t); (-x+1/2,y,z,t); (x,-y+1/2,z+1/2,t)
Reflection conditions: h0lm:l=2n; hk00:h+k=2n

Affine transformation to standard basic space group setting

$S * g(input) * S^{-1} = g(standard)$,
where g is an augmented matrix for an operation in the superspace group.
Also, S * r(input) = r(standard),
where r is an augmented position vector, (x,y,z,t,1).

$$S = \begin{pmatrix} 0 & 1 & 0 & 0 & 0 \\ 0 & 0 & 1 & 0 & 0 \\ 1 & 0 & 0 & 0 & 0 \\ 1 & 0 & 0 & 1 & 0 \\ 0 & 0 & 0 & 0 & 1 \end{pmatrix} \quad S^{-1} = \begin{pmatrix} 0 & 0 & 1 & 0 & 0 \\ 1 & 0 & 0 & 0 & 0 \\ 0 & 1 & 0 & 0 & 0 \\ 0 & 0 & -1 & 1 & 0 \\ 0 & 0 & 0 & 0 & 1 \end{pmatrix}$$

a1' = a2
a2' = a3
a3' = a1

a1 = a3'
a2 = a1'
a3 = a2'

a1*' = a2*
a2*' = a3*
a3*' = a1*

a1* = a3*'
a2* = a1*'
a3* = a2*'

q1' = q1 + a1* = (0,0,g)

q1 = q1' - a3*' = (g-1,0,0)

Pnma(a00)0s0

findssg

Input setting

Centering
none
Operators
(-x+1/2,y+1/2,z+1/2,-t+1/2); (x,-y+1/2,z,t+1/2); (x+1/2,y,-z+1/2,t); (x,y,z,t); (-x+1/2,-y,z+1/2,-t); (-x,y+1/2,-z,-t+1/2); (x+1/2,-y+1/2,-z+1/2,t+1/2); (-x,-y,-z,-t)

Standard settings

Superspace group: 62.1.9.6 Pmcn(0,0,g)s00 [Y:1.357]
Bravais class: 1.9 Pmmm(0,0,g) [JJdW:1.9]
Transformation to supercentered setting: none
Modulation vectors: q1'=(0,0,g)
Centering: (0,0,0,0)
Non-lattice generators: (-x+1/2,y,z,t+1/2); (x,-y+1/2,z+1/2,t); (x+1/2,y+1/2,-z+1/2,-t+1/2)
Non-lattice operators: (x,y,z,t); (-x+1/2,-y+1/2,z+1/2,t+1/2); (x+1/2,-y,-z,-t+1/2); (-x,y+1/2,-z+1/2,-t); (-x,-y,-z,-t); (x+1/2,y+1/2,-z+1/2,-t+1/2); (-x+1/2,y,z,t+1/2); (x,-y+1/2,z+1/2,t)
Reflection conditions: h0lm:l=2n; 0klm:m=2n; hk00:h+k=2n

Affine transformation to standard basic space group setting

$S * g(\text{input}) * S^{-1} = g(\text{standard})$,
where g is an augmented matrix for an operation in the superspace group.
Also, S * r(input) = r(standard),
where r is an augmented position vector, (x,y,z,t,1).

$$S = \begin{matrix} 0 & 1 & 0 & 0 & 0 \\ 0 & 0 & 1 & 0 & 0 \\ 1 & 0 & 0 & 0 & 0 \\ 0 & 0 & 0 & 1 & 0 \\ 0 & 0 & 0 & 0 & 1 \end{matrix} \qquad S^{-1} = \begin{matrix} 0 & 0 & 1 & 0 & 0 \\ 1 & 0 & 0 & 0 & 0 \\ 0 & 1 & 0 & 0 & 0 \\ 0 & 0 & 0 & 1 & 0 \\ 0 & 0 & 0 & 0 & 1 \end{matrix}$$

a1' = a2
a2' = a3
a3' = a1

a1 = a3'
a2 = a1'
a3 = a2'

a1*' = a2*
a2*' = a3*
a3*' = a1*

a1* = a3*'
a2* = a1*'
a3* = a2*'

q1' = q1 = (0,0,g)

q1 = q1' = (g,0,0)

Pnam(a00)00s

findssg

Input setting

Centering
none
Operators
(-x+1/2,y+1/2,z+1/2,-t+1/2); (x+1/2,-y+1/2,z,t); (x,y,-z+1/2,t+1/2); (x,y,z,t); (-x,-y,z+1/2,-t+1/2); (-x+1/2,y+1/2,-z,-t); (x+1/2,-y+1/2,-z+1/2,t+1/2); (-x,-y,-z,-t)

Standard settings

Superspace group: 62.1.9.6 Pmcn(0,0,g)s00 [Y:1.357]
Bravais class: 1.9 Pmmm(0,0,g) [JJdW:1.9]
Transformation to supercentered setting: none
Modulation vectors: q1'=(0,0,g)
Centering: (0,0,0,0)
Non-lattice generators: (-x+1/2,y,z,t+1/2); (x,-y+1/2,z+1/2,t); (x+1/2,y+1/2,-z+1/2,-t+1/2)
Non-lattice operators: (x,y,z,t); (-x+1/2,-y+1/2,z+1/2,t+1/2); (x+1/2,-y,-z,-t+1/2); (-x,y+1/2,-z+1/2,-t); (-x,-y,-z,-t); (x+1/2,y+1/2,-z+1/2,-t+1/2); (-x+1/2,y,z,t+1/2); (x,-y+1/2,z+1/2,t)
Reflection conditions: h0lm:l=2n; 0klm:m=2n; hk00:h+k=2n

Affine transformation to standard basic space group setting

$S * g(\text{input}) * S^{-1} = g(\text{standard})$,
where g is an augmented matrix for an operation in the superspace group.
Also, S * r(input) = r(standard),
where r is an augmented position vector, (x,y,z,t,1).

$$S = \begin{matrix} 0 & 0 & 1 & 0 & 0 \\ 0 & 1 & 0 & 0 & 0 \\ -1 & 0 & 0 & 0 & 0 \\ 0 & 0 & 0 & 1 & 0 \\ 0 & 0 & 0 & 0 & 1 \end{matrix} \qquad S^{-1} = \begin{matrix} 0 & 0 & -1 & 0 & 0 \\ 0 & 1 & 0 & 0 & 0 \\ 1 & 0 & 0 & 0 & 0 \\ 0 & 0 & 0 & 1 & 0 \\ 0 & 0 & 0 & 0 & 1 \end{matrix}$$

a1' = a3
a2' = a2
a3' = -a1

a1 = -a3'
a2 = a2'
a3 = a1'

a1*' = a3*
a2*' = a2*
a3*' = -a1*

a1* = -a3*'
a2* = a2*'
a3* = a1*'

q1' = q1 = (0,0,g)

q1 = q1' = (-g,0,0)

Pnam(a00)0ss **K_2SeO_4**

findssg

Input setting

Centering
none
Operators
(-x+1/2,y+1/2,z+1/2,-t); (x+1/2,-y+1/2,z,t+1/2); (x,y,-z+1/2,t+1/2); (x,y,z,t); (-x,-y,z+1/2,-t+1/2); (-x+1/2,y+1/2,-z,-t+1/2); (x+1/2,-y+1/2,-z+1/2,t); (-x,-y,-z,-t)

Standard settings

Superspace group: 62.1.9.6 Pmcn(0,0,g)s00 [Y:1.357]
Bravais class: 1.9 Pmmm(0,0,g) [JJdW:1.9]
Transformation to supercentered setting: none
Modulation vectors: q1'=(0,0,g)
Centering: (0,0,0,0)
Non-lattice generators: (-x+1/2,y,z,t+1/2); (x,-y+1/2,z+1/2,t); (x+1/2,y+1/2,-z+1/2,-t+1/2)
Non-lattice operators: (x,y,z,t); (-x+1/2,-y+1/2,z+1/2,t+1/2); (x+1/2,-y,-z,-t+1/2); (-x,y+1/2,-z+1/2,-t); (-x,-y,-z,-t); (x+1/2,y+1/2,-z+1/2,-t+1/2); (-x+1/2,y,z,t+1/2); (x,-y+1/2,z+1/2,t)
Reflection conditions: h0lm:l=2n; 0klm:m=2n; hk00:h+k=2n

Affine transformation to standard basic space group setting

S * g(input) * S $^{-1}$ = g(standard),
where g is an augmented matrix for an operation in the superspace group.
Also, S * r(input) = r(standard),
where r is an augmented position vector, (x,y,z,t,1).

$$S = \begin{matrix} 0 & 0 & 1 & 0 & 0 \\ 0 & 1 & 0 & 0 & 0 \\ -1 & 0 & 0 & 0 & 0 \\ 1 & 0 & 0 & 1 & 0 \\ 0 & 0 & 0 & 0 & 1 \end{matrix} \qquad S^{-1} = \begin{matrix} 0 & 0 & -1 & 0 & 0 \\ 0 & 1 & 0 & 0 & 0 \\ 1 & 0 & 0 & 0 & 0 \\ 0 & 0 & 1 & 1 & 0 \\ 0 & 0 & 0 & 0 & 1 \end{matrix}$$

a1' = a3
a2' = a2
a3' = -a1

a1 = -a3'
a2 = a2'
a3 = a1'

a1*' = a3*
a2*' = a2*
a3*' = -a1*

a1* = -a3*'
a2* = a2*'

a3* = a1*'

q1' = q1 + a1* = (0,0,g)

q1 = q1' + a3*' = (-g-1,0,0)

Pmcn(00g)ss0 **Rb_2ZnCl_4**

findssg

Input setting

Centering
none
Operators
(-x+1/2,y,z,t+1/2); (x,-y+1/2,z+1/2,t+1/2); (x+1/2,y+1/2,-z+1/2,-t); (x,y,z,t); (-x+1/2,-y+1/2,z+1/2,t); (-x,y+1/2,-z+1/2,-t+1/2); (x+1/2,-y,-z,-t+1/2); (-x,-y,-z,-t)

Standard settings

Superspace group: 62.1.9.6 Pmcn(0,0,g)s00 [Y:1.357]
Bravais class: 1.9 Pmmm(0,0,g) [JJdW:1.9]
Transformation to supercentered setting: none
Modulation vectors: q1'=(0,0,g)
Centering: (0,0,0,0)
Non-lattice generators: (-x+1/2,y,z,t+1/2); (x,-y+1/2,z+1/2,t); (x+1/2,y+1/2,-z+1/2,-t+1/2)
Non-lattice operators: (x,y,z,t); (-x+1/2,-y+1/2,z+1/2,t+1/2); (x+1/2,-y,-z,-t+1/2); (-x,y+1/2,-z+1/2,-t); (-x,-y,-z,-t); (x+1/2,y+1/2,-z+1/2,-t+1/2); (-x+1/2,y,z,t+1/2); (x,-y+1/2,z+1/2,t)
Reflection conditions: h0lm:l=2n; 0klm:m=2n; hk00:h+k=2n

Affine transformation to standard basic space group setting

$S * g(input) * S^{-1} = g(standard)$,
where g is an augmented matrix for an operation in the superspace group.
Also, S * r(input) = r(standard),
where r is an augmented position vector, (x,y,z,t,1).

$$S = \begin{matrix} 1 & 0 & 0 & 0 & 0 \\ 0 & 1 & 0 & 0 & 0 \\ 0 & 0 & 1 & 0 & 0 \\ 0 & 0 & 1 & 1 & 0 \\ 0 & 0 & 0 & 0 & 1 \end{matrix} \qquad S^{-1} = \begin{matrix} 1 & 0 & 0 & 0 & 0 \\ 0 & 1 & 0 & 0 & 0 \\ 0 & 0 & 1 & 0 & 0 \\ 0 & 0 & -1 & 1 & 0 \\ 0 & 0 & 0 & 0 & 1 \end{matrix}$$

a1' = a1
a2' = a2
a3' = a3

a1 = a1'
a2 = a2'
a3 = a3'

a1*' = a1*
a2*' = a2*
a3*' = a3*

a1* = a1*'
a2* = a2*'
a3* = a3*'

q1' = q1 + a3* = (0,0,g)

q1 = q1' - a3*' = (0,0,g-1)
