# Supplementary material for: Equivalence of superspace groups
Source: Acta Crystallogr A. 2012 Nov 14;69(Pt 1):75–90. doi: 10.1107/S0108767312041657 (PMC3553647; doi:10.1107/S0108767312041657)
Supplement: Supplementary file 1 [file a-69-00075-sup1.zip › ssg1d_r3m_srxtis3.pdf]

166.1.22.2 R-3m(0,0,g)0s

Superspace group: 166.1.22.2 R-3m(0,0,g)0s [Y:1.701]
Bravais class: 1.22 R-3m(0,0,g) [JJdW:1.22]
Transformation to supercentered setting: none
Modulation vectors: q1=(0,0,g)
Centering: (0,0,0,0); (2/3,1/3,1/3,0); (1/3,2/3,2/3,0)
Non-lattice generators: (y,-x+y,-z,-t); (x,x-y,z,t+1/2)
Non-lattice operators: (x,y,z,t); (-y,x-y,z,t); (-x+y,-x,z,t); (y,x,-z,-t+1/2); (-x,-x+y,-z,-t+1/2); (x-y,-y,-z,-t+1/2); (-x,-y,-z,-t); (y,-x+y,-z,-t); (x-y,x,-z,-t); (-y,-x,z,t+1/2); (x,x-y,z,t+1/2); (-x+y,y,z,t+1/2)
Reflection conditions: hklm:h-k-l=3n; h-hlm:m=2n; h0lm:m=2n; 0klm:m=2n
-----

This is the superspace group of the incommensurate composite crystal [Sr]x[TiS3] with x = 1.132 or similar. See:
M. Onoda, M. Saeki, A. Yamamoto and K. Kato, Acta Crystallogr. B 49, 929-936 (1993).

There is no supercentered setting.
-----

findssg R-3m(0,0,g)0s

Generators of the standard BSG setting have been given as input to findssg.

Input setting

Centering
(0,0,0,0); (2/3,1/3,1/3,0); (1/3,2/3,2/3,0)
Operators
(y,-x+y,-z,-t); (x,x-y,z,t+1/2); (-x+y,-x,z,t); (x-y,-y,-z,-t+1/2); (y,x,-z,-t+1/2); (x,y,z,t); (-x+y,y,z,t+1/2); (x-y,x,-z,-t); (-x,-y,-z,-t); (-y,-x,z,t+1/2); (-x,-x+y,-z,-t+1/2); (-y,x-y,z,t)

Standard settings

Superspace group: 166.1.22.2 R-3m(0,0,g)0s [Y:1.701]
Bravais class: 1.22 R-3m(0,0,g) [JJdW:1.22]
Transformation to supercentered setting: none
Modulation vectors: q1'=(0,0,g)
Centering: (0,0,0,0); (2/3,1/3,1/3,0); (1/3,2/3,2/3,0)
Non-lattice generators: (y,-x+y,-z,-t); (x,x-y,z,t+1/2)
Non-lattice operators: (x,y,z,t); (-y,x-y,z,t); (-x+y,-x,z,t); (y,x,-z,-t+1/2); (-x,-x+y,-z,-t+1/2); (x-y,-y,-z,-t+1/2); (-x,-y,-z,-t); (y,-x+y,-z,-t); (x-y,x,-z,-t); (-y,-x,z,t+1/2); (x,x-y,z,t+1/2); (-x+y,y,z,t+1/2)
Reflection conditions: hklm:h-k-l=3n; h-hlm:m=2n; h0lm:m=2n; 0klm:m=2n

Affine transformation to standard basic space group setting

$S * g(input) * S^{-1} = g(standard)$,
where g is an augmented matrix for an operation in the superspace group.
Also, S * r(input) = r(standard),
where r is an augmented position vector, (x,y,z,t,1).

$$S = \begin{pmatrix} 1&0&0&0&0 \\ 0&1&0&0&0 \\ 0&0&1&0&0 \\ 0&0&0&1&0 \\ 0&0&0&0&1 \end{pmatrix} \quad S^{-1} = \begin{pmatrix} 1&0&0&0&0 \\ 0&1&0&0&0 \\ 0&0&1&0&0 \\ 0&0&0&1&0 \\ 0&0&0&0&1 \end{pmatrix}$$

a1' = a1
a2' = a2
a3' = a3

a1 = a1'
a2 = a2'
a3 = a3'

a1*' = a1*
a2*' = a2*
a3*' = a3*

a1* = a1*'
a2* = a2*'
a3* = a3*'

q1' = q1 = (0,0,g)

q1 = q1' = (0,0,g)
