# Supplementary material for: Equivalence of superspace groups
Source: Acta Crystallogr A. 2012 Nov 14;69(Pt 1):75–90. doi: 10.1107/S0108767312041657 (PMC3553647; doi:10.1107/S0108767312041657)
Supplement: Supplementary file 1 [file a-69-00075-sup1.zip › ssg2d_aa_bisrcacuo.pdf]

9.2.4.1 Bb(a1,b1,0)0(a2,b2,0)0

Superspace group: 9.2.4.1 Bb(a1,b1,0)0(a2,b2,0)0 [Y:2.57]
Bravais class: 2.4 B2/m(a1,b1,0)(a2,b2,0)   [JJdW:2.4]
Transformation to supercentered setting: none
Modulation vectors: q1=(a1,b1,0), q2=(a2,b2,0)
Centering: (0,0,0,0,0); (1/2,0,1/2,0,0)
Non-lattice generators: (x,y+1/2,-z,t,u)
Non-lattice operators: (x,y,z,t,u); (x,y+1/2,-z,t,u)
Reflection conditions: hklmn:h+l=2n; hk0mn:k=2n

There is no supercentered setting, i.e. this is a primitive superspace lattice.
This is the symmetry of 221 BiSrCaCuO : Y. Gao, P. Lee, H. Graafsma, J. Yeh, P. Bush, V. Petricek and P. Coppens, Chem. Mater. 2, 323-328 (1990).
"Incommensurate modulations in the Pb-doped BiSrCaCuO 221 superconducting phase; a five-dimensional superspace description."

-----

findssg Bb(a1,b1,0)0(a2,b2,0)0

Generators of standard BSG setting entered into findssg.

Input setting

Centering
(0,0,0,0,0); (1/2,0,1/2,0,0)
Operators
(x,y+1/2,-z,t,u); (x,y,z,t,u)

Standard settings

Superspace group: 9.2.4.1 Bb(a1,b1,0)0(a2,b2,0)0 [Y:2.57]
Bravais class: 2.4 B2/m(a1,b1,0)(a2,b2,0) [JJdW:2.4]
Transformation to supercentered setting: none
Modulation vectors: q1'=(a1,b1,0), q2'=(a2,b2,0)
Centering: (0,0,0,0,0); (1/2,0,1/2,0,0)
Non-lattice generators: (x,y+1/2,-z,t,u)
Non-lattice operators: (x,y,z,t,u); (x,y+1/2,-z,t,u)
Reflection conditions: hklmn:h+l=2n; hk0mn:k=2n

Affine transformation to standard basic space group setting

S * g(input) * S $^{-1}$ = g(standard),
where g is an augmented matrix for an operation in the superspace group.
Also, S * r(input) = r(standard),
where r is an augmented position vector, (x,y,z,t,u,1).

$$S = \begin{pmatrix} 1 & 0 & 0 & 0 & 0 & 0 \\ 0 & 1 & 0 & 0 & 0 & 0 \\ 0 & 0 & 1 & 0 & 0 & 0 \\ 0 & 0 & 0 & 1 & 0 & 0 \\ 0 & 0 & 0 & 0 & 1 & 0 \\ 0 & 0 & 0 & 0 & 0 & 1 \end{pmatrix} \quad S^{-1} = \begin{pmatrix} 1 & 0 & 0 & 0 & 0 & 0 \\ 0 & 1 & 0 & 0 & 0 & 0 \\ 0 & 0 & 1 & 0 & 0 & 0 \\ 0 & 0 & 0 & 1 & 0 & 0 \\ 0 & 0 & 0 & 0 & 1 & 0 \\ 0 & 0 & 0 & 0 & 0 & 1 \end{pmatrix}$$

a1' = a1
a2' = a2
a3' = a3

a1 = a1'
a2 = a2'
a3 = a3'

a1*' = a1*
a2*' = a2*
a3*' = a3*

a1* = a1*'
a2* = a2*'
a3* = a3*'

q1' = q1 = (a1,b1,0)
q2' = q2 = (a2,b2,0)

q1 = q1' = (a1,b1,0)
q2 = q2' = (a2,b2,0)

findssg

Generators of published supercentered setting entered into findssg.

Input setting

Centering
(0,0,0,0,0); (0,1/2,1/2,1/2,0)
Operators
(x+1/2,-y,z,t,u); (x,y,z,t,u)

Standard settings

Superspace group: 9.2.4.1 Bb(a1,b1,0)0(a2,b2,0)0 [Y:2.57]
Bravais class: 2.4 B2/m(a1,b1,0)(a2,b2,0) [JJdW:2.4]
Transformation to supercentered setting: none
Modulation vectors: q1'=(a1,b1,0), q2'=(a2,b2,0)
Centering: (0,0,0,0,0); (1/2,0,1/2,0,0)
Non-lattice generators: (x,y+1/2,-z,t,u)
Non-lattice operators: (x,y,z,t,u); (x,y+1/2,-z,t,u)
Reflection conditions: hklmn:h+l=2n; hk0mn:k=2n

Affine transformation to standard basic space group setting

$S * g(input) * S^{-1} = g(standard)$,
where g is an augmented matrix for an operation in the superspace group.
Also, S * r(input) = r(standard),
where r is an augmented position vector, (x,y,z,t,u,1).

$$S = \begin{pmatrix} 0 & 0 & 1 & 0 & 0 & 0 \\ 1 & 0 & 0 & 0 & 0 & 0 \\ 0 & 1 & 0 & 0 & 0 & 0 \\ 0 & 0 & -1 & 1 & 0 & 0 \\ 0 & 0 & 0 & 0 & 1 & 0 \\ 0 & 0 & 0 & 0 & 0 & 1 \end{pmatrix} \quad S^{-1} = \begin{pmatrix} 0 & 1 & 0 & 0 & 0 & 0 \\ 0 & 0 & 1 & 0 & 0 & 0 \\ 1 & 0 & 0 & 0 & 0 & 0 \\ 1 & 0 & 0 & 1 & 0 & 0 \\ 0 & 0 & 0 & 0 & 1 & 0 \\ 0 & 0 & 0 & 0 & 0 & 1 \end{pmatrix}$$

a1' = a3
a2' = a1
a3' = a2

a1 = a2'
a2 = a3'
a3 = a1'

a1*' = a3*
a2*' = a1*
a3*' = a2*

a1* = a2*'
a2* = a3*'
a3* = a1*'

q1' = q1 - a3* = (a1,b1,0)
q2' = q2 = (a2,b2,0)

q1 = q1' + a1*' = (b1,0,a1+1)
q2 = q2' = (b2,0,a2)
