# Supplementary material for: Equivalence of superspace groups
Source: Acta Crystallogr A. 2012 Nov 14;69(Pt 1):75–90. doi: 10.1107/S0108767312041657 (PMC3553647; doi:10.1107/S0108767312041657)
Supplement: Supplementary file 1 [file a-69-00075-sup1.zip › ssg2d_c2221_urea_inclusion.pdf]

20.2.24.2 $C222_1$(1,0,g1)000(0,0,g2)000

Superspace group: 20.2.24.2 C222_1(1,0,g1)000(0,0,g2)000 [Y:2.209]
Bravais class: 2.24 Cmmm(1,0,g1)(0,0,g2) [JJdW:2.24]
Transformation to supercentered setting: A1=a1+a4, A2=a2, A3=a3, A4=a4, A5=a5

BASIC SPACE GROUP SETTING
Modulation vectors: q1=(1,0,g1), q2=(0,0,g2)
Centering: (0,0,0,0,0); (1/2,1/2,0,0,0)
Non-lattice generators: (x,-y,-z,2x-t,-u); (-x,y,-z+1/2,-t,-u); (-x,-y,z+1/2,-2x+t,u)
Non-lattice operators: (x,y,z,t,u); (x,-y,-z,2x-t,-u); (-x,y,-z+1/2,-t,-u); (-x,-y,z+1/2,-2x+t,u)

SUPERCENTERED SETTING
Modulation vectors: Q1=(0,0,G1), Q2=(0,0,G2), where G1=g1, G2=g2
Centering: (0,0,0,0,0); (1/2,1/2,0,1/2,0)
Non-lattice generators: (X,-Y,-Z,-T,-U); (-X,Y,-Z+1/2,-T,-U); (-X,-Y,Z+1/2,T,U)
Non-lattice operators: (X,Y,Z,T,U); (X,-Y,-Z,-T,-U); (-X,Y,-Z+1/2,-T,-U); (-X,-Y,Z+1/2,T,U)
Reflection conditions: HKLMN:H+K+M=2n; 00LMN:L=2n

This is the SSG of the urea inclusion compound nonadecane/urea.
No other SSG exists for this symmetry.
(0 0 g2) arises due to intermodulation of the mutually incommensurate host (urea) and guest (nonadecane) subsystems. g2 = c_host/c_guest = 0.428 is approx 3/7 at 0.5 GPa.
(1 0 g1) is an additional incommensurate modulation independent from the subsystem interaction. g1 = 0.143 is not 1/7 but incommensurate.

-----

findssg $C222_1$(1,0,g1)000(0,0,g2)000

Generators of the BSG setting have been entered into findssg.

Input setting

Centering
(0,0,0,0,0); (1/2,1/2,0,0,0)
Operators
(x,-y,-z,2x-t,-u); (-x,y,-z+1/2,-t,-u); (-x,-y,z+1/2,-2x+t,u); (x,y,z,t,u)

Standard settings

Superspace group: 20.2.24.2 C222_1(1,0,g1)000(0,0,g2)000 [Y:2.209]
Bravais class: 2.24 Cmmm(1,0,g1)(0,0,g2) [JJdW:2.24]
Transformation to supercentered setting: A1=a1+a4, A2=a2, A3=a3, A4=a4, A5=a5

BASIC SPACE GROUP SETTING
Modulation vectors: q1'=(1,0,g1), q2'=(0,0,g2)
Centering: (0,0,0,0,0); (1/2,1/2,0,0,0)
Non-lattice generators: (x,-y,-z,2x-t,-u); (-x,y,-z+1/2,-t,-u); (-x,-y,z+1/2,-2x+t,u)
Non-lattice operators: (x,y,z,t,u); (x,-y,-z,2x-t,-u); (-x,y,-z+1/2,-t,-u); (-x,-y,z+1/2,-2x+t,u)

SUPERCENTERED SETTING
Modulation vectors: Q1'=(0,0,G1), Q2'=(0,0,G2), where G1=g1, G2=g2
Centering: (0,0,0,0,0); (1/2,1/2,0,1/2,0)
Non-lattice generators: (X,-Y,-Z,-T,-U); (-X,Y,-Z+1/2,-T,-U); (-X,-Y,Z+1/2,T,U)
Non-lattice operators: (X,Y,Z,T,U); (X,-Y,-Z,-T,-U); (-X,Y,-Z+1/2,-T,-U); (-X,-Y,Z+1/2,T,U)
Reflection conditions: HKLMN:H+K+M=2n; 00LMN:L=2n

Affine transformation to standard basic space group setting

$S * g(input) * S^{-1} = g(standard)$,
where g is an augmented matrix for an operation in the superspace group.
Also, S * r(input) = r(standard),
where r is an augmented position vector, (x,y,z,t,u,1).

$$S = \begin{matrix} 1 & 0 & 0 & 0 & 0 & 0 \\ 0 & 1 & 0 & 0 & 0 & 0 \\ 0 & 0 & 1 & 0 & 0 & 0 \\ 0 & 0 & 0 & 1 & 0 & 0 \\ 0 & 0 & 0 & 0 & 1 & 0 \\ 0 & 0 & 0 & 0 & 0 & 1 \end{matrix} \quad S^{-1} = \begin{matrix} 1 & 0 & 0 & 0 & 0 & 0 \\ 0 & 1 & 0 & 0 & 0 & 0 \\ 0 & 0 & 1 & 0 & 0 & 0 \\ 0 & 0 & 0 & 1 & 0 & 0 \\ 0 & 0 & 0 & 0 & 1 & 0 \\ 0 & 0 & 0 & 0 & 0 & 1 \end{matrix}$$

a1' = a1
a2' = a2
a3' = a3

a1 = a1'
a2 = a2'
a3 = a3'

a1*' = a1*
a2*' = a2*
a3*' = a3*

a1* = a1*'
a2* = a2*'
a3* = a3*'

q1' = q1 = (1,0,g1)
q2' = q2 = (0,0,g2)

q1 = q1' = (1,0,g1)
q2 = q2' = (0,0,g2)

findssg X222$_1$(0,0,g1)000(0,0,g2)000

Generators of the supercentered setting of 20.2.24.2 have been entered into findssg.

Input setting

Centering
(0,0,0,0,0); (1/2,1/2,0,1/2,0)
Operators
(x,-y,-z,-t,-u); (-x,y,-z+1/2,-t,-u); (-x,-y,z+1/2,t,u); (x,y,z,t,u)

Standard settings

Superspace group: 20.2.24.2 C222_1(1,0,g1)000(0,0,g2)000 [Y:2.209]
Bravais class: 2.24 Cmmm(1,0,g1)(0,0,g2) [JJdW:2.24]
Transformation to supercentered setting: A1=a1+a4, A2=a2, A3=a3, A4=a4, A5=a5

BASIC SPACE GROUP SETTING
Modulation vectors: q1'=(1,0,g1), q2'=(0,0,g2)
Centering: (0,0,0,0,0); (1/2,1/2,0,0,0)
Non-lattice generators: (x,-y,-z,2x-t,-u); (-x,y,-z+1/2,-t,-u); (-x,-y,z+1/2,-2x+t,u)
Non-lattice operators: (x,y,z,t,u); (x,-y,-z,2x-t,-u); (-x,y,-z+1/2,-t,-u); (-x,-y,z+1/2,-2x+t,u)

SUPERCENTERED SETTING
Modulation vectors: Q1'=(0,0,G1), Q2'=(0,0,G2), where G1=g1, G2=g2
Centering: (0,0,0,0,0); (1/2,1/2,0,1/2,0)
Non-lattice generators: (X,-Y,-Z,-T,-U); (-X,Y,-Z+1/2,-T,-U); (-X,-Y,Z+1/2,T,U)
Non-lattice operators: (X,Y,Z,T,U); (X,-Y,-Z,-T,-U); (-X,Y,-Z+1/2,-T,-U); (-X,-Y,Z+1/2,T,U)
Reflection conditions: HKLMN:H+K+M=2n; 00LMN:L=2n

Affine transformation to standard basic space group setting

$S * g(input) * S^{-1} = g(standard)$,
where g is an augmented matrix for an operation in the superspace group.
Also, S * r(input) = r(standard),
where r is an augmented position vector, (x,y,z,t,u,1).

$$S = \begin{pmatrix} 1&0&0&0&0&0\\ 0&1&0&0&0&0\\ 0&0&1&0&0&0\\ 1&0&0&1&0&0\\ 0&0&0&0&1&0\\ 0&0&0&0&0&1 \end{pmatrix} \quad S^{-1} = \begin{pmatrix} 1&0&0&0&0&0\\ 0&1&0&0&0&0\\ 0&0&1&0&0&0\\ -1&0&0&1&0&0\\ 0&0&0&0&1&0\\ 0&0&0&0&0&1 \end{pmatrix}$$

a1' = a1
a2' = a2
a3' = a3

a1 = a1'
a2 = a2'
a3 = a3'

a1*' = a1*
a2*' = a2*
a3*' = a3*

a1* = a1*'
a2* = a2*'
a3* = a3*'

q1' = q1 + a1* = (1,0,g1)
q2' = q2 = (0,0,g2)

q1 = q1' - a1*' = (0,0,g1)
q2 = q2' = (0,0,g2)
