# Supplementary material for: Equivalence of superspace groups
Source: Acta Crystallogr A. 2012 Nov 14;69(Pt 1):75–90. doi: 10.1107/S0108767312041657 (PMC3553647; doi:10.1107/S0108767312041657)
Supplement: Supplementary file 1 [file a-69-00075-sup1.zip › ssg2d_fddd_nh4fe2p2o8.pdf]

70.2.56.6 Fddd(0,b,g)000(0,-b,g)000

Superspace group: 70.2.56.6 Fddd(0,b,g)000(0,-b,g)000 [Y:2.2433]
Bravais class: 2.56 Fmmm(0,b,g)(0,-b,g) [JJdW:2.56]
Transformation to supercentered setting: A1=a1, A2=a2, A3=a3, A4=a4-a5, A5=a4+a5

BASIC SPACE GROUP SETTING
Modulation vectors: q1=(0,b,g), q2=(0,-b,g)
Centering: (0,0,0,0,0); (0,1/2,1/2,0,0); (1/2,0,1/2,0,0); (1/2,1/2,0,0,0)
Non-lattice generators: (-x,y+1/4,z+1/4,t,u); (x+1/4,-y,z+1/4,u,t); (x+1/4,y+1/4,-z,-u,-t)
Non-lattice operators: (x,y,z,t,u); (x,-y+1/4,-z+1/4,-t,-u); (-x+1/4,y,-z+1/4,-u,-t); (-x+1/4,-y+1/4,z,u,t); (-x,-y,-z,-t,-u); (-x,y+1/4,z+1/4,t,u); (x+1/4,-y,z+1/4,u,t); (x+1/4,y+1/4,-z,-u,-t)

SUPERCENTERED SETTING
Modulation vectors: Q1=(0,B,0), Q2=(0,0,G), where B=b, G=g
Centering: (0,0,0,0,0); (0,1/2,1/2,0,0); (1/2,0,1/2,0,0); (1/2,1/2,0,0,0); (0,0,0,1/2,1/2); (0,1/2,1/2,1/2,1/2); (1/2,0,1/2,1/2,1/2); (1/2,1/2,0,1/2,1/2)
Non-lattice generators: (-X,Y+1/4,Z+1/4,T,U); (X+1/4,-Y,Z+1/4,-T,U); (X+1/4,Y+1/4,-Z,T,-U)
Non-lattice operators: (X,Y,Z,T,U); (X,-Y+1/4,-Z+1/4,-T,-U); (-X+1/4,Y,-Z+1/4,T,-U); (-X+1/4,-Y+1/4,Z,-T,U); (-X,-Y,-Z,-T,-U); (-X,Y+1/4,Z+1/4,T,U); (X+1/4,-Y,Z+1/4,-T,U); (X+1/4,Y+1/4,-Z,T,-U)
Reflection conditions: HKLMN:H+K=2n; HKLMN:H+L=2n; HKLMN:M+N=2n; 0KLMN:K+L=4n; HK0M0:H+K=4n; H0L0N:H+L=4n

-----

There exist just one SSG of this type.
SSG for beta-Nh4Fe2(PO4)2 with a commensurate modulation b=g=0.5
