# Supplementary material for: Equivalence of superspace groups
Source: Acta Crystallogr A. 2012 Nov 14;69(Pt 1):75–90. doi: 10.1107/S0108767312041657 (PMC3553647; doi:10.1107/S0108767312041657)
Supplement: Supplementary file 1 [file a-69-00075-sup1.zip › ssg2d_i2m_ab0_00c_bisrcoox_misfit.pdf]

15.2.14.4 B2/b(a1,b1,0)00(0,0,g2)00

Superspace group: 15.2.14.4 B2/b(a1,b1,0)00(0,0,g2)00 [Y:2.124]
Bravais class: 2.14 B2/m(a1,b1,0)(0,0,g2) [JJdW:2.14]
Transformation to supercentered setting: none
Modulation vectors: q1=(a1,b1,0), q2=(0,0,g2)
Centering: (0,0,0,0,0); (1/2,0,1/2,0,0)
Non-lattice generators: (-x,-y+1/2,z,-t,u); (x,y+1/2,-z,t,-u)
Non-lattice operators: (x,y,z,t,u); (-x,-y+1/2,z,-t,u); (-x,-y,-z,-t,-u); (x,y+1/2,-z,t,-u)
Reflection conditions: hklmn:h+l=2n; hk0m0:k=2n

15.2.14.5 B2/b(a1,b1,0)00(0,0,g2)s0

Superspace group: 15.2.14.5 B2/b(a1,b1,0)00(0,0,g2)s0 [Y:2.127]
Bravais class: 2.14 B2/m(a1,b1,0)(0,0,g2) [JJdW:2.14]
Transformation to supercentered setting: none
Modulation vectors: q1=(a1,b1,0), q2=(0,0,g2)
Centering: (0,0,0,0,0); (1/2,0,1/2,0,0)
Non-lattice generators: (-x,-y+1/2,z,-t,u+1/2); (x,y+1/2,-z,t,-u+1/2)
Non-lattice operators: (x,y,z,t,u); (-x,-y+1/2,z,-t,u+1/2); (-x,-y,-z,-t,-u); (x,y+1/2,-z,t,-u+1/2)
Reflection conditions: hklmn:h+l=2n; hk0m0:k=2n; 00l0n:n=2n

Two different SSG with the same Bravais class and the same BSG.
There is no supercentered setting.
15.2.14.4 is the symmetry of the misfit layer compound [Bi0.87SrO2]2[CoO2]1.82.

-----

findssg B2/b(a1,b1,0)00(0,0,g2)00

Operators of the standard BSG setting of 15.2.14.4 have been given to findssg.

Input setting

Centering
(0,0,0,0,0); (1/2,0,1/2,0,0)
Operators
(-x,-y+1/2,z,-t,u); (x,y+1/2,-z,t,-u); (x,y,z,t,u); (-x,-y,-z,-t,-u)

Standard settings

Superspace group: 15.2.14.4 B2/b(a1,b1,0)00(0,0,g2)00 [Y:2.124]
Bravais class: 2.14 B2/m(a1,b1,0)(0,0,g2) [JJdW:2.14]
Transformation to supercentered setting: none
Modulation vectors: q1'=(a1,b1,0), q2'=(0,0,g2)
Centering: (0,0,0,0,0); (1/2,0,1/2,0,0)
Non-lattice generators: (-x,-y+1/2,z,-t,u); (x,y+1/2,-z,t,-u)
Non-lattice operators: (x,y,z,t,u); (-x,-y+1/2,z,-t,u); (-x,-y,-z,-t,-u); (x,y+1/2,-z,t,-u)
Reflection conditions: hklmn:h+l=2n; hk0m0:k=2n

Affine transformation to standard basic space group setting

S * g(input) * S $^{-1}$ = g(standard),
where g is an augmented matrix for an operation in the superspace group.
Also, S * r(input) = r(standard),
where r is an augmented position vector, (x,y,z,t,u,1).

$$S = \begin{pmatrix} 1&0&0&0&0&0\\ 0&1&0&0&0&0\\ 0&0&1&0&0&0\\ 0&0&0&1&0&0\\ 0&0&0&0&1&0\\ 0&0&0&0&0&1 \end{pmatrix} \quad S^{-1} = \begin{pmatrix} 1&0&0&0&0&0\\ 0&1&0&0&0&0\\ 0&0&1&0&0&0\\ 0&0&0&1&0&0\\ 0&0&0&0&1&0\\ 0&0&0&0&0&1 \end{pmatrix}$$

a1' = a1		a3* = a3*'
a2' = a2	a1*' = a1*	
a3' = a3	a2*' = a2*	q1' = q1 = (a1,b1,0)
	a3*' = a3*	q2' = q2 = (0,0,g2)
a1 = a1'		
a2 = a2'	a1* = a1*'	q1 = q1' = (a1,b1,0)
a3 = a3'	a2* = a2*'	q2 = q2' = (0,0,g2)

findssg B2/b(a1,b1,0)00(0,0,g2)s0

Operators of the standard BSG setting of 15.2.14.5 have been given to findssg.

Input setting

Centering
(0,0,0,0,0); (1/2,0,1/2,0,0)
Operators
(-x,-y+1/2,z,-t,u+1/2); (-x,-y,-z,-t,-u); (x,y,z,t,u); (x,y+1/2,-z,t,-u+1/2)

Standard settings

Superspace group: 15.2.14.5 B2/b(a1,b1,0)00(0,0,g2)s0 [Y:2.127]
Bravais class: 2.14 B2/m(a1,b1,0)(0,0,g2) [JJdW:2.14]
Transformation to supercentered setting: none
Modulation vectors: q1'=(a1,b1,0), q2'=(0,0,g2)
Centering: (0,0,0,0,0); (1/2,0,1/2,0,0)
Non-lattice generators: (-x,-y+1/2,z,-t,u+1/2); (x,y+1/2,-z,t,-u+1/2)
Non-lattice operators: (x,y,z,t,u); (-x,-y+1/2,z,-t,u+1/2); (-x,-y,-z,-t,-u); (x,y+1/2,-z,t,-u+1/2)
Reflection conditions: hklmn:h+l=2n; hk0m0:k=2n; 00l0n:n=2n

Affine transformation to standard basic space group setting

$S * g(\text{input}) * S^{-1} = g(\text{standard})$,
where g is an augmented matrix for an operation in the superspace group.
Also, S * r(input) = r(standard),
where r is an augmented position vector, (x,y,z,t,u,1).

$$S = \begin{matrix} 1 & 0 & 0 & 0 & 0 & 0 \\ 0 & 1 & 0 & 0 & 0 & 0 \\ 0 & 0 & 1 & 0 & 0 & 0 \\ 0 & 0 & 0 & 1 & 0 & 0 \\ 0 & 0 & 0 & 0 & 1 & 0 \\ 0 & 0 & 0 & 0 & 0 & 1 \end{matrix} \qquad S^{-1} = \begin{matrix} 1 & 0 & 0 & 0 & 0 & 0 \\ 0 & 1 & 0 & 0 & 0 & 0 \\ 0 & 0 & 1 & 0 & 0 & 0 \\ 0 & 0 & 0 & 1 & 0 & 0 \\ 0 & 0 & 0 & 0 & 1 & 0 \\ 0 & 0 & 0 & 0 & 0 & 1 \end{matrix}$$

a1' = a1
a2' = a2
a3' = a3

a1 = a1'
a2 = a2'
a3 = a3'

a1*' = a1*
a2*' = a2*
a3*' = a3*

a1* = a1*'
a2* = a2*'
a3* = a3*'

q1' = q1 = (a1,b1,0)
q2' = q2 = (0,0,g2)

q1 = q1' = (a1,b1,0)
q2 = q2' = (0,0,g2)

findssg I2/a(a1,0,c1)00(0,b2,0)00

Operators of this alternate BSG setting of 15.2.14.4 have been given to findssg.

Input setting

Centering
(0,0,0,0,0); (1/2,1/2,1/2,0,0)
Operators
(-x+1/2,y,-z,-t,u); (-x,-y,-z,-t,-u); (x,y,z,t,u); (x+1/2,-y,z,t,-u)

Standard settings

Superspace group: 15.2.14.4 B2/b(a1,b1,0)00(0,0,g2)00 [Y:2.124]
Bravais class: 2.14 B2/m(a1,b1,0)(0,0,g2) [JJdW:2.14]
Transformation to supercentered setting: none
Modulation vectors: q1'=(a1,b1,0), q2'=(0,0,g2)
Centering: (0,0,0,0,0); (1/2,0,1/2,0,0)
Non-lattice generators: (-x,-y+1/2,z,-t,u); (x,y+1/2,-z,t,-u)
Non-lattice operators: (x,y,z,t,u); (-x,-y+1/2,z,-t,u); (-x,-y,-z,-t,-u); (x,y+1/2,-z,t,-u)
Reflection conditions: hklmn:h+l=2n; hk0m0:k=2n

Affine transformation to standard basic space group setting

$S * g(\text{input}) * S^{-1} = g(\text{standard})$,
where g is an augmented matrix for an operation in the superspace group.
Also, S * r(input) = r(standard),
where r is an augmented position vector, (x,y,z,t,u,1).

$$S = \begin{pmatrix} 0 & 0 & 1 & 0 & 0 & 0 \\ 1 & 0 & 1 & 0 & 0 & 0 \\ 0 & 1 & 0 & 0 & 0 & 0 \\ 0 & 0 & 0 & 1 & 0 & 0 \\ 0 & 0 & 0 & 0 & 1 & 0 \\ 0 & 0 & 0 & 0 & 0 & 1 \end{pmatrix} \quad S^{-1} = \begin{pmatrix} -1 & 1 & 0 & 0 & 0 & 0 \\ 0 & 0 & 1 & 0 & 0 & 0 \\ 1 & 0 & 0 & 0 & 0 & 0 \\ 0 & 0 & 0 & 1 & 0 & 0 \\ 0 & 0 & 0 & 0 & 1 & 0 \\ 0 & 0 & 0 & 0 & 0 & 1 \end{pmatrix}$$

a1' = -a1 + a3		a3* = a1*'
a2' = a1	a1*' = a3*	
a3' = a2	a2*' = a1* + a3*	q1' = q1 = (a1,b1,0)
	a3*' = a2*	q2' = q2 = (0,0,g2)
a1 = a2'		
a2 = a3'	a1* = -a1*' + a2*'	q1 = q1' = (b1,0,a1+b1)
a3 = a1' + a2'	a2* = a3*'	q2 = q2' = (0,g2,0)
