# Supplementary material for: Equivalence of superspace groups
Source: Acta Crystallogr A. 2012 Nov 14;69(Pt 1):75–90. doi: 10.1107/S0108767312041657 (PMC3553647; doi:10.1107/S0108767312041657)
Supplement: Supplementary file 1 [file a-69-00075-sup1.zip › ssg2d_i4mmm_a00_0a0_ni6snte2.pdf]

139.2.67.7 I4/mmm(a,0,0)0s00(0,a,0)0s00

Superspace group: 139.2.67.7 I4/mmm(a,0,0)0s00(0,a,0)0s00 [Y:2.3138]
Bravais class: 2.67 I4/mmm(a,0,0)(0,a,0) [JJdW:2.67]
Transformation to supercentered setting: none
Modulation vectors: q1=(a,0,0), q2=(0,a,0)
Centering: (0,0,0,0,0); (1/2,1/2,1/2,0,0)
Non-lattice generators: (-y,x,z,-u+1/2,t); (x,y,-z,t+1/2,u+1/2); (-x,y,z,-t+1/2,u); (y,x,z,u,t)
Non-lattice operators: (x,y,z,t,u); (x,-y,-z,t+1/2,-u); (-x,y,-z,-t,u+1/2); (-x,-y,z,-t+1/2,-u+1/2); (-y,-x,-z,-u,-t); (-y,x,z,-u+1/2,t); (y,-x,z,u,-t+1/2); (y,x,-z,u+1/2,t+1/2); (-x,-y,-z,-t,-u); (-x,y,z,-t+1/2,u); (x,-y,z,t,-u+1/2); (x,y,-z,t+1/2,u+1/2); (y,x,z,u,t); (y,-x,-z,u+1/2,-t); (-y,x,-z,-u,t+1/2); (-y,-x,z,-u+1/2,-t+1/2)
Reflection conditions: hklmn:h+k+l=2n; hk0mn:m+n=2n

Four SSG exists, that only differ in the intrinsic translational components along x4,x5:
139.2.67.6 I4/mmm(a,0,0)0000(0,a,0)0000
139.2.67.7 I4/mmm(a,0,0)0s00(0,a,0)0s00
139.2.67.8 I4/mmm(a,0,0)0000(0,a,0)00s0
139.2.67.9 I4/mmm(a,0,0)0s00(0,a,0)0ss0

This is the SSG of $Ni_{5.81(1)}SnTe_2$. Isaeva et al., JSSC 180, 221 (2007).
Published SSG is I4/mmm(0-a0, a00)0.ss.mm; symbol according to Yamamoto.

Yamamoto, from WEB site
2.3138 I4/mmm(0-p0,p00)0.ss.mm
(00000;1/21/21/200)
x,y,z,t,u; -x,-y,z,1/2-t,1/2-u; -y,x,z,1/2-u,t; y,-x,z,u,1/2-t; -x,y,-z,1/2+t,-u; x,-y,-z,-t,1/2+u; y,x,-z,-u,-t; -y,-x,-z,1/2+u,1/2+t; -x,-y,-z,-t,-u; x,y,-z,1/2+t,1/2+u; y,-x,-z,1/2+u,-t; -y,x,-z,-u,1/2+t; x,-y,z,1/2-t,u; -x,y,z,t,1/2-u; -y,-x,z,u,t; y,x,z,1/2-u,1/2-t;
hklmn:h+k+l=2n 0k0m0:m=2n h000n:n=2n h-h0nn:n+n=2n hk0mn:m+n=2n

-----

findssg I4/mmm(a,0,0)0s00(0,a,0)0s00

Generators of standard BSG setting entered into findssg.

Input setting

Centering
(0,0,0,0,0); (1/2,1/2,1/2,0,0)
Operators
(-y,x,z,-u+1/2,t); (x,y,-z,t+1/2,u+1/2); (-x,y,z,-t+1/2,u); (y,x,z,u,t); (-x,-y,z,-t+1/2,-u+1/2); (-y,x,-z,-u,t+1/2); (-y,-x,z,-u+1/2,-t+1/2); (x,y,z,t,u); (-x,y,-z,-t,u+1/2); (y,x,-z,u+1/2,t+1/2); (-x,-y,-z,-t,-u); (-y,-x,-z,-u,-t); (x,-y,z,t,-u+1/2); (y,-x,z,u,-t+1/2); (x,-y,-z,t+1/2,-u); (y,-x,-z,u+1/2,-t)

Standard settings

Superspace group: 139.2.67.7 I4/mmm(a,0,0)0s00(0,a,0)0s00 [Y:2.3138]
Bravais class: 2.67 I4/mmm(a,0,0)(0,a,0) [JJdW:2.67]
Transformation to supercentered setting: none
Modulation vectors: q1'=(a,0,0), q2'=(0,a,0)
Centering: (0,0,0,0,0); (1/2,1/2,1/2,0,0)
Non-lattice generators: (-y,x,z,-u+1/2,t); (x,y,-z,t+1/2,u+1/2); (-x,y,z,-t+1/2,u); (y,x,z,u,t)
Non-lattice operators: (x,y,z,t,u); (x,-y,-z,t+1/2,-u); (-x,y,-z,-t,u+1/2); (-x,-y,z,-t+1/2,-u+1/2); (-y,-x,-z,-u,-t); (-y,x,z,-u+1/2,t); (y,-x,z,u,-t+1/2); (y,x,-z,u+1/2,t+1/2); (-x,-y,-z,-t,-u); (-x,y,z,-t+1/2,u); (x,-y,z,t,-u+1/2); (x,y,-z,t+1/2,u+1/2); (y,x,z,u,t); (y,-x,-z,u+1/2,-t); (-y,x,-z,-u,t+1/2); (-y,-x,z,-u+1/2,-t+1/2)
Reflection conditions: hklmn:h+k+l=2n; hk0mn:m+n=2n

Affine transformation to standard basic space group setting

S * g(input) * S^{-1} = g(standard),
where g is an augmented matrix for an operation in the superspace group.
Also, S * r(input) = r(standard),
where r is an augmented position vector, (x,y,z,t,u,1).

$$S = \begin{matrix} 1 & 0 & 0 & 0 & 0 & 0 \\ 0 & 1 & 0 & 0 & 0 & 0 \\ 0 & 0 & 1 & 0 & 0 & 0 \\ 0 & 0 & 0 & 1 & 0 & 0 \\ 0 & 0 & 0 & 0 & 1 & 0 \\ 0 & 0 & 0 & 0 & 0 & 1 \end{matrix} \quad S^{-1} = \begin{matrix} 1 & 0 & 0 & 0 & 0 & 0 \\ 0 & 1 & 0 & 0 & 0 & 0 \\ 0 & 0 & 1 & 0 & 0 & 0 \\ 0 & 0 & 0 & 1 & 0 & 0 \\ 0 & 0 & 0 & 0 & 1 & 0 \\ 0 & 0 & 0 & 0 & 0 & 1 \end{matrix}$$

a1' = a1	a3 = a3'	a1* = a1*'	
a2' = a2		a2* = a2*'	q1 = q1' = (a,0,0)
a3' = a3	a1*' = a1*	a3* = a3*'	q2 = q2' = (0,a,0)
	a2*' = a2*		
a1 = a1'	a3*' = a3*	q1' = q1 = (a,0,0)	
a2 = a2'		q2' = q2 = (0,a,0)	

findssg 2.3138 I4/mmm(0-p0,p00)0.ss.mm

Operators of Yamamoto entered into findssg.

Input setting

Centering
(0,0,0,0,0); (1/2,1/2,1/2,0,0)
Operators
(-x,-y,z,-t+1/2,-u+1/2); (-y,x,z,-u+1/2,t); (y,-x,z,u,-t+1/2); (-x,y,-z,t+1/2,-u); (x,-y,-z,-t,u+1/2); (y,x,-z,-u,-t); (-y,-x,-z,u+1/2,t+1/2); (-x,-y,-z,-t,-u); (x,y,-z,t+1/2,u+1/2); (y,-x,-z,u+1/2,-t); (-y,x,-z,-u,t+1/2); (x,-y,z,-t+1/2,u); (-x,y,z,t,-u+1/2); (-y,-x,z,u,t); (y,x,z,-u+1/2,-t+1/2); (x,y,z,t,u)

Standard settings

Superspace group: 139.2.67.7 I4/mmm(a,0,0)0s00(0,a,0)0s00 [Y:2.3138]
Bravais class: 2.67 I4/mmm(a,0,0)(0,a,0) [JJdW:2.67]
Transformation to supercentered setting: none
Modulation vectors: q1'=(a,0,0), q2'=(0,a,0)
Centering: (0,0,0,0,0); (1/2,1/2,1/2,0,0)
Non-lattice generators: (-y,x,z,-u+1/2,t); (x,y,-z,t+1/2,u+1/2); (-x,y,z,-t+1/2,u); (y,x,z,u,t)
Non-lattice operators: (x,y,z,t,u); (x,-y,-z,t+1/2,-u); (-x,y,-z,-t,u+1/2); (-x,-y,z,-t+1/2,-u+1/2); (-y,-x,-z,-u,-t); (-y,x,z,-u+1/2,t); (y,-x,z,u,-t+1/2); (y,x,-z,u+1/2,t+1/2); (-x,-y,-z,-t,-u); (-x,y,z,-t+1/2,u); (x,-y,z,t,-u+1/2); (x,y,-z,t+1/2,u+1/2); (y,x,z,u,t); (y,-x,-z,u+1/2,-t); (-y,x,-z,-u,t+1/2); (-y,-x,z,-u+1/2,-t+1/2)
Reflection conditions: hklmn:h+k+l=2n; hk0mn:m+n=2n

Affine transformation to standard basic space group setting

$S * g(input) * S^{-1} = g(standard)$,
where g is an augmented matrix for an operation in the superspace group.
Also, S * r(input) = r(standard),
where r is an augmented position vector, (x,y,z,t,u,1).

$$S = \begin{matrix} 0 & 1 & 0 & 0 & 0 & 0 \\ -1 & 0 & 0 & 0 & 0 & 0 \\ 0 & 0 & 1 & 0 & 0 & 0 \\ 0 & 0 & 0 & 1 & 0 & 0 \\ 0 & 0 & 0 & 0 & 1 & 0 \\ 0 & 0 & 0 & 0 & 0 & 1 \end{matrix} \qquad S^{-1} = \begin{matrix} 0 & -1 & 0 & 0 & 0 & 0 \\ 1 & 0 & 0 & 0 & 0 & 0 \\ 0 & 0 & 1 & 0 & 0 & 0 \\ 0 & 0 & 0 & 1 & 0 & 0 \\ 0 & 0 & 0 & 0 & 1 & 0 \\ 0 & 0 & 0 & 0 & 0 & 1 \end{matrix}$$

a1' = a2	a3 = a3'	a1* = -a2*'	
a2' = -a1		a2* = a1*'	q1 = q1' = (0,a,0)
a3' = a3	a1*' = a2*	a3* = a3*'	q2 = q2' = (-a,0,0)
	a2*' = -a1*		
a1 = -a2'	a3*' = a3*	q1' = q1 = (a,0,0)	
a2 = a1'		q2' = q2 = (0,a,0)	

transformssg

Input setting

Centering
none
Operators
(-y,x,z,-u+1/2,t); (x,y,-z,t+1/2,u+1/2); (-x,y,z,-t+1/2,u); (y,x,z,u,t); (-x,-y,z,-t+1/2,-u+1/2); (-y,x,-z,-u,t+1/2); (-y,-x,z,-u+1/2,-t+1/2); (x,y,z,t,u); (-x,y,-z,-t,u+1/2); (y,x,-z,u+1/2,t+1/2); (-x,-y,-z,-t,-u); (-y,-x,-z,-u,-t); (x,-y,z,t,-u+1/2); (y,-x,z,u,-t+1/2); (x,-y,-z,t+1/2,-u); (y,-x,-z,u+1/2,-t)
q vectors
$\mathbf{q}_1$=(-0.410,0,0); $\mathbf{q}_2$=(0,-0.410,0)

New setting

Centering
none
Operators
(-y,x,z,-u+1/2,t); (x,y,-z,t+1/2,u+1/2); (-x,y,z,-t+1/2,u); (y,x,z,u,t); (-x,-y,z,-t+1/2,-u+1/2); (-y,x,-z,-u,t+1/2); (-y,-x,z,-u+1/2,-t+1/2); (x,y,z,t,u); (-x,y,-z,-t,u+1/2); (y,x,-z,u+1/2,t+1/2); (-x,-y,-z,-t,-u); (-y,-x,-z,-u,-t); (x,-y,z,t,-u+1/2); (y,-x,z,u,-t+1/2); (x,-y,-z,t+1/2,-u); (y,-x,-z,u+1/2,-t)
q vectors
$\mathbf{q}_1'$=(0.410,0,0); $\mathbf{q}_2'$=(0,0.410,0)

Affine transformation to new setting

$$S = \begin{pmatrix} -1 & 0 & 0 & 0 & 0 & 0 \\ 0 & -1 & 0 & 0 & 0 & 0 \\ 0 & 0 & 1 & 0 & 0 & 0 \\ 0 & 0 & 0 & 1 & 0 & 0 \\ 0 & 0 & 0 & 0 & 1 & 0 \\ 0 & 0 & 0 & 0 & 0 & 1 \end{pmatrix} \quad S^{-1} = \begin{pmatrix} -1 & 0 & 0 & 0 & 0 & 0 \\ 0 & -1 & 0 & 0 & 0 & 0 \\ 0 & 0 & 1 & 0 & 0 & 0 \\ 0 & 0 & 0 & 1 & 0 & 0 \\ 0 & 0 & 0 & 0 & 1 & 0 \\ 0 & 0 & 0 & 0 & 0 & 1 \end{pmatrix}$$

g' = S * g * S $^{-1}$, where g is an augmented matrix for an operation in the superspace group.
r' = S * **r**, where **r** is an augmented position vector, (x,y,z,t,u,1).

Basis vectors of the lattice
$\mathbf{a}_1' = -\mathbf{a}_1$; $\mathbf{a}_2' = -\mathbf{a}_2$; $\mathbf{a}_3' = \mathbf{a}_3$
$\mathbf{a}_1 = -\mathbf{a}_1'$; $\mathbf{a}_2 = -\mathbf{a}_2'$; $\mathbf{a}_3 = \mathbf{a}_3'$
Basis vectors of the reciprocal lattice
$\mathbf{a}_1^{*\prime} = -\mathbf{a}_1^*$; $\mathbf{a}_2^{*\prime} = -\mathbf{a}_2^*$; $\mathbf{a}_3^{*\prime} = \mathbf{a}_3^*$
$\mathbf{a}_1^* = -\mathbf{a}_1^{*\prime}$; $\mathbf{a}_2^* = -\mathbf{a}_2^{*\prime}$; $\mathbf{a}_3^* = \mathbf{a}_3^{*\prime}$

q vectors
$\mathbf{q}_1' = \mathbf{q}_1$ = (0.410,0,0); $\mathbf{q}_2' = \mathbf{q}_2$ = (0,0.410,0)
$\mathbf{q}_1 = \mathbf{q}_1'$ = (-0.410,0,0); $\mathbf{q}_2 = \mathbf{q}_2'$ = (0,-0.410,0)

Origin
$\boldsymbol{\tau}' = 0$
$\boldsymbol{\tau} = 0$
