# Supplementary material for: Equivalence of superspace groups
Source: Acta Crystallogr A. 2012 Nov 14;69(Pt 1):75–90. doi: 10.1107/S0108767312041657 (PMC3553647; doi:10.1107/S0108767312041657)
Supplement: Supplementary file 1 [file a-69-00075-sup1.zip › ssg2d_i4_g1g2_qs_q0.pdf]

79.2.62.3 I4(0,0,g1)q(0,0,g2)0

Superspace group: 79.2.62.3 I4(0,0,g1)q(0,0,g2)0 [Y:2.2562]
Bravais class: 2.62 I4/mmm(0,0,g1)(0,0,g2) [JJdW:2.62]
Transformation to supercentered setting: none
Modulation vectors: q1=(0,0,g1), q2=(0,0,g2)
Centering: (0,0,0,0,0); (1/2,1/2,1/2,0,0)
Non-lattice generators: (-y,x,z,t+1/4,u)
Non-lattice operators: (x,y,z,t,u); (-x,-y,z,t+1/2,u); (-y,x,z,t+1/4,u); (y,-x,z,t+3/4,u)
Reflection conditions: hklmn:h+k+l=2n; 00lmn:m=4n

No supercentered setting.

-----

findssg I4(0,0,g1)q(0,0,g2)0

Generators of BSG setting have been entered into findssg.

Input setting

Centering
(0,0,0,0,0); (1/2,1/2,1/2,0,0)
Operators
(-y,x,z,t+1/4,u); (-x,-y,z,t+1/2,u); (y,-x,z,t+3/4,u); (x,y,z,t,u)

Standard settings

Superspace group: 79.2.62.3 I4(0,0,g1)q(0,0,g2)0 [Y:2.2562]
Bravais class: 2.62 I4/mmm(0,0,g1)(0,0,g2) [JJdW:2.62]
Transformation to supercentered setting: none
Modulation vectors: q1'=(0,0,g1), q2'=(0,0,g2)
Centering: (0,0,0,0,0); (1/2,1/2,1/2,0,0)
Non-lattice generators: (-y,x,z,t+1/4,u)
Non-lattice operators: (x,y,z,t,u); (-x,-y,z,t+1/2,u); (-y,x,z,t+1/4,u); (y,-x,z,t+3/4,u)
Reflection conditions: hklmn:h+k+l=2n; 00lmn:m=4n

Affine transformation to standard basic space group setting

S * g(input) * S $^{-1}$ = g(standard),
where g is an augmented matrix for an operation in the superspace group.
Also, S * r(input) = r(standard),
where r is an augmented position vector, (x,y,z,t,u,1).

$$S = \begin{matrix} 1&0&0&0&0&0\\ 0&1&0&0&0&0\\ 0&0&1&0&0&0\\ 0&0&0&1&0&0\\ 0&0&0&0&1&0\\ 0&0&0&0&0&1 \end{matrix} \qquad S^{-1} = \begin{matrix} 1&0&0&0&0&0\\ 0&1&0&0&0&0\\ 0&0&1&0&0&0\\ 0&0&0&1&0&0\\ 0&0&0&0&1&0\\ 0&0&0&0&0&1 \end{matrix}$$

a1' = a1		a3* = a3*'
a2' = a2	a1*' = a1*	
a3' = a3	a2*' = a2*	q1' = q1 = (0,0,g1)
	a3*' = a3*	q2' = q2 = (0,0,g2)
a1 = a1'		
a2 = a2'	a1* = a1*'	q1 = q1' = (0,0,g1)
a3 = a3'	a2* = a2*'	q2 = q2' = (0,0,g2)

findssg I4(0,0,g1)q(0,0,g2)s

Input setting

Generators of this alternate setting have been entered into findssg.

Input setting

Centering
(0,0,0,0,0); (1/2,1/2,1/2,0,0)
Operators
(-y,x,z,t+1/4,u+1/2); (-x,-y,z,t+1/2,u); (y,-x,z,t+3/4,u+1/2); (x,y,z,t,u)

Standard settings

Superspace group: 79.2.62.3 I4(0,0,g1)q(0,0,g2)0 [Y:2.2562]
Bravais class: 2.62 I4/mmm(0,0,g1)(0,0,g2) [JJdW:2.62]
Transformation to supercentered setting: none
Modulation vectors: q1'=(0,0,g1), q2'=(0,0,g2)
Centering: (0,0,0,0,0); (1/2,1/2,1/2,0,0)
Non-lattice generators: (-y,x,z,t+1/4,u)
Non-lattice operators: (x,y,z,t,u); (-x,-y,z,t+1/2,u); (-y,x,z,t+1/4,u); (y,-x,z,t+3/4,u)
Reflection conditions: hklmn:h+k+l=2n; 00lmn:m=4n

Affine transformation to standard basic space group setting

$S * g(\text{input}) * S^{-1} = g(\text{standard})$,
where g is an augmented matrix for an operation in the superspace group.
Also, S * r(input) = r(standard),
where r is an augmented position vector, (x,y,z,t,u,1).

$$S = \begin{matrix} 1 & 0 & 0 & 0 & 0 & 0 \\ 0 & 1 & 0 & 0 & 0 & 0 \\ 0 & 0 & 1 & 0 & 0 & 0 \\ 0 & 0 & 0 & 1 & 0 & 0 \\ 0 & 0 & 0 & 2 & 1 & 0 \\ 0 & 0 & 0 & 0 & 0 & 1 \end{matrix} \qquad S^{-1} = \begin{matrix} 1 & 0 & 0 & 0 & 0 & 0 \\ 0 & 1 & 0 & 0 & 0 & 0 \\ 0 & 0 & 1 & 0 & 0 & 0 \\ 0 & 0 & 0 & 1 & 0 & 0 \\ 0 & 0 & 0 & -2 & 1 & 0 \\ 0 & 0 & 0 & 0 & 0 & 1 \end{matrix}$$

a1' = a1		a3* = a3*'
a2' = a2	a1*' = a1*	
a3' = a3	a2*' = a2*	q1' = q1 = (0,0,g1)
	a3*' = a3*	q2' = 2 q1 + q2 = (0,0,g2)
a1 = a1'		
a2 = a2'	a1* = a1*'	q1 = q1' = (0,0,g1)
a3 = a3'	a2* = a2*'	q2 = -2 q1' + q2' = (0,0,-2g1+g2)
