# Supplementary material for: Equivalence of superspace groups
Source: Acta Crystallogr A. 2012 Nov 14;69(Pt 1):75–90. doi: 10.1107/S0108767312041657 (PMC3553647; doi:10.1107/S0108767312041657)
Supplement: Supplementary file 1 [file a-69-00075-sup1.zip › ssg2d_p-1_mo2s3.pdf]

2.2.1.1 P-1(a1,b1,g1)0(a2,b2,g2)0

Superspace group: 2.2.1.1 P-1(a1,b1,g1)0(a2,b2,g2)0 [Y:2.2]
Bravais class: 2.1 P-1(a1,b1,g1)(a2,b2,g2)   [JJdW:2.1]
Transformation to supercentered setting: none
Modulation vectors: q1=(a1,b1,g1), q2=(a2,b2,g2)
Centering: (0,0,0,0,0)
Non-lattice generators: (-x,-y,-z,-t,-u)
Non-lattice operators: (x,y,z,t,u); (-x,-y,-z,-t,-u)
Reflection conditions: none

There is no supercentered setting, i.e. this is a primitive superspace lattice.
This is the symmetry of Mo2S3 : W. J. Schutte, F. Disselborg and J. L. De Boer, Acta Crystallogr. B 49, 787-794 (1993).   There is only one possible SSG.
-----

findssg P-1(a1,b1,g1)0(a2,b2,g2)0

Input setting

Centering
none
Operators
(-x,-y,-z,-t,-u); (x,y,z,t,u)

Standard settings

Superspace group: 2.2.1.1 P-1(a1,b1,g1)0(a2,b2,g2)0 [Y:2.2]
Bravais class: 2.1 P-1(a1,b1,g1)(a2,b2,g2)   [JJdW:2.1]
Transformation to supercentered setting: none
Modulation vectors: q1'=(a1,b1,g1), q2'=(a2,b2,g2)
Centering: (0,0,0,0,0)
Non-lattice generators: (-x,-y,-z,-t,-u)
Non-lattice operators: (x,y,z,t,u); (-x,-y,-z,-t,-u)
Reflection conditions: none

Affine transformation to standard basic space group setting

$S * g(input) * S^{-1} = g(standard)$,
where g is an augmented matrix for an operation in the superspace group.
Also, S * r(input) = r(standard),
where r is an augmented position vector, (x,y,z,t,u,1).

$$S = \begin{pmatrix} 1&0&0&0&0&0\\ 0&1&0&0&0&0\\ 0&0&1&0&0&0\\ 0&0&0&1&0&0\\ 0&0&0&0&1&0\\ 0&0&0&0&0&1 \end{pmatrix} \quad S^{-1} = \begin{pmatrix} 1&0&0&0&0&0\\ 0&1&0&0&0&0\\ 0&0&1&0&0&0\\ 0&0&0&1&0&0\\ 0&0&0&0&1&0\\ 0&0&0&0&0&1 \end{pmatrix}$$

a1' = a1
a2' = a2
a3' = a3

a1 = a1'
a2 = a2'
a3 = a3'

a1*' = a1*
a2*' = a2*
a3*' = a3*

a1* = a1*'
a2* = a2*'
a3* = a3*'

q1' = q1 = (a1,b1,g1)
q2' = q2 = (a2,b2,g2)

q1 = q1' = (a1,b1,g1)
q2 = q2' = (a2,b2,g2)
