# Supplementary material for: Equivalence of superspace groups
Source: Acta Crystallogr A. 2012 Nov 14;69(Pt 1):75–90. doi: 10.1107/S0108767312041657 (PMC3553647; doi:10.1107/S0108767312041657)
Supplement: Supplementary file 1 [file a-69-00075-sup1.zip › ssg2d_p212121_ab0_p4w8o32.pdf]

19.2.50.3 $P2_12_12_1(0,b,g)000(0,-b,g)000$

Superspace group: 19.2.50.3 P2_12_12_1(0,b,g)000(0,-b,g)000 [Y:2.199]
Bravais class: 2.50 Pmmm(0,b,g)(0,-b,g)   [JJdW:2.50]
Transformation to supercentered setting: A1=a1, A2=a2, A3=a3, A4=a4-a5, A5=a4+a5

BASIC SPACE GROUP SETTING
Modulation vectors: q1=(0,b,g), q2=(0,-b,g)
Centering: (0,0,0,0,0)
Non-lattice generators: (x+1/2,-y+1/2,-z,-t,-u); (-x,y+1/2,-z+1/2,-u,-t); (-x+1/2,-y,z+1/2,u,t)
Non-lattice operators: (x,y,z,t,u); (x+1/2,-y+1/2,-z,-t,-u); (-x,y+1/2,-z+1/2,-u,-t); (-x+1/2,-y,z+1/2,u,t)

SUPERCENTERED SETTING
Modulation vectors: Q1=(0,B,0), Q2=(0,0,G), where B=b, G=g
Centering: (0,0,0,0,0); (0,0,0,1/2,1/2)
Non-lattice generators: (X+1/2,-Y+1/2,-Z,-T,-U); (-X,Y+1/2,-Z+1/2,T,-U); (-X+1/2,-Y,Z+1/2,-T,U)
Non-lattice operators: (X,Y,Z,T,U); (X+1/2,-Y+1/2,-Z,-T,-U); (-X,Y+1/2,-Z+1/2,T,-U); (-X+1/2,-Y,Z+1/2,-T,U)
Reflection conditions: HKLMN:M+N=2n; 0K0M0:K=2n; 00L0N:L=2n; H0000:H=2n

-----

This is the symmetry of (PO2)4(WO3)8 (Ludecke 2001).
There is only one SSG with the same BSG in this Bravais class.

-----

findssg $P2_12_12_1(0,b,g)000(0,-b,g)000$

Generators of the standard BSG setting have been entered into findssg.

Input setting

Centering
none
Operators
(x+1/2,-y+1/2,-z,-t,-u); (-x,y+1/2,-z+1/2,-u,-t); (x,y,z,t,u); (-x+1/2,-y,z+1/2,u,t)

Standard settings

Superspace group: 19.2.50.3 P2_12_12_1(0,b,g)000(0,-b,g)000 [Y:2.199]
Bravais class: 2.50 Pmmm(0,b,g)(0,-b,g)   [JJdW:2.50]
Transformation to supercentered setting: A1=a1, A2=a2, A3=a3, A4=a4-a5, A5=a4+a5

BASIC SPACE GROUP SETTING
Modulation vectors: q1'=(0,b,g), q2'=(0,-b,g)
Centering: (0,0,0,0,0)
Non-lattice generators: (x+1/2,-y+1/2,-z,-t,-u); (-x,y+1/2,-z+1/2,-u,-t); (-x+1/2,-y,z+1/2,u,t)
Non-lattice operators: (x,y,z,t,u); (x+1/2,-y+1/2,-z,-t,-u); (-x,y+1/2,-z+1/2,-u,-t); (-x+1/2,-y,z+1/2,u,t)

SUPERCENTERED SETTING
Modulation vectors: Q1'=(0,B,0), Q2'=(0,0,G), where B=b, G=g
Centering: (0,0,0,0,0); (0,0,0,1/2,1/2)
Non-lattice generators: (X+1/2,-Y+1/2,-Z,-T,-U); (-X,Y+1/2,-Z+1/2,T,-U); (-X+1/2,-Y,Z+1/2,-T,U)
Non-lattice operators: (X,Y,Z,T,U); (X+1/2,-Y+1/2,-Z,-T,-U); (-X,Y+1/2,-Z+1/2,T,-U); (-X+1/2,-Y,Z+1/2,-T,U)
Reflection conditions: HKLMN:M+N=2n; 0K0M0:K=2n; 00L0N:L=2n; H0000:H=2n

Affine transformation to standard basic space group setting

$S * g(input) * S^{-1} = g(standard)$,
where g is an augmented matrix for an operation in the superspace group.
Also, S * r(input) = r(standard),
where r is an augmented position vector, (x,y,z,t,u,1).

$$S = \begin{pmatrix} 1&0&0&0&0&0\\ 0&1&0&0&0&0\\ 0&0&1&0&0&0\\ 0&0&0&1&0&0\\ 0&0&0&0&1&0\\ 0&0&0&0&0&1 \end{pmatrix} \quad S^{-1} = \begin{pmatrix} 1&0&0&0&0&0\\ 0&1&0&0&0&0\\ 0&0&1&0&0&0\\ 0&0&0&1&0&0\\ 0&0&0&0&1&0\\ 0&0&0&0&0&1 \end{pmatrix}$$

a1' = a1
a2' = a2
a3' = a3

a1 = a1'
a2 = a2'
a3 = a3'

a1*' = a1*
a2*' = a2*
a3*' = a3*

a1* = a1*'
a2* = a2*'
a3* = a3*'

q1' = q1 = (0,b,g)
q2' = q2 = (0,-b,g)

q1 = q1' = (0,b,g)
q2 = q2' = (0,-b,g)

findssg $X2_12_12_1(0,b,0)000(0,0,g)000$

Generators of the standard supercentered setting have been entered into findssg.

Input setting

Centering
(0,0,0,0,0); (0,0,0,1/2,1/2)
Operators
(x+1/2,-y+1/2,-z,-t,-u); (-x,y+1/2,-z+1/2,t,-u); (x,y,z,t,u); (-x+1/2,-y,z+1/2,-t,u)

Standard settings

Superspace group: 19.2.50.3 P2_12_12_1(0,b,g)000(0,-b,g)000 [Y:2.199]
Bravais class: 2.50 Pmmm(0,b,g)(0,-b,g) [JJdW:2.50]
Transformation to supercentered setting: A1=a1, A2=a2, A3=a3, A4=a4-a5, A5=a4+a5

BASIC SPACE GROUP SETTING
Modulation vectors: q1'=(0,b,g), q2'=(0,-b,g)
Centering: (0,0,0,0,0)
Non-lattice generators: (x+1/2,-y+1/2,-z,-t,-u); (-x,y+1/2,-z+1/2,-u,-t); (-x+1/2,-y,z+1/2,u,t)
Non-lattice operators: (x,y,z,t,u); (x+1/2,-y+1/2,-z,-t,-u); (-x,y+1/2,-z+1/2,-u,-t); (-x+1/2,-y,z+1/2,u,t)

SUPERCENTERED SETTING
Modulation vectors: Q1'=(0,B,0), Q2'=(0,0,G), where B=b, G=g
Centering: (0,0,0,0,0); (0,0,0,1/2,1/2)
Non-lattice generators: (X+1/2,-Y+1/2,-Z,-T,-U); (-X,Y+1/2,-Z+1/2,T,-U); (-X+1/2,-Y,Z+1/2,-T,U)
Non-lattice operators: (X,Y,Z,T,U); (X+1/2,-Y+1/2,-Z,-T,-U); (-X,Y+1/2,-Z+1/2,T,-U); (-X+1/2,-Y,Z+1/2,-T,U)
Reflection conditions: HKLMN:M+N=2n; 0K0M0:K=2n; 00L0N:L=2n; H0000:H=2n

Affine transformation to standard basic space group setting

$S * g(\text{input}) * S^{-1} = g(\text{standard})$,
where g is an augmented matrix for an operation in the superspace group.
Also, $S * r(\text{input}) = r(\text{standard})$,
where r is an augmented position vector, (x,y,z,t,u,1).

$$S = \begin{array}{cccccc} 1 & 0 & 0 & 0 & 0 & 0 \\ 0 & 1 & 0 & 0 & 0 & 0 \\ 0 & 0 & 1 & 0 & 0 & 0 \\ 0 & 0 & 0 & -1 & 1 & 0 \\ 0 & 0 & 0 & 1 & 1 & 0 \\ 0 & 0 & 0 & 0 & 0 & 1 \end{array} \quad S^{-1} = \begin{array}{cccccc} 1 & 0 & 0 & 0 & 0 & 0 \\ 0 & 1 & 0 & 0 & 0 & 0 \\ 0 & 0 & 1 & 0 & 0 & 0 \\ 0 & 0 & 0 & -1/2 & 1/2 & 0 \\ 0 & 0 & 0 & 1/2 & 1/2 & 0 \\ 0 & 0 & 0 & 0 & 0 & 1 \end{array}$$

a1' = a1
a2' = a2
a3' = a3

a1 = a1'
a2 = a2'
a3 = a3'

a1*' = a1*
a2*' = a2*
a3*' = a3*

a1* = a1*'
a2* = a2*'
a3* = a3*'

q1' = -q1 + q2 = (0,b,g)
q2' = q1 + q2 = (0,-b,g)

q1 = -1/2 q1' + 1/2 q2' = (0,-b,0)
q2 = 1/2 q1' + 1/2 q2' = (0,0,g)

findssg $P2_12_12_1(a,b,0)000(-a,b,0)000$

Generators of the published SSG have been entered into findssg.

Input setting

Centering
none
Operators
(x+1/2,-y+1/2,-z,-u,-t); (-x,y+1/2,-z+1/2,u,t); (x,y,z,t,u); (-x+1/2,-y,z+1/2,-t,-u)

Standard settings

Superspace group: 19.2.50.3 P2_12_12_1(0,b,g)000(0,-b,g)000 [Y:2.199]
Bravais class: 2.50 Pmmm(0,b,g)(0,-b,g) [JJdW:2.50]
Transformation to supercentered setting: A1=a1, A2=a2, A3=a3, A4=a4-a5, A5=a4+a5

BASIC SPACE GROUP SETTING
Modulation vectors: q1'=(0,b,g), q2'=(0,-b,g)
Centering: (0,0,0,0,0)
Non-lattice generators: (x+1/2,-y+1/2,-z,-t,-u); (-x,y+1/2,-z+1/2,-u,-t); (-x+1/2,-y,z+1/2,u,t)
Non-lattice operators: (x,y,z,t,u); (x+1/2,-y+1/2,-z,-t,-u); (-x,y+1/2,-z+1/2,-u,-t); (-x+1/2,-y,z+1/2,u,t)

SUPERCENTERED SETTING
Modulation vectors: Q1'=(0,B,0), Q2'=(0,0,G), where B=b, G=g
Centering: (0,0,0,0,0); (0,0,0,1/2,1/2)
Non-lattice generators: (X+1/2,-Y+1/2,-Z,-T,-U); (-X,Y+1/2,-Z+1/2,T,-U); (-X+1/2,-Y,Z+1/2,-T,U)
Non-lattice operators: (X,Y,Z,T,U); (X+1/2,-Y+1/2,-Z,-T,-U); (-X,Y+1/2,-Z+1/2,T,-U); (-X+1/2,-Y,Z+1/2,-T,U)
Reflection conditions: HKLMN:M+N=2n; 0K0M0:K=2n; 00L0N:L=2n; H0000:H=2n

Affine transformation to standard basic space group setting

$S * g(input) * S^{-1} = g(standard)$,
where g is an augmented matrix for an operation in the superspace group.
Also, S * r(input) = r(standard),
where r is an augmented position vector, (x,y,z,t,u,1).

$$S = \begin{pmatrix} 0&0&1&0&0&0 \\ 1&0&0&0&0&0 \\ 0&1&0&0&0&0 \\ 0&0&0&1&0&0 \\ 0&0&0&0&1&0 \\ 0&0&0&0&0&1 \end{pmatrix} \quad S^{-1} = \begin{pmatrix} 0&1&0&0&0&0 \\ 0&0&1&0&0&0 \\ 1&0&0&0&0&0 \\ 0&0&0&1&0&0 \\ 0&0&0&0&1&0 \\ 0&0&0&0&0&1 \end{pmatrix}$$

a1' = a3
a2' = a1
a3' = a2

a1 = a2'
a2 = a3'
a3 = a1'

a1*' = a3*
a2*' = a1*
a3*' = a2*

a1* = a2*'
a2* = a3*'
a3* = a1*'

q1' = q1 = (0,b,g)
q2' = q2 = (0,-b,g)

q1 = q1' = (b,g,0)
q2 = q2' = (-b,g,0)
