# Supplementary material for: Equivalence of superspace groups
Source: Acta Crystallogr A. 2012 Nov 14;69(Pt 1):75–90. doi: 10.1107/S0108767312041657 (PMC3553647; doi:10.1107/S0108767312041657)
Supplement: Supplementary file 1 [file a-69-00075-sup1.zip › ssg2d_p21c_abg_ttftcnq.pdf]

14.2.16.6 $P2_1/a(a,b,g)00(-a,-b,g)00$

Superspace group: 14.2.16.6 P2_1/a(a,b,g)00(-a,-b,g)00 [Y:2.113]
Bravais class: 2.16 P2/m(a,b,g)(-a,-b,g)   [JJdW:2.16]
Transformation to supercentered setting: A1=a1, A2=a2, A3=a3, A4=a4-a5, A5=a4+a5

BASIC SPACE GROUP SETTING
Modulation vectors: q1=(a,b,g), q2=(-a,-b,g)
Centering: (0,0,0,0,0)
Non-lattice generators: (-x+1/2,-y,z+1/2,u,t); (x+1/2,y,-z+1/2,-u,-t)
Non-lattice operators: (x,y,z,t,u); (-x+1/2,-y,z+1/2,u,t); (-x,-y,-z,-t,-u);
(x+1/2,y,-z+1/2,-u,-t)

SUPERCENTERED SETTING
Modulation vectors: Q1=(A,B,0), Q2=(0,0,G), where A=a, B=b, G=g
Centering: (0,0,0,0,0); (0,0,0,1/2,1/2)
Non-lattice generators: (-X+1/2,-Y,Z+1/2,-T,U); (X+1/2,Y,-Z+1/2,T,-U)
Non-lattice operators: (X,Y,Z,T,U); (-X+1/2,-Y,Z+1/2,-T,U); (-X,-Y,-Z,-T,-U);
(X+1/2,Y,-Z+1/2,T,-U)
Reflection conditions: HKLMN:M+N=2n; HK0M0:H=2n; 00L0N:L=2n

This is the symmetry for TTF TCNQ.

Published setting in case of 2D modulation is $P2_1/c(a,b,g)00(-a,b,-g)00$

Published setting in case of 1D modulation is 14.1.6.3 $P2_1/c(1/2,b,0)00$

findssg $P2_1/a(a,b,g)00(-a,-b,g)00$

Operators of the standard BSG setting have been entered into 'findssg'.
The resulting S-matrix is the identity, as it should be.

Input setting

Centering
none
Operators
(-x+1/2,-y,z+1/2,u,t); (x+1/2,y,-z+1/2,-u,-t); (x,y,z,t,u); (-x,-y,-z,-t,-u)

Standard settings

Superspace group: 14.2.16.6 P2_1/a(a,b,g)00(-a,-b,g)00 [Y:2.113]
Bravais class: 2.16 P2/m(a,b,g)(-a,-b,g) [JJdW:2.16]
Transformation to supercentered setting: A1=a1, A2=a2, A3=a3, A4=a4-a5, A5=a4+a5

BASIC SPACE GROUP SETTING
Modulation vectors: q1'=(a,b,g), q2'=(-a,-b,g)
Centering: (0,0,0,0,0)
Non-lattice generators: (-x+1/2,-y,z+1/2,u,t); (x+1/2,y,-z+1/2,-u,-t)
Non-lattice operators: (x,y,z,t,u); (-x+1/2,-y,z+1/2,u,t); (-x,-y,-z,-t,-u); (x+1/2,y,-z+1/2,-u,-t)

SUPERCENTERED SETTING
Modulation vectors: Q1'=(A,B,0), Q2'=(0,0,G), where A=a, B=b, G=g
Centering: (0,0,0,0,0); (0,0,0,1/2,1/2)
Non-lattice generators: (-X+1/2,-Y,Z+1/2,-T,U); (X+1/2,Y,-Z+1/2,T,-U)
Non-lattice operators: (X,Y,Z,T,U); (-X+1/2,-Y,Z+1/2,-T,U); (-X,-Y,-Z,-T,-U); (X+1/2,Y,-Z+1/2,T,-U)
Reflection conditions: HKLMN:M+N=2n; HK0M0:H=2n; 00L0N:L=2n

Affine transformation to standard basic space group setting

$S * g(input) * S^{-1} = g(standard)$,
where g is an augmented matrix for an operation in the superspace group.
Also, S * r(input) = r(standard),
where r is an augmented position vector, (x,y,z,t,u,1).

$$S = \begin{pmatrix} 1&0&0&0&0&0\\ 0&1&0&0&0&0\\ 0&0&1&0&0&0\\ 0&0&0&1&0&0\\ 0&0&0&0&1&0\\ 0&0&0&0&0&1 \end{pmatrix} \quad S^{-1} = \begin{pmatrix} 1&0&0&0&0&0\\ 0&1&0&0&0&0\\ 0&0&1&0&0&0\\ 0&0&0&1&0&0\\ 0&0&0&0&1&0\\ 0&0&0&0&0&1 \end{pmatrix}$$

a1' = a1	a1 = a1'	a1*' = a1*	a1* = a1*'	q1' = q1 = (a,b,g)
a2' = a2	a2 = a2'	a2*' = a2*	a2* = a2*'	q2' = q2 = (-a,-b,g)
a3' = a3	a3 = a3'	a3*' = a3*	a3* = a3*'	q1 = q1' = (a,b,g)
				q2 = q2' = (-a,-b,g)

findssg X2_1/a(A,B,0)00(0,0,G)00

X = (0,0,0,1/2,1/2)
Operators of the supercentered setting have been entered into 'findssg'.
The resulting S-matrix gives the transformation to the BSG setting.

Input setting

Centering
(0,0,0,0,0); (0,0,0,1/2,1/2)
Operators
(-x+1/2,-y,z+1/2,-t,u); (x+1/2,y,-z+1/2,t,-u); (x,y,z,t,u); (-x,-y,-z,-t,-u)

Standard settings

Superspace group: 14.2.16.6 P2_1/a(a,b,g)00(-a,-b,g)00 [Y:2.113]
Bravais class: 2.16 P2/m(a,b,g)(-a,-b,g) [JJdW:2.16]
Transformation to supercentered setting: A1=a1, A2=a2, A3=a3, A4=a4-a5, A5=a4+a5

BASIC SPACE GROUP SETTING
Modulation vectors: q1'=(a,b,g), q2'=(-a,-b,g)
Centering: (0,0,0,0,0)
Non-lattice generators: (-x+1/2,-y,z+1/2,u,t); (x+1/2,y,-z+1/2,-u,-t)
Non-lattice operators: (x,y,z,t,u); (-x+1/2,-y,z+1/2,u,t); (-x,-y,-z,-t,-u); (x+1/2,y,-z+1/2,-u,-t)

SUPERCENTERED SETTING
Modulation vectors: Q1'=(A,B,0), Q2'=(0,0,G), where A=a, B=b, G=g
Centering: (0,0,0,0,0); (0,0,0,1/2,1/2)
Non-lattice generators: (-X+1/2,-Y,Z+1/2,-T,U); (X+1/2,Y,-Z+1/2,T,-U)
Non-lattice operators: (X,Y,Z,T,U); (-X+1/2,-Y,Z+1/2,-T,U); (-X,-Y,-Z,-T,-U); (X+1/2,Y,-Z+1/2,T,-U)
Reflection conditions: HKLMN:M+N=2n; HK0M0:H=2n; 00L0N:L=2n

Affine transformation to standard basic space group setting

S * g(input) * S -1 = g(standard),
where g is an augmented matrix for an operation in the superspace group.
Also, S * r(input) = r(standard),
where r is an augmented position vector, (x,y,z,t,u,1).

$$S = \begin{pmatrix} 1&0&0&0&0&0\\ 0&1&0&0&0&0\\ 0&0&1&0&0&0\\ 0&0&0&-1&1&0\\ 0&0&0&1&1&0\\ 0&0&0&0&0&1 \end{pmatrix} \quad S^{-1} = \begin{pmatrix} 1&0&0&0&0&0\\ 0&1&0&0&0&0\\ 0&0&1&0&0&0\\ 0&0&0&-1/2&1/2&0\\ 0&0&0&1/2&1/2&0\\ 0&0&0&0&0&1 \end{pmatrix}$$

a1' = a1

a2' = a2
a3' = a3

a1 = a1'
a2 = a2'
a3 = a3'

a1*' = a1*
a2*' = a2*
a3*' = a3*

a1* = a1*'
a2* = a2*'
a3* = a3*'

q1' = -q1 + q2 = (a,b,g)
q2' = q1 + q2 = (-a,-b,g)

q1 = -1/2 q1' + 1/2 q2' = (-a,-b,0)
q2 = 1/2 q1' + 1/2 q2' = (0,0,g)

findssg $P2_1/c(a,b,g)00(-a,b,-g)00$

Input is published setting for TTF-TCNQ with b unique.
The S-matrix is a cyclic interchange of the axes a, b, c.

Input setting

Centering
none
Operators
(-x,y+1/2,-z+1/2,u,t); (-x,-y,-z,-t,-u); (x,y,z,t,u); (x,-y+1/2,z+1/2,-u,-t)

Standard settings

Superspace group: 14.2.16.6 P2_1/a(a,b,g)00(-a,-b,g)00 [Y:2.113]
Bravais class: 2.16 P2/m(a,b,g)(-a,-b,g) [JJdW:2.16]
Transformation to supercentered setting: A1=a1, A2=a2, A3=a3, A4=a4-a5, A5=a4+a5

BASIC SPACE GROUP SETTING
Modulation vectors: q1'=(a,b,g), q2'=(-a,-b,g)
Centering: (0,0,0,0,0)
Non-lattice generators: (-x+1/2,-y,z+1/2,u,t); (x+1/2,y,-z+1/2,-u,-t)
Non-lattice operators: (x,y,z,t,u); (-x+1/2,-y,z+1/2,u,t); (-x,-y,-z,-t,-u); (x+1/2,y,-z+1/2,-u,-t)

SUPERCENTERED SETTING
Modulation vectors: Q1'=(A,B,0), Q2'=(0,0,G), where A=a, B=b, G=g
Centering: (0,0,0,0,0); (0,0,0,1/2,1/2)
Non-lattice generators: (-X+1/2,-Y,Z+1/2,-T,U); (X+1/2,Y,-Z+1/2,T,-U)
Non-lattice operators: (X,Y,Z,T,U); (-X+1/2,-Y,Z+1/2,-T,U); (-X,-Y,-Z,-T,-U); (X+1/2,Y,-Z+1/2,T,-U)
Reflection conditions: HKLMN:M+N=2n; HK0M0:H=2n; 00L0N:L=2n

Affine transformation to standard basic space group setting

S * g(input) * S $^{-1}$ = g(standard),
where g is an augmented matrix for an operation in the superspace group.
Also, S * r(input) = r(standard),
where r is an augmented position vector, (x,y,z,t,u,1).

$$S = \begin{pmatrix} 0&0&1&0&0&0\\ 1&0&0&0&0&0\\ 0&1&0&0&0&0\\ 0&0&0&1&0&0\\ 0&0&0&0&1&0\\ 0&0&0&0&0&1 \end{pmatrix} \quad S^{-1} = \begin{pmatrix} 0&1&0&0&0&0\\ 0&0&1&0&0&0\\ 1&0&0&0&0&0\\ 0&0&0&1&0&0\\ 0&0&0&0&1&0\\ 0&0&0&0&0&1 \end{pmatrix}$$

a1' = a3	a1 = a2'	a1*' = a3*	a1* = a2*'	q1' = q1 = (a,b,g)
a2' = a1	a2 = a3'	a2*' = a1*	a2* = a3*'	q2' = q2 = (-a,-b,g)
a3' = a2	a3 = a1'	a3*' = a2*	a3* = a1*'	q1 = q1' = (b,g,a)
				q2 = q2' = (-b,g,-a)

findssg $X2_1/c(a,0,g)00(0,b,0)00$

Input is the nonstandard supercentered setting corresponding to the published nonstandard BSG setting. The S matrix is a cyclic interchange of axes a1,a2,a3, as well as a rearrangement of modulation wave vectors.

Input setting

Centering
(0,0,0,0,0); (0,0,0,1/2,1/2)
Operators
(-x,y+1/2,-z+1/2,-t,u); (-x,-y,-z,-t,-u); (x,y,z,t,u); (x,-y+1/2,z+1/2,t,-u)

Standard settings

Superspace group: 14.2.16.6 P2_1/a(a,b,g)00(-a,-b,g)00 [Y:2.113]
Bravais class: 2.16 P2/m(a,b,g)(-a,-b,g) [JJdW:2.16]
Transformation to supercentered setting: A1=a1, A2=a2, A3=a3, A4=a4-a5, A5=a4+a5

BASIC SPACE GROUP SETTING
Modulation vectors: q1'=(a,b,g), q2'=(-a,-b,g)
Centering: (0,0,0,0,0)
Non-lattice generators: (-x+1/2,-y,z+1/2,u,t); (x+1/2,y,-z+1/2,-u,-t)
Non-lattice operators: (x,y,z,t,u); (-x+1/2,-y,z+1/2,u,t); (-x,-y,-z,-t,-u); (x+1/2,y,-z+1/2,-u,-t)

SUPERCENTERED SETTING
Modulation vectors: Q1'=(A,B,0), Q2'=(0,0,G), where A=a, B=b, G=g
Centering: (0,0,0,0,0); (0,0,0,1/2,1/2)
Non-lattice generators: (-X+1/2,-Y,Z+1/2,-T,U); (X+1/2,Y,-Z+1/2,T,-U)
Non-lattice operators: (X,Y,Z,T,U); (-X+1/2,-Y,Z+1/2,-T,U); (-X,-Y,-Z,-T,-U); (X+1/2,Y,-Z+1/2,T,-U)
Reflection conditions: HKLMN:M+N=2n; HK0M0:H=2n; 00L0N:L=2n

Affine transformation to standard basic space group setting

$S * g(\text{input}) * S^{-1} = g(\text{standard})$,
where g is an augmented matrix for an operation in the superspace group.
Also, $S * r(\text{input}) = r(\text{standard})$,
where r is an augmented position vector, (x,y,z,t,u,1).

$$S = \begin{pmatrix} 0 & 0 & 1 & 0 & 0 & 0 \\ 1 & 0 & 0 & 0 & 0 & 0 \\ 0 & 1 & 0 & 0 & 0 & 0 \\ 0 & 0 & 0 & -1 & 1 & 0 \\ 0 & 0 & 0 & 1 & 1 & 0 \\ 0 & 0 & 0 & 0 & 0 & 1 \end{pmatrix} \quad S^{-1} = \begin{pmatrix} 0 & 1 & 0 & 0 & 0 & 0 \\ 0 & 0 & 1 & 0 & 0 & 0 \\ 1 & 0 & 0 & 0 & 0 & 0 \\ 0 & 0 & 0 & -1/2 & 1/2 & 0 \\ 0 & 0 & 0 & 1/2 & 1/2 & 0 \\ 0 & 0 & 0 & 0 & 0 & 1 \end{pmatrix}$$

a1' = a3
a2' = a1
a3' = a2

a1 = a2'
a2 = a3'
a3 = a1'

a1*' = a3*
a2*' = a1*
a3*' = a2*

a1* = a2*'
a2* = a3*'
a3* = a1*'

q1' = -q1 + q2 = (a,b,g)
q2' = q1 + q2 = (-a,-b,g)

q1 = -1/2 q1' + 1/2 q2' = (-b,0,-a)
q2 = 1/2 q1' + 1/2 q2' = (0,g,0)

14.1.6.3 $P2_1/b(1/2,0,g)00$

Superspace group: 14.1.6.3 P2_1/b(1/2,0,g)00 [Y:1.39]
Bravais class: 1.6 P2/m(1/2,0,g)   [JJdW:1.6]
Transformation to supercentered setting: A1=2a1+a4, A2=a2, A3=a3, A4=a4

BASIC SPACE GROUP SETTING
Modulation vectors: q1=(1/2,0,g)
Centering: (0,0,0,0)
Non-lattice generators: (-x,-y+1/2,z+1/2,-x+t); (x,y+1/2,-z+1/2,x-t)
Non-lattice operators: (x,y,z,t); (-x,-y+1/2,z+1/2,-x+t); (-x,-y,-z,-t); (x,y+1/2,-z+1/2,x-t)

SUPERCENTERED SETTING
Modulation vectors: Q1=(0,0,G), where G=g
Centering: (0,0,0,0); (1/2,0,0,1/2)
Non-lattice generators: (-X,-Y+1/2,Z+1/2,T); (X,Y+1/2,-Z+1/2,-T)
Non-lattice operators: (X,Y,Z,T); (-X,-Y+1/2,Z+1/2,T); (-X,-Y,-Z,-T); (X,Y+1/2,-Z+1/2,-T)
Reflection conditions: HKLM:H+M=2n; HK00:K=2n; 00LM:L=2n

This is the symmetry of the 1D modulated phase of TTF TCNQ.

findssg P2$_1$/b(1/2,0,g)00

Operators of the standard BSG setting have been entered into 'findssg'.
The resulting S-matrix is the identity, as it should be.

Input setting

Centering
none
Operators
(-x,-y+1/2,z+1/2,-x+t); (-x,-y,-z,-t); (x,y,z,t); (x,y+1/2,-z+1/2,x-t)

Standard settings

Superspace group: 14.1.6.3 P2_1/b(1/2,0,g)00 [Y:1.39]
Bravais class: 1.6 P2/m(1/2,0,g) [JJdW:1.6]
Transformation to supercentered setting: A1=2a1+a4, A2=a2, A3=a3, A4=a4

BASIC SPACE GROUP SETTING
Modulation vectors: q1'=(1/2,0,g)
Centering: (0,0,0,0)
Non-lattice generators: (-x,-y+1/2,z+1/2,-x+t); (x,y+1/2,-z+1/2,x-t)
Non-lattice operators: (x,y,z,t); (-x,-y+1/2,z+1/2,-x+t); (-x,-y,-z,-t); (x,y+1/2,-z+1/2,x-t)

SUPERCENTERED SETTING
Modulation vectors: Q1'=(0,0,G), where G=g
Centering: (0,0,0,0); (1/2,0,0,1/2)
Non-lattice generators: (-X,-Y+1/2,Z+1/2,T); (X,Y+1/2,-Z+1/2,-T)
Non-lattice operators: (X,Y,Z,T); (-X,-Y+1/2,Z+1/2,T); (-X,-Y,-Z,-T); (X,Y+1/2,-Z+1/2,-T)
Reflection conditions: HKLM:H+M=2n; HK00:K=2n; 00LM:L=2n

Affine transformation to standard basic space group setting

S * g(input) * S $^{-1}$ = g(standard),
where g is an augmented matrix for an operation in the superspace group.
Also, S * r(input) = r(standard),
where r is an augmented position vector, (x,y,z,t,1).

$$S = \begin{matrix} 1 & 0 & 0 & 0 & 0 \\ 0 & 1 & 0 & 0 & 0 \\ 0 & 0 & 1 & 0 & 0 \\ 0 & 0 & 0 & 1 & 0 \\ 0 & 0 & 0 & 0 & 1 \end{matrix} \quad S^{-1} = \begin{matrix} 1 & 0 & 0 & 0 & 0 \\ 0 & 1 & 0 & 0 & 0 \\ 0 & 0 & 1 & 0 & 0 \\ 0 & 0 & 0 & 1 & 0 \\ 0 & 0 & 0 & 0 & 1 \end{matrix}$$

a1' = a1	a1 = a1'	a1*' = a1*	a1* = a1*'	q1' = q1 = (1/2,0,g)
a2' = a2	a2 = a2'	a2*' = a2*	a2* = a2*'	
a3' = a3	a3 = a3'	a3*' = a3*	a3* = a3*'	q1 = q1' = (1/2,0,g)

findssg $P_a2_1/b(0,0,g)00$

Operators of the supercentered setting have been entered into 'findssg'.
The resulting S-matrix reflects the transformation a1=(A1-A4)/2.

Input setting

Centering
(0,0,0,0); (1/2,0,0,1/2)
Operators
(-x,-y+1/2,z+1/2,t); (-x,-y,-z,-t); (x,y,z,t); (x,y+1/2,-z+1/2,-t)

Standard settings

Superspace group: 14.1.6.3 P2_1/b(1/2,0,g)00 [Y:1.39]
Bravais class: 1.6 P2/m(1/2,0,g) [JJdW:1.6]
Transformation to supercentered setting: A1=2a1+a4, A2=a2, A3=a3, A4=a4

BASIC SPACE GROUP SETTING
Modulation vectors: q1'=(1/2,0,g)
Centering: (0,0,0,0)
Non-lattice generators: (-x,-y+1/2,z+1/2,-x+t); (x,y+1/2,-z+1/2,x-t)
Non-lattice operators: (x,y,z,t); (-x,-y+1/2,z+1/2,-x+t); (-x,-y,-z,-t); (x,y+1/2,-z+1/2,x-t)

SUPERCENTERED SETTING
Modulation vectors: Q1'=(0,0,G), where G=g
Centering: (0,0,0,0); (1/2,0,0,1/2)
Non-lattice generators: (-X,-Y+1/2,Z+1/2,T); (X,Y+1/2,-Z+1/2,-T)
Non-lattice operators: (X,Y,Z,T); (-X,-Y+1/2,Z+1/2,T); (-X,-Y,-Z,-T); (X,Y+1/2,-Z+1/2,-T)
Reflection conditions: HKLM:H+M=2n; HK00:K=2n; 00LM:L=2n

Affine transformation to standard basic space group setting

$S * g(\text{input}) * S^{-1} = g(\text{standard})$,
where g is an augmented matrix for an operation in the superspace group.
Also, $S * r(\text{input}) = r(\text{standard})$,
where r is an augmented position vector, (x,y,z,t,1).

$$S = \begin{pmatrix} 2 & 0 & 0 & 0 & 0 \\ 0 & 1 & 0 & 0 & 0 \\ 0 & 0 & 1 & 0 & 0 \\ 1 & 0 & 0 & 1 & 0 \\ 0 & 0 & 0 & 0 & 1 \end{pmatrix} \quad S^{-1} = \begin{pmatrix} 1/2 & 0 & 0 & 0 & 0 \\ 0 & 1 & 0 & 0 & 0 \\ 0 & 0 & 1 & 0 & 0 \\ -1/2 & 0 & 0 & 1 & 0 \\ 0 & 0 & 0 & 0 & 1 \end{pmatrix}$$

a1' = 1/2 a1	a2 = a2'	a3*' = a3*	
a2' = a2	a3 = a3'		q1' = q1 + a1* = (1/2,0,g)
a3' = a3		a1* = 1/2 a1*'	
	a1*' = 2 a1*	a2* = a2*'	q1 = q1' - 1/2 a1*' = (0,0,g)
a1 = 2 a1'	a2*' = a2*	a3* = a3*'	

findssg P2$_1$/c(1/2,b,0)00

Published BSG setting for TTF-TCNQ

Input setting

Centering
none
Operators
(-x,y+1/2,-z+1/2,-x+t); (-x,-y,-z,-t); (x,y,z,t); (x,-y+1/2,z+1/2,x-t)

Standard settings

Superspace group: 14.1.6.3 P2_1/b(1/2,0,g)00 [Y:1.39]
Bravais class: 1.6 P2/m(1/2,0,g) [JJdW:1.6]
Transformation to supercentered setting: A1=2a1+a4, A2=a2, A3=a3, A4=a4

BASIC SPACE GROUP SETTING
Modulation vectors: q1'=(1/2,0,g)
Centering: (0,0,0,0)
Non-lattice generators: (-x,-y+1/2,z+1/2,-x+t); (x,y+1/2,-z+1/2,x-t)
Non-lattice operators: (x,y,z,t); (-x,-y+1/2,z+1/2,-x+t); (-x,-y,-z,-t); (x,y+1/2,-z+1/2,x-t)

SUPERCENTERED SETTING
Modulation vectors: Q1'=(0,0,G), where G=g
Centering: (0,0,0,0); (1/2,0,0,1/2)
Non-lattice generators: (-X,-Y+1/2,Z+1/2,T); (X,Y+1/2,-Z+1/2,-T)
Non-lattice operators: (X,Y,Z,T); (-X,-Y+1/2,Z+1/2,T); (-X,-Y,-Z,-T); (X,Y+1/2,-Z+1/2,-T)
Reflection conditions: HKLM:H+M=2n; HK00:K=2n; 00LM:L=2n

Affine transformation to standard basic space group setting

S * g(input) * S $^{-1}$ = g(standard),
where g is an augmented matrix for an operation in the superspace group.
Also, S * r(input) = r(standard),
where r is an augmented position vector, (x,y,z,t,1).

$$S = \begin{pmatrix} 1 & 0 & 0 & 0 & 0 \\ 0 & 0 & -1 & 0 & 0 \\ 0 & 1 & 0 & 0 & 0 \\ 0 & 0 & 0 & 1 & 0 \\ 0 & 0 & 0 & 0 & 1 \end{pmatrix} \quad S^{-1} = \begin{pmatrix} 1 & 0 & 0 & 0 & 0 \\ 0 & 0 & 1 & 0 & 0 \\ 0 & -1 & 0 & 0 & 0 \\ 0 & 0 & 0 & 1 & 0 \\ 0 & 0 & 0 & 0 & 1 \end{pmatrix}$$

a1' = a1	a1 = a1'	a1*' = a1*	a1* = a1*'	q1' = q1 = (1/2,0,g)
a2' = -a3	a2 = a3'	a2*' = -a3*	a2* = a3*'	
a3' = a2	a3 = -a2'	a3*' = a2*	a3* = -a2*'	q1 = q1' = (1/2,g,0)

findssg $P_a2_1/c(0,b,0)00$

Non-standard supercentered setting corresponding to the published BSG setting for TTF-TCNQ has been entered into findssg.

Input setting

Centering
(0,0,0,0); (1/2,0,0,1/2)
Operators
(-x,y+1/2,-z+1/2,t); (-x,-y,-z,-t); (x,y,z,t); (x,-y+1/2,z+1/2,-t)

Standard settings

Superspace group: 14.1.6.3 P2_1/b(1/2,0,g)00 [Y:1.39]
Bravais class: 1.6 P2/m(1/2,0,g) [JJdW:1.6]
Transformation to supercentered setting: A1=2a1+a4, A2=a2, A3=a3, A4=a4

BASIC SPACE GROUP SETTING
Modulation vectors: q1'=(1/2,0,g)
Centering: (0,0,0,0)
Non-lattice generators: (-x,-y+1/2,z+1/2,-x+t); (x,y+1/2,-z+1/2,x-t)
Non-lattice operators: (x,y,z,t); (-x,-y+1/2,z+1/2,-x+t); (-x,-y,-z,-t); (x,y+1/2,-z+1/2,x-t)

SUPERCENTERED SETTING
Modulation vectors: Q1'=(0,0,G), where G=g
Centering: (0,0,0,0); (1/2,0,0,1/2)
Non-lattice generators: (-X,-Y+1/2,Z+1/2,T); (X,Y+1/2,-Z+1/2,-T)
Non-lattice operators: (X,Y,Z,T); (-X,-Y+1/2,Z+1/2,T); (-X,-Y,-Z,-T); (X,Y+1/2,-Z+1/2,-T)
Reflection conditions: HKLM:H+M=2n; HK00:K=2n; 00LM:L=2n

Affine transformation to standard basic space group setting

$S * g(\text{input}) * S^{-1} = g(\text{standard})$,
where g is an augmented matrix for an operation in the superspace group.
Also, $S * r(\text{input}) = r(\text{standard})$,
where r is an augmented position vector, (x,y,z,t,1).

$$S = \begin{matrix} 2 & 0 & 0 & 0 & 0 \\ 0 & 0 & -1 & 0 & 0 \\ 0 & 1 & 0 & 0 & 0 \\ 1 & 0 & 0 & 1 & 0 \\ 0 & 0 & 0 & 0 & 1 \end{matrix} \qquad S^{-1} = \begin{matrix} 1/2 & 0 & 0 & 0 & 0 \\ 0 & 0 & 1 & 0 & 0 \\ 0 & -1 & 0 & 0 & 0 \\ -1/2 & 0 & 0 & 1 & 0 \\ 0 & 0 & 0 & 0 & 1 \end{matrix}$$

a1' = 1/2 a1	a1 = 2 a1'	a1*' = 2 a1*	a1* = 1/2 a1*'	q1' = q1 + a1* = (1/2,0,g)
a2' = -a3	a2 = a3'	a2*' = -a3*	a2* = a3*'	q1 = q1' - 1/2 a1*' = (0,g,0)
a3' = a2	a3 = -a2'	a3*' = a2*	a3* = -a2*'	
