# Supplementary material for: Equivalence of superspace groups
Source: Acta Crystallogr A. 2012 Nov 14;69(Pt 1):75–90. doi: 10.1107/S0108767312041657 (PMC3553647; doi:10.1107/S0108767312041657)
Supplement: Supplementary file 1 [file a-69-00075-sup1.zip › ssg2d_p21m_nbse3.pdf]

11.2.6.4 $P2_1/m(1/2,0,g1)00(0,0,g2)00$

Superspace group: 11.2.6.4 P2_1/m(1/2,0,g1)00(0,0,g2)00 [Y:2.73]
Bravais class: 2.6 P2/m(1/2,0,g1)(0,0,g2) [JJdW:2.6]
Transformation to supercentered setting: A1=2a1+a4, A2=a2, A3=a3, A4=a4, A5=a5

BASIC SPACE GROUP SETTING
Modulation vectors: q1=(1/2,0,g1), q2=(0,0,g2)
Centering: (0,0,0,0,0)
Non-lattice generators: (-x,-y,z+1/2,-x+t,u); (x,y,-z+1/2,x-t,-u)
Non-lattice operators: (x,y,z,t,u); (-x,-y,z+1/2,-x+t,u); (-x,-y,-z,-t,-u); (x,y,-z+1/2,x-t,-u)

SUPERCENTERED SETTING
Modulation vectors: Q1=(0,0,G1), Q2=(0,0,G2), where G1=g1, G2=g2
Centering: (0,0,0,0,0); (1/2,0,0,1/2,0)
Non-lattice generators: (-X,-Y,Z+1/2,T,U); (X,Y,-Z+1/2,-T,-U)
Non-lattice operators: (X,Y,Z,T,U); (-X,-Y,Z+1/2,T,U); (-X,-Y,-Z,-T,-U); (X,Y,-Z+1/2,-T,-U)
Reflection conditions: HKLMN:H+M=2n; 00LMN:L=2n

This is the superspace group for the 2D modulated CDW phase of $NbSe_3$.

The published setting is $P2_1/m(0,b2,0)s0$ (1/2,b1,1/2)00.

findssg $X2_1/m(0,0,g1)00(0,0,g2)00$

X = (1/2,0,0,1/2,0)
Notice that operators of the standard supercentered setting are entered into findssg, thus S is the transformation from the standard supercentered to the standard BSG settings.

Input setting

Centering
(0,0,0,0,0); (1/2,0,0,1/2,0)
Operators
(-x,-y,z+1/2,t,u); (-x,-y,-z,-t,-u); (x,y,z,t,u); (x,y,-z+1/2,-t,-u)

Standard settings

Superspace group: 11.2.6.4 P2_1/m(1/2,0,g1)00(0,0,g2)00 [Y:2.73]
Bravais class: 2.6 P2/m(1/2,0,g1)(0,0,g2) [JJdW:2.6]
Transformation to supercentered setting: A1=2a1+a4, A2=a2, A3=a3, A4=a4, A5=a5

BASIC SPACE GROUP SETTING
Modulation vectors: q1'=(1/2,0,g1), q2'=(0,0,g2)
Centering: (0,0,0,0,0)
Non-lattice generators: (-x,-y,z+1/2,-x+t,u); (x,y,-z+1/2,x-t,-u)
Non-lattice operators: (x,y,z,t,u); (-x,-y,z+1/2,-x+t,u); (-x,-y,-z,-t,-u); (x,y,-z+1/2,x-t,-u)

SUPERCENTERED SETTING
Modulation vectors: Q1'=(0,0,G1), Q2'=(0,0,G2), where G1=g1, G2=g2
Centering: (0,0,0,0,0); (1/2,0,0,1/2,0)
Non-lattice generators: (-X,-Y,Z+1/2,T,U); (X,Y,-Z+1/2,-T,-U)
Non-lattice operators: (X,Y,Z,T,U); (-X,-Y,Z+1/2,T,U); (-X,-Y,-Z,-T,-U); (X,Y,-Z+1/2,-T,-U)
Reflection conditions: HKLMN:H+M=2n; 00LMN:L=2n

Affine transformation to standard basic space group setting

$S * g(input) * S^{-1} = g(standard)$,
where g is an augmented matrix for an operation in the superspace group.
Also, S * r(input) = r(standard),
where r is an augmented position vector, (x,y,z,t,u,1).

$$S = \begin{pmatrix} 2 & 0 & 0 & 0 & 0 & 0 \\ 0 & 1 & 0 & 0 & 0 & 0 \\ 0 & 0 & 1 & 0 & 0 & 0 \\ 1 & 0 & 0 & 1 & 0 & 0 \\ 0 & 0 & 0 & 0 & 1 & 0 \\ 0 & 0 & 0 & 0 & 0 & 1 \end{pmatrix} \quad S^{-1} = \begin{pmatrix} 1/2 & 0 & 0 & 0 & 0 & 0 \\ 0 & 1 & 0 & 0 & 0 & 0 \\ 0 & 0 & 1 & 0 & 0 & 0 \\ -1/2 & 0 & 0 & 1 & 0 & 0 \\ 0 & 0 & 0 & 0 & 1 & 0 \\ 0 & 0 & 0 & 0 & 0 & 1 \end{pmatrix}$$

$a1' = 1/2\ a1$
$a2' = a2$
$a3' = a3$

$a1 = 2\ a1'$
$a2 = a2'$
$a3 = a3'$

$a1^{*\prime} = 2\ a1^*$
$a2^{*\prime} = a2^*$
$a3^{*\prime} = a3^*$

$a1^* = 1/2\ a1^{*\prime}$
$a2^* = a2^{*\prime}$
$a3^* = a3^{*\prime}$

$q1' = q1 + a1^* = (1/2,0,g1)$
$q2' = q2 = (0,0,g2)$

$q1 = q1' - 1/2\ a1^{*\prime} = (0,0,g1)$
$q2 = q2' = (0,0,g2)$

findssg P2$_1$/m(1/2,0,g1)00(0,0,g2)00

Notice operators of standard BSG setting are entered into findssg, thus S is the identity matrix.

Input setting

Centering
none
Operators
(-x,-y,z+1/2,-x+t,u); (x,y,-z+1/2,x-t,-u); (x,y,z,t,u); (-x,-y,-z,-t,-u)

Standard settings

Superspace group: 11.2.6.4 P2_1/m(1/2,0,g1)00(0,0,g2)00 [Y:2.73]
Bravais class: 2.6 P2/m(1/2,0,g1)(0,0,g2) [JJdW:2.6]
Transformation to supercentered setting: A1=2a1+a4, A2=a2, A3=a3, A4=a4, A5=a5

BASIC SPACE GROUP SETTING
Modulation vectors: q1'=(1/2,0,g1), q2'=(0,0,g2)
Centering: (0,0,0,0,0)
Non-lattice generators: (-x,-y,z+1/2,-x+t,u); (x,y,-z+1/2,x-t,-u)
Non-lattice operators: (x,y,z,t,u); (-x,-y,z+1/2,-x+t,u); (-x,-y,-z,-t,-u); (x,y,-z+1/2,x-t,-u)

SUPERCENTERED SETTING
Modulation vectors: Q1'=(0,0,G1), Q2'=(0,0,G2), where G1=g1, G2=g2
Centering: (0,0,0,0,0); (1/2,0,0,1/2,0)
Non-lattice generators: (-X,-Y,Z+1/2,T,U); (X,Y,-Z+1/2,-T,-U)
Non-lattice operators: (X,Y,Z,T,U); (-X,-Y,Z+1/2,T,U); (-X,-Y,-Z,-T,-U); (X,Y,-Z+1/2,-T,-U)
Reflection conditions: HKLMN:H+M=2n; 00LMN:L=2n

Affine transformation to standard basic space group setting

$S * g(input) * S^{-1} = g(standard)$,
where g is an augmented matrix for an operation in the superspace group.
Also, S * r(input) = r(standard),
where r is an augmented position vector, (x,y,z,t,u,1).

$$S = \begin{pmatrix} 1&0&0&0&0&0\\ 0&1&0&0&0&0\\ 0&0&1&0&0&0\\ 0&0&0&1&0&0\\ 0&0&0&0&1&0\\ 0&0&0&0&0&1 \end{pmatrix} \quad S^{-1} = \begin{pmatrix} 1&0&0&0&0&0\\ 0&1&0&0&0&0\\ 0&0&1&0&0&0\\ 0&0&0&1&0&0\\ 0&0&0&0&1&0\\ 0&0&0&0&0&1 \end{pmatrix}$$

a1' = a1
a2' = a2
a3' = a3

a1 = a1'
a2 = a2'
a3 = a3'

a1*' = a1*
a2*' = a2*
a3*' = a3*

a1* = a1*'
a2* = a2*'
a3* = a3*'

q1' = q1 = (1/2,0,g1)
q2' = q2 = (0,0,g2)

q1 = q1' = (1/2,0,g1)
q2 = q2' = (0,0,g2)

findssg $P2_1/m(1/2,0,g1)s0(0,0,g2)00$

(2_1,s0) entered into findssg; origin shift to standard setting.
The origin shift is (1/2,0,0,0,0) in the BSC setting, which amounts to (1/4,0,0,-1/4,0) in the supercentered setting.

Input setting

Centering
none
Operators
(-x,-y,z+1/2,-x+t+1/2,u); (-x,-y,-z,-t,-u); (x,y,z,t,u); (x,y,-z+1/2,x-t+1/2,-u)

Standard settings

Superspace group: 11.2.6.4 P2_1/m(1/2,0,g1)00(0,0,g2)00 [Y:2.73]
Bravais class: 2.6 P2/m(1/2,0,g1)(0,0,g2) [JJdW:2.6]
Transformation to supercentered setting: A1=2a1+a4, A2=a2, A3=a3, A4=a4, A5=a5

BASIC SPACE GROUP SETTING
Modulation vectors: q1'=(1/2,0,g1), q2'=(0,0,g2)
Centering: (0,0,0,0,0)
Non-lattice generators: (-x,-y,z+1/2,-x+t,u); (x,y,-z+1/2,x-t,-u)
Non-lattice operators: (x,y,z,t,u); (-x,-y,z+1/2,-x+t,u); (-x,-y,-z,-t,-u); (x,y,-z+1/2,x-t,-u)

SUPERCENTERED SETTING
Modulation vectors: Q1'=(0,0,G1), Q2'=(0,0,G2), where G1=g1, G2=g2
Centering: (0,0,0,0,0); (1/2,0,0,1/2,0)
Non-lattice generators: (-X,-Y,Z+1/2,T,U); (X,Y,-Z+1/2,-T,-U)
Non-lattice operators: (X,Y,Z,T,U); (-X,-Y,Z+1/2,T,U); (-X,-Y,-Z,-T,-U); (X,Y,-Z+1/2,-T,-U)
Reflection conditions: HKLMN:H+M=2n; 00LMN:L=2n

Affine transformation to standard basic space group setting

$S * g(input) * S^{-1} = g(standard)$,
where g is an augmented matrix for an operation in the superspace group.
Also, S * r(input) = r(standard),
where r is an augmented position vector, (x,y,z,t,u,1).

$$S = \begin{pmatrix} 1 & 0 & 0 & 0 & 0 & 1/2 \\ 0 & 1 & 0 & 0 & 0 & 0 \\ 0 & 0 & 1 & 0 & 0 & 0 \\ 0 & 0 & 0 & 1 & 0 & 0 \\ 0 & 0 & 0 & 0 & 1 & 0 \\ 0 & 0 & 0 & 0 & 0 & 1 \end{pmatrix} \quad S^{-1} = \begin{pmatrix} 1 & 0 & 0 & 0 & 0 & -1/2 \\ 0 & 1 & 0 & 0 & 0 & 0 \\ 0 & 0 & 1 & 0 & 0 & 0 \\ 0 & 0 & 0 & 1 & 0 & 0 \\ 0 & 0 & 0 & 0 & 1 & 0 \\ 0 & 0 & 0 & 0 & 0 & 1 \end{pmatrix}$$

a1' = a1
a2' = a2
a3' = a3

a1 = a1'
a2 = a2'
a3 = a3'

a1*' = a1*
a2*' = a2*
a3*' = a3*

a1* = a1*'
a2* = a2*'
a3* = a3*'

q1' = q1 = (1/2,0,g1)
q2' = q2 = (0,0,g2)

q1 = q1' = (1/2,0,g1)
q2 = q2' = (0,0,g2)

findssg $P2_1/m(1/2,0,g1)00(0,0,g2)s0$

(2_1,0s) entered into findssg; q2new = a3* - q2 towards standard setting is desired. Notice that findssg gives as new q2-vector (a3* + q2).

Input setting

Centering
none
Operators
(-x,-y,z+1/2,-x+t,u+1/2); (-x,-y,-z,-t,-u); (x,y,z,t,u); (x,y,-z+1/2,x-t,-u+1/2)

Standard settings

Superspace group: 11.2.6.4 P2_1/m(1/2,0,g1)00(0,0,g2)00 [Y:2.73]
Bravais class: 2.6 P2/m(1/2,0,g1)(0,0,g2)   [JJdW:2.6]
Transformation to supercentered setting: A1=2a1+a4, A2=a2, A3=a3, A4=a4, A5=a5

BASIC SPACE GROUP SETTING
Modulation vectors: q1'=(1/2,0,g1), q2'=(0,0,g2)
Centering: (0,0,0,0,0)
Non-lattice generators: (-x,-y,z+1/2,-x+t,u); (x,y,-z+1/2,x-t,-u)
Non-lattice operators: (x,y,z,t,u); (-x,-y,z+1/2,-x+t,u); (-x,-y,-z,-t,-u); (x,y,-z+1/2,x-t,-u)

SUPERCENTERED SETTING
Modulation vectors: Q1'=(0,0,G1), Q2'=(0,0,G2), where G1=g1, G2=g2
Centering: (0,0,0,0,0); (1/2,0,0,1/2,0)
Non-lattice generators: (-X,-Y,Z+1/2,T,U); (X,Y,-Z+1/2,-T,-U)
Non-lattice operators: (X,Y,Z,T,U); (-X,-Y,Z+1/2,T,U); (-X,-Y,-Z,-T,-U); (X,Y,-Z+1/2,-T,-U)
Reflection conditions: HKLMN:H+M=2n; 00LMN:L=2n

Affine transformation to standard basic space group setting

$S * g(\text{input}) * S^{-1} = g(\text{standard})$,
where g is an augmented matrix for an operation in the superspace group.
Also, S * r(input) = r(standard),
where r is an augmented position vector, (x,y,z,t,u,1).

$$S = \begin{pmatrix} 1&0&0&0&0&0\\ 0&1&0&0&0&0\\ 0&0&1&0&0&0\\ 0&0&0&1&0&0\\ 0&0&1&0&1&0\\ 0&0&0&0&0&1 \end{pmatrix} \quad S^{-1} = \begin{pmatrix} 1&0&0&0&0&0\\ 0&1&0&0&0&0\\ 0&0&1&0&0&0\\ 0&0&0&1&0&0\\ 0&0&-1&0&1&0\\ 0&0&0&0&0&1 \end{pmatrix}$$

a1' = a1
a2' = a2
a3' = a3

a1 = a1'
a2 = a2'
a3 = a3'

a1*' = a1*
a2*' = a2*
a3*' = a3*

a1* = a1*'
a2* = a2*'
a3* = a3*'

q1' = q1 = (1/2,0,g1)
q2' = q2 + a3* = (0,0,g2)

q1 = q1' = (1/2,0,g1)
q2 = q2' - a3*' = (0,0,g2-1)

findssg P2_1/m(1/2,0,g1)s0(0,0,g2)s0

(2_1,ss) entered into findssg; q2new = a3* - q2 as well as origin shift towards standard setting is desired. Notice that findssg gives as new q2-vector (a3* + q2). The origin shift is (1/2,0,0,0,0) in the BSC setting, which amounts to (1/4,0,0,-1/4,0) in the supercentered setting.

Input setting

Centering
none
Operators
(-x,-y,z+1/2,-x+t+1/2,u+1/2); (-x,-y,-z,-t,-u); (x,y,z,t,u); (x,y,-z+1/2,x-t+1/2,-u+1/2)

Standard settings

Superspace group: 11.2.6.4 P2_1/m(1/2,0,g1)00(0,0,g2)00 [Y:2.73]
Bravais class: 2.6 P2/m(1/2,0,g1)(0,0,g2) [JJdW:2.6]
Transformation to supercentered setting: A1=2a1+a4, A2=a2, A3=a3, A4=a4, A5=a5

BASIC SPACE GROUP SETTING
Modulation vectors: q1'=(1/2,0,g1), q2'=(0,0,g2)
Centering: (0,0,0,0,0)
Non-lattice generators: (-x,-y,z+1/2,-x+t,u); (x,y,-z+1/2,x-t,-u)
Non-lattice operators: (x,y,z,t,u); (-x,-y,z+1/2,-x+t,u); (-x,-y,-z,-t,-u); (x,y,-z+1/2,x-t,-u)

SUPERCENTERED SETTING
Modulation vectors: Q1'=(0,0,G1), Q2'=(0,0,G2), where G1=g1, G2=g2
Centering: (0,0,0,0,0); (1/2,0,0,1/2,0)
Non-lattice generators: (-X,-Y,Z+1/2,T,U); (X,Y,-Z+1/2,-T,-U)
Non-lattice operators: (X,Y,Z,T,U); (-X,-Y,Z+1/2,T,U); (-X,-Y,-Z,-T,-U); (X,Y,-Z+1/2,-T,-U)
Reflection conditions: HKLMN:H+M=2n; 00LMN:L=2n

Affine transformation to standard basic space group setting

$S * g(\text{input}) * S^{-1} = g(\text{standard})$,
where g is an augmented matrix for an operation in the superspace group.
Also, S * r(input) = r(standard),
where r is an augmented position vector, (x,y,z,t,u,1).

$$S = \begin{matrix} 1 & 0 & 0 & 0 & 0 & 1/2 \\ 0 & 1 & 0 & 0 & 0 & 0 \\ 0 & 0 & 1 & 0 & 0 & 0 \\ 0 & 0 & 0 & 1 & 0 & 0 \\ 0 & 0 & 1 & 0 & 1 & 0 \\ 0 & 0 & 0 & 0 & 0 & 1 \end{matrix} \quad S^{-1} = \begin{matrix} 1 & 0 & 0 & 0 & 0 & -1/2 \\ 0 & 1 & 0 & 0 & 0 & 0 \\ 0 & 0 & 1 & 0 & 0 & 0 \\ 0 & 0 & 0 & 1 & 0 & 0 \\ 0 & 0 & -1 & 0 & 1 & 0 \\ 0 & 0 & 0 & 0 & 0 & 1 \end{matrix}$$

a1' = a1
a2' = a2
a3' = a3

a1 = a1'
a2 = a2'
a3 = a3'

a1*' = a1*
a2*' = a2*
a3*' = a3*

a1* = a1*'
a2* = a2*'
a3* = a3*'

q1' = q1 = (1/2,0,g1)
q2' = q2 + a3* = (0,0,g2)

q1 = q1' = (1/2,0,g1)
q2 = q2' - a3*' = (0,0,g2-1)

findssg $P2_1/m(1/2,1/2,g1)00(0,0,g2)00$

Alternate first q-vector (1/2, 1/2, g1) requires a diagonal unit cell for the basic structure for transforming to the standard setting.
a1new = a1; a2new=-a1+a2; a3new=a3; a4new=a4; a5new=a5.
a*1new = a*1+a*2; a*2new=a*2; a*3new=a*3; q1new= q1; q2new=q2.

Input setting

Centering
none
Operators
(-x,-y,z+1/2,-x-y+t,u); (x,y,-z+1/2,x+y-t,-u); (x,y,z,t,u); (-x,-y,-z,-t,-u)

Standard settings

Superspace group: 11.2.6.4 P2_1/m(1/2,0,g1)00(0,0,g2)00 [Y:2.73]
Bravais class: 2.6 P2/m(1/2,0,g1)(0,0,g2) [JJdW:2.6]
Transformation to supercentered setting: A1=2a1+a4, A2=a2, A3=a3, A4=a4, A5=a5

BASIC SPACE GROUP SETTING
Modulation vectors: q1'=(1/2,0,g1), q2'=(0,0,g2)
Centering: (0,0,0,0,0)
Non-lattice generators: (-x,-y,z+1/2,-x+t,u); (x,y,-z+1/2,x-t,-u)
Non-lattice operators: (x,y,z,t,u); (-x,-y,z+1/2,-x+t,u); (-x,-y,-z,-t,-u); (x,y,-z+1/2,x-t,-u)

SUPERCENTERED SETTING
Modulation vectors: Q1'=(0,0,G1), Q2'=(0,0,G2), where G1=g1, G2=g2
Centering: (0,0,0,0,0); (1/2,0,0,1/2,0)
Non-lattice generators: (-X,-Y,Z+1/2,T,U); (X,Y,-Z+1/2,-T,-U)
Non-lattice operators: (X,Y,Z,T,U); (-X,-Y,Z+1/2,T,U); (-X,-Y,-Z,-T,-U); (X,Y,-Z+1/2,-T,-U)
Reflection conditions: HKLMN:H+M=2n; 00LMN:L=2n

Affine transformation to standard basic space group setting

$S * g(\text{input}) * S^{-1} = g(\text{standard})$,
where g is an augmented matrix for an operation in the superspace group.
Also, S * r(input) = r(standard),
where r is an augmented position vector, (x,y,z,t,u,1).

$$S = \begin{pmatrix} 1 & 1 & 0 & 0 & 0 & 0 \\ 0 & 1 & 0 & 0 & 0 & 0 \\ 0 & 0 & 1 & 0 & 0 & 0 \\ 0 & 0 & 0 & 1 & 0 & 0 \\ 0 & 0 & 0 & 0 & 1 & 0 \\ 0 & 0 & 0 & 0 & 0 & 1 \end{pmatrix} \quad S^{-1} = \begin{pmatrix} 1 & -1 & 0 & 0 & 0 & 0 \\ 0 & 1 & 0 & 0 & 0 & 0 \\ 0 & 0 & 1 & 0 & 0 & 0 \\ 0 & 0 & 0 & 1 & 0 & 0 \\ 0 & 0 & 0 & 0 & 1 & 0 \\ 0 & 0 & 0 & 0 & 0 & 1 \end{pmatrix}$$

a1' = a1
a2' = -a1 + a2
a3' = a3

a1 = a1'
a2 = a1' + a2'
a3 = a3'

a1*' = a1* + a2*
a2*' = a2*
a3*' = a3*

a1* = a1*' - a2*'
a2* = a2*'
a3* = a3*'

q1' = q1 = (1/2,0,g1)
q2' = q2 = (0,0,g2)

q1 = q1' = (1/2,1/2,g1)
q2 = q2' = (0,0,g2)

Not required anymore:
g(standard)=Talt * g(BSG) * Talt $^{-1}$ => g(BSG) = Talt^{-1} T * g(input) * T^{-1} Talt => Transformation from any setting "input" to the standard BSG setting goes by T_{new} = (Talt^{-1} T) and its inverse (T^{-1} Talt), where Talt is the transformation from standard BSG to supercentered setting.

findssg $P2_1/m(1/2,1/2,g1)00(0,0,g2)s0$

Alternate first q-vector (1/2, 1/2, g1) and superspace operator (2_1,0s).

Input setting

Centering
none
Operators
(-x,-y,z+1/2,-x-y+t,u+1/2); (x,y,-z+1/2,x+y-t,-u+1/2); (x,y,z,t,u); (-x,-y,-z,-t,-u)

Standard settings

Superspace group: 11.2.6.4 P2_1/m(1/2,0,g1)00(0,0,g2)00 [Y:2.73]
Bravais class: 2.6 P2/m(1/2,0,g1)(0,0,g2) [JJdW:2.6]
Transformation to supercentered setting: A1=2a1+a4, A2=a2, A3=a3, A4=a4, A5=a5

BASIC SPACE GROUP SETTING
Modulation vectors: q1'=(1/2,0,g1), q2'=(0,0,g2)
Centering: (0,0,0,0,0)
Non-lattice generators: (-x,-y,z+1/2,-x+t,u); (x,y,-z+1/2,x-t,-u)
Non-lattice operators: (x,y,z,t,u); (-x,-y,z+1/2,-x+t,u); (-x,-y,-z,-t,-u); (x,y,-z+1/2,x-t,-u)

SUPERCENTERED SETTING
Modulation vectors: Q1'=(0,0,G1), Q2'=(0,0,G2), where G1=g1, G2=g2
Centering: (0,0,0,0,0); (1/2,0,0,1/2,0)
Non-lattice generators: (-X,-Y,Z+1/2,T,U); (X,Y,-Z+1/2,-T,-U)
Non-lattice operators: (X,Y,Z,T,U); (-X,-Y,Z+1/2,T,U); (-X,-Y,-Z,-T,-U); (X,Y,-Z+1/2,-T,-U)
Reflection conditions: HKLMN:H+M=2n; 00LMN:L=2n

Affine transformation to standard basic space group setting

$S * g(input) * S^{-1} = g(standard)$,
where g is an augmented matrix for an operation in the superspace group.
Also, S * r(input) = r(standard),
where r is an augmented position vector, (x,y,z,t,u,1).

$$S = \begin{pmatrix} 1&1&0&0&0&0\\ 0&1&0&0&0&0\\ 0&0&1&0&0&0\\ 0&0&0&1&0&0\\ 0&0&1&0&1&0\\ 0&0&0&0&0&1 \end{pmatrix} \quad S^{-1} = \begin{pmatrix} 1&-1&0&0&0&0\\ 0&1&0&0&0&0\\ 0&0&1&0&0&0\\ 0&0&0&1&0&0\\ 0&0&-1&0&1&0\\ 0&0&0&0&0&1 \end{pmatrix}$$

a1' = a1
a2' = -a1 + a2
a3' = a3

a1 = a1'
a2 = a1' + a2'
a3 = a3'

a1*' = a1* + a2*
a2*' = a2*
a3*' = a3*

a1* = a1*' - a2*'
a2* = a2*'
a3* = a3*'

q1' = q1 = (1/2,0,g1)
q2' = q2 + a3* = (0,0,g2)

q1 = q1' = (1/2,1/2,g1)
q2 = q2' - a3*' = (0,0,g2-1)

findssg P2$_1$/m(1/2,1/2,g1)s0(0,0,g2)s0

Input setting

Centering
none
Operators
(-x,-y,z+1/2,-x-y+t+1/2,u+1/2); (x,y,-z+1/2,x+y-t+1/2,-u+1/2); (x,y,z,t,u); (-x,-y,-z,-t,-u)

Standard settings

Superspace group: 11.2.6.4 P2_1/m(1/2,0,g1)00(0,0,g2)00 [Y:2.73]
Bravais class: 2.6 P2/m(1/2,0,g1)(0,0,g2) [JJdW:2.6]
Transformation to supercentered setting: A1=2a1+a4, A2=a2, A3=a3, A4=a4, A5=a5

BASIC SPACE GROUP SETTING
Modulation vectors: q1'=(1/2,0,g1), q2'=(0,0,g2)
Centering: (0,0,0,0,0)
Non-lattice generators: (-x,-y,z+1/2,-x+t,u); (x,y,-z+1/2,x-t,-u)
Non-lattice operators: (x,y,z,t,u); (-x,-y,z+1/2,-x+t,u); (-x,-y,-z,-t,-u); (x,y,-z+1/2,x-t,-u)

SUPERCENTERED SETTING
Modulation vectors: Q1'=(0,0,G1), Q2'=(0,0,G2), where G1=g1, G2=g2
Centering: (0,0,0,0,0); (1/2,0,0,1/2,0)
Non-lattice generators: (-X,-Y,Z+1/2,T,U); (X,Y,-Z+1/2,-T,-U)
Non-lattice operators: (X,Y,Z,T,U); (-X,-Y,Z+1/2,T,U); (-X,-Y,-Z,-T,-U); (X,Y,-Z+1/2,-T,-U)
Reflection conditions: HKLMN:H+M=2n; 00LMN:L=2n

Affine transformation to standard basic space group setting

S * g(input) * S $^{-1}$ = g(standard),
where g is an augmented matrix for an operation in the superspace group.
Also, S * r(input) = r(standard),
where r is an augmented position vector, (x,y,z,t,u,1).

$$S = \begin{pmatrix} 1 & 1 & 0 & 0 & 0 & 1/2 \\ 0 & 1 & 0 & 0 & 0 & 0 \\ 0 & 0 & 1 & 0 & 0 & 0 \\ 0 & 0 & 0 & 1 & 0 & 0 \\ 0 & 0 & 1 & 0 & 1 & 0 \\ 0 & 0 & 0 & 0 & 0 & 1 \end{pmatrix} \quad S^{-1} = \begin{pmatrix} 1 & -1 & 0 & 0 & 0 & -1/2 \\ 0 & 1 & 0 & 0 & 0 & 0 \\ 0 & 0 & 1 & 0 & 0 & 0 \\ 0 & 0 & 0 & 1 & 0 & 0 \\ 0 & 0 & -1 & 0 & 1 & 0 \\ 0 & 0 & 0 & 0 & 0 & 1 \end{pmatrix}$$

a1' = a1
a2' = -a1 + a2
a3' = a3

a1 = a1'
a2 = a1' + a2'
a3 = a3'

a1*' = a1* + a2*
a2*' = a2*
a3*' = a3*

a1* = a1*' - a2*'
a2* = a2*'
a3* = a3*'

q1' = q1 = (1/2,0,g1)
q2' = q2 + a3* = (0,0,g2)

q1 = q1' = (1/2,1/2,g1)
q2 = q2' - a3*' = (0,0,g2-1)

findssg $P2_1/m(1/2,1/2,g1)s0(0,0,g2)00$

Input setting

Centering
none
Operators
(-x,-y,z+1/2,-x-y+t+1/2,u); (x,y,-z+1/2,x+y-t+1/2,-u); (x,y,z,t,u); (-x,-y,-z,-t,-u)

Standard settings

Superspace group: 11.2.6.4 P2_1/m(1/2,0,g1)00(0,0,g2)00 [Y:2.73]
Bravais class: 2.6 P2/m(1/2,0,g1)(0,0,g2) [JJdW:2.6]
Transformation to supercentered setting: A1=2a1+a4, A2=a2, A3=a3, A4=a4, A5=a5

BASIC SPACE GROUP SETTING
Modulation vectors: q1'=(1/2,0,g1), q2'=(0,0,g2)
Centering: (0,0,0,0,0)
Non-lattice generators: (-x,-y,z+1/2,-x+t,u); (x,y,-z+1/2,x-t,-u)
Non-lattice operators: (x,y,z,t,u); (-x,-y,z+1/2,-x+t,u); (-x,-y,-z,-t,-u); (x,y,-z+1/2,x-t,-u)

SUPERCENTERED SETTING
Modulation vectors: Q1'=(0,0,G1), Q2'=(0,0,G2), where G1=g1, G2=g2
Centering: (0,0,0,0,0); (1/2,0,0,1/2,0)
Non-lattice generators: (-X,-Y,Z+1/2,T,U); (X,Y,-Z+1/2,-T,-U)
Non-lattice operators: (X,Y,Z,T,U); (-X,-Y,Z+1/2,T,U); (-X,-Y,-Z,-T,-U); (X,Y,-Z+1/2,-T,-U)
Reflection conditions: HKLMN:H+M=2n; 00LMN:L=2n

Affine transformation to standard basic space group setting

$S * g(input) * S^{-1} = g(standard)$,
where g is an augmented matrix for an operation in the superspace group.
Also, S * r(input) = r(standard),
where r is an augmented position vector, (x,y,z,t,u,1).

$$S = \begin{pmatrix} 1 & 1 & 0 & 0 & 0 & 1/2 \\ 0 & 1 & 0 & 0 & 0 & 0 \\ 0 & 0 & 1 & 0 & 0 & 0 \\ 0 & 0 & 0 & 1 & 0 & 0 \\ 0 & 0 & 0 & 0 & 1 & 0 \\ 0 & 0 & 0 & 0 & 0 & 1 \end{pmatrix} \quad S^{-1} = \begin{pmatrix} 1 & -1 & 0 & 0 & 0 & -1/2 \\ 0 & 1 & 0 & 0 & 0 & 0 \\ 0 & 0 & 1 & 0 & 0 & 0 \\ 0 & 0 & 0 & 1 & 0 & 0 \\ 0 & 0 & 0 & 0 & 1 & 0 \\ 0 & 0 & 0 & 0 & 0 & 1 \end{pmatrix}$$

a1' = a1
a2' = -a1 + a2
a3' = a3

a1 = a1'
a2 = a1' + a2'
a3 = a3'

a1*' = a1* + a2*
a2*' = a2*
a3*' = a3*

a1* = a1*' - a2*'
a2* = a2*'
a3* = a3*'

q1' = q1 = (1/2,0,g1)
q2' = q2 = (0,0,g2)

q1 = q1' = (1/2,1/2,g1)
q2 = q2' = (0,0,g2)

findssg P2$_1$/m(1/2,b1,1/2)00(0,b2,0)s0

Input setting

Centering
none
Operators
(-x,y+1/2,-z,-x-z+t,u+1/2); (-x,-y,-z,-t,-u); (x,y,z,t,u); (x,-y+1/2,z,x+z-t,-u+1/2)

Standard settings

Superspace group: 11.2.6.4 P2_1/m(1/2,0,g1)00(0,0,g2)00 [Y:2.73]
Bravais class: 2.6 P2/m(1/2,0,g1)(0,0,g2) [JJdW:2.6]
Transformation to supercentered setting: A1=2a1+a4, A2=a2, A3=a3, A4=a4, A5=a5

BASIC SPACE GROUP SETTING
Modulation vectors: q1'=(1/2,0,g1), q2'=(0,0,g2)
Centering: (0,0,0,0,0)
Non-lattice generators: (-x,-y,z+1/2,-x+t,u); (x,y,-z+1/2,x-t,-u)
Non-lattice operators: (x,y,z,t,u); (-x,-y,z+1/2,-x+t,u); (-x,-y,-z,-t,-u); (x,y,-z+1/2,x-t,-u)

SUPERCENTERED SETTING
Modulation vectors: Q1'=(0,0,G1), Q2'=(0,0,G2), where G1=g1, G2=g2
Centering: (0,0,0,0,0); (1/2,0,0,1/2,0)
Non-lattice generators: (-X,-Y,Z+1/2,T,U); (X,Y,-Z+1/2,-T,-U)
Non-lattice operators: (X,Y,Z,T,U); (-X,-Y,Z+1/2,T,U); (-X,-Y,-Z,-T,-U); (X,Y,-Z+1/2,-T,-U)
Reflection conditions: HKLMN:H+M=2n; 00LMN:L=2n

Affine transformation to standard basic space group setting

S * g(input) * S $^{-1}$ = g(standard),
where g is an augmented matrix for an operation in the superspace group.
Also, S * r(input) = r(standard),
where r is an augmented position vector, (x,y,z,t,u,1).

$$S = \begin{pmatrix} 1&0&1&0&0&0\\ 1&0&0&0&0&0\\ 0&1&0&0&0&0\\ 0&0&0&1&0&0\\ 0&1&0&0&1&0\\ 0&0&0&0&0&1 \end{pmatrix} \quad S^{-1} = \begin{pmatrix} 0&1&0&0&0&0\\ 0&0&1&0&0&0\\ 1&-1&0&0&0&0\\ 0&0&0&1&0&0\\ 0&0&-1&0&1&0\\ 0&0&0&0&0&1 \end{pmatrix}$$

a1' = a3
a2' = a1 - a3

$a_3' = a_2$

$a_1 = a_1' + a_2'$
$a_2 = a_3'$
$a_3 = a_1'$

$a_1^{*\prime} = a_1^* + a_3^*$
$a_2^{*\prime} = a_1^*$
$a_3^{*\prime} = a_2^*$

$a_1^* = a_2^{*\prime}$
$a_2^* = a_3^{*\prime}$
$a_3^* = a_1^{*\prime} - a_2^{*\prime}$

$q_1' = q_1 = (1/2,0,g_1)$
$q_2' = q_2 + a_2^* = (0,0,g_2)$

$q_1 = q_1' = (1/2,g_1,1/2)$
$q_2 = q_2' - a_3^{*\prime} = (0,g_2-1,0)$

findssg $P2_1/m(0,b2,0)s0\ (1/2,b1,1/2)00$

This is the published setting for NbSe3. Permuted basic-structure axis; interchanged q-vectors; alternate q-vector (1/2, 1/2, g1). Requires a diagonal unit cell for the basic structure for transforming to the standard setting.
a1new = a3; a2new=a1-a3; a3new=a2; a4new=a5; a5new=a4.
a*1new= a*1+a*3; a*2new=a*1; a*3new=a*2; q1new= q2; q2new= a*2+q1.

Input setting

Centering
none
Operators
(-x,y+1/2,-z,t+1/2,-x-z+u); (-x,-y,-z,-t,-u); (x,y,z,t,u); (x,-y+1/2,z,-t+1/2,x+z-u)

Standard settings

Superspace group: 11.2.6.4 P2_1/m(1/2,0,g1)00(0,0,g2)00 [Y:2.73]
Bravais class: 2.6 P2/m(1/2,0,g1)(0,0,g2) [JJdW:2.6]
Transformation to supercentered setting: A1=2a1+a4, A2=a2, A3=a3, A4=a4, A5=a5

BASIC SPACE GROUP SETTING
Modulation vectors: q1'=(1/2,0,g1), q2'=(0,0,g2)
Centering: (0,0,0,0,0)
Non-lattice generators: (-x,-y,z+1/2,-x+t,u); (x,y,-z+1/2,x-t,-u)
Non-lattice operators: (x,y,z,t,u); (-x,-y,z+1/2,-x+t,u); (-x,-y,-z,-t,-u); (x,y,-z+1/2,x-t,-u)

SUPERCENTERED SETTING
Modulation vectors: Q1'=(0,0,G1), Q2'=(0,0,G2), where G1=g1, G2=g2
Centering: (0,0,0,0,0); (1/2,0,0,1/2,0)
Non-lattice generators: (-X,-Y,Z+1/2,T,U); (X,Y,-Z+1/2,-T,-U)
Non-lattice operators: (X,Y,Z,T,U); (-X,-Y,Z+1/2,T,U); (-X,-Y,-Z,-T,-U); (X,Y,-Z+1/2,-T,-U)
Reflection conditions: HKLMN:H+M=2n; 00LMN:L=2n

Affine transformation to standard basic space group setting

$S * g(\text{input}) * S^{-1} = g(\text{standard})$,
where g is an augmented matrix for an operation in the superspace group.
Also, S * r(input) = r(standard),
where r is an augmented position vector, (x,y,z,t,u,1).

$$S = \begin{pmatrix} 1&0&1&0&0&0\\ 1&0&0&0&0&0\\ 0&1&0&0&0&0\\ 0&0&0&0&1&0\\ 0&1&0&1&0&0\\ 0&0&0&0&0&1 \end{pmatrix} \quad S^{-1} = \begin{pmatrix} 0&1&0&0&0&0\\ 0&0&1&0&0&0\\ 1&-1&0&0&0&0\\ 0&0&-1&0&1&0\\ 0&0&0&1&0&0\\ 0&0&0&0&0&1 \end{pmatrix}$$

a1' = a3
a2' = a1 - a3
a3' = a2

a1 = a1' + a2'
a2 = a3'
a3 = a1'

a1*' = a1* + a3*
a2*' = a1*
a3*' = a2*

a1* = a2*'
a2* = a3*'
a3* = a1*' - a2*'

q1' = q2 = (1/2,0,g1)
q2' = q1 + a2* = (0,0,g2)

q1 = q2' - a3*' = (0,g2-1,0)
q2 = q1' = (1/2,g1,1/2)

transformssg

Applied to standard BSG setting of 11.2.6.4 P2$_1$/m(1/2,0,g1)00(0,0,g2)00. Transformed setting is again the standard BSG setting.

Input setting

Centering
none
Operators
(-x,-y,z+1/2,-x+t,u); (x,y,-z+1/2,x-t,-u); (x,y,z,t,u); (-x,-y,-z,-t,-u)
q vectors
$\mathbf{q}_1$=(1/2,0,0.260); $\mathbf{q}_2$=(0,0,1.241)

New setting

Centering
none
Operators
(-x,-y,z+1/2,-x+t,u+1); (x,y,-z+1/2,x-t,-u+1); (x,y,z,t,u); (-x,-y,-z,-t,-u)
q vectors
$\mathbf{q}_1$'=(1/2,0,0.260); $\mathbf{q}_2$'=(0,0,0.759)

Affine transformation to new setting

$$S = \begin{pmatrix} 1 & 0 & 0 & 0 & 0 & 0 \\ 0 & 1 & 0 & 0 & 0 & 0 \\ 0 & 0 & 1 & 0 & 0 & 0 \\ 0 & 0 & 0 & 1 & 0 & 0 \\ 0 & 0 & 2 & 0 & -1 & 0 \\ 0 & 0 & 0 & 0 & 0 & 1 \end{pmatrix} \qquad S^{-1} = \begin{pmatrix} 1 & 0 & 0 & 0 & 0 & 0 \\ 0 & 1 & 0 & 0 & 0 & 0 \\ 0 & 0 & 1 & 0 & 0 & 0 \\ 0 & 0 & 0 & 1 & 0 & 0 \\ 0 & 0 & 2 & 0 & -1 & 0 \\ 0 & 0 & 0 & 0 & 0 & 1 \end{pmatrix}$$

g' = S * g * S $^{-1}$, where g is an augmented matrix for an operation in the superspace group.
r' = S * **r**, where **r** is an augmented position vector, (x,y,z,t,u,1).

Basis vectors of the lattice
$\mathbf{a}_1' = \mathbf{a}_1$; $\mathbf{a}_2' = \mathbf{a}_2$; $\mathbf{a}_3' = \mathbf{a}_3$
$\mathbf{a}_1 = \mathbf{a}_1'$; $\mathbf{a}_2 = \mathbf{a}_2'$; $\mathbf{a}_3 = \mathbf{a}_3'$
Basis vectors of the reciprocal lattice
$\mathbf{a}_1^{*\prime} = \mathbf{a}_1^*$; $\mathbf{a}_2^{*\prime} = \mathbf{a}_2^*$; $\mathbf{a}_3^{*\prime} = \mathbf{a}_3^*$
$\mathbf{a}_1^* = \mathbf{a}_1^{*\prime}$; $\mathbf{a}_2^* = \mathbf{a}_2^{*\prime}$; $\mathbf{a}_3^* = \mathbf{a}_3^{*\prime}$
q vectors
$\mathbf{q}_1' = \mathbf{q}_1$ = (1/2,0,0.260); $\mathbf{q}_2' = -\mathbf{q}_2 + 2\,\mathbf{a}_3^*$ = (0,0,0.759)
$\mathbf{q}_1 = \mathbf{q}_1'$ = (1/2,0,0.260); $\mathbf{q}_2 = -\mathbf{q}_2' + 2\,\mathbf{a}_3^{*\prime}$ = (0,0,1.241)
Origin
$\boldsymbol{\tau}' = 0$
$\boldsymbol{\tau} = 0$
