# Supplementary material for: Equivalence of superspace groups
Source: Acta Crystallogr A. 2012 Nov 14;69(Pt 1):75–90. doi: 10.1107/S0108767312041657 (PMC3553647; doi:10.1107/S0108767312041657)
Supplement: Supplementary file 1 [file a-69-00075-sup1.zip › ssg2d_p222_00g1_120g2.pdf]

16.2.19.3 P222(0,1/2,g1)000(0,0,g2)000

Superspace group: 16.2.19.3 P222(0,1/2,g1)000(0,0,g2)000 [Y:2.141]
Bravais class: 2.19 Pmmm(0,1/2,g1)(0,0,g2) [JJdW:2.19]
Transformation to supercentered setting: A1=a1, A2=2a2+a4, A3=a3, A4=a4, A5=a5

BASIC SPACE GROUP SETTING
Modulation vectors: q1=(0,1/2,g1), q2=(0,0,g2)
Centering: (0,0,0,0,0)
Non-lattice generators: (x,-y,-z,-t,-u); (-x,y,-z,y-t,-u); (-x,-y,z,-y+t,u)
Non-lattice operators: (x,y,z,t,u); (x,-y,-z,-t,-u); (-x,y,-z,y-t,-u); (-x,-y,z,-y+t,u)

SUPERCENTERED SETTING
Modulation vectors: Q1=(0,0,G1), Q2=(0,0,G2), where G1=g1, G2=g2
Centering: (0,0,0,0,0); (0,1/2,0,1/2,0)
Non-lattice generators: (X,-Y,-Z,-T,-U); (-X,Y,-Z,-T,-U); (-X,-Y,Z,T,U)
Non-lattice operators: (X,Y,Z,T,U); (X,-Y,-Z,-T,-U); (-X,Y,-Z,-T,-U); (-X,-Y,Z,T,U)
Reflection conditions: HKLMN:K+M=2n

-----

findssg P222(0,1/2,g1)000(0,0,g2)000

Generators of the standard BSG setting have been entered into findssg.

Input setting

Centering
none
Operators
(x,-y,-z,-t,-u); (-x,y,-z,y-t,-u); (-x,-y,z,-y+t,u); (x,y,z,t,u)

Standard settings

Superspace group: 16.2.19.3 P222(0,1/2,g1)000(0,0,g2)000 [Y:2.141]
Bravais class: 2.19 Pmmm(0,1/2,g1)(0,0,g2) [JJdW:2.19]
Transformation to supercentered setting: A1=a1, A2=2a2+a4, A3=a3, A4=a4, A5=a5

BASIC SPACE GROUP SETTING
Modulation vectors: q1'=(0,1/2,g1), q2'=(0,0,g2)
Centering: (0,0,0,0,0)
Non-lattice generators: (x,-y,-z,-t,-u); (-x,y,-z,y-t,-u); (-x,-y,z,-y+t,u)
Non-lattice operators: (x,y,z,t,u); (x,-y,-z,-t,-u); (-x,y,-z,y-t,-u); (-x,-y,z,-y+t,u)

SUPERCENTERED SETTING
Modulation vectors: Q1'=(0,0,G1), Q2'=(0,0,G2), where G1=g1, G2=g2
Centering: (0,0,0,0,0); (0,1/2,0,1/2,0)
Non-lattice generators: (X,-Y,-Z,-T,-U); (-X,Y,-Z,-T,-U); (-X,-Y,Z,T,U)
Non-lattice operators: (X,Y,Z,T,U); (X,-Y,-Z,-T,-U); (-X,Y,-Z,-T,-U); (-X,-Y,Z,T,U)
Reflection conditions: HKLMN:K+M=2n

Affine transformation to standard basic space group setting

$S * g(input) * S^{-1} = g(standard)$,
where g is an augmented matrix for an operation in the superspace group.
Also, S * r(input) = r(standard),
where r is an augmented position vector, (x,y,z,t,u,1).

$$S = \begin{matrix} 1 & 0 & 0 & 0 & 0 & 0 \\ 0 & 1 & 0 & 0 & 0 & 0 \\ 0 & 0 & 1 & 0 & 0 & 0 \\ 0 & 0 & 0 & 1 & 0 & 0 \\ 0 & 0 & 0 & 0 & 1 & 0 \\ 0 & 0 & 0 & 0 & 0 & 1 \end{matrix} \quad S^{-1} = \begin{matrix} 1 & 0 & 0 & 0 & 0 & 0 \\ 0 & 1 & 0 & 0 & 0 & 0 \\ 0 & 0 & 1 & 0 & 0 & 0 \\ 0 & 0 & 0 & 1 & 0 & 0 \\ 0 & 0 & 0 & 0 & 1 & 0 \\ 0 & 0 & 0 & 0 & 0 & 1 \end{matrix}$$

a1' = a1
a2' = a2
a3' = a3

a1 = a1'
a2 = a2'
a3 = a3'

a1*' = a1*
a2*' = a2*
a3*' = a3*

a1* = a1*'
a2* = a2*'
a3* = a3*'

q1' = q1 = (0,1/2,g1)
q2' = q2 = (0,0,g2)

q1 = q1' = (0,1/2,g1)
q2 = q2' = (0,0,g2)

findssg X222(0,0,g1)000(0,0,g2)000

Generators of the standard supercentered setting have been entered into findssg.

Input setting

Centering
(0,0,0,0,0); (0,1/2,0,1/2,0)
Operators
(x,-y,-z,-t,-u); (-x,y,-z,-t,-u); (-x,-y,z,t,u); (x,y,z,t,u)

Standard settings

Superspace group: 16.2.19.3 P222(0,1/2,g1)000(0,0,g2)000 [Y:2.141]
Bravais class: 2.19 Pmmm(0,1/2,g1)(0,0,g2) [JJdW:2.19]
Transformation to supercentered setting: A1=a1, A2=2a2+a4, A3=a3, A4=a4, A5=a5

BASIC SPACE GROUP SETTING
Modulation vectors: q1'=(0,1/2,g1), q2'=(0,0,g2)
Centering: (0,0,0,0,0)
Non-lattice generators: (x,-y,-z,-t,-u); (-x,y,-z,y-t,-u); (-x,-y,z,-y+t,u)
Non-lattice operators: (x,y,z,t,u); (x,-y,-z,-t,-u); (-x,y,-z,y-t,-u); (-x,-y,z,-y+t,u)

SUPERCENTERED SETTING
Modulation vectors: Q1'=(0,0,G1), Q2'=(0,0,G2), where G1=g1, G2=g2
Centering: (0,0,0,0,0); (0,1/2,0,1/2,0)
Non-lattice generators: (X,-Y,-Z,-T,-U); (-X,Y,-Z,-T,-U); (-X,-Y,Z,T,U)
Non-lattice operators: (X,Y,Z,T,U); (X,-Y,-Z,-T,-U); (-X,Y,-Z,-T,-U); (-X,-Y,Z,T,U)
Reflection conditions: HKLMN:K+M=2n

Affine transformation to standard basic space group setting

S * g(input) * S^{-1} = g(standard),
where g is an augmented matrix for an operation in the superspace group.
Also, S * r(input) = r(standard),
where r is an augmented position vector, (x,y,z,t,u,1).

$$S = \begin{pmatrix} 1&0&0&0&0&0\\ 0&2&0&0&0&0\\ 0&0&1&0&0&0\\ 0&1&0&1&0&0\\ 0&0&0&0&1&0\\ 0&0&0&0&0&1 \end{pmatrix} \quad S^{-1} = \begin{pmatrix} 1&0&0&0&0&0\\ 0&1/2&0&0&0&0\\ 0&0&1&0&0&0\\ 0&-1/2&0&1&0&0\\ 0&0&0&0&1&0\\ 0&0&0&0&0&1 \end{pmatrix}$$

a1' = a1
a2' = 1/2 a2
a3' = a3

a1 = a1'
a2 = 2 a2'
a3 = a3'

a1*' = a1*
a2*' = 2 a2*
a3*' = a3*

a1* = a1*'
a2* = 1/2 a2*'
a3* = a3*'

q1' = q1 + a2* = (0,1/2,g1)
q2' = q2 = (0,0,g2)

q1 = q1' - 1/2 a2*' = (0,0,g1)
q2 = q2' = (0,0,g2)

findssg P222(1/2,b1,0)000(1/2,b2,0)000

Input setting

Operators of this alternative BSG setting have been given into findssg.

Input setting

Centering
none
Operators
(x,-y,-z,x-t,x-u); (-x,y,-z,-x+t,-x+u); (-x,-y,z,-t,-u); (x,y,z,t,u)

Standard settings

Superspace group: 16.2.19.3 P222(0,1/2,g1)000(0,0,g2)000 [Y:2.141]
Bravais class: 2.19 Pmmm(0,1/2,g1)(0,0,g2) [JJdW:2.19]
Transformation to supercentered setting: A1=a1, A2=2a2+a4, A3=a3, A4=a4, A5=a5

BASIC SPACE GROUP SETTING
Modulation vectors: q1'=(0,1/2,g1), q2'=(0,0,g2)
Centering: (0,0,0,0,0)
Non-lattice generators: (x,-y,-z,-t,-u); (-x,y,-z,y-t,-u); (-x,-y,z,-y+t,u)
Non-lattice operators: (x,y,z,t,u); (x,-y,-z,-t,-u); (-x,y,-z,y-t,-u); (-x,-y,z,-y+t,u)

SUPERCENTERED SETTING
Modulation vectors: Q1'=(0,0,G1), Q2'=(0,0,G2), where G1=g1, G2=g2
Centering: (0,0,0,0,0); (0,1/2,0,1/2,0)
Non-lattice generators: (X,-Y,-Z,-T,-U); (-X,Y,-Z,-T,-U); (-X,-Y,Z,T,U)
Non-lattice operators: (X,Y,Z,T,U); (X,-Y,-Z,-T,-U); (-X,Y,-Z,-T,-U); (-X,-Y,Z,T,U)
Reflection conditions: HKLMN:K+M=2n

Affine transformation to standard basic space group setting

$S * g(\text{input}) * S^{-1} = g(\text{standard})$,
where g is an augmented matrix for an operation in the superspace group.
Also, S * r(input) = r(standard),
where r is an augmented position vector, (x,y,z,t,u,1).

$$S = \begin{pmatrix} 0 & 0 & 1 & 0 & 0 & 0 \\ 1 & 0 & 0 & 0 & 0 & 0 \\ 0 & 1 & 0 & 0 & 0 & 0 \\ 0 & 0 & 0 & 1 & 0 & 0 \\ 0 & 0 & 0 & -1 & 1 & 0 \\ 0 & 0 & 0 & 0 & 0 & 1 \end{pmatrix} \quad S^{-1} = \begin{pmatrix} 0 & 1 & 0 & 0 & 0 & 0 \\ 0 & 0 & 1 & 0 & 0 & 0 \\ 1 & 0 & 0 & 0 & 0 & 0 \\ 0 & 0 & 0 & 1 & 0 & 0 \\ 0 & 0 & 0 & 1 & 1 & 0 \\ 0 & 0 & 0 & 0 & 0 & 1 \end{pmatrix}$$

a1' = a3
a2' = a1
a3' = a2

a1 = a2'
a2 = a3'
a3 = a1'

a1*' = a3*
a2*' = a1*
a3*' = a2*

a1* = a2*'
a2* = a3*'
a3* = a1*'

q1' = q1 = (0,1/2,g1)
q2' = -q1 + q2 = (0,0,g2)

q1 = q1' = (1/2,g1,0)
q2 = q1' + q2' = (1/2,g1+g2,0)
