# Supplementary material for: Equivalence of superspace groups
Source: Acta Crystallogr A. 2012 Nov 14;69(Pt 1):75–90. doi: 10.1107/S0108767312041657 (PMC3553647; doi:10.1107/S0108767312041657)
Supplement: Supplementary file 1 [file a-69-00075-sup1.zip › ssg2d_p2m_120g1g2.pdf]

10.2.6.7 P2/m(1/2,0,g1)00(0,0,g2)00

Superspace group: 10.2.6.7 P2/m(1/2,0,g1)00(0,0,g2)00 [Y:2.72]
Bravais class: 2.6 P2/m(1/2,0,g1)(0,0,g2)   [JJdW:2.6]
Transformation to supercentered setting: A1=2a1+a4, A2=a2, A3=a3, A4=a4, A5=a5

BASIC SPACE GROUP SETTING
Modulation vectors: q1=(1/2,0,g1), q2=(0,0,g2)
Centering: (0,0,0,0,0)
Non-lattice generators: (-x,-y,z,-x+t,u); (x,y,-z,x-t,-u)
Non-lattice operators: (x,y,z,t,u); (-x,-y,z,-x+t,u); (-x,-y,-z,-t,-u); (x,y,-z,x-t,-u)

SUPERCENTERED SETTING
Modulation vectors: Q1=(0,0,G1), Q2=(0,0,G2), where G1=g1, G2=g2
Centering: (0,0,0,0,0); (1/2,0,0,1/2,0)
Non-lattice generators: (-X,-Y,Z,T,U); (X,Y,-Z,-T,-U)
Non-lattice operators: (X,Y,Z,T,U); (-X,-Y,Z,T,U); (-X,-Y,-Z,-T,-U); (X,Y,-Z,-T,-U)
Reflection conditions: HKLMN:H+M=2n

-----

findssg P2/m(1/2,0,g1)00(0,0,g2)00

Operators of the BSG setting have been entered into findssg.

Input setting

Centering
none
Operators
(-x,-y,z,-x+t,u); (-x,-y,-z,-t,-u); (x,y,z,t,u); (x,y,-z,x-t,-u)

Standard settings

Superspace group: 10.2.6.7 P2/m(1/2,0,g1)00(0,0,g2)00 [Y:2.72]
Bravais class: 2.6 P2/m(1/2,0,g1)(0,0,g2) [JJdW:2.6]
Transformation to supercentered setting: A1=2a1+a4, A2=a2, A3=a3, A4=a4, A5=a5

BASIC SPACE GROUP SETTING
Modulation vectors: q1'=(1/2,0,g1), q2'=(0,0,g2)
Centering: (0,0,0,0,0)
Non-lattice generators: (-x,-y,z,-x+t,u); (x,y,-z,x-t,-u)
Non-lattice operators: (x,y,z,t,u); (-x,-y,z,-x+t,u); (-x,-y,-z,-t,-u); (x,y,-z,x-t,-u)

SUPERCENTERED SETTING
Modulation vectors: Q1'=(0,0,G1), Q2'=(0,0,G2), where G1=g1, G2=g2
Centering: (0,0,0,0,0); (1/2,0,0,1/2,0)
Non-lattice generators: (-X,-Y,Z,T,U); (X,Y,-Z,-T,-U)
Non-lattice operators: (X,Y,Z,T,U); (-X,-Y,Z,T,U); (-X,-Y,-Z,-T,-U); (X,Y,-Z,-T,-U)
Reflection conditions: HKLMN:H+M=2n

Affine transformation to standard basic space group setting

$S * g(\text{input}) * S^{-1} = g(\text{standard})$,
where g is an augmented matrix for an operation in the superspace group.
Also, S * r(input) = r(standard),
where r is an augmented position vector, (x,y,z,t,u,1).

$$S = \begin{matrix} 1 & 0 & 0 & 0 & 0 & 0 \\ 0 & 1 & 0 & 0 & 0 & 0 \\ 0 & 0 & 1 & 0 & 0 & 0 \\ 0 & 0 & 0 & 1 & 0 & 0 \\ 0 & 0 & 0 & 0 & 1 & 0 \\ 0 & 0 & 0 & 0 & 0 & 1 \end{matrix} \quad S^{-1} = \begin{matrix} 1 & 0 & 0 & 0 & 0 & 0 \\ 0 & 1 & 0 & 0 & 0 & 0 \\ 0 & 0 & 1 & 0 & 0 & 0 \\ 0 & 0 & 0 & 1 & 0 & 0 \\ 0 & 0 & 0 & 0 & 1 & 0 \\ 0 & 0 & 0 & 0 & 0 & 1 \end{matrix}$$

a1' = a1
a2' = a2
a3' = a3

a1 = a1'
a2 = a2'
a3 = a3'

a1*' = a1*
a2*' = a2*
a3*' = a3*

a1* = a1*'
a2* = a2*'
a3* = a3*'

q1' = q1 = (1/2,0,g1)
q2' = q2 = (0,0,g2)

q1 = q1' = (1/2,0,g1)
q2 = q2' = (0,0,g2)

findssg X2/m(0,0,g1)00(0,0,g2)00

Operators of the standard supercentered setting have been entered into findssg.

Input setting

Centering
(0,0,0,0,0); (1/2,0,0,1/2,0)
Operators
(-x,-y,z,t,u); (-x,-y,-z,-t,-u); (x,y,z,t,u); (x,y,-z,-t,-u)

Standard settings

Superspace group: 10.2.6.7 P2/m(1/2,0,g1)00(0,0,g2)00 [Y:2.72]
Bravais class: 2.6 P2/m(1/2,0,g1)(0,0,g2) [JJdW:2.6]
Transformation to supercentered setting: A1=2a1+a4, A2=a2, A3=a3, A4=a4, A5=a5

BASIC SPACE GROUP SETTING
Modulation vectors: q1'=(1/2,0,g1), q2'=(0,0,g2)
Centering: (0,0,0,0,0)
Non-lattice generators: (-x,-y,z,-x+t,u); (x,y,-z,x-t,-u)
Non-lattice operators: (x,y,z,t,u); (-x,-y,z,-x+t,u); (-x,-y,-z,-t,-u); (x,y,-z,x-t,-u)

SUPERCENTERED SETTING
Modulation vectors: Q1'=(0,0,G1), Q2'=(0,0,G2), where G1=g1, G2=g2
Centering: (0,0,0,0,0); (1/2,0,0,1/2,0)
Non-lattice generators: (-X,-Y,Z,T,U); (X,Y,-Z,-T,-U)
Non-lattice operators: (X,Y,Z,T,U); (-X,-Y,Z,T,U); (-X,-Y,-Z,-T,-U); (X,Y,-Z,-T,-U)
Reflection conditions: HKLMN:H+M=2n

Affine transformation to standard basic space group setting

$S * g(input) * S^{-1} = g(standard)$,
where g is an augmented matrix for an operation in the superspace group.
Also, S * r(input) = r(standard),
where r is an augmented position vector, (x,y,z,t,u,1).

$$S = \begin{pmatrix} 2 & 0 & 0 & 0 & 0 & 0 \\ 0 & 1 & 0 & 0 & 0 & 0 \\ 0 & 0 & 1 & 0 & 0 & 0 \\ 1 & 0 & 0 & 1 & 0 & 0 \\ 0 & 0 & 0 & 0 & 1 & 0 \\ 0 & 0 & 0 & 0 & 0 & 1 \end{pmatrix} \quad S^{-1} = \begin{pmatrix} 1/2 & 0 & 0 & 0 & 0 & 0 \\ 0 & 1 & 0 & 0 & 0 & 0 \\ 0 & 0 & 1 & 0 & 0 & 0 \\ -1/2 & 0 & 0 & 1 & 0 & 0 \\ 0 & 0 & 0 & 0 & 1 & 0 \\ 0 & 0 & 0 & 0 & 0 & 1 \end{pmatrix}$$

$a1' = 1/2\ a1$
$a2' = a2$
$a3' = a3$

$a1 = 2\ a1'$
$a2 = a2'$
$a3 = a3'$

$a1^{*\prime} = 2\ a1^*$
$a2^{*\prime} = a2^*$
$a3^{*\prime} = a3^*$

$a1^* = 1/2\ a1^{*\prime}$
$a2^* = a2^{*\prime}$
$a3^* = a3^{*\prime}$

$q1' = q1 + a1^* = (1/2,0,g1)$
$q2' = q2 = (0,0,g2)$

$q1 = q1' - 1/2\ a1^{*\prime} = (0,0,g1)$
$q2 = q2' = (0,0,g2)$

findssg P2/m(1/2,0,g1)s0(0,0,g2)00

Input of (2,s,0) in findssg shows that only a shift of the origin brings this setting to the standard setting.

Input setting

Centering
none
Operators
(-x,-y,z,-x+t+1/2,u); (-x,-y,-z,-t,-u); (x,y,z,t,u); (x,y,-z,x-t+1/2,-u)

Standard settings

Superspace group: 10.2.6.7 P2/m(1/2,0,g1)00(0,0,g2)00 [Y:2.72]
Bravais class: 2.6 P2/m(1/2,0,g1)(0,0,g2) [JJdW:2.6]
Transformation to supercentered setting: A1=2a1+a4, A2=a2, A3=a3, A4=a4, A5=a5

BASIC SPACE GROUP SETTING
Modulation vectors: q1'=(1/2,0,g1), q2'=(0,0,g2)
Centering: (0,0,0,0,0)
Non-lattice generators: (-x,-y,z,-x+t,u); (x,y,-z,x-t,-u)
Non-lattice operators: (x,y,z,t,u); (-x,-y,z,-x+t,u); (-x,-y,-z,-t,-u); (x,y,-z,x-t,-u)

SUPERCENTERED SETTING
Modulation vectors: Q1'=(0,0,G1), Q2'=(0,0,G2), where G1=g1, G2=g2
Centering: (0,0,0,0,0); (1/2,0,0,1/2,0)
Non-lattice generators: (-X,-Y,Z,T,U); (X,Y,-Z,-T,-U)
Non-lattice operators: (X,Y,Z,T,U); (-X,-Y,Z,T,U); (-X,-Y,-Z,-T,-U); (X,Y,-Z,-T,-U)
Reflection conditions: HKLMN:H+M=2n

Affine transformation to standard basic space group setting

S * g(input) * S $^{-1}$ = g(standard),
where g is an augmented matrix for an operation in the superspace group.
Also, S * r(input) = r(standard),
where r is an augmented position vector, (x,y,z,t,u,1).

$$S = \begin{pmatrix} 1 & 0 & 0 & 0 & 0 & 1/2 \\ 0 & 1 & 0 & 0 & 0 & 0 \\ 0 & 0 & 1 & 0 & 0 & 0 \\ 0 & 0 & 0 & 1 & 0 & 0 \\ 0 & 0 & 0 & 0 & 1 & 0 \\ 0 & 0 & 0 & 0 & 0 & 1 \end{pmatrix} \quad S^{-1} = \begin{pmatrix} 1 & 0 & 0 & 0 & 0 & -1/2 \\ 0 & 1 & 0 & 0 & 0 & 0 \\ 0 & 0 & 1 & 0 & 0 & 0 \\ 0 & 0 & 0 & 1 & 0 & 0 \\ 0 & 0 & 0 & 0 & 1 & 0 \\ 0 & 0 & 0 & 0 & 0 & 1 \end{pmatrix}$$

a1' = a1
a2' = a2
a3' = a3

a1 = a1'
a2 = a2'
a3 = a3'

a1*' = a1*
a2*' = a2*
a3*' = a3*

a1* = a1*'
a2* = a2*'
a3* = a3*'

q1' = q1 = (1/2,0,g1)
q2' = q2 = (0,0,g2)

q1 = q1' = (1/2,0,g1)
q2 = q2' = (0,0,g2)
