# Supplementary material for: Equivalence of superspace groups
Source: Acta Crystallogr A. 2012 Nov 14;69(Pt 1):75–90. doi: 10.1107/S0108767312041657 (PMC3553647; doi:10.1107/S0108767312041657)
Supplement: Supplementary file 1 [file a-69-00075-sup1.zip › ssg2d_p2m_g1g2.pdf]

10.2.5.6 P2/m(0,0,g1)s0(0,0,g2)00

Superspace group: 10.2.5.6 P2/m(0,0,g1)s0(0,0,g2)00 [Y:2.70]
Bravais class: 2.5 P2/m(0,0,g1)(0,0,g2) [JJdW:2.5]
Transformation to supercentered setting: none
Modulation vectors: q1=(0,0,g1), q2=(0,0,g2)
Centering: (0,0,0,0,0)
Non-lattice generators: (-x,-y,z,t+1/2,u); (x,y,-z,-t+1/2,-u)
Non-lattice operators: (x,y,z,t,u); (-x,-y,z,t+1/2,u); (-x,-y,-z,-t,-u); (x,y,-z,-t+1/2,-u)
Reflection conditions: 00lmn:m=2n

A compound with this SSG has not been found.
No other SSG exists for this symmetry.

-----

findssg P2/m(0,0,g1)s0(0,0,g2)00

Input setting

Centering
none
Operators
(-x,-y,z,t+1/2,u); (-x,-y,-z,-t,-u); (x,y,z,t,u); (x,y,-z,-t+1/2,-u)

Standard settings

Superspace group: 10.2.5.6 P2/m(0,0,g1)s0(0,0,g2)00 [Y:2.70]
Bravais class: 2.5 P2/m(0,0,g1)(0,0,g2) [JJdW:2.5]
Transformation to supercentered setting: none
Modulation vectors: q1'=(0,0,g1), q2'=(0,0,g2)
Centering: (0,0,0,0,0)
Non-lattice generators: (-x,-y,z,t+1/2,u); (x,y,-z,-t+1/2,-u)
Non-lattice operators: (x,y,z,t,u); (-x,-y,z,t+1/2,u); (-x,-y,-z,-t,-u); (x,y,-z,-t+1/2,-u)
Reflection conditions: 00lmn:m=2n

Affine transformation to standard basic space group setting

$S * g(\text{input}) * S^{-1} = g(\text{standard})$,
where g is an augmented matrix for an operation in the superspace group.
Also, S * r(input) = r(standard),
where r is an augmented position vector, (x,y,z,t,u,1).

$$S = \begin{pmatrix} 1&0&0&0&0&0\\ 0&1&0&0&0&0\\ 0&0&1&0&0&0\\ 0&0&0&1&0&0\\ 0&0&0&0&1&0\\ 0&0&0&0&0&1 \end{pmatrix} \quad S^{-1} = \begin{pmatrix} 1&0&0&0&0&0\\ 0&1&0&0&0&0\\ 0&0&1&0&0&0\\ 0&0&0&1&0&0\\ 0&0&0&0&1&0\\ 0&0&0&0&0&1 \end{pmatrix}$$

a1' = a1
a2' = a2
a3' = a3

a1 = a1'
a2 = a2'
a3 = a3'

a1*' = a1*
a2*' = a2*
a3*' = a3*

a1* = a1*'
a2* = a2*'
a3* = a3*'

q1' = q1 = (0,0,g1)
q2' = q2 = (0,0,g2)

q1 = q1' = (0,0,g1)
q2 = q2' = (0,0,g2)

findssg P2/m(0,0,g1)s0(0,0,g2)s0

Input setting

Centering
none
Operators
(-x,-y,z,t+1/2,u+1/2); (-x,-y,-z,-t,-u); (x,y,z,t,u); (x,y,-z,-t+1/2,-u+1/2)

Standard settings

Superspace group: 10.2.5.6 P2/m(0,0,g1)s0(0,0,g2)00 [Y:2.70]
Bravais class: 2.5 P2/m(0,0,g1)(0,0,g2) [JJdW:2.5]
Transformation to supercentered setting: none
Modulation vectors: q1'=(0,0,g1), q2'=(0,0,g2)
Centering: (0,0,0,0,0)
Non-lattice generators: (-x,-y,z,t+1/2,u); (x,y,-z,-t+1/2,-u)
Non-lattice operators: (x,y,z,t,u); (-x,-y,z,t+1/2,u); (-x,-y,-z,-t,-u); (x,y,-z,-t+1/2,-u)
Reflection conditions: 00lmn:m=2n

Affine transformation to standard basic space group setting

S * g(input) * S^{-1} = g(standard),
where g is an augmented matrix for an operation in the superspace group.
Also, S * r(input) = r(standard),
where r is an augmented position vector, (x,y,z,t,u,1).

$$S = \begin{pmatrix} 1&0&0&0&0&0 \\ 0&1&0&0&0&0 \\ 0&0&1&0&0&0 \\ 0&0&0&1&0&0 \\ 0&0&0&1&1&0 \\ 0&0&0&0&0&1 \end{pmatrix} \quad S^{-1} = \begin{pmatrix} 1&0&0&0&0&0 \\ 0&1&0&0&0&0 \\ 0&0&1&0&0&0 \\ 0&0&0&1&0&0 \\ 0&0&0&-1&1&0 \\ 0&0&0&0&0&1 \end{pmatrix}$$

a1' = a1
a2' = a2
a3' = a3

a1 = a1'
a2 = a2'
a3 = a3'

a1*' = a1*
a2*' = a2*
a3*' = a3*

a1* = a1*'
a2* = a2*'
a3* = a3*'

q1' = q1 = (0,0,g1)
q2' = q1 + q2 = (0,0,g2)

q1 = q1' = (0,0,g1)
q2 = -q1' + q2' = (0,0,-g1+g2)
