# Supplementary material for: Equivalence of superspace groups
Source: Acta Crystallogr A. 2012 Nov 14;69(Pt 1):75–90. doi: 10.1107/S0108767312041657 (PMC3553647; doi:10.1107/S0108767312041657)
Supplement: Supplementary file 1 [file a-69-00075-sup1.zip › ssg2d_p3m1_p00_sc2bc3.pdf]

164.2.82.4 P-3m1(a,0,0)000(-a,a,0)000

Superspace group: 164.2.82.4 P-3m1(a,0,0)000(-a,a,0)000 [Y:2.3263]
Bravais class: 2.82 P6/mmm(a,0,0)(-a,a,0) [JJdW:2.82]
Transformation to supercentered setting: none
Modulation vectors: q1=(a,0,0), q2=(-a,a,0)
Centering: (0,0,0,0,0)
Non-lattice generators: (y,-x+y,-z,t+u,-t); (x,x-y,z,t,-t-u); (x,y,z,t,u)
Non-lattice operators: (x,y,z,t,u); (-y,x-y,z,-t-u,t); (-x+y,-x,z,u,-t-u); (x-y,-y,-z,-u,-t); (y,x,-z,t+u,-u); (-x,-x+y,-z,-t,t+u); (-x,-y,-z,-t,-u); (y,-x+y,-z,t+u,-t); (x-y,x,-z,-u,t+u); (-x+y,y,z,u,t); (-y,-x,z,-t-u,u); (x,x-y,z,t,-t-u)
Reflection conditions: none

SSG of $Sc_2B_{1.1}C_{3.2}$
M. Onodo, Y. Shi, a. Leithe-Jasper and T. Tanaka, Acta Crystallogr. B 57, 449-457 (2001).
Onodo et al give P-3m1(p 0 0)(0 p 0)0m0
which is wrong, because (p 0 0) and (0 p 0) are not related by any of the symmetry operators of -3m1. The harmonic of (0 p 0) can be used, because (0 –p 0) does belong to the star of (p 0 0).
There is just one SSG of this kind (no extra groups with different intrinsic translations along the superspace dimensions).
There is no supercentered setting.

-----

findssg P-3m1(a,0,0)000(-a,a,0)000

Operators of the BSG setting have been entered into findssg.

Input setting

Centering
none
Operators
(y,-x+y,-z,t+u,-t); (x,x-y,z,t,-t-u); (x,y,z,t,u); (-x+y,-x,z,u,-t-u); (x-y,-y,-z,-u,-t); (y,x,-z,t+u,-u); (-x+y,y,z,u,t); (x-y,x,-z,-u,t+u); (-x,-y,-z,-t,-u); (-y,-x,z,-t-u,u); (-x,-x+y,-z,-t,t+u); (-y,x-y,z,-t-u,t)

Standard settings

Superspace group: 164.2.82.4 P-3m1(a,0,0)000(-a,a,0)000 [Y:2.3263]
Bravais class: 2.82 P6/mmm(a,0,0)(-a,a,0) [JJdW:2.82]
Transformation to supercentered setting: none
Modulation vectors: q1'=(a,0,0), q2'=(-a,a,0)
Centering: (0,0,0,0,0)
Non-lattice generators: (y,-x+y,-z,t+u,-t); (x,x-y,z,t,-t-u); (x,y,z,t,u)
Non-lattice operators: (x,y,z,t,u); (-y,x-y,z,-t-u,t); (-x+y,-x,z,u,-t-u); (x-y,-y,-z,-u,-t); (y,x,-z,t+u,-u); (-x,-x+y,-z,-t,t+u); (-x,-y,-z,-t,-u); (y,-x+y,-z,t+u,-t); (x-y,x,-z,-u,t+u); (-x+y,y,z,u,t); (-y,-x,z,-t-u,u); (x,x-y,z,t,-t-u)
Reflection conditions: none

Affine transformation to standard basic space group setting

S * g(input) * S $^{-1}$ = g(standard),
where g is an augmented matrix for an operation in the superspace group.
Also, S * r(input) = r(standard),
where r is an augmented position vector, (x,y,z,t,u,1).

$$S = \begin{matrix} 1 & 0 & 0 & 0 & 0 & 0 \\ 0 & 1 & 0 & 0 & 0 & 0 \\ 0 & 0 & 1 & 0 & 0 & 0 \\ 0 & 0 & 0 & 1 & 0 & 0 \\ 0 & 0 & 0 & 0 & 1 & 0 \\ 0 & 0 & 0 & 0 & 0 & 1 \end{matrix} \qquad S^{-1} = \begin{matrix} 1 & 0 & 0 & 0 & 0 & 0 \\ 0 & 1 & 0 & 0 & 0 & 0 \\ 0 & 0 & 1 & 0 & 0 & 0 \\ 0 & 0 & 0 & 1 & 0 & 0 \\ 0 & 0 & 0 & 0 & 1 & 0 \\ 0 & 0 & 0 & 0 & 0 & 1 \end{matrix}$$

a1' = a1
a2' = a2
a3' = a3

a1 = a1'
a2 = a2'
a3 = a3'

a1*' = a1*
a2*' = a2*
a3*' = a3*

a1* = a1*'
a2* = a2*'
a3* = a3*'

q1' = q1 = (a,0,0)
q2' = q2 = (-a,a,0)

q1 = q1' = (a,0,0)
q2 = q2' = (-a,a,0)
