# Supplementary material for: Equivalence of superspace groups
Source: Acta Crystallogr A. 2012 Nov 14;69(Pt 1):75–90. doi: 10.1107/S0108767312041657 (PMC3553647; doi:10.1107/S0108767312041657)
Supplement: Supplementary file 1 [file a-69-00075-sup1.zip › ssg2d_p3_ab13_mx2.pdf]

147.2.72.1 P-3(a,b,1/3)0(-a-b,a,1/3)0

Superspace group: 147.2.72.1 P-3(a,b,1/3)0(-a-b,a,1/3)0 [Y:2.3183]
Bravais class: 2.72 P-3(a,b,1/3)(-a-b,a,1/3) [JJdW:2.72]
Transformation to supercentered setting: A1=a1, A2=a2, A3=3a3+a4+a5, A4=a4, A5=a5

BASIC SPACE GROUP SETTING
Modulation vectors: q1=(a,b,1/3), q2=(-a-b,a,1/3)
Centering: (0,0,0,0,0)
Non-lattice generators: (y,-x+y,-z,-z+t+u,-t)
Non-lattice operators: (x,y,z,t,u); (-y,x-y,z,z-t-u,t); (-x+y,-x,z,u,z-t-u); (-x,-y,-z,-t,-u); (y,-x+y,-z,-z+t+u,-t); (x-y,x,-z,-u,-z+t+u)

SUPERCENTERED SETTING
Modulation vectors: Q1=(A,B,0), Q2=(-A-B,A,0), where A=a, B=b
Centering: (0,0,0,0,0); (0,0,1/3,2/3,2/3); (0,0,2/3,1/3,1/3)
Non-lattice generators: (Y,-X+Y,-Z,T+U,-T)
Non-lattice operators: (X,Y,Z,T,U); (-Y,X-Y,Z,-T-U,T); (-X+Y,-X,Z,U,-T-U); (-X,-Y,-Z,-T,-U); (Y,-X+Y,-Z,T+U,-T); (X-Y,X,-Z,-U,T+U)
Reflection conditions: HKLMN:L-M-N=3n

This is the symmetry of the NC phase of 1T-TaS$_2$ CDW compound.
Notice that A. Spijkerman, J. L. De Boer, A. Meetsma, G. A. Wiegers and S. van Smaalen, Phys. Rev. B 56, 13757-13767 (1997)
give the wrong q2 and thus get into a left-handed coordinate system for x4,x5.

and the corresponding acentric SSG 143.2.72.1:

Superspace group: 143.2.72.1 P3(a,b,1/3)0(-a-b,a,1/3)0 [Y:2.3177]
Bravais class: 2.72 P-3(a,b,1/3)(-a-b,a,1/3) [JJdW:2.72]
Transformation to supercentered setting: A1=a1, A2=a2, A3=3a3+a4+a5, A4=a4, A5=a5

BASIC SPACE GROUP SETTING
Modulation vectors: q1=(a,b,1/3), q2=(-a-b,a,1/3)
Centering: (0,0,0,0,0)
Non-lattice generators: (-y,x-y,z,z-t-u,t)
Non-lattice operators: (x,y,z,t,u); (-y,x-y,z,z-t-u,t); (-x+y,-x,z,u,z-t-u)

SUPERCENTERED SETTING
Modulation vectors: Q1=(A,B,0), Q2=(-A-B,A,0), where A=a, B=b
Centering: (0,0,0,0,0); (0,0,1/3,2/3,2/3); (0,0,2/3,1/3,1/3)
Non-lattice generators: (-Y,X-Y,Z,-T-U,T)
Non-lattice operators: (X,Y,Z,T,U); (-Y,X-Y,Z,-T-U,T); (-X+Y,-X,Z,U,-T-U)
Reflection conditions: HKLMN:L-M-N=3n
-----

findssg P-3(a,b,1/3)0(-a-b,a,1/3)0

Generator of the standard BSG setting is given to findssg.

Input setting

Centering
none
Operators
(y,-x+y,-z,-z+t+u,-t); (-x+y,-x,z,u,z-t-u); (-x,-y,-z,-t,-u); (-y,x-y,z,z-t-u,t); (x-y,x,-z,-u,-z+t+u); (x,y,z,t,u)

Standard settings

Superspace group: 147.2.72.1 P-3(a,b,1/3)0(-a-b,a,1/3)0 [Y:2.3183]
Bravais class: 2.72 P-3(a,b,1/3)(-a-b,a,1/3) [JJdW:2.72]
Transformation to supercentered setting: A1=a1, A2=a2, A3=3a3+a4+a5, A4=a4, A5=a5

BASIC SPACE GROUP SETTING
Modulation vectors: q1'=(a,b,1/3), q2'=(-a-b,a,1/3)
Centering: (0,0,0,0,0)
Non-lattice generators: (y,-x+y,-z,-z+t+u,-t)
Non-lattice operators: (x,y,z,t,u); (-y,x-y,z,z-t-u,t); (-x+y,-x,z,u,z-t-u); (-x,-y,-z,-t,-u); (y,-x+y,-z,-z+t+u,-t); (x-y,x,-z,-u,-z+t+u)

SUPERCENTERED SETTING
Modulation vectors: Q1'=(A,B,0), Q2'=(-A-B,A,0), where A=a, B=b
Centering: (0,0,0,0,0); (0,0,1/3,2/3,2/3); (0,0,2/3,1/3,1/3)
Non-lattice generators: (Y,-X+Y,-Z,T+U,-T)
Non-lattice operators: (X,Y,Z,T,U); (-Y,X-Y,Z,-T-U,T); (-X+Y,-X,Z,U,-T-U); (-X,-Y,-Z,-T,-U); (Y,-X+Y,-Z,T+U,-T); (X-Y,X,-Z,-U,T+U)
Reflection conditions: HKLMN:L-M-N=3n

Affine transformation to standard basic space group setting

S * g(input) * S $^{-1}$ = g(standard),
where g is an augmented matrix for an operation in the superspace group.
Also, S * r(input) = r(standard),
where r is an augmented position vector, (x,y,z,t,u,1).

$$S = \begin{pmatrix} 1&0&0&0&0&0\\ 0&1&0&0&0&0\\ 0&0&1&0&0&0\\ 0&0&0&1&0&0\\ 0&0&0&0&1&0\\ 0&0&0&0&0&1 \end{pmatrix} \quad S^{-1} = \begin{pmatrix} 1&0&0&0&0&0\\ 0&1&0&0&0&0\\ 0&0&1&0&0&0\\ 0&0&0&1&0&0\\ 0&0&0&0&1&0\\ 0&0&0&0&0&1 \end{pmatrix}$$

a1' = a1
a2' = a2
a3' = a3

a1 = a1'
a2 = a2'
a3 = a3'

a1*' = a1*
a2*' = a2*
a3*' = a3*

a1* = a1*'
a2* = a2*'
a3* = a3*'

q1' = q1 = (a,b,1/3)
q2' = q2 = (-a-b,a,1/3)

q1 = q1' = (a,b,1/3)
q2 = q2' = (-a-b,a,1/3)

findssg X-3(a,b,0)0(-a-b,a,0)0

Generators of the standard supercentered setting are given to findssg.

findssg

Input setting

Centering
(0,0,0,0,0); (0,0,1/3,2/3,2/3); (0,0,2/3,1/3,1/3)
Operators
(y,-x+y,-z,t+u,-t); (-x+y,-x,z,u,-t-u); (-x,-y,-z,-t,-u); (-y,x-y,z,-t-u,t); (x-y,x,-z,-u,t+u); (x,y,z,t,u)

Standard settings

Superspace group: 147.2.72.1 P-3(a,b,1/3)0(-a-b,a,1/3)0 [Y:2.3183]
Bravais class: 2.72 P-3(a,b,1/3)(-a-b,a,1/3) [JJdW:2.72]
Transformation to supercentered setting: A1=a1, A2=a2, A3=3a3+a4+a5, A4=a4, A5=a5

BASIC SPACE GROUP SETTING
Modulation vectors: q1'=(a,b,1/3), q2'=(-a-b,a,1/3)
Centering: (0,0,0,0,0)
Non-lattice generators: (y,-x+y,-z,-z+t+u,-t)
Non-lattice operators: (x,y,z,t,u); (-y,x-y,z,z-t-u,t); (-x+y,-x,z,u,z-t-u); (-x,-y,-z,-t,-u); (y,-x+y,-z,-z+t+u,-t); (x-y,x,-z,-u,-z+t+u)

SUPERCENTERED SETTING
Modulation vectors: Q1'=(A,B,0), Q2'=(-A-B,A,0), where A=a, B=b
Centering: (0,0,0,0,0); (0,0,1/3,2/3,2/3); (0,0,2/3,1/3,1/3)
Non-lattice generators: (Y,-X+Y,-Z,T+U,-T)
Non-lattice operators: (X,Y,Z,T,U); (-Y,X-Y,Z,-T-U,T); (-X+Y,-X,Z,U,-T-U); (-X,-Y,-Z,-T,-U); (Y,-X+Y,-Z,T+U,-T); (X-Y,X,-Z,-U,T+U)
Reflection conditions: HKLMN:L-M-N=3n

Affine transformation to standard basic space group setting

$S * g(input) * S^{-1} = g(standard)$,
where g is an augmented matrix for an operation in the superspace group.
Also, S * r(input) = r(standard),
where r is an augmented position vector, (x,y,z,t,u,1).

$$S = \begin{matrix} 1 & 0 & 0 & 0 & 0 & 0 \\ 0 & 1 & 0 & 0 & 0 & 0 \\ 0 & 0 & 3 & 0 & 0 & 0 \\ 0 & 0 & 1 & 1 & 0 & 0 \\ 0 & 0 & 1 & 0 & 1 & 0 \\ 0 & 0 & 0 & 0 & 0 & 1 \end{matrix} \qquad S^{-1} = \begin{matrix} 1 & 0 & 0 & 0 & 0 & 0 \\ 0 & 1 & 0 & 0 & 0 & 0 \\ 0 & 0 & 1/3 & 0 & 0 & 0 \\ 0 & 0 & -1/3 & 1 & 0 & 0 \\ 0 & 0 & -1/3 & 0 & 1 & 0 \\ 0 & 0 & 0 & 0 & 0 & 1 \end{matrix}$$

a1' = a1
a2' = a2
a3' = 1/3 a3

a1 = a1'
a2 = a2'
a3 = 3 a3'

a1*' = a1*
a2*' = a2*
a3*' = 3 a3*

a1* = a1*'
a2* = a2*'
a3* = 1/3 a3*'

q1' = q1 + a3* = (a,b,1/3)
q2' = q2 + a3* = (-a-b,a,1/3)

q1 = q1' - 1/3 a3*' = (a,b,0)
q2 = q2' - 1/3 a3*' = (-a-b,a,0)

findssg X-3(a,b,0)0(-a-b,a,0)0

Generators of the alternate supercentered setting of Spijkerman et al. (1997) are given to findssg.

Input setting

Centering
(0,0,0,0,0); (0,0,1/3,2/3,1/3); (0,0,2/3,1/3,2/3)
Operators
(y,-x+y,-z,u,-t+u); (-x+y,-x,z,-t+u,-t); (-x,-y,-z,-t,-u); (-y,x-y,z,-u,t-u); (x-y,x,-z,t-u,t); (x,y,z,t,u)

Standard settings

Superspace group: 147.2.72.1 P-3(a,b,1/3)0(-a-b,a,1/3)0 [Y:2.3183]
Bravais class: 2.72 P-3(a,b,1/3)(-a-b,a,1/3) [JJdW:2.72]
Transformation to supercentered setting: A1=a1, A2=a2, A3=3a3+a4+a5, A4=a4, A5=a5

BASIC SPACE GROUP SETTING
Modulation vectors: q1'=(a,b,1/3), q2'=(-a-b,a,1/3)
Centering: (0,0,0,0,0)
Non-lattice generators: (y,-x+y,-z,-z+t+u,-t)
Non-lattice operators: (x,y,z,t,u); (-y,x-y,z,z-t-u,t); (-x+y,-x,z,u,z-t-u); (-x,-y,-z,-t,-u); (y,-x+y,-z,-z+t+u,-t); (x-y,x,-z,-u,-z+t+u)

SUPERCENTERED SETTING
Modulation vectors: Q1'=(A,B,0), Q2'=(-A-B,A,0), where A=a, B=b
Centering: (0,0,0,0,0); (0,0,1/3,2/3,2/3); (0,0,2/3,1/3,1/3)
Non-lattice generators: (Y,-X+Y,-Z,T+U,-T)
Non-lattice operators: (X,Y,Z,T,U); (-Y,X-Y,Z,-T-U,T); (-X+Y,-X,Z,U,-T-U); (-X,-Y,-Z,-T,-U); (Y,-X+Y,-Z,T+U,-T); (X-Y,X,-Z,-U,T+U)
Reflection conditions: HKLMN:L-M-N=3n

Affine transformation to standard basic space group setting

S * g(input) * S $^{-1}$ = g(standard),
where g is an augmented matrix for an operation in the superspace group.
Also, S * r(input) = r(standard),
where r is an augmented position vector, (x,y,z,t,u,1).

$$S = \begin{matrix} 1 & 0 & 0 & 0 & 0 & 0 \\ 0 & 1 & 0 & 0 & 0 & 0 \\ 0 & 0 & 3 & 0 & 0 & 0 \\ 0 & 0 & 1 & 0 & -1 & 0 \\ 0 & 0 & 1 & 1 & 0 & 0 \\ 0 & 0 & 0 & 0 & 0 & 1 \end{matrix} \qquad S^{-1} = \begin{matrix} 1 & 0 & 0 & 0 & 0 & 0 \\ 0 & 1 & 0 & 0 & 0 & 0 \\ 0 & 0 & 1/3 & 0 & 0 & 0 \\ 0 & 0 & -1/3 & 0 & 1 & 0 \\ 0 & 0 & 1/3 & -1 & 0 & 0 \\ 0 & 0 & 0 & 0 & 0 & 1 \end{matrix}$$

a1' = a1
a2' = a2
a3' = 1/3 a3

a1 = a1'
a2 = a2'
a3 = 3 a3'

a1*' = a1*
a2*' = a2*
a3*' = 3 a3*

a1* = a1*'
a2* = a2*'
a3* = 1/3 a3*'

q1' = -q2 + a3* = (a,b,1/3)
q2' = q1 + a3* = (-a-b,a,1/3)

q1 = q2' - 1/3 a3*' = (-a-b,a,0)
q2 = -q1' + 1/3 a3*' = (-a,-b,0)

From previous version of findssg :
Affine transformation to standard supercentered setting

$T * g(\text{input}) * T^{-1} = g(\text{standard})$,
where g is an augmented matrix for an operation in the superspace group.
Also, $T * r(\text{input}) = r(\text{standard})$,
where r is an augmented position vector, (x,y,z,t,u,1) or (X,Y,Z,T,U,1).

$$T = \begin{matrix} 1 & 0 & 0 & 0 & 0 & 0 \\ 0 & 1 & 0 & 0 & 0 & 0 \\ 0 & 0 & 1 & 0 & 0 & 0 \\ 0 & 0 & 0 & 1 & 0 & 0 \\ 0 & 0 & 0 & -1 & 1 & 0 \\ 0 & 0 & 0 & 0 & 0 & 1 \end{matrix} \qquad T^{-1} = \begin{matrix} 1 & 0 & 0 & 0 & 0 & 0 \\ 0 & 1 & 0 & 0 & 0 & 0 \\ 0 & 0 & 1 & 0 & 0 & 0 \\ 0 & 0 & 0 & 1 & 0 & 0 \\ 0 & 0 & 0 & 1 & 1 & 0 \\ 0 & 0 & 0 & 0 & 0 & 1 \end{matrix}$$
