# Supplementary material for: Equivalence of superspace groups
Source: Acta Crystallogr A. 2012 Nov 14;69(Pt 1):75–90. doi: 10.1107/S0108767312041657 (PMC3553647; doi:10.1107/S0108767312041657)
Supplement: Supplementary file 1 [file a-69-00075-sup1.zip › ssg2d_p421m_aa0_akermanite_mellite.pdf]

113.2.68.6 P-42_1m(a,a,0)00s(-a,a,0)000

Superspace group: 113.2.68.6 P-42_1m(a,a,0)00s(-a,a,0)000 [Y:2.2833]
Bravais class: 2.68 P4/mmm(a,a,0)(-a,a,0) [JJdW:2.68]
Transformation to supercentered setting: none
Modulation vectors: q1=(a,a,0), q2=(-a,a,0)
Centering: (0,0,0,0,0)
Non-lattice generators: (y,-x,-z,u,-t); (x+1/2,-y+1/2,-z,-u+1/2,-t+1/2); (y+1/2,x-1/2,z,t+1/2,-u+1/2)
Non-lattice operators: (x,y,z,t,u); (x+1/2,-y+1/2,-z,-u+1/2,-t+1/2); (-x+1/2,y+1/2,-z,u+1/2,t+1/2); (-x,-y,z,-t,-u); (y+1/2,x+1/2,z,t+1/2,-u+1/2); (y,-x,-z,u,-t); (-y,x,-z,-u,t); (-y+1/2,-x+1/2,z,-t+1/2,u+1/2)
Reflection conditions: hhlm0:m=2n; h-hl0n:n=2n; h00m-m:h=2n; 0k0mm:k=2n

-----

There is no supercentered setting, i.e. this is a primitive superspace lattice.
This is the symmetry of Akermanite Ca2CoSi2O7, see [1]
K. Hagiya, M. Ohmasa and K. Iishi, Acta Crystallogr. B 49, 172-179 (1993).
Compounds with the same symmetry and similar crystal structure are
[2] $(Sr_{0.13}Ca_{0.87})_2CoSi_2O_7$: q1 = 0.286 (3)(a* + b*) ; q2 = 0.286 (3)(-a* + b*),
B. Bagautdinov et al., Acta Crystallogr. (2000). B56, 811-821.
[3] (Ca1.89Sr0.01Na0.08K0.02)(Mg0.92Al0.08)-(Si1.98Al0.02)O7 :
P-421m:p4mg ; q1 = 0.2815 (3)(a* + b*), q2 = 0.2815 (3)(-a* + b*).
L. Bindi et al., Acta Crystallogr. B 57, 739-746 (2001).
[4] [CaNd]2[Ga]2[Ga2O7]2 ; P-421m(α,α,0)00s(a,a,0)000.
q1 = 0.2319(2)(a* + b*) and q2 = 0.2319(2)-a* + b*).
F. Wei et al., J. Am. Chem. Soc. 133, 15200 (2011).

There is one other group differing in the intrinsic translational component along the superspace dimensions.

-----

findssg P-42_1m(a,a,0)00s(-a,a,0)000

Generators of the BSG setting have been entered into findssg.

Input setting

Centering
none
Operators
(y,-x,-z,u,-t); (x+1/2,-y+1/2,-z,-u+1/2,-t+1/2); (y+1/2,x+1/2,z,t+1/2,-u+1/2); (-x,-y,z,-t,-u); (-y+1/2,-x+1/2,z,-t+1/2,u+1/2); (x,y,z,t,u); (-x+1/2,y+1/2,-z,u+1/2,t+1/2); (-y,x,-z,-u,t)

Standard settings

Superspace group: 113.2.68.6 P-42_1m(a,a,0)00s(-a,a,0)000 [Y:2.2833]
Bravais class: 2.68 P4/mmm(a,a,0)(-a,a,0) [JJdW:2.68]
Transformation to supercentered setting: none
Modulation vectors: q1'=(a,a,0), q2'=(-a,a,0)
Centering: (0,0,0,0,0)
Non-lattice generators: (y,-x,-z,u,-t); (x+1/2,-y+1/2,-z,-u+1/2,-t+1/2); (y+1/2,x-1/2,z,t+1/2,-u+1/2)
Non-lattice operators: (x,y,z,t,u); (x+1/2,-y+1/2,-z,-u+1/2,-t+1/2); (-x+1/2,y+1/2,-z,u+1/2,t+1/2); (-x,-y,z,-t,-u); (y+1/2,x+1/2,z,t+1/2,-u+1/2); (y,-x,-z,u,-t); (-y,x,-z,-u,t); (-y+1/2,-x+1/2,z,-t+1/2,u+1/2)
Reflection conditions: hhlm0:m=2n; h-hl0n:n=2n; h00m-m:h=2n; 0k0mm:k=2n

Affine transformation to standard basic space group setting

S * g(input) * S $^{-1}$ = g(standard),
where g is an augmented matrix for an operation in the superspace group.
Also, S * r(input) = r(standard),
where r is an augmented position vector, (x,y,z,t,u,1).

$$S = \begin{matrix} 1 & 0 & 0 & 0 & 0 & 0 \\ 0 & 1 & 0 & 0 & 0 & 0 \\ 0 & 0 & 1 & 0 & 0 & 0 \\ 0 & 0 & 0 & 1 & 0 & 0 \\ 0 & 0 & 0 & 0 & 1 & 0 \\ 0 & 0 & 0 & 0 & 0 & 1 \end{matrix} \qquad S^{-1} = \begin{matrix} 1 & 0 & 0 & 0 & 0 & 0 \\ 0 & 1 & 0 & 0 & 0 & 0 \\ 0 & 0 & 1 & 0 & 0 & 0 \\ 0 & 0 & 0 & 1 & 0 & 0 \\ 0 & 0 & 0 & 0 & 1 & 0 \\ 0 & 0 & 0 & 0 & 0 & 1 \end{matrix}$$

a1' = a1
a2' = a2
a3' = a3

a1 = a1'
a2 = a2'

a3 = a3'

a1*' = a1*
a2*' = a2*
a3*' = a3*

a1* = a1*'
a2* = a2*'
a3* = a3*'

q1' = q1 = (a,a,0)

q2' = q2 = (-a,a,0)

q1 = q1' = (a,a,0)
q2 = q2' = (-a,a,0)

findssg P-4$2_1$m:p4mg

Generators of publication Hagiya (1993) have been entered into findssg.

Input setting

Centering
none
Operators
(-y,x,-z,-u,t); (y+1/2,x+1/2,z,t+1/2,-u+1/2); (-x,-y,z,-t,-u); (-x+1/2,y+1/2,-z,u+1/2,t+1/2); (y,-x,-z,u,-t); (-y+1/2,-x+1/2,z,-t+1/2,u+1/2); (x+1/2,-y+1/2,-z,-u+1/2,-t+1/2); (x,y,z,t,u)

Standard settings

Superspace group: 113.2.68.6 P-42_1m(a,a,0)00s(-a,a,0)000 [Y:2.2833]
Bravais class: 2.68 P4/mmm(a,a,0)(-a,a,0) [JJdW:2.68]
Transformation to supercentered setting: none
Modulation vectors: q1'=(a,a,0), q2'=(-a,a,0)
Centering: (0,0,0,0,0)
Non-lattice generators: (y,-x,-z,u,-t); (x+1/2,-y+1/2,-z,-u+1/2,-t+1/2); (y+1/2,x-1/2,z,t+1/2,-u+1/2)
Non-lattice operators: (x,y,z,t,u); (x+1/2,-y+1/2,-z,-u+1/2,-t+1/2); (-x+1/2,y+1/2,-z,u+1/2,t+1/2); (-x,-y,z,-t,-u); (y+1/2,x+1/2,z,t+1/2,-u+1/2); (y,-x,-z,u,-t); (-y,x,-z,-u,t); (-y+1/2,-x+1/2,z,-t+1/2,u+1/2)
Reflection conditions: hhlm0:m=2n; h-hl0n:n=2n; h00m-m:h=2n; 0k0mm:k=2n

Affine transformation to standard basic space group setting

S * g(input) * S $^{-1}$ = g(standard),
where g is an augmented matrix for an operation in the superspace group.
Also, S * r(input) = r(standard),
where r is an augmented position vector, (x,y,z,t,u,1).

$$S = \begin{pmatrix} 1&0&0&0&0&0\\ 0&1&0&0&0&0\\ 0&0&1&0&0&0\\ 0&0&0&1&0&0\\ 0&0&0&0&1&0\\ 0&0&0&0&0&1 \end{pmatrix} \quad S^{-1} = \begin{pmatrix} 1&0&0&0&0&0\\ 0&1&0&0&0&0\\ 0&0&1&0&0&0\\ 0&0&0&1&0&0\\ 0&0&0&0&1&0\\ 0&0&0&0&0&1 \end{pmatrix}$$

a1' = a1		a3* = a3*'
a2' = a2	a1*' = a1*	
a3' = a3	a2*' = a2*	q1' = q1 = (a,a,0)
	a3*' = a3*	q2' = q2 = (-a,a,0)
a1 = a1'		
a2 = a2'	a1* = a1*'	q1 = q1' = (a,a,0)
a3 = a3'	a2* = a2*'	q2 = q2' = (-a,a,0)

113.2.68.5 $P\text{-}42_1m(a,a,0)000(-a,a,0)000$

Superspace group: 113.2.68.5 P-42_1m(a,a,0)000(-a,a,0)000 [Y:2.2831]
Bravais class: 2.68 P4/mmm(a,a,0)(-a,a,0) [JJdW:2.68]
Transformation to supercentered setting: none
Modulation vectors: q1=(a,a,0), q2=(-a,a,0)
Centering: (0,0,0,0,0)
Non-lattice generators: (y,-x,-z,u,-t); (x+1/2,-y+1/2,-z,-u,-t); (y+1/2,x-1/2,z,t,-u)
Non-lattice operators: (x,y,z,t,u); (x+1/2,-y+1/2,-z,-u,-t); (-x+1/2,y+1/2,-z,u,t); (-x,-y,z,-t,-u); (y+1/2,x+1/2,z,t,-u); (y,-x,-z,u,-t); (-y,x,-z,-u,t); (-y+1/2,-x+1/2,z,-t,u)
Reflection conditions: h00m-m:h=2n; 0k0mm:k=2n

There is no supercentered setting, i.e. this is a primitive superspace lattice.

There is one other group differing in the intrinsic translational component along the superspace dimensions.

findssg P-4$2_1$m(a,a,0)000(-a,a,0)000

Generators of standard BSG setting given to findssg.

Input setting

Centering
none
Operators
(y,-x,-z,u,-t); (x+1/2,-y+1/2,-z,-u,-t); (y+1/2,x+1/2,z,t,-u); (-x,-y,z,-t,-u); (-y+1/2,-x+1/2,z,-t,u); (x,y,z,t,u); (-x+1/2,y+1/2,-z,u,t); (-y,x,-z,-u,t)

Standard settings

Superspace group: 113.2.68.5 P-42_1m(a,a,0)000(-a,a,0)000 [Y:2.2831]
Bravais class: 2.68 P4/mmm(a,a,0)(-a,a,0) [JJdW:2.68]
Transformation to supercentered setting: none
Modulation vectors: q1'=(a,a,0), q2'=(-a,a,0)
Centering: (0,0,0,0,0)
Non-lattice generators: (y,-x,-z,u,-t); (x+1/2,-y+1/2,-z,-u,-t); (y+1/2,x-1/2,z,t,-u)
Non-lattice operators: (x,y,z,t,u); (x+1/2,-y+1/2,-z,-u,-t); (-x+1/2,y+1/2,-z,u,t); (-x,-y,z,-t,-u); (y+1/2,x+1/2,z,t,-u); (y,-x,-z,u,-t); (-y,x,-z,-u,t); (-y+1/2,-x+1/2,z,-t,u)
Reflection conditions: h00m-m:h=2n; 0k0mm:k=2n

Affine transformation to standard basic space group setting

$S * g(input) * S^{-1} = g(standard)$,
where g is an augmented matrix for an operation in the superspace group.
Also, S * r(input) = r(standard),
where r is an augmented position vector, (x,y,z,t,u,1).

$$S = \begin{matrix} 1&0&0&0&0&0 \\ 0&1&0&0&0&0 \\ 0&0&1&0&0&0 \\ 0&0&0&1&0&0 \\ 0&0&0&0&1&0 \\ 0&0&0&0&0&1 \end{matrix} \quad S^{-1} = \begin{matrix} 1&0&0&0&0&0 \\ 0&1&0&0&0&0 \\ 0&0&1&0&0&0 \\ 0&0&0&1&0&0 \\ 0&0&0&0&1&0 \\ 0&0&0&0&0&1 \end{matrix}$$

a1' = a1		a3* = a3*'
a2' = a2	a1*' = a1*	
a3' = a3	a2*' = a2*	q1' = q1 = (a,a,0)
	a3*' = a3*	q2' = q2 = (-a,a,0)
a1 = a1'		
a2 = a2'	a1* = a1*'	q1 = q1' = (a,a,0)
a3 = a3'	a2* = a2*'	q2 = q2' = (-a,a,0)
