# Supplementary material for: Equivalence of superspace groups
Source: Acta Crystallogr A. 2012 Nov 14;69(Pt 1):75–90. doi: 10.1107/S0108767312041657 (PMC3553647; doi:10.1107/S0108767312041657)
Supplement: Supplementary file 1 [file a-69-00075-sup1.zip › ssg2d_p4bm_aa12_sr2tisi2o8.pdf]

100.2.69.13 P4bm(a,a,1/2)000(-a,a,1/2)000

Superspace group: 100.2.69.13 P4bm(a,a,1/2)000(-a,a,1/2)000 [Y:2.2765]
Bravais class: 2.69 P4/mmm(a,a,1/2)(-a,a,1/2) [JJdW:2.69]
Transformation to supercentered setting: A1=a1, A2=a2, A3=2a3+a4+a5, A4=a4, A5=a5

BASIC SPACE GROUP SETTING
Modulation vectors: q1=(a,a,1/2), q2=(-a,a,1/2)
Centering: (0,0,0,0,0)
Non-lattice generators: (-y,x,z,z-u,t); (-x+1/2,y+1/2,z,u,t); (y+1/2,x-1/2,z,t,z-u)
Non-lattice operators: (x,y,z,t,u); (-x,-y,z,z-t,z-u); (-y,x,z,z-u,t); (y,-x,z,u,z-t); (-x+1/2,y+1/2,z,u,t); (x+1/2,-y+1/2,z,z-u,z-t); (y+1/2,x+1/2,z,t,z-u); (-y+1/2,-x+1/2,z,z-t,u)

SUPERCENTERED SETTING
Modulation vectors: Q1=(A,A,0), Q2=(-A,A,0), where A=a
Centering: (0,0,0,0,0); (0,0,1/2,1/2,1/2)
Non-lattice generators: (-Y,X,Z,-U,T); (-X+1/2,Y+1/2,Z,U,T); (Y+1/2,X-1/2,Z,T,-U)
Non-lattice operators: (X,Y,Z,T,U); (-X,-Y,Z,-T,-U); (-Y,X,Z,-U,T); (Y,-X,Z,U,-T); (-X+1/2,Y+1/2,Z,U,T); (X+1/2,-Y+1/2,Z,-U,-T); (Y+1/2,X+1/2,Z,T,-U); (-Y+1/2,-X+1/2,Z,-T,U)
Reflection conditions: HKLMN:L+M+N=2n; H0LM-M:H=2n; 0KLMM:K=2n

-----

One other SSG exists, with different intrinsic translational components:
100.2.69.14 P4bm(a,a,1/2)00s(-a,a,1/2)000 [Y:2.2766].

This is the SSG of Ba0.39Sr0.61Nb2O6, see: Woike, V. Petricek, M. Dusek, N. K. Hansen, P. Fertey, C. Lecomte, A. Arakcheeva, G. Chapuis, M. Imlaue and R. Pankrathe Acta Crystallogr. B 59, 28 (2003).

-----

findssg P4bm(a,a,1/2)000(-a,a,1/2)000

Generators of the standard BSG setting are entered into findssg.

Input setting

Centering
none
Operators
(-y,x,z,z-u,t); (-x+1/2,y+1/2,z,u,t); (y+1/2,x+1/2,z,t,z-u); (-x,-y,z,z-t,z-u); (-y+1/2,-x+1/2,z,z-t,u); (x,y,z,t,u); (x+1/2,-y+1/2,z,z-u,z-t); (y,-x,z,u,z-t)

Standard settings

Superspace group: 100.2.69.13 P4bm(a,a,1/2)000(-a,a,1/2)000 [Y:2.2765]
Bravais class: 2.69 P4/mmm(a,a,1/2)(-a,a,1/2) [JJdW:2.69]
Transformation to supercentered setting: A1=a1, A2=a2, A3=2a3+a4+a5, A4=a4, A5=a5

BASIC SPACE GROUP SETTING
Modulation vectors: q1'=(a,a,1/2), q2'=(-a,a,1/2)
Centering: (0,0,0,0,0)
Non-lattice generators: (-y,x,z,z-u,t); (-x+1/2,y+1/2,z,u,t); (y+1/2,x-1/2,z,t,z-u)
Non-lattice operators: (x,y,z,t,u); (-x,-y,z,z-t,z-u); (-y,x,z,z-u,t); (y,-x,z,u,z-t); (-x+1/2,y+1/2,z,u,t); (x+1/2,-y+1/2,z,z-u,z-t); (y+1/2,x+1/2,z,t,z-u); (-y+1/2,-x+1/2,z,z-t,u)

SUPERCENTERED SETTING
Modulation vectors: Q1'=(A,A,0), Q2'=(-A,A,0), where A=a
Centering: (0,0,0,0,0); (0,0,1/2,1/2,1/2)
Non-lattice generators: (-Y,X,Z,-U,T); (-X+1/2,Y+1/2,Z,U,T); (Y+1/2,X-1/2,Z,T,-U)
Non-lattice operators: (X,Y,Z,T,U); (-X,-Y,Z,-T,-U); (-Y,X,Z,-U,T); (Y,-X,Z,U,-T); (-X+1/2,Y+1/2,Z,U,T); (X+1/2,-Y+1/2,Z,-U,-T); (Y+1/2,X+1/2,Z,T,-U); (-Y+1/2,-X+1/2,Z,-T,U)
Reflection conditions: HKLMN:L+M+N=2n; H0LM-M:H=2n; 0KLMM:K=2n

Affine transformation to standard basic space group setting

S * g(input) * S $^{-1}$ = g(standard),
where g is an augmented matrix for an operation in the superspace group.
Also, S * r(input) = r(standard),
where r is an augmented position vector, (x,y,z,t,u,1).

$$S = \begin{pmatrix} 1&0&0&0&0&0\\ 0&1&0&0&0&0\\ 0&0&1&0&0&0\\ 0&0&0&1&0&0\\ 0&0&0&0&1&0\\ 0&0&0&0&0&1 \end{pmatrix} \quad S^{-1} = \begin{pmatrix} 1&0&0&0&0&0\\ 0&1&0&0&0&0\\ 0&0&1&0&0&0\\ 0&0&0&1&0&0\\ 0&0&0&0&1&0\\ 0&0&0&0&0&1 \end{pmatrix}$$

a1' = a1
a2' = a2
a3' = a3

a1 = a1'
a2 = a2'
a3 = a3'

a1*' = a1*
a2*' = a2*
a3*' = a3*

a1* = a1*'
a2* = a2*'
a3* = a3*'

q1' = q1 = (a,a,1/2)
q2' = q2 = (-a,a,1/2)

q1 = q1' = (a,a,1/2)
q2 = q2' = (-a,a,1/2)

findssg P4bm(p p 1/2, p –p 1/2)000

Generators according to Woike Ba0.39Sr0.61Nb2O6 have been entered into findssg.

Input setting

Centering
none
Operators
(-y,x,z,z+u,-t); (-x+1/2,y+1/2,z,-u,-t); (-x,-y,z,z-t,-z-u); (-y+1/2,-x+1/2,z,z-t,u); (y,-x,z,-u,-z+t); (y+1/2,x+1/2,z,t,-z-u); (x,y,z,t,u); (x+1/2,-y+1/2,z,z+u,-z+t)

Standard settings

Superspace group: 100.2.69.13 P4bm(a,a,1/2)000(-a,a,1/2)000 [Y:2.2765]
Bravais class: 2.69 P4/mmm(a,a,1/2)(-a,a,1/2) [JJdW:2.69]
Transformation to supercentered setting: A1=a1, A2=a2, A3=2a3+a4+a5, A4=a4, A5=a5

BASIC SPACE GROUP SETTING
Modulation vectors: q1'=(a,a,1/2), q2'=(-a,a,1/2)
Centering: (0,0,0,0,0)
Non-lattice generators: (-y,x,z,z-u,t); (-x+1/2,y+1/2,z,u,t); (y+1/2,x-1/2,z,t,z-u)
Non-lattice operators: (x,y,z,t,u); (-x,-y,z,z-t,z-u); (-y,x,z,z-u,t); (y,-x,z,u,z-t); (-x+1/2,y+1/2,z,u,t); (x+1/2,-y+1/2,z,z-u,z-t); (y+1/2,x+1/2,z,t,z-u); (-y+1/2,-x+1/2,z,z-t,u)

SUPERCENTERED SETTING
Modulation vectors: Q1'=(A,A,0), Q2'=(-A,A,0), where A=a
Centering: (0,0,0,0,0); (0,0,1/2,1/2,1/2)
Non-lattice generators: (-Y,X,Z,-U,T); (-X+1/2,Y+1/2,Z,U,T); (Y+1/2,X-1/2,Z,T,-U)
Non-lattice operators: (X,Y,Z,T,U); (-X,-Y,Z,-T,-U); (-Y,X,Z,-U,T); (Y,-X,Z,U,-T); (-X+1/2,Y+1/2,Z,U,T); (X+1/2,-Y+1/2,Z,-U,-T); (Y+1/2,X+1/2,Z,T,-U); (-Y+1/2,-X+1/2,Z,-T,U)
Reflection conditions: HKLMN:L+M+N=2n; H0LM-M:H=2n; 0KLMM:K=2n

Affine transformation to standard basic space group setting

S * g(input) * S $^{-1}$ = g(standard),
where g is an augmented matrix for an operation in the superspace group.
Also, S * r(input) = r(standard),
where r is an augmented position vector, (x,y,z,t,u,1).

$$S = \begin{pmatrix} 1 & 0 & 0 & 0 & 0 & 0 \\ 0 & 1 & 0 & 0 & 0 & 0 \\ 0 & 0 & 1 & 0 & 0 & 0 \\ 0 & 0 & 0 & 1 & 0 & 0 \\ 0 & 0 & 0 & 0 & -1 & 0 \\ 0 & 0 & 0 & 0 & 0 & 1 \end{pmatrix} \quad S^{-1} = \begin{pmatrix} 1 & 0 & 0 & 0 & 0 & 0 \\ 0 & 1 & 0 & 0 & 0 & 0 \\ 0 & 0 & 1 & 0 & 0 & 0 \\ 0 & 0 & 0 & 1 & 0 & 0 \\ 0 & 0 & 0 & 0 & -1 & 0 \\ 0 & 0 & 0 & 0 & 0 & 1 \end{pmatrix}$$

a1' = a1
a2' = a2
a3' = a3

a1 = a1'
a2 = a2'
a3 = a3'

a1*' = a1*
a2*' = a2*
a3*' = a3*

a1* = a1*'
a2* = a2*'
a3* = a3*'

q1' = q1 = (a,a,1/2)
q2' = -q2 = (-a,a,1/2)

q1 = q1' = (a,a,1/2)
q2 = -q2' = (a,-a,-1/2)

Output of previous version of findssg:
Affine transformation to standard supercentered setting

$T * g(\text{input}) * T^{-1} = g(\text{standard})$,
where g is an augmented matrix for an operation in the superspace group.
Also, T * r(input) = r(standard),
where r is an augmented position vector, (x,y,z,t,u,1) or (X,Y,Z,T,U,1).

$$T = \begin{pmatrix} 1 & 0 & 0 & 0 & 0 & 0 \\ 0 & 1 & 0 & 0 & 0 & 0 \\ 0 & 0 & 1/2 & 0 & 0 & 0 \\ 0 & 0 & 1/2 & -1 & 0 & 0 \\ 0 & 0 & 1/2 & 0 & 1 & 0 \\ 0 & 0 & 0 & 0 & 0 & 1 \end{pmatrix} \quad T^{-1} = \begin{pmatrix} 1 & 0 & 0 & 0 & 0 & 0 \\ 0 & 1 & 0 & 0 & 0 & 0 \\ 0 & 0 & 2 & 0 & 0 & 0 \\ 0 & 0 & 1 & -1 & 0 & 0 \\ 0 & 0 & -1 & 0 & 1 & 0 \\ 0 & 0 & 0 & 0 & 0 & 1 \end{pmatrix}$$

findssg   Y:2.2765 P4bm(-pp1/2,pp1/2)0mm

Operators of Yamamoto entered into findssg. Notice that Yamamoto provides the operators in the supercentered setting.

Input setting

Centering
(0,0,0,0,0); (0,0,1/2,1/2,1/2)
Operators
(-x,-y,z,-t,-u); (-y,x,z,u,-t); (y,-x,z,-u,t); (x+1/2,-y+1/2,z,-u,-t); (-x+1/2,y+1/2,z,u,t); (x,y,z,t,u); (y+1/2,x+1/2,z,-t,u); (-y+1/2,-x+1/2,z,t,-u)

Standard settings

Superspace group: 100.2.69.13 P4bm(a,a,1/2)000(-a,a,1/2)000 [Y:2.2765]
Bravais class: 2.69 P4/mmm(a,a,1/2)(-a,a,1/2)   [JJdW:2.69]
Transformation to supercentered setting: A1=a1, A2=a2, A3=2a3+a4+a5, A4=a4, A5=a5

BASIC SPACE GROUP SETTING
Modulation vectors: q1'=(a,a,1/2), q2'=(-a,a,1/2)
Centering: (0,0,0,0,0)
Non-lattice generators: (-y,x,z,z-u,t); (-x+1/2,y+1/2,z,u,t); (y+1/2,x-1/2,z,t,z-u)
Non-lattice operators: (x,y,z,t,u); (-x,-y,z,z-t,z-u); (-y,x,z,z-u,t); (y,-x,z,u,z-t); (-x+1/2,y+1/2,z,u,t); (x+1/2,-y+1/2,z,z-u,z-t); (y+1/2,x+1/2,z,t,z-u); (-y+1/2,-x+1/2,z,z-t,u)

SUPERCENTERED SETTING
Modulation vectors: Q1'=(A,A,0), Q2'=(-A,A,0), where A=a
Centering: (0,0,0,0,0); (0,0,1/2,1/2,1/2)
Non-lattice generators: (-Y,X,Z,-U,T); (-X+1/2,Y+1/2,Z,U,T); (Y+1/2,X-1/2,Z,T,-U)
Non-lattice operators: (X,Y,Z,T,U); (-X,-Y,Z,-T,-U); (-Y,X,Z,-U,T); (Y,-X,Z,U,-T); (-X+1/2,Y+1/2,Z,U,T); (X+1/2,-Y+1/2,Z,-U,-T); (Y+1/2,X+1/2,Z,T,-U); (-Y+1/2,-X+1/2,Z,-T,U)
Reflection conditions: HKLMN:L+M+N=2n; H0LM-M:H=2n; 0KLMM:K=2n

Affine transformation to standard basic space group setting

$S * g(input) * S^{-1} = g(standard)$,
where g is an augmented matrix for an operation in the superspace group.
Also, S * r(input) = r(standard),
where r is an augmented position vector, (x,y,z,t,u,1).

$$S = \begin{pmatrix} 1 & 0 & 0 & 0 & 0 & 0 \\ 0 & 1 & 0 & 0 & 0 & 0 \\ 0 & 0 & 2 & 0 & 0 & 0 \\ 0 & 0 & 1 & 0 & 1 & 0 \\ 0 & 0 & 1 & 1 & 0 & 0 \\ 0 & 0 & 0 & 0 & 0 & 1 \end{pmatrix} \quad S^{-1} = \begin{pmatrix} 1 & 0 & 0 & 0 & 0 & 0 \\ 0 & 1 & 0 & 0 & 0 & 0 \\ 0 & 0 & 1/2 & 0 & 0 & 0 \\ 0 & 0 & -1/2 & 0 & 1 & 0 \\ 0 & 0 & -1/2 & 1 & 0 & 0 \\ 0 & 0 & 0 & 0 & 0 & 1 \end{pmatrix}$$

a1' = a1
a2' = a2
a3' = 1/2 a3

a1 = a1'
a2 = a2'
a3 = 2 a3'

a1*' = a1*
a2*' = a2*
a3*' = 2 a3*

a1* = a1*'
a2* = a2*'
a3* = 1/2 a3*'

q1' = q2 + a3* = (a,a,1/2)
q2' = q1 + a3* = (-a,a,1/2)

q1 = q2' - 1/2 a3*' = (-a,a,0)
q2 = q1' - 1/2 a3*' = (a,a,0)

100.2.69.14 P4bm(a,a,1/2)00s(-a,a,1/2)000

Superspace group: 100.2.69.14 P4bm(a,a,1/2)00s(-a,a,1/2)000 [Y:2.2766]
Bravais class: 2.69 P4/mmm(a,a,1/2)(-a,a,1/2) [JJdW:2.69]
Transformation to supercentered setting: A1=a1, A2=a2, A3=2a3+a4+a5, A4=a4, A5=a5

BASIC SPACE GROUP SETTING
Modulation vectors: q1=(a,a,1/2), q2=(-a,a,1/2)
Centering: (0,0,0,0,0)
Non-lattice generators: (-y,x,z,z-u,t); (-x+1/2,y+1/2,z,u+1/2,t-1/2); (y+1/2,x-1/2,z,t+1/2,z-u+1/2)
Non-lattice operators: (x,y,z,t,u); (-x,-y,z,z-t,z-u); (-y,x,z,z-u,t); (y,-x,z,u,z-t); (-x+1/2,y+1/2,z,u+1/2,t+1/2); (x+1/2,-y+1/2,z,z-u+1/2,z-t+1/2); (y+1/2,x+1/2,z,t+1/2,z-u+1/2); (-y+1/2,-x+1/2,z,z-t+1/2,u+1/2)

SUPERCENTERED SETTING
Modulation vectors: Q1=(A,A,0), Q2=(-A,A,0), where A=a
Centering: (0,0,0,0,0); (0,0,1/2,1/2,1/2)
Non-lattice generators: (-Y,X,Z,-U,T); (-X+1/2,Y+1/2,Z,U+1/2,T-1/2); (Y+1/2,X-1/2,Z,T+1/2,-U+1/2)
Non-lattice operators: (X,Y,Z,T,U); (-X,-Y,Z,-T,-U); (-Y,X,Z,-U,T); (Y,-X,Z,U,-T); (-X+1/2,Y+1/2,Z+1/2,U,T); (X+1/2,-Y+1/2,Z+1/2,-U,-T); (Y+1/2,X+1/2,Z+1/2,T,-U); (-Y+1/2,-X+1/2,Z+1/2,-T,U)
Reflection conditions: HKLMN:L+M+N=2n; HHLM0:L=2n; H-HL0N:L=2n; H0LM-M:H+L=2n; 0KLMM:K+L=2n

-----

This is the SSG of Sr2TiSi2O8 : Hoche et al., JSSC 166, 15 (2004).

One other SSG exists, with different intrinsic translational components:
100.2.69.13 P4bm(a,a,1/2)000(-a,a,1/2)000 [Y:2.2765].

-----

findssg P4bm(a,a,1/2)00s(-a,a,1/2)000

Generators of the standard BSG setting entered into findssg.

Input setting

Centering
none
Operators
(-y,x,z,z-u,t); (-x+1/2,y+1/2,z,u+1/2,t+1/2); (y+1/2,x+1/2,z,t+1/2,z-u+1/2); (-x,-y,z,z-t,z-u); (-y+1/2,-x+1/2,z,z-t+1/2,u+1/2); (x,y,z,t,u); (x+1/2,-y+1/2,z,z-u+1/2,z-t+1/2); (y,-x,z,u,z-t)

Standard settings

Superspace group: 100.2.69.14 P4bm(a,a,1/2)00s(-a,a,1/2)000 [Y:2.2766]
Bravais class: 2.69 P4/mmm(a,a,1/2)(-a,a,1/2) [JJdW:2.69]
Transformation to supercentered setting: A1=a1, A2=a2, A3=2a3+a4+a5, A4=a4, A5=a5

BASIC SPACE GROUP SETTING
Modulation vectors: q1'=(a,a,1/2), q2'=(-a,a,1/2)
Centering: (0,0,0,0,0)
Non-lattice generators: (-y,x,z,z-u,t); (-x+1/2,y+1/2,z,u+1/2,t-1/2); (y+1/2,x-1/2,z,t+1/2,z-u+1/2)
Non-lattice operators: (x,y,z,t,u); (-x,-y,z,z-t,z-u); (-y,x,z,z-u,t); (y,-x,z,u,z-t); (-x+1/2,y+1/2,z,u+1/2,t+1/2); (x+1/2,-y+1/2,z,z-u+1/2,z-t+1/2); (y+1/2,x+1/2,z,t+1/2,z-u+1/2); (-y+1/2,-x+1/2,z,z-t+1/2,u+1/2)

SUPERCENTERED SETTING
Modulation vectors: Q1'=(A,A,0), Q2'=(-A,A,0), where A=a
Centering: (0,0,0,0,0); (0,0,1/2,1/2,1/2)
Non-lattice generators: (-Y,X,Z,-U,T); (-X+1/2,Y+1/2,Z,U+1/2,T-1/2); (Y+1/2,X-1/2,Z,T+1/2,-U+1/2)
Non-lattice operators: (X,Y,Z,T,U); (-X,-Y,Z,-T,-U); (-Y,X,Z,-U,T); (Y,-X,Z,U,-T); (-X+1/2,Y+1/2,Z+1/2,U,T); (X+1/2,-Y+1/2,Z+1/2,-U,-T); (Y+1/2,X+1/2,Z+1/2,T,-U); (-Y+1/2,-X+1/2,Z+1/2,-T,U)
Reflection conditions: HKLMN:L+M+N=2n; HHLM0:L=2n; H-HL0N:L=2n; H0LM-M:H+L=2n; 0KLMM:K+L=2n

Affine transformation to standard basic space group setting

$S * g(input) * S^{-1} = g(standard)$,
where g is an augmented matrix for an operation in the superspace group.
Also, S * r(input) = r(standard),
where r is an augmented position vector, (x,y,z,t,u,1).

$$S = \begin{pmatrix} 1&0&0&0&0&0 \\ 0&1&0&0&0&0 \\ 0&0&1&0&0&0 \\ 0&0&0&1&0&0 \\ 0&0&0&0&1&0 \\ 0&0&0&0&0&1 \end{pmatrix} \quad S^{-1} = \begin{pmatrix} 1&0&0&0&0&0 \\ 0&1&0&0&0&0 \\ 0&0&1&0&0&0 \\ 0&0&0&1&0&0 \\ 0&0&0&0&1&0 \\ 0&0&0&0&0&1 \end{pmatrix}$$

a1' = a1
a2' = a2
a3' = a3

a1 = a1'
a2 = a2'
a3 = a3'

a1*' = a1*
a2*' = a2*
a3*' = a3*

a1* = a1*'
a2* = a2*'
a3* = a3*'

q1' = q1 = (a,a,1/2)
q2' = q2 = (-a,a,1/2)

q1 = q1' = (a,a,1/2)
q2 = q2' = (-a,a,1/2)

findssg   Y:2.2766 P4bm(-pp1/2,pp1/2)0gg

Operators of Yamamoto entered into findssg.

Input setting

Centering
(0,0,0,0,0); (0,0,1/2,1/2,1/2)
Operators
(-x,-y,z,-t+1/2,-u+1/2); (-y,x,z,u,-t+1/2); (y,-x,z,-u+1/2,t); (x+1/2,-y+1/2,z,-u,-t); (-x+1/2,y+1/2,z,u+1/2,t+1/2); (x,y,z,t,u); (y+1/2,x+1/2,z,-t,u+1/2); (-y+1/2,-x+1/2,z,t+1/2,-u)

Standard settings

Superspace group: 100.2.69.14 P4bm(a,a,1/2)00s(-a,a,1/2)000 [Y:2.2766]
Bravais class: 2.69 P4/mmm(a,a,1/2)(-a,a,1/2)   [JJdW:2.69]
Transformation to supercentered setting: A1=a1, A2=a2, A3=2a3+a4+a5, A4=a4, A5=a5

BASIC SPACE GROUP SETTING
Modulation vectors: q1'=(a,a,1/2), q2'=(-a,a,1/2)
Centering: (0,0,0,0,0)
Non-lattice generators: (-y,x,z,z-u,t); (-x+1/2,y+1/2,z,u+1/2,t-1/2); (y+1/2,x-1/2,z,t+1/2,z-u+1/2)
Non-lattice operators: (x,y,z,t,u); (-x,-y,z,z-t,z-u); (-y,x,z,z-u,t); (y,-x,z,u,z-t); (-x+1/2,y+1/2,z,u+1/2,t+1/2); (x+1/2,-y+1/2,z,z-u+1/2,z-t+1/2); (y+1/2,x+1/2,z,t+1/2,z-u+1/2); (-y+1/2,-x+1/2,z,z-t+1/2,u+1/2)

SUPERCENTERED SETTING
Modulation vectors: Q1'=(A,A,0), Q2'=(-A,A,0), where A=a
Centering: (0,0,0,0,0); (0,0,1/2,1/2,1/2)
Non-lattice generators: (-Y,X,Z,-U,T); (-X+1/2,Y+1/2,Z,U+1/2,T-1/2); (Y+1/2,X-1/2,Z,T+1/2,-U+1/2)
Non-lattice operators: (X,Y,Z,T,U); (-X,-Y,Z,-T,-U); (-Y,X,Z,-U,T); (Y,-X,Z,U,-T); (-X+1/2,Y+1/2,Z+1/2,U,T); (X+1/2,-Y+1/2,Z+1/2,-U,-T); (Y+1/2,X+1/2,Z+1/2,T,-U); (-Y+1/2,-X+1/2,Z+1/2,-T,U)
Reflection conditions: HKLMN:L+M+N=2n; HHLM0:L=2n; H-HL0N:L=2n; H0LM-M:H+L=2n; 0KLMM:K+L=2n

Affine transformation to standard basic space group setting

S * g(input) * S^{-1} = g(standard),
where g is an augmented matrix for an operation in the superspace group.
Also, S * r(input) = r(standard),
where r is an augmented position vector, (x,y,z,t,u,1).

$$S = \begin{pmatrix} 1 & 0 & 0 & 0 & 0 & 0 \\ 0 & 1 & 0 & 0 & 0 & 0 \\ 0 & 0 & 2 & 0 & 0 & 1/2 \\ 0 & 0 & 1 & 0 & 1 & 0 \\ 0 & 0 & 1 & 1 & 0 & 0 \\ 0 & 0 & 0 & 0 & 0 & 1 \end{pmatrix} \quad S^{-1} = \begin{pmatrix} 1 & 0 & 0 & 0 & 0 & 0 \\ 0 & 1 & 0 & 0 & 0 & 0 \\ 0 & 0 & 1/2 & 0 & 0 & -1/4 \\ 0 & 0 & -1/2 & 0 & 1 & 1/4 \\ 0 & 0 & -1/2 & 1 & 0 & 1/4 \\ 0 & 0 & 0 & 0 & 0 & 1 \end{pmatrix}$$

a1' = a1
a2' = a2
a3' = 1/2 a3

a1 = a1'
a2 = a2'
a3 = 2 a3'

a1*' = a1*
a2*' = a2*
a3*' = 2 a3*

a1* = a1*'
a2* = a2*'
a3* = 1/2 a3*'

q1' = q2 + a3* = (a,a,1/2)
q2' = q1 + a3* = (-a,a,1/2)

q1 = q2' - 1/2 a3*' = (-a,a,0)
q2 = q1' - 1/2 a3*' = (a,a,0)

Yamamoto, from his WEB site

2765 P4bm(-pp1/2,pp1/2)0mm
(00000;001/21/21/2)
x,y,z,t,u; -x,-y,z,-t,-u; -y,x,z,u,-t; y,-x,z,-u,t; 1/2+x,1/2-y,z,-u,-t; 1/2-x,1/2+y,z,u,t; 1/2-y,1/2-x,z,t,-u; 1/2+y,1/2+x,z,-t,u;
hklmn:l+m+n=2n h0lm-m:h=2n 0klnn:k=2n kkl0n:k+k=2n

2766 P4bm(-pp1/2,pp1/2)0gg
(00000;001/21/21/2)
x,y,z,t,u; -x,-y,z,1/2-t,1/2-u; -y,x,z,u,1/2-t; y,-x,z,1/2-u,t; 1/2+x,1/2-y,z,-u,-t; 1/2-x,1/2+y,z,1/2+u,1/2+t; 1/2-y,1/2-x,z,1/2+t,-u; 1/2+y,1/2+x,z,-t,1/2+u;
hklmn:l+m+n=2n h0lm-m:h=2n 0klnn:k+n+n=2n -kklm0:m=2n kkl0n:k+k+n=2n
