# Supplementary material for: Equivalence of superspace groups
Source: Acta Crystallogr A. 2012 Nov 14;69(Pt 1):75–90. doi: 10.1107/S0108767312041657 (PMC3553647; doi:10.1107/S0108767312041657)
Supplement: Supplementary file 1 [file a-69-00075-sup1.zip › ssg2d_p4n_aa12_rex2-d.pdf]

85.2.58.2 P4/n(a,b,1/2)00(-b,a,1/2)00

Superspace group: 85.2.58.2 P4/n(a,b,1/2)00(-b,a,1/2)00 [Y:2.2593]
Bravais class: 2.58 P4/m(a,b,1/2)(-b,a,1/2) [JJdW:2.58]
Transformation to supercentered setting: A1=a1, A2=a2, A3=2a3+a4+a5, A4=a4, A5=a5

BASIC SPACE GROUP SETTING
Modulation vectors: q1=(a,b,1/2), q2=(-b,a,1/2)
Centering: (0,0,0,0,0)
Non-lattice generators: (-y+1/2,x,z,z-u,t); (x+1/2,y+1/2,-z,-z+t,-z+u)
Non-lattice operators: (x,y,z,t,u); (-x+1/2,-y+1/2,z,z-t,z-u); (-y+1/2,x,z,z-u,t); (y,-x+1/2,z,u,z-t); (-x,-y,-z,-t,-u); (x+1/2,y+1/2,-z,-z+t,-z+u); (y+1/2,-x,-z,-z+u,-t); (-y,x+1/2,-z,-u,-z+t)

SUPERCENTERED SETTING
Modulation vectors: Q1=(A,B,0), Q2=(-B,A,0), where A=a, B=b
Centering: (0,0,0,0,0); (0,0,1/2,1/2,1/2)
Non-lattice generators: (-Y+1/2,X,Z,-U,T); (X+1/2,Y+1/2,-Z,T,U)
Non-lattice operators: (X,Y,Z,T,U); (-X+1/2,-Y+1/2,Z,-T,-U); (-Y+1/2,X,Z,-U,T); (Y,-X+1/2,Z,U,-T); (-X,-Y,-Z,-T,-U); (X+1/2,Y+1/2,-Z,T,U); (Y+1/2,-X,-Z,U,-T); (-Y,X+1/2,-Z,-U,T)
Reflection conditions: HKLMN:L+M+N=2n; HK0MN:H+K=2n

There exist only one SSG in this Bravais class that possesses BSG P4/n.

This is the symmetry of $LaSe_{1.85}$, $CeSe_{1.83}$, $NdSe_{1.83}$ and $SmSe_{1.84}$ and several other RSe_{2-x} compounds. See C. Graf and T. Doert, Z. Kristallogr. 224, 568-579 (2009).

This is the SSG of $Nd_{0.6}Gd_{0.4}Se_{1.8}$ (Doert, 2004) .

P4/n(a,b,1/2)0s(-b,a,1/2)0s is an alternate setting that has been given for $GdS_{1.82}$ (Tamazyan, 2003).

findssg P4/n(a,b,1/2)00(-b,a,1/2)00

Generators of standard BSG setting entered into findssg.

Input setting

Centering
none
Operators
(-y+1/2,x,z,z-u,t); (x+1/2,y+1/2,-z,-z+t,-z+u); (-x+1/2,-y+1/2,z,z-t,z-u); (-y,x+1/2,-z,-u,-z+t); (x,y,z,t,u); (-x,-y,-z,-t,-u); (y,-x+1/2,z,u,z-t); (y+1/2,-x,-z,-z+u,-t)

Standard settings

Superspace group: 85.2.58.2 P4/n(a,b,1/2)00(-b,a,1/2)00 [Y:2.2593]
Bravais class: 2.58 P4/m(a,b,1/2)(-b,a,1/2) [JJdW:2.58]
Transformation to supercentered setting: A1=a1, A2=a2, A3=2a3+a4+a5, A4=a4, A5=a5

BASIC SPACE GROUP SETTING
Modulation vectors: q1'=(a,b,1/2), q2'=(-b,a,1/2)
Centering: (0,0,0,0,0)
Non-lattice generators: (-y+1/2,x,z,z-u,t); (x+1/2,y+1/2,-z,-z+t,-z+u)
Non-lattice operators: (x,y,z,t,u); (-x+1/2,-y+1/2,z,z-t,z-u); (-y+1/2,x,z,z-u,t); (y,-x+1/2,z,u,z-t); (-x,-y,-z,-t,-u); (x+1/2,y+1/2,-z,-z+t,-z+u); (y+1/2,-x,-z,-z+u,-t); (-y,x+1/2,-z,-u,-z+t)

SUPERCENTERED SETTING
Modulation vectors: Q1'=(A,B,0), Q2'=(-B,A,0), where A=a, B=b
Centering: (0,0,0,0,0); (0,0,1/2,1/2,1/2)
Non-lattice generators: (-Y+1/2,X,Z,-U,T); (X+1/2,Y+1/2,-Z,T,U)
Non-lattice operators: (X,Y,Z,T,U); (-X+1/2,-Y+1/2,Z,-T,-U); (-Y+1/2,X,Z,-U,T); (Y,-X+1/2,Z,U,-T); (-X,-Y,-Z,-T,-U); (X+1/2,Y+1/2,-Z,T,U); (Y+1/2,-X,-Z,U,-T); (-Y,X+1/2,-Z,-U,T)
Reflection conditions: HKLMN:L+M+N=2n; HK0MN:H+K=2n

Affine transformation to standard basic space group setting

$S * g(\text{input}) * S^{-1} = g(\text{standard})$,
where g is an augmented matrix for an operation in the superspace group.
Also, S * r(input) = r(standard),
where r is an augmented position vector, (x,y,z,t,u,1).

$$S = \begin{matrix} 1&0&0&0&0&0 \\ 0&1&0&0&0&0 \\ 0&0&1&0&0&0 \\ 0&0&0&1&0&0 \\ 0&0&0&0&1&0 \\ 0&0&0&0&0&1 \end{matrix} \qquad S^{-1} = \begin{matrix} 1&0&0&0&0&0 \\ 0&1&0&0&0&0 \\ 0&0&1&0&0&0 \\ 0&0&0&1&0&0 \\ 0&0&0&0&1&0 \\ 0&0&0&0&0&1 \end{matrix}$$

a1' = a1
a2' = a2
a3' = a3

a1 = a1'
a2 = a2'
a3 = a3'

a1*' = a1*
a2*' = a2*
a3*' = a3*

a1* = a1*'
a2* = a2*'
a3* = a3*'

q1' = q1 = (a,b,1/2)
q2' = q2 = (-b,a,1/2)

q1 = q1' = (a,b,1/2)
q2 = q2' = (-b,a,1/2)

findssg X4/n(a,b,0)00(-b,a,0)00

Generators of standard supercentered setting entered into findssg.

Input setting

Centering
(0,0,0,0,0); (0,0,1/2,1/2,1/2)
Operators
(-y+1/2,x,z,-u,t); (x+1/2,y+1/2,-z,t,u); (-x+1/2,-y+1/2,z,-t,-u); (-y,x+1/2,-z,-u,t); (x,y,z,t,u); (-x,-y,-z,-t,-u); (y,-x+1/2,z,u,-t); (y+1/2,-x,-z,u,-t)

Standard settings

Superspace group: 85.2.58.2 P4/n(a,b,1/2)00(-b,a,1/2)00 [Y:2.2593]
Bravais class: 2.58 P4/m(a,b,1/2)(-b,a,1/2) [JJdW:2.58]
Transformation to supercentered setting: A1=a1, A2=a2, A3=2a3+a4+a5, A4=a4, A5=a5

BASIC SPACE GROUP SETTING
Modulation vectors: q1'=(a,b,1/2), q2'=(-b,a,1/2)
Centering: (0,0,0,0,0)
Non-lattice generators: (-y+1/2,x,z,z-u,t); (x+1/2,y+1/2,-z,-z+t,-z+u)
Non-lattice operators: (x,y,z,t,u); (-x+1/2,-y+1/2,z,z-t,z-u); (-y+1/2,x,z,z-u,t); (y,-x+1/2,z,u,z-t); (-x,-y,-z,-t,-u); (x+1/2,y+1/2,-z,-z+t,-z+u); (y+1/2,-x,-z,-z+u,-t); (-y,x+1/2,-z,-u,-z+t)

SUPERCENTERED SETTING
Modulation vectors: Q1'=(A,B,0), Q2'=(-B,A,0), where A=a, B=b
Centering: (0,0,0,0,0); (0,0,1/2,1/2,1/2)
Non-lattice generators: (-Y+1/2,X,Z,-U,T); (X+1/2,Y+1/2,-Z,T,U)
Non-lattice operators: (X,Y,Z,T,U); (-X+1/2,-Y+1/2,Z,-T,-U); (-Y+1/2,X,Z,-U,T); (Y,-X+1/2,Z,U,-T); (-X,-Y,-Z,-T,-U); (X+1/2,Y+1/2,-Z,T,U); (Y+1/2,-X,-Z,U,-T); (-Y,X+1/2,-Z,-U,T)
Reflection conditions: HKLMN:L+M+N=2n; HK0MN:H+K=2n

Affine transformation to standard basic space group setting

S * g(input) * S $^{-1}$ = g(standard),
where g is an augmented matrix for an operation in the superspace group.
Also, S * r(input) = r(standard),
where r is an augmented position vector, (x,y,z,t,u,1).

$$S = \begin{matrix} 1 & 0 & 0 & 0 & 0 & 0 \\ 0 & 1 & 0 & 0 & 0 & 0 \\ 0 & 0 & 2 & 0 & 0 & 0 \\ 0 & 0 & 1 & 1 & 0 & 0 \\ 0 & 0 & 1 & 0 & 1 & 0 \\ 0 & 0 & 0 & 0 & 0 & 1 \end{matrix} \qquad S^{-1} = \begin{matrix} 1 & 0 & 0 & 0 & 0 & 0 \\ 0 & 1 & 0 & 0 & 0 & 0 \\ 0 & 0 & 1/2 & 0 & 0 & 0 \\ 0 & 0 & -1/2 & 1 & 0 & 0 \\ 0 & 0 & -1/2 & 0 & 1 & 0 \\ 0 & 0 & 0 & 0 & 0 & 1 \end{matrix}$$

a1' = a1
a2' = a2
a3' = 1/2 a3

a1 = a1'
a2 = a2'
a3 = 2 a3'

a1*' = a1*
a2*' = a2*
a3*' = 2 a3*

a1* = a1*'
a2* = a2*'
a3* = 1/2 a3*'

q1' = q1 + a3* = (a,b,1/2)
q2' = q2 + a3* = (-b,a,1/2)

q1 = q1' - 1/2 a3*' = (a,b,0)
q2 = q2' - 1/2 a3*' = (-b,a,0)

findssg P4/n(a,b,1/2)0s(-b,a,1/2)0s

Published BSG setting of $GdS_{1.82}$ (Tamazyan, 2003) has been entered into findssg.
The transformation to standard BSG setting is an origin shift.

Input setting

Centering
none
Operators
(-y+1/2,x,z,z-u,t); (x+1/2,y+1/2,-z,-z+t+1/2,-z+u+1/2); (-x+1/2,-y+1/2,z,z-t,z-u); (-y,x+1/2,-z,-u+1/2,-z+t+1/2); (y,-x+1/2,z,u,z-t); (x,y,z,t,u); (-x,-y,-z,-t+1/2,-u+1/2); (y+1/2,-x,-z,-z+u+1/2,-t+1/2)

Standard settings

Superspace group: 85.2.58.2 P4/n(a,b,1/2)00(-b,a,1/2)00 [Y:2.2593]
Bravais class: 2.58 P4/m(a,b,1/2)(-b,a,1/2) [JJdW:2.58]
Transformation to supercentered setting: A1=a1, A2=a2, A3=2a3+a4+a5, A4=a4, A5=a5

BASIC SPACE GROUP SETTING
Modulation vectors: q1'=(a,b,1/2), q2'=(-b,a,1/2)
Centering: (0,0,0,0,0)
Non-lattice generators: (-y+1/2,x,z,z-u,t); (x+1/2,y+1/2,-z,-z+t,-z+u)
Non-lattice operators: (x,y,z,t,u); (-x+1/2,-y+1/2,z,z-t,z-u); (-y+1/2,x,z,z-u,t); (y,-x+1/2,z,u,z-t); (-x,-y,-z,-t,-u); (x+1/2,y+1/2,-z,-z+t,-z+u); (y+1/2,-x,-z,-z+u,-t); (-y,x+1/2,-z,-u,-z+t)

SUPERCENTERED SETTING
Modulation vectors: Q1'=(A,B,0), Q2'=(-B,A,0), where A=a, B=b
Centering: (0,0,0,0,0); (0,0,1/2,1/2,1/2)
Non-lattice generators: (-Y+1/2,X,Z,-U,T); (X+1/2,Y+1/2,-Z,T,U)
Non-lattice operators: (X,Y,Z,T,U); (-X+1/2,-Y+1/2,Z,-T,-U); (-Y+1/2,X,Z,-U,T); (Y,-X+1/2,Z,U,-T); (-X,-Y,-Z,-T,-U); (X+1/2,Y+1/2,-Z,T,U); (Y+1/2,-X,-Z,U,-T); (-Y,X+1/2,-Z,-U,T)
Reflection conditions: HKLMN:L+M+N=2n; HK0MN:H+K=2n

Affine transformation to standard basic space group setting

$S * g(input) * S^{-1} = g(standard)$,
where g is an augmented matrix for an operation in the superspace group.
Also, S * r(input) = r(standard),
where r is an augmented position vector, (x,y,z,t,u,1).

$$S = \begin{pmatrix} 1 & 0 & 0 & 0 & 0 & 0 \\ 0 & 1 & 0 & 0 & 0 & 0 \\ 0 & 0 & 1 & 0 & 0 & 1/2 \\ 0 & 0 & 0 & 1 & 0 & 3/4 \\ 0 & 0 & 0 & 0 & 1 & 3/4 \\ 0 & 0 & 0 & 0 & 0 & 1 \end{pmatrix} \quad S^{-1} = \begin{pmatrix} 1 & 0 & 0 & 0 & 0 & 0 \\ 0 & 1 & 0 & 0 & 0 & 0 \\ 0 & 0 & 1 & 0 & 0 & -1/2 \\ 0 & 0 & 0 & 1 & 0 & -3/4 \\ 0 & 0 & 0 & 0 & 1 & -3/4 \\ 0 & 0 & 0 & 0 & 0 & 1 \end{pmatrix}$$

a1' = a1
a2' = a2
a3' = a3

a1 = a1'
a2 = a2'
a3 = a3'

a1*' = a1*
a2*' = a2*
a3*' = a3*

a1* = a1*'
a2* = a2*'
a3* = a3*'

q1' = q1 = (a,b,1/2)
q2' = q2 = (-b,a,1/2)

q1 = q1' = (a,b,1/2)
q2 = q2' = (-b,a,1/2)

findssg

**Published supercentered setting of $LaSe_{1.85}$, [C. Graf and T. Doert, Z. Kristallogr. 224, 568-579 (2009)]---which is the setting of superspace group No.
2593 P4/n(-pq1/2,qp1/2)00
(00000;001/21/21/2)
x,y,z,t,u; 1/2-x,1/2-y,z,-t,-u; -y,1/2+x,z,u,-t; 1/2+y,-x,z,-u,t; -x,-y,-z,-t,-u; 1/2+x,1/2+y,-z,t,u; y,1/2-x,-z,-u,t; 1/2-y,x,-z,u,-t;
hklmn:l+m+n=2n hk0mn:h+k=2n
of Yamamoto-Tables--- has been entered into findssg.**

Input setting

Centering
(0,0,0,0,0); (0,0,1/2,1/2,1/2)
Operators
(-x+1/2,-y+1/2,z,-t,-u); (-y,x+1/2,z,u,-t); (y+1/2,-x,z,-u,t); (-x,-y,-z,-t,-u); (x,y,z,t,u); (x+1/2,y+1/2,-z,t,u); (y,-x+1/2,-z,-u,t); (-y+1/2,x,-z,u,-t)

Standard settings

Superspace group: 85.2.58.2 P4/n(a,b,1/2)00(-b,a,1/2)00 [Y:2.2593]
Bravais class: 2.58 P4/m(a,b,1/2)(-b,a,1/2) [JJdW:2.58]
Transformation to supercentered setting: A1=a1, A2=a2, A3=2a3+a4+a5, A4=a4, A5=a5

BASIC SPACE GROUP SETTING
Modulation vectors: q1'=(a,b,1/2), q2'=(-b,a,1/2)
Centering: (0,0,0,0,0)
Non-lattice generators: (-y+1/2,x,z,z-u,t); (x+1/2,y+1/2,-z,-z+t,-z+u)
Non-lattice operators: (x,y,z,t,u); (-x+1/2,-y+1/2,z,z-t,z-u); (-y+1/2,x,z,z-u,t); (y,-x+1/2,z,u,z-t); (-x,-y,-z,-t,-u); (x+1/2,y+1/2,-z,-z+t,-z+u); (y+1/2,-x,-z,-z+u,-t); (-y,x+1/2,-z,-u,-z+t)

SUPERCENTERED SETTING
Modulation vectors: Q1'=(A,B,0), Q2'=(-B,A,0), where A=a, B=b
Centering: (0,0,0,0,0); (0,0,1/2,1/2,1/2)
Non-lattice generators: (-Y+1/2,X,Z,-U,T); (X+1/2,Y+1/2,-Z,T,U)
Non-lattice operators: (X,Y,Z,T,U); (-X+1/2,-Y+1/2,Z,-T,-U); (-Y+1/2,X,Z,-U,T); (Y,-X+1/2,Z,U,-T); (-X,-Y,-Z,-T,-U); (X+1/2,Y+1/2,-Z,T,U); (Y+1/2,-X,-Z,U,-T); (-Y,X+1/2,-Z,-U,T)
Reflection conditions: HKLMN:L+M+N=2n; HK0MN:H+K=2n

Affine transformation to standard basic space group setting

S * g(input) * S^{-1} = g(standard),
where g is an augmented matrix for an operation in the superspace group.
Also, S * r(input) = r(standard),
where r is an augmented position vector, (x,y,z,t,u,1).

$$S = \begin{matrix} 1 & 0 & 0 & 0 & 0 & 1/2 \\ 0 & 1 & 0 & 0 & 0 & 0 \\ 0 & 0 & 2 & 0 & 0 & 0 \\ 0 & 0 & 1 & 0 & 1 & 0 \\ 0 & 0 & 1 & 1 & 0 & 0 \\ 0 & 0 & 0 & 0 & 0 & 1 \end{matrix} \quad S^{-1} = \begin{matrix} 1 & 0 & 0 & 0 & 0 & -1/2 \\ 0 & 1 & 0 & 0 & 0 & 0 \\ 0 & 0 & 1/2 & 0 & 0 & 0 \\ 0 & 0 & -1/2 & 0 & 1 & 0 \\ 0 & 0 & -1/2 & 1 & 0 & 0 \\ 0 & 0 & 0 & 0 & 0 & 1 \end{matrix}$$

a1' = a1
a2' = a2
a3' = 1/2 a3

a1 = a1'
a2 = a2'
a3 = 2 a3'

a1*' = a1*
a2*' = a2*
a3*' = 2 a3*

a1* = a1*'
a2* = a2*'
a3* = 1/2 a3*'

q1' = q2 + a3* = (a,b,1/2)
q2' = q1 + a3* = (-b,a,1/2)

q1 = q2' - 1/2 a3*' = (-b,a,0)
q2 = q1' - 1/2 a3*' = (a,b,0)
