# Supplementary material for: Equivalence of superspace groups
Source: Acta Crystallogr A. 2012 Nov 14;69(Pt 1):75–90. doi: 10.1107/S0108767312041657 (PMC3553647; doi:10.1107/S0108767312041657)
Supplement: Supplementary file 1 [file a-69-00075-sup1.zip › ssg2d_p63mc_aa0_agxnbs2.pdf]

186.2.83.4 $P6_3mc(a,a,0)000(-2a,a,0)000$

Superspace group: 186.2.83.4 P6_3mc(a,a,0)000(-2a,a,0)000 [Y:2.3317]
Bravais class: 2.83 P6/mmm(a,a,0)(-2a,a,0)   [JJdW:2.83]
Transformation to supercentered setting: none
Modulation vectors: q1=(a,a,0), q2=(-2a,a,0)
Centering: (0,0,0,0,0)
Non-lattice generators: (x-y,x,z+1/2,-u,t+u); (x,x-y,z,-u,-t); (y,x,z+1/2,t,-t-u)
Non-lattice operators: (x,y,z,t,u); (x-y,x,z+1/2,-u,t+u); (-y,x-y,z,-t-u,t); (-x,-y,z+1/2,-t,-u); (-x+y,-x,z,u,-t-u); (y,-x+y,z+1/2,t+u,-t); (-x+y,y,z,t+u,-u); (-x,-x+y,z+1/2,u,t); (-y,-x,z,-t,t+u); (x-y,-y,z+1/2,-t-u,u); (x,x-y,z,-u,-t); (y,x,z+1/2,t,-t-u)
Reflection conditions: h2hlmm:l=2n; hhlm0:l=2n; 2kkl0n:l=2n

There is no supercentered setting, i.e. this is a primitive superspace lattice.
This is the symmetry of $Ag_{0.6}NbS_2$:
A. van der Lee et al., Phys. Rev. B 43, 12, 9420-9430 (1991).

There is only one possible SSG.

findssg $P6_3mc(a,a,0)000(-2a,a,0)000$

Generators of standard BSG setting entered into findssg.

Input setting

Centering
none
Operators
(x-y,x,z+1/2,-u,t+u); (x,x-y,z,-u,-t); (y,x,z+1/2,t,-t-u); (-y,x-y,z,-t-u,t); (-x+y,y,z,t+u,-u); (x-y,-y,z+1/2,-t-u,u); (x,y,z,t,u); (y,-x+y,z+1/2,t+u,-t); (-y,-x,z,-t,t+u); (-x+y,-x,z,u,-t-u); (-x,-y,z+1/2,-t,-u); (-x,-x+y,z+1/2,u,t)

Standard settings

Superspace group: 186.2.83.4 P6_3mc(a,a,0)000(-2a,a,0)000 [Y:2.3317]
Bravais class: 2.83 P6/mmm(a,a,0)(-2a,a,0) [JJdW:2.83]
Transformation to supercentered setting: none
Modulation vectors: q1'=(a,a,0), q2'=(-2a,a,0)
Centering: (0,0,0,0,0)
Non-lattice generators: (x-y,x,z+1/2,-u,t+u); (x,x-y,z,-u,-t); (y,x,z+1/2,t,-t-u)
Non-lattice operators: (x,y,z,t,u); (x-y,x,z+1/2,-u,t+u); (-y,x-y,z,-t-u,t); (-x,-y,z+1/2,-t,-u); (-x+y,-x,z,u,-t-u); (y,-x+y,z+1/2,t+u,-t); (-x+y,y,z,t+u,-u); (-x,-x+y,z+1/2,u,t); (-y,-x,z,-t,t+u); (x-y,-y,z+1/2,-t-u,u); (x,x-y,z,-u,-t); (y,x,z+1/2,t,-t-u)
Reflection conditions: h2hlmm:l=2n; hhlm0:l=2n; 2kkl0n:l=2n

Affine transformation to standard basic space group setting

S * g(input) * S $^{-1}$ = g(standard),
where g is an augmented matrix for an operation in the superspace group.
Also, S * r(input) = r(standard),
where r is an augmented position vector, (x,y,z,t,u,1).

$$S = \begin{pmatrix} 1&0&0&0&0&0\\ 0&1&0&0&0&0\\ 0&0&1&0&0&0\\ 0&0&0&1&0&0\\ 0&0&0&0&1&0\\ 0&0&0&0&0&1 \end{pmatrix} \quad S^{-1} = \begin{pmatrix} 1&0&0&0&0&0\\ 0&1&0&0&0&0\\ 0&0&1&0&0&0\\ 0&0&0&1&0&0\\ 0&0&0&0&1&0\\ 0&0&0&0&0&1 \end{pmatrix}$$

a1' = a1		a3* = a3*'
a2' = a2	a1*' = a1*	
a3' = a3	a2*' = a2*	q1' = q1 = (a,a,0)
	a3*' = a3*	q2' = q2 = (-2a,a,0)
a1 = a1'		
a2 = a2'	a1* = a1*'	q1 = q1' = (a,a,0)
a3 = a3'	a2* = a2*'	q2 = q2' = (-2a,a,0)

findssg $P6_3mc(a,a,0)000(-2a,a,0)000$

Operators of the published setting have been entered into findssg. The published setting employs q1=(ap, ap, 0) ; q2=(-ap,2ap,0).

Input setting

Centering
none
Operators
(-y,x-y,z,-u,t-u); (-x+y,-x,z,-t+u,-t); (-x,-y,z+1/2,-t,-u); (y,-x+y,z+1/2,u,-t+u); (x-y,x,z+1/2,t-u,t); (-y,-x,z,-t,-t+u); (-x+y,y,z,u,t); (x,x-y,z,t-u,-u); (y,x,z+1/2,t,t-u); (x-y,-y,z+1/2,-u,-t); (-x,-x+y,z+1/2,-t+u,u); (x,y,z,t,u)

Standard settings

Superspace group: 186.2.83.4 P6_3mc(a,a,0)000(-2a,a,0)000 [Y:2.3317]
Bravais class: 2.83 P6/mmm(a,a,0)(-2a,a,0) [JJdW:2.83]
Transformation to supercentered setting: none
Modulation vectors: q1'=(a,a,0), q2'=(-2a,a,0)
Centering: (0,0,0,0,0)
Non-lattice generators: (x-y,x,z+1/2,-u,t+u); (x,x-y,z,-u,-t); (y,x,z+1/2,t,-t-u)
Non-lattice operators: (x,y,z,t,u); (x-y,x,z+1/2,-u,t+u); (-y,x-y,z,-t-u,t); (-x,-y,z+1/2,-t,-u); (-x+y,-x,z,u,-t-u); (y,-x+y,z+1/2,t+u,-t); (-x+y,y,z,t+u,-u); (-x,-x+y,z+1/2,u,t); (-y,-x,z,-t,t+u); (x-y,-y,z+1/2,-t-u,u); (x,x-y,z,-u,-t); (y,x,z+1/2,t,-t-u)
Reflection conditions: h2hlmm:l=2n; hhlm0:l=2n; 2kkl0n:l=2n

Affine transformation to standard basic space group setting

$S * g(\text{input}) * S^{-1} = g(\text{standard})$,
where g is an augmented matrix for an operation in the superspace group.
Also, S * r(input) = r(standard),
where r is an augmented position vector, (x,y,z,t,u,1).

$$S = \begin{pmatrix} 0 & 1 & 0 & 0 & 0 & 0 \\ 1 & 0 & 0 & 0 & 0 & 0 \\ 0 & 0 & -1 & 0 & 0 & 0 \\ 0 & 0 & 0 & -1 & 0 & 0 \\ 0 & 0 & 0 & 0 & 1 & 0 \\ 0 & 0 & 0 & 0 & 0 & 1 \end{pmatrix} \quad S^{-1} = \begin{pmatrix} 0 & 1 & 0 & 0 & 0 & 0 \\ 1 & 0 & 0 & 0 & 0 & 0 \\ 0 & 0 & -1 & 0 & 0 & 0 \\ 0 & 0 & 0 & -1 & 0 & 0 \\ 0 & 0 & 0 & 0 & 1 & 0 \\ 0 & 0 & 0 & 0 & 0 & 1 \end{pmatrix}$$

a1' = a2	a3 = -a3'	a1* = a2*'	
a2' = a1		a2* = a1*'	q1 = -q1' = (-a,-a,0)
a3' = -a3	a1*' = a2*	a3* = -a3*'	q2 = q2' = (a,-2a,0)
	a2*' = a1*		
a1 = a2'	a3*' = -a3*	q1' = -q1 = (a,a,0)	
a2 = a1'		q2' = q2 = (-2a,a,0)	
