# Supplementary material for: Equivalence of superspace groups
Source: Acta Crystallogr A. 2012 Nov 14;69(Pt 1):75–90. doi: 10.1107/S0108767312041657 (PMC3553647; doi:10.1107/S0108767312041657)
Supplement: Supplementary file 1 [file a-69-00075-sup1.zip › ssg2d_p63mmc_aa0_au2cd.pdf]

194.2.83.4 $P6_3/mmc(a,a,0)0000(-2a,a,0)0000$

Superspace group: 194.2.83.4 P6_3/mmc(a,a,0)0000(-2a,a,0)0000 [Y:2.3354]
Bravais class: 2.83 P6/mmm(a,a,0)(-2a,a,0) [JJdW:2.83]
Transformation to supercentered setting: none
Modulation vectors: q1=(a,a,0), q2=(-2a,a,0)
Centering: (0,0,0,0,0)
Non-lattice generators: (x-y,x,z+1/2,-u,t+u); (x,y,-z+1/2,t,u); (x,x-y,z,-u,-t); (y,x,z+1/2,t,-t-u)
Non-lattice operators: (x,y,z,t,u); (x-y,x,z+1/2,-u,t+u); (-y,x-y,z,-t-u,t); (-x,-y,z+1/2,-t,-u); (-x+y,-x,z,u,-t-u); (y,-x+y,z+1/2,t+u,-t); (x-y,-y,-z,-t-u,u); (x,x-y,-z+1/2,-u,-t); (y,x,-z,t,-t-u); (-x+y,y,-z+1/2,t+u,-u); (-x,-x+y,-z,u,t); (-y,-x,-z+1/2,-t,t+u); (-x,-y,-z,-t,-u); (-x+y,-x,-z+1/2,u,-t-u); (y,-x+y,-z,t+u,-t); (x,y,-z+1/2,t,u); (x-y,x,-z,-u,t+u); (-y,x-y,-z+1/2,-t-u,t); (-x+y,y,z,t+u,-u); (-x,-x+y,z+1/2,u,t); (-y,-x,z,-t,t+u); (x-y,-y,z+1/2,-t-u,u); (x,x-y,z,-u,-t); (y,x,z+1/2,t,-t-u)
Reflection conditions: h2hlmm:l=2n; hhlm0:l=2n; 2kkl0n:l=2n

There is no supercentered setting, i.e. this is a primitive superspace lattice.
This is the symmetry of $Au_{2+x}Cd_{1-x}$:
A. Yamamoto, Acta Crystallogr. B 39, 17-20 (1983).

This is also the symmetry of isostructural Cu_5Sb:
K. Motai, Y. Watanabe and S. Hashimoto, Acta Crystallogr. B 49, 655-661 (1993).
"Lattice modulation of the epsilon-phase in a Cu-Sb alloy."
q1 = 0.430 (a*+b*); q2 = 0.430 (-a*+2b*). Observe q1+q2 mixed higher-order satellites.

There is only one possible SSG.

findssg $P6_3$/mmc(a,a,0)0000(-2a,a,0)0000

Generators of standard BSG setting given to findssg.

Input setting

Centering
none
Operators
(x-y,x,z+1/2,-u,t+u); (x,y,-z+1/2,t,u); (x,x-y,z,-u,-t); (y,x,z+1/2,t,-t-u); (-y,x-y,z,-t-u,t); (x-y,x,-z,-u,t+u); (-x+y,y,z,t+u,-u); (x,y,z,t,u); (x,x-y,-z+1/2,-u,-t); (y,x,-z,t,-t-u); (-y,x-y,-z+1/2,-t-u,t); (-x+y,y,-z+1/2,t+u,-u); (x-y,-y,z+1/2,-t-u,u); (y,-x+y,z+1/2,t+u,-t); (-y,-x,z,-t,t+u); (x-y,-y,-z,-t-u,u); (-x+y,-x,z,u,-t-u); (y,-x+y,-z,t+u,-t); (-y,-x,-z+1/2,-t,t+u); (-x+y,-x,-z+1/2,u,-t-u); (-x,-y,z+1/2,-t,-u); (-x,-x+y,z+1/2,u,t); (-x,-y,-z,-t,-u); (-x,-x+y,-z,u,t)

Standard settings

Superspace group: 194.2.83.4 P6_3/mmc(a,a,0)0000(-2a,a,0)0000 [Y:2.3354]
Bravais class: 2.83 P6/mmm(a,a,0)(-2a,a,0) [JJdW:2.83]
Transformation to supercentered setting: none
Modulation vectors: q1'=(a,a,0), q2'=(-2a,a,0)
Centering: (0,0,0,0,0)
Non-lattice generators: (x-y,x,z+1/2,-u,t+u); (x,y,-z+1/2,t,u); (x,x-y,z,-u,-t); (y,x,z+1/2,t,-t-u)
Non-lattice operators: (x,y,z,t,u); (x-y,x,z+1/2,-u,t+u); (-y,x-y,z,-t-u,t); (-x,-y,z+1/2,-t,-u); (-x+y,-x,z,u,-t-u); (y,-x+y,z+1/2,t+u,-t); (x-y,-y,-z,-t-u,u); (x,x-y,-z+1/2,-u,-t); (y,x,-z,t,-t-u); (-x+y,y,-z+1/2,t+u,-u); (-x,-x+y,-z,u,t); (-y,-x,-z+1/2,-t,t+u); (-x,-y,-z,-t,-u); (-x+y,-x,-z+1/2,u,-t-u); (y,-x+y,-z,t+u,-t); (x,y,-z+1/2,t,u); (x-y,x,-z,-u,t+u); (-y,x-y,-z+1/2,-t-u,t); (-x+y,y,z,t+u,-u); (-x,-x+y,z+1/2,u,t); (-y,-x,z,-t,t+u); (x-y,-y,z+1/2,-t-u,u); (x,x-y,z,-u,-t); (y,x,z+1/2,t,-t-u)
Reflection conditions: h2hlmm:l=2n; hhlm0:l=2n; 2kkl0n:l=2n

Affine transformation to standard basic space group setting

S * g(input) * S^{-1} = g(standard),
where g is an augmented matrix for an operation in the superspace group.
Also, S * r(input) = r(standard),
where r is an augmented position vector, (x,y,z,t,u,1).

$$S = \begin{pmatrix} 1&0&0&0&0&0\\ 0&1&0&0&0&0\\ 0&0&1&0&0&0\\ 0&0&0&1&0&0\\ 0&0&0&0&1&0\\ 0&0&0&0&0&1 \end{pmatrix} \quad S^{-1} = \begin{pmatrix} 1&0&0&0&0&0\\ 0&1&0&0&0&0\\ 0&0&1&0&0&0\\ 0&0&0&1&0&0\\ 0&0&0&0&1&0\\ 0&0&0&0&0&1 \end{pmatrix}$$

$a1' = a1$
$a2' = a2$
$a3' = a3$

$a1 = a1'$
$a2 = a2'$
$a3 = a3'$

$a1^{*\prime} = a1^*$
$a2^{*\prime} = a2^*$
$a3^{*\prime} = a3^*$

$a1^* = a1^{*\prime}$
$a2^* = a2^{*\prime}$
$a3^* = a3^{*\prime}$

$q1' = q1 = (a,a,0)$
$q2' = q2 = (-2a,a,0)$

$q1 = q1' = (a,a,0)$
$q2 = q2' = (-2a,a,0)$

findssg $P6_3$/mmc(a,a,0)0000(-a,2a,0)0000

Generators of of published setting have been entered into findssg.

Input setting

Centering
none
Operators
(-y,x-y,z,-u,t-u); (-x+y,-x,z,-t+u,-t); (-x,-y,z+1/2,-t,-u); (y,-x+y,z+1/2,u,-t+u); (x-y,x,z+1/2,t-u,t); (y,x,-z,t,t-u); (x-y,-y,-z,-u,-t); (-x,-x+y,-z,-t+u,u); (-y,-x,-z+1/2,-t,-t+u); (-x+y,y,-z+1/2,u,t); (x,x-y,-z+1/2,t-u,-u); (-x,-y,-z,-t,-u); (y,-x+y,-z,u,-t+u); (x-y,x,-z,t-u,t); (x,y,-z+1/2,t,u); (-y,x-y,-z+1/2,-u,t-u); (-x+y,-x,-z+1/2,-t+u,-t); (-y,-x,z,-t,-t+u); (-x+y,y,z,u,t); (x,x-y,z,t-u,-u); (y,x,z+1/2,t,t-u); (x-y,-y,z+1/2,-u,-t); (-x,-x+y,z+1/2,-t+u,u); (x,y,z,t,u)

Standard settings

Superspace group: 194.2.83.4 P6_3/mmc(a,a,0)0000(-2a,a,0)0000 [Y:2.3354]
Bravais class: 2.83 P6/mmm(a,a,0)(-2a,a,0)   [JJdW:2.83]
Transformation to supercentered setting: none
Modulation vectors: q1'=(a,a,0), q2'=(-2a,a,0)
Centering: (0,0,0,0,0)
Non-lattice generators: (x-y,x,z+1/2,-u,t+u); (x,y,-z+1/2,t,u); (x,x-y,z,-u,-t); (y,x,z+1/2,t,-t-u)
Non-lattice operators: (x,y,z,t,u); (x-y,x,z+1/2,-u,t+u); (-y,x-y,z,-t-u,t); (-x,-y,z+1/2,-t,-u); (-x+y,-x,z,u,-t-u); (y,-x+y,z+1/2,t+u,-t); (x-y,-y,-z,-t-u,u); (x,x-y,-z+1/2,-u,-t); (y,x,-z,t,-t-u); (-x+y,y,-z+1/2,t+u,-u); (-x,-x+y,-z,u,t); (-y,-x,-z+1/2,-t,t+u); (-x,-y,-z,-t,-u); (-x+y,-x,-z+1/2,u,-t-u); (y,-x+y,-z,t+u,-t); (x,y,-z+1/2,t,u); (x-y,x,-z,-u,t+u); (-y,x-y,-z+1/2,-t-u,t); (-x+y,y,z,t+u,-u); (-x,-x+y,z+1/2,u,t); (-y,-x,z,-t,t+u); (x-y,-y,z+1/2,-t-u,u); (x,x-y,z,-u,-t); (y,x,z+1/2,t,-t-u)
Reflection conditions: h2hlmm:l=2n; hhlm0:l=2n; 2kkl0n:l=2n

Affine transformation to standard basic space group setting

S * g(input) * S $^{-1}$ = g(standard),
where g is an augmented matrix for an operation in the superspace group.
Also, S * r(input) = r(standard),
where r is an augmented position vector, (x,y,z,t,u,1).

$$S = \begin{matrix} 0 & 1 & 0 & 0 & 0 & 0 \\ 1 & 0 & 0 & 0 & 0 & 0 \\ 0 & 0 & -1 & 0 & 0 & 0 \\ 0 & 0 & 0 & -1 & 0 & 0 \\ 0 & 0 & 0 & 0 & 1 & 0 \\ 0 & 0 & 0 & 0 & 0 & 1 \end{matrix} \quad S^{-1} = \begin{matrix} 0 & 1 & 0 & 0 & 0 & 0 \\ 1 & 0 & 0 & 0 & 0 & 0 \\ 0 & 0 & -1 & 0 & 0 & 0 \\ 0 & 0 & 0 & -1 & 0 & 0 \\ 0 & 0 & 0 & 0 & 1 & 0 \\ 0 & 0 & 0 & 0 & 0 & 1 \end{matrix}$$

a1' = a2	a3 = -a3'	a1* = a2*'	
a2' = a1		a2* = a1*'	q1 = -q1' = (-a,-a,0)
a3' = -a3	a1*' = a2*	a3* = -a3*'	q2 = q2' = (a,-2a,0)
	a2*' = a1*		
a1 = a2'	a3*' = -a3*	q1' = -q1 = (a,a,0)	
a2 = a1'		q2' = q2 = (-2a,a,0)	

transformssg

Input setting

Centering
none
Operators
(x-y,x,z+1/2,-u,t+u); (x,y,-z+1/2,t,u); (x,x-y,z,-u,-t); (y,x,z+1/2,t,-t-u); (-y,x-y,z,-t-u,t); (x-y,x,-z,-u,t+u); (-x+y,y,z,t+u,-u); (x,y,z,t,u); (x,x-y,-z+1/2,-u,-t); (y,x,-z,t,-t-u); (-y,x-y,-z+1/2,-t-u,t); (-x+y,y,-z+1/2,t+u,-u); (x-y,-y,z+1/2,-t-u,u); (y,-x+y,z+1/2,t+u,-t); (-y,-x,z,-t,t+u); (x-y,-y,-z,-t-u,u); (-x+y,-x,z,u,-t-u); (y,-x+y,-z,t+u,-t); (-y,-x,-z+1/2,-t,t+u); (-x+y,-x,-z+1/2,u,-t-u); (-x,-y,z+1/2,-t,-u); (-x,-x+y,z+1/2,u,t); (-x,-y,-z,-t,-u); (-x,-x+y,-z,u,t)
q vectors
$\mathbf{q}_1$=(-0.430,-0.430,0); $\mathbf{q}_2$=(0.860,-0.430,0)

New setting

Centering
none
Operators
(x-y,x,z+1/2,-u,t+u); (x,y,-z+1/2,t,u); (x,x-y,z,-u,-t); (y,x,z+1/2,t,-t-u); (-y,x-y,z,-t-u,t); (x-y,x,-z,-u,t+u); (-x+y,y,z,t+u,-u); (x,y,z,t,u); (x,x-y,-z+1/2,-u,-t); (y,x,-z,t,-t-u); (-y,x-y,-z+1/2,-t-u,t); (-x+y,y,-z+1/2,t+u,-u); (x-y,-y,z+1/2,-t-u,u); (y,-x+y,z+1/2,t+u,-t); (-y,-x,z,-t,t+u); (x-y,-y,-z,-t-u,u); (-x+y,-x,z,u,-t-u); (y,-x+y,-z,t+u,-t); (-y,-x,-z+1/2,-t,t+u); (-x+y,-x,-z+1/2,u,-t-u); (-x,-y,z+1/2,-t,-u); (-x,-x+y,z+1/2,u,t); (-x,-y,-z,-t,-u); (-x,-x+y,-z,u,t)
q vectors
$\mathbf{q}_1'$=(0.430,0.430,0); $\mathbf{q}_2'$=(-0.860,0.430,0)

Affine transformation to new setting

$$S = \begin{matrix} 1 & 0 & 0 & 0 & 0 & 0 \\ 0 & 1 & 0 & 0 & 0 & 0 \\ 0 & 0 & 1 & 0 & 0 & 0 \\ 0 & 0 & 0 & -1 & 0 & 0 \\ 0 & 0 & 0 & 0 & -1 & 0 \\ 0 & 0 & 0 & 0 & 0 & 1 \end{matrix} \qquad S^{-1} = \begin{matrix} 1 & 0 & 0 & 0 & 0 & 0 \\ 0 & 1 & 0 & 0 & 0 & 0 \\ 0 & 0 & 1 & 0 & 0 & 0 \\ 0 & 0 & 0 & -1 & 0 & 0 \\ 0 & 0 & 0 & 0 & -1 & 0 \\ 0 & 0 & 0 & 0 & 0 & 1 \end{matrix}$$

$g' = S * g * S^{-1}$, where g is an augmented matrix for an operation in the superspace group.
$\mathbf{r}' = S * \mathbf{r}$, where $\mathbf{r}$ is an augmented position vector, (x,y,z,t,u,1).

Basis vectors of the lattice
$\mathbf{a}_1' = \mathbf{a}_1$; $\mathbf{a}_2' = \mathbf{a}_2$; $\mathbf{a}_3' = \mathbf{a}_3$
$\mathbf{a}_1 = \mathbf{a}_1'$; $\mathbf{a}_2 = \mathbf{a}_2'$; $\mathbf{a}_3 = \mathbf{a}_3'$
Basis vectors of the reciprocal lattice
$\mathbf{a}_1^{*\prime} = \mathbf{a}_1^*$; $\mathbf{a}_2^{*\prime} = \mathbf{a}_2^*$; $\mathbf{a}_3^{*\prime} = \mathbf{a}_3^*$
$\mathbf{a}_1^* = \mathbf{a}_1^{*\prime}$; $\mathbf{a}_2^* = \mathbf{a}_2^{*\prime}$; $\mathbf{a}_3^* = \mathbf{a}_3^{*\prime}$

q vectors
$\mathbf{q}_1' = -\mathbf{q}_1$ = (0.430,0.430,0); $\mathbf{q}_2' = -\mathbf{q}_2$ = (-0.860,0.430,0)
$\mathbf{q}_1 = -\mathbf{q}_1'$ = (-0.430,-0.430,0); $\mathbf{q}_2 = -\mathbf{q}_2'$ = (0.860,-0.430,0)

Origin
$\boldsymbol{\tau}' = 0$
$\boldsymbol{\tau} = 0$

findssg P6$_3$/mmc(-p,2p,0)0000(-2p,p,0)0000

Generators of Yamamoto entered into findssg.

Input setting

Centering
none
Operators
(x,y,z,t,u); (-y,x-y,z,-u,t-u); (-x+y,-x,z,-t+u,-t); (-x,-y,z+1/2,-t,-u); (y,-x+y,z+1/2,u,-t+u); (x-y,x,z+1/2,t-u,t); (y,x,-z,-u,-t); (x-y,-y,-z,-t+u,u); (-x,-x+y,-z,t,t-u); (-y,-x,-z+1/2,u,t); (-x+y,y,-z+1/2,t-u,-u); (x,x-y,-z+1/2,-t,-t+u); (-x,-y,-z,-t,-u); (y,-x+y,-z,u,-t+u); (x-y,x,-z,t-u,t); (x,y,-z+1/2,t,u); (-y,x-y,-z+1/2,-u,t-u); (-x+y,-x,-z+1/2,-t+u,-t); (-y,-x,z,u,t); (-x+y,y,z,t-u,-u); (x,x-y,z,-t,-t+u); (y,x,z+1/2,-u,-t); (x-y,-y,z+1/2,-t+u,u); (-x,-x+y,z+1/2,t,t-u)

Standard settings

Superspace group: 194.2.83.4 P6_3/mmc(a,a,0)0000(-2a,a,0)0000 [Y:2.3354]
Bravais class: 2.83 P6/mmm(a,a,0)(-2a,a,0) [JJdW:2.83]
Transformation to supercentered setting: none
Modulation vectors: q1'=(a,a,0), q2'=(-2a,a,0)
Centering: (0,0,0,0,0)
Non-lattice generators: (x-y,x,z+1/2,-u,t+u); (x,y,-z+1/2,t,u); (x,x-y,z,-u,-t); (y,x,z+1/2,t,-t-u)
Non-lattice operators: (x,y,z,t,u); (x-y,x,z+1/2,-u,t+u); (-y,x-y,z,-t-u,t); (-x,-y,z+1/2,-t,-u); (-x+y,-x,z,u,-t-u); (y,-x+y,z+1/2,t+u,-t); (x-y,-y,-z,-t-u,u); (x,x-y,-z+1/2,-u,-t); (y,x,-z,t,-t-u); (-x+y,y,-z+1/2,t+u,-u); (-x,-x+y,-z,u,t); (-y,-x,-z+1/2,-t,t+u); (-x,-y,-z,-t,-u); (-x+y,-x,-z+1/2,u,-t-u); (y,-x+y,-z,t+u,-t); (x,y,-z+1/2,t,u); (x-y,x,-z,-u,t+u); (-y,x-y,-z+1/2,-t-u,t); (-x+y,y,z,t+u,-u); (-x,-x+y,z+1/2,u,t); (-y,-x,z,-t,t+u); (x-y,-y,z+1/2,-t-u,u); (x,x-y,z,-u,-t); (y,x,z+1/2,t,-t-u)
Reflection conditions: h2hlmm:l=2n; hhlm0:l=2n; 2kkl0n:l=2n

Affine transformation to standard basic space group setting

S * g(input) * S $^{-1}$ = g(standard),
where g is an augmented matrix for an operation in the superspace group.
Also, S * r(input) = r(standard),
where r is an augmented position vector, (x,y,z,t,u,1).

$$
S = \begin{pmatrix} 1&0&0&0&0&0\\ 0&1&0&0&0&0\\ 0&0&1&0&0&0\\ 0&0&0&1&-1&0\\ 0&0&0&0&1&0\\ 0&0&0&0&0&1 \end{pmatrix} \qquad S^{-1} = \begin{pmatrix} 1&0&0&0&0&0\\ 0&1&0&0&0&0\\ 0&0&1&0&0&0\\ 0&0&0&1&1&0\\ 0&0&0&0&1&0\\ 0&0&0&0&0&1 \end{pmatrix}
$$

a1' = a1
a2' = a2
a3' = a3

a1 = a1'
a2 = a2'
a3 = a3'

a1*' = a1*
a2*' = a2*
a3*' = a3*

a1* = a1*'
a2* = a2*'
a3* = a3*'

q1' = q1 - q2 = (a,a,0)
q2' = q2 = (-2a,a,0)

q1 = q1' + q2' = (-a,2a,0)
q2 = q2' = (-2a,a,0)

findssg $P6_3$/mmc(a,0,0)0000(0,a,0)0000

Generators of Yamamoto entered into findssg.

Input setting

Centering
none
Operators
(x-y,x,z+1/2,t-u,t); (x,x-y,z,-u,-t); (-x,-y,-z,-t,-u); (-y,x-y,z,-u,t-u); (y,x,z+1/2,t-u,-u); (-x+y,-x,-z+1/2,-t+u,-t); (x-y,-y,z+1/2,-t,-t+u); (x,y,z,t,u); (-x,-x+y,-z,u,t); (-y,-x,z,-t+u,u); (y,-x+y,z+1/2,u,-t+u); (-x+y,y,-z+1/2,t,t-u); (y,-x+y,-z,u,-t+u); (-y,-x,-z+1/2,-t+u,u); (y,x,-z,t-u,-u); (-y,x-y,-z+1/2,-u,t-u); (-x,-y,z+1/2,-t,-u); (-x+y,y,z,t,t-u); (x,y,-z+1/2,t,u); (x-y,-y,-z,-t,-t+u); (-x,-x+y,z+1/2,u,t); (-x+y,-x,z,-t+u,-t); (x,x-y,-z+1/2,-u,-t); (x-y,x,-z,t-u,t)

Standard settings

Superspace group: 194.2.82.3 P6_3/mmc(a,0,0)0000(-a,a,0)0000 [Y:2.3350]
Bravais class: 2.82 P6/mmm(a,0,0)(-a,a,0)   [JJdW:2.82]
Transformation to supercentered setting: none
Modulation vectors: q1'=(a,0,0), q2'=(-a,a,0)
Centering: (0,0,0,0,0)
Non-lattice generators: (x-y,x,z+1/2,-u,t+u); (x,y,-z+1/2,t,u); (x,x-y,z,t,-t-u); (y,x,z+1/2,t+u,-u)
Non-lattice operators: (x,y,z,t,u); (x-y,x,z+1/2,-u,t+u); (-y,x-y,z,-t-u,t); (-x,-y,z+1/2,-t,-u); (-x+y,-x,z,u,-t-u); (y,-x+y,z+1/2,t+u,-t); (x-y,-y,-z,-u,-t); (x,x-y,-z+1/2,t,-t-u); (y,x,-z,t+u,-u); (-x+y,y,-z+1/2,u,t); (-x,-x+y,-z,-t,t+u); (-y,-x,-z+1/2,-t-u,u); (-x,-y,-z,-t,-u); (-x+y,-x,-z+1/2,u,-t-u); (y,-x+y,-z,t+u,-t); (x,y,-z+1/2,t,u); (x-y,x,-z,-u,t+u); (-y,x-y,-z+1/2,-t-u,t); (-x+y,y,z,u,t); (-x,-x+y,z+1/2,-t,t+u); (-y,-x,z,-t-u,u); (x-y,-y,z+1/2,-u,-t); (x,x-y,z,t,-t-u); (y,x,z+1/2,t+u,-u)
Reflection conditions: h2hlm2m:l=2n; hhl2nn:l=2n; 2kklm-m:l=2n

Affine transformation to standard basic space group setting

S * g(input) * S^{-1} = g(standard),
where g is an augmented matrix for an operation in the superspace group.
Also, S * r(input) = r(standard),
where r is an augmented position vector, (x,y,z,t,u,1).

$$S = \begin{matrix} 0 & 1 & 0 & 0 & 0 & 0 \\ 1 & 0 & 0 & 0 & 0 & 0 \\ 0 & 0 & -1 & 0 & 0 & 0 \\ 0 & 0 & 0 & -1 & 0 & 0 \\ 0 & 0 & 0 & 0 & 1 & 0 \\ 0 & 0 & 0 & 0 & 0 & 1 \end{matrix} \qquad S^{-1} = \begin{matrix} 0 & 1 & 0 & 0 & 0 & 0 \\ 1 & 0 & 0 & 0 & 0 & 0 \\ 0 & 0 & -1 & 0 & 0 & 0 \\ 0 & 0 & 0 & -1 & 0 & 0 \\ 0 & 0 & 0 & 0 & 1 & 0 \\ 0 & 0 & 0 & 0 & 0 & 1 \end{matrix}$$

$a1' = a2$
$a2' = a1$
$a3' = -a3$

$a1 = a2'$
$a2 = a1'$
$a3 = -a3'$

$a1*' = a2*$
$a2*' = a1*$
$a3*' = -a3*$

$a1* = a2*'$
$a2* = a1*'$
$a3* = -a3*'$

$q1' = -q1 = (a,0,0)$
$q2' = q2 = (-a,a,0)$

$q1 = -q1' = (0,-a,0)$
$q2 = q2' = (a,-a,0)$
