# Supplementary material for: Equivalence of superspace groups
Source: Acta Crystallogr A. 2012 Nov 14;69(Pt 1):75–90. doi: 10.1107/S0108767312041657 (PMC3553647; doi:10.1107/S0108767312041657)
Supplement: Supplementary file 1 [file a-69-00075-sup1.zip › ssg2d_p63m_aa0_4hb_tase2.pdf]

176.2.80.1 $P6_3/m(a,b,0)000(-a-b,a,0)000$

Superspace group: 176.2.80.1 P6_3/m(a,b,0)00(-a-b,a,0)00 [Y:2.3286]
Bravais class: 2.80 P6/m(a,b,0)(-a-b,a,0)   [JJdW:2.80]
Transformation to supercentered setting: none
Modulation vectors: q1=(a,b,0), q2=(-a-b,a,0)
Centering: (0,0,0,0,0)
Non-lattice generators: (x-y,x,z+1/2,-u,t+u); (x,y,-z+1/2,t,u)
Non-lattice operators: (x,y,z,t,u); (x-y,x,z+1/2,-u,t+u); (-y,x-y,z,-t-u,t); (-x,-y,z+1/2,-t,-u); (-x+y,-x,z,u,-t-u); (y,-x+y,z+1/2,t+u,-t); (-x,-y,-z,-t,-u); (-x+y,-x,-z+1/2,u,-t-u); (y,-x+y,-z,t+u,-t); (x,y,-z+1/2,t,u); (x-y,x,-z,-u,t+u); (-y,x-y,-z+1/2,-t-u,t)
Reflection conditions: 00l00:l=2n

There is no supercentered setting, i.e. this is a primitive superspace lattice.
This is the symmetry of $4H_b$-$TaSe_2$:
J. Ludecke, S. van Smaalen, A. Spijkerman, J.L. de Boer and G.A. Wiegers, Phys. Rev. B 59, 6063-6071 (1999).

There is only one possible SSG.
-----

findssg P6$_3$/m(a,b,0)000(-a-b,a,0)000

Operators of Lüdecke et al. (1997) have been entered into findssg. Notice that lüdecke et al. (1997) have used q1=(3/13, 1/13, 0) ; q2=(-1/13, 4/13, 0).

Input setting

Centering
none
Operators
(-y,x-y,z,-u,t-u); (-x+y,-x,z,-t+u,-t); (-x,-y,z+1/2,-t,-u); (y,-x+y,z+1/2,u,-t+u); (x-y,x,z+1/2,t-u,t); (-x,-y,-z,-t,-u); (y,-x+y,-z,u,-t+u); (x-y,x,-z,t-u,t); (x,y,-z+1/2,t,u); (-y,x-y,-z+1/2,-u,t-u); (-x+y,-x,-z+1/2,-t+u,-t); (x,y,z,t,u)

Standard settings

Superspace group: 176.2.80.1 P6_3/m(a,b,0)00(-a-b,a,0)00 [Y:2.3286]
Bravais class: 2.80 P6/m(a,b,0)(-a-b,a,0) [JJdW:2.80]
Transformation to supercentered setting: none
Modulation vectors: q1'=(a,b,0), q2'=(-a-b,a,0)
Centering: (0,0,0,0,0)
Non-lattice generators: (x-y,x,z+1/2,-u,t+u); (x,y,-z+1/2,t,u)
Non-lattice operators: (x,y,z,t,u); (x-y,x,z+1/2,-u,t+u); (-y,x-y,z,-t-u,t); (-x,-y,z+1/2,-t,-u); (-x+y,-x,z,u,-t-u); (y,-x+y,z+1/2,t+u,-t); (-x,-y,-z,-t,-u); (-x+y,-x,-z+1/2,u,-t-u); (y,-x+y,-z,t+u,-t); (x,y,-z+1/2,t,u); (x-y,x,-z,-u,t+u); (-y,x-y,-z+1/2,-t-u,t)
Reflection conditions: 00l00:l=2n

Affine transformation to standard basic space group setting

S * g(input) * S $^{-1}$ = g(standard),
where g is an augmented matrix for an operation in the superspace group.
Also, S * r(input) = r(standard),
where r is an augmented position vector, (x,y,z,t,u,1).

$$S = \begin{pmatrix} 1&0&0&0&0&0\\ 0&1&0&0&0&0\\ 0&0&1&0&0&0\\ 0&0&0&1&0&0\\ 0&0&0&-1&1&0\\ 0&0&0&0&0&1 \end{pmatrix} \quad S^{-1} = \begin{pmatrix} 1&0&0&0&0&0\\ 0&1&0&0&0&0\\ 0&0&1&0&0&0\\ 0&0&0&1&0&0\\ 0&0&0&1&1&0\\ 0&0&0&0&0&1 \end{pmatrix}$$

a1' = a1	a3 = a3'	a1* = a1*'	
a2' = a2		a2* = a2*'	q1 = q1' = (a,b,0)
a3' = a3	a1*' = a1*	a3* = a3*'	q2 = q1' + q2' = (-b,a+b,0)
	a2*' = a2*		
a1 = a1'	a3*' = a3*	q1' = q1 = (a,b,0)	
a2 = a2'		q2' = -q1 + q2 = (-a-b,a,0)	
