# Supplementary material for: Equivalence of superspace groups
Source: Acta Crystallogr A. 2012 Nov 14;69(Pt 1):75–90. doi: 10.1107/S0108767312041657 (PMC3553647; doi:10.1107/S0108767312041657)
Supplement: Supplementary file 1 [file a-69-00075-sup1.zip › ssg2d_pmcn_0bg_sm23cr2s4.pdf]

62.2.50.22 Pbnm(0,b,g)000(0,-b,g)000

Superspace group: 62.2.50.22 Pbnm(0,b,g)000(0,-b,g)000 [Y:2.1937]
Bravais class: 2.50 Pmmm(0,b,g)(0,-b,g) [JJdW:2.50]
Transformation to supercentered setting: A1=a1, A2=a2, A3=a3, A4=a4-a5, A5=a4+a5

BASIC SPACE GROUP SETTING
Modulation vectors: q1=(0,b,g), q2=(0,-b,g)
Centering: (0,0,0,0,0)
Non-lattice generators: (-x+1/2,y+1/2,z,t,u); (x+1/2,-y+1/2,z+1/2,u,t); (x,y,-z+1/2,-u,-t)
Non-lattice operators: (x,y,z,t,u); (-x+1/2,y+1/2,-z+1/2,-u,-t); (-x,-y,z+1/2,u,t); (x+1/2,-y+1/2,-z,-t,-u); (-x,-y,-z,-t,-u); (x+1/2,-y+1/2,z+1/2,u,t); (x,y,-z+1/2,-u,-t); (-x+1/2,y+1/2,z,t,u)

SUPERCENTERED SETTING
Modulation vectors: Q1=(0,B,0), Q2=(0,0,G), where B=b, G=g
Centering: (0,0,0,0,0); (0,0,0,1/2,1/2)
Non-lattice generators: (-X+1/2,Y+1/2,Z,T,U); (X+1/2,-Y+1/2,Z+1/2,-T,U); (X,Y,-Z+1/2,T,-U)
Non-lattice operators: (X,Y,Z,T,U); (-X+1/2,Y+1/2,-Z+1/2,T,-U); (-X,-Y,Z+1/2,-T,U); (X+1/2,-Y+1/2,-Z,-T,-U); (-X,-Y,-Z,-T,-U); (X+1/2,-Y+1/2,Z+1/2,-T,U); (X,Y,-Z+1/2,T,-U); (-X+1/2,Y+1/2,Z,T,U)
Reflection conditions: HKLMN:M+N=2n; 0KLMN:K=2n; H0L0N:H+L=2n
-----

This is the symmetry of $Sm_{2/3}Cr_2S_4$ (Lafond 1997).
There is only one SSG with the same BSG in this Bravais class.

-----

transformssg

Input setting

Centering
none
Operators
(-x+1/2,y+1/2,z,t,u); (x+1/2,-y+1/2,z+1/2,u,t); (x,y,-z+1/2,-u,-t); (x,y,z,t,u); (-x,-y,z+1/2,u,t); (-x+1/2,y+1/2,-z+1/2,-u,-t); (x+1/2,-y+1/2,-z,-t,-u); (-x,-y,-z,-t,-u)
q vectors
$\mathbf{q}_1$=(0,1.322,0.333); $\mathbf{q}_2$=(0,-1.332,0.333)

New setting

Centering
none
Operators
(-x+1/2,y+1/2,z,t+1,u-1); (x+1/2,-y+1/2,z+1/2,u+1,t-1); (x,y,-z+1/2,-u,-t); (x,y,z,t,u); (-x,-y,z+1/2,u,t); (-x+1/2,y+1/2,-z+1/2,-u+1,-t-1); (x+1/2,-y+1/2,-z,-t+1,-u-1); (-x,-y,-z,-t,-u)
q vectors
$\mathbf{q}_1$'=(0,0.668,0.333); $\mathbf{q}_2$'=(0,-0.678,0.333)

Affine transformation to new setting

$$S = \begin{matrix} 1 & 0 & 0 & 0 & 0 & 0 \\ 0 & 1 & 0 & 0 & 0 & 0 \\ 0 & 0 & 1 & 0 & 0 & 0 \\ 0 & 2 & 0 & 0 & 1 & 0 \\ 0 & -2 & 0 & 1 & 0 & 0 \\ 0 & 0 & 0 & 0 & 0 & 1 \end{matrix} \quad S^{-1} = \begin{matrix} 1 & 0 & 0 & 0 & 0 & 0 \\ 0 & 1 & 0 & 0 & 0 & 0 \\ 0 & 0 & 1 & 0 & 0 & 0 \\ 0 & 2 & 0 & 0 & 1 & 0 \\ 0 & -2 & 0 & 1 & 0 & 0 \\ 0 & 0 & 0 & 0 & 0 & 1 \end{matrix}$$

g' = S * g * S $^{-1}$, where g is an augmented matrix for an operation in the superspace group.
r' = S * **r**, where **r** is an augmented position vector, (x,y,z,t,u,1).
Basis vectors of the lattice
$\mathbf{a}_1' = \mathbf{a}_1$; $\mathbf{a}_2' = \mathbf{a}_2$; $\mathbf{a}_3' = \mathbf{a}_3$
$\mathbf{a}_1 = \mathbf{a}_1'$; $\mathbf{a}_2 = \mathbf{a}_2'$; $\mathbf{a}_3 = \mathbf{a}_3'$
Basis vectors of the reciprocal lattice
$\mathbf{a}_1{*}' = \mathbf{a}_1{*}$; $\mathbf{a}_2{*}' = \mathbf{a}_2{*}$; $\mathbf{a}_3{*}' = \mathbf{a}_3{*}$
$\mathbf{a}_1{*} = \mathbf{a}_1{*}'$; $\mathbf{a}_2{*} = \mathbf{a}_2{*}'$; $\mathbf{a}_3{*} = \mathbf{a}_3{*}'$
q vectors
$\mathbf{q}_1' = \mathbf{q}_2 + 2\ \mathbf{a}_2{*}$ = (0,0.668,0.333); $\mathbf{q}_2' = \mathbf{q}_1 - 2\ \mathbf{a}_2{*}$ = (0,-0.678,0.333)
$\mathbf{q}_1 = \mathbf{q}_2' + 2\ \mathbf{a}_2{*}'$ = (0,1.322,0.333); $\mathbf{q}_2 = \mathbf{q}_1' - 2\ \mathbf{a}_2{*}'$ = (0,-1.332,0.333)
Origin
$\boldsymbol{\tau}' = 0$
$\boldsymbol{\tau} = 0$

findssg Pbnm(0,b,g)000(0,-b,g)000

Generators of the standard BSG setting have been entered into findssg.

Input setting

Centering
none
Operators
(-x+1/2,y+1/2,z,t,u); (x+1/2,-y+1/2,z+1/2,u,t); (x,y,-z+1/2,-u,-t); (x,y,z,t,u); (-x,-y,z+1/2,u,t); (-x+1/2,y+1/2,-z+1/2,-u,-t); (x+1/2,-y+1/2,-z,-t,-u); (-x,-y,-z,-t,-u)

Standard settings

Superspace group: 62.2.50.22 Pbnm(0,b,g)000(0,-b,g)000 [Y:2.1937]
Bravais class: 2.50 Pmmm(0,b,g)(0,-b,g) [JJdW:2.50]
Transformation to supercentered setting: A1=a1, A2=a2, A3=a3, A4=a4-a5, A5=a4+a5

BASIC SPACE GROUP SETTING
Modulation vectors: q1'=(0,b,g), q2'=(0,-b,g)
Centering: (0,0,0,0,0)
Non-lattice generators: (-x+1/2,y+1/2,z,t,u); (x+1/2,-y+1/2,z+1/2,u,t); (x,y,-z+1/2,-u,-t)
Non-lattice operators: (x,y,z,t,u); (-x+1/2,y+1/2,-z+1/2,-u,-t); (-x,-y,z+1/2,u,t); (x+1/2,-y+1/2,-z,-t,-u); (-x,-y,-z,-t,-u); (x+1/2,-y+1/2,z+1/2,u,t); (x,y,-z+1/2,-u,-t); (-x+1/2,y+1/2,z,t,u)

SUPERCENTERED SETTING
Modulation vectors: Q1'=(0,B,0), Q2'=(0,0,G), where B=b, G=g
Centering: (0,0,0,0,0); (0,0,0,1/2,1/2)
Non-lattice generators: (-X+1/2,Y+1/2,Z,T,U); (X+1/2,-Y+1/2,Z+1/2,-T,U); (X,Y,-Z+1/2,T,-U)
Non-lattice operators: (X,Y,Z,T,U); (-X+1/2,Y+1/2,-Z+1/2,T,-U); (-X,-Y,Z+1/2,-T,U); (X+1/2,-Y+1/2,-Z,-T,-U); (-X,-Y,-Z,-T,-U); (X+1/2,-Y+1/2,Z+1/2,-T,U); (X,Y,-Z+1/2,T,-U); (-X+1/2,Y+1/2,Z,T,U)
Reflection conditions: HKLMN:M+N=2n; 0KLMN:K=2n; H0L0N:H+L=2n

Affine transformation to standard basic space group setting

$S * g(input) * S^{-1} = g(standard)$,
where g is an augmented matrix for an operation in the superspace group.
Also, S * r(input) = r(standard),
where r is an augmented position vector, (x,y,z,t,u,1).

$$S = \begin{pmatrix} 1&0&0&0&0&0\\ 0&1&0&0&0&0\\ 0&0&1&0&0&0\\ 0&0&0&1&0&0\\ 0&0&0&0&1&0\\ 0&0&0&0&0&1 \end{pmatrix} \quad S^{-1} = \begin{pmatrix} 1&0&0&0&0&0\\ 0&1&0&0&0&0\\ 0&0&1&0&0&0\\ 0&0&0&1&0&0\\ 0&0&0&0&1&0\\ 0&0&0&0&0&1 \end{pmatrix}$$

a1' = a1
a2' = a2
a3' = a3

a1 = a1'
a2 = a2'
a3 = a3'

a1*' = a1*
a2*' = a2*
a3*' = a3*

a1* = a1*'
a2* = a2*'
a3* = a3*'

q1' = q1 = (0,b,g)
q2' = q2 = (0,-b,g)

q1 = q1' = (0,b,g)
q2 = q2' = (0,-b,g)

findssg Xbnm(0,b,0)000(0,0,g)000

Generators of the supercentered setting have been entered into findssg

Input setting

Centering
(0,0,0,0,0); (0,0,0,1/2,1/2)
Operators
(-x+1/2,y+1/2,z,t,u); (x+1/2,-y+1/2,z+1/2,-t,u); (x,y,-z+1/2,t,-u); (x,y,z,t,u); (-x,-y,z+1/2,-t,u); (-x+1/2,y+1/2,-z+1/2,t,-u); (x+1/2,-y+1/2,-z,-t,-u); (-x,-y,-z,-t,-u)

Standard settings

Superspace group: 62.2.50.22 Pbnm(0,b,g)000(0,-b,g)000 [Y:2.1937]
Bravais class: 2.50 Pmmm(0,b,g)(0,-b,g) [JJdW:2.50]
Transformation to supercentered setting: A1=a1, A2=a2, A3=a3, A4=a4-a5, A5=a4+a5

BASIC SPACE GROUP SETTING
Modulation vectors: q1'=(0,b,g), q2'=(0,-b,g)
Centering: (0,0,0,0,0)
Non-lattice generators: (-x+1/2,y+1/2,z,t,u); (x+1/2,-y+1/2,z+1/2,u,t); (x,y,-z+1/2,-u,-t)
Non-lattice operators: (x,y,z,t,u); (-x+1/2,y+1/2,-z+1/2,-u,-t); (-x,-y,z+1/2,u,t); (x+1/2,-y+1/2,-z,-t,-u); (-x,-y,-z,-t,-u); (x+1/2,-y+1/2,z+1/2,u,t); (x,y,-z+1/2,-u,-t); (-x+1/2,y+1/2,z,t,u)

SUPERCENTERED SETTING
Modulation vectors: Q1'=(0,B,0), Q2'=(0,0,G), where B=b, G=g
Centering: (0,0,0,0,0); (0,0,0,1/2,1/2)
Non-lattice generators: (-X+1/2,Y+1/2,Z,T,U); (X+1/2,-Y+1/2,Z+1/2,-T,U); (X,Y,-Z+1/2,T,-U)
Non-lattice operators: (X,Y,Z,T,U); (-X+1/2,Y+1/2,-Z+1/2,T,-U); (-X,-Y,Z+1/2,-T,U); (X+1/2,-Y+1/2,-Z,-T,-U); (-X,-Y,-Z,-T,-U); (X+1/2,-Y+1/2,Z+1/2,-T,U); (X,Y,-Z+1/2,T,-U); (-X+1/2,Y+1/2,Z,T,U)
Reflection conditions: HKLMN:M+N=2n; 0KLMN:K=2n; H0L0N:H+L=2n

Affine transformation to standard basic space group setting

$S * g(input) * S^{-1} = g(standard)$,
where g is an augmented matrix for an operation in the superspace group.
Also, S * r(input) = r(standard),
where r is an augmented position vector, (x,y,z,t,u,1).

$$S = \begin{pmatrix} 1 & 0 & 0 & 0 & 0 & 0 \\ 0 & 1 & 0 & 0 & 0 & 0 \\ 0 & 0 & 1 & 0 & 0 & 0 \\ 0 & 0 & 0 & -1 & 1 & 0 \\ 0 & 0 & 0 & 1 & 1 & 0 \\ 0 & 0 & 0 & 0 & 0 & 1 \end{pmatrix} \quad S^{-1} = \begin{pmatrix} 1 & 0 & 0 & 0 & 0 & 0 \\ 0 & 1 & 0 & 0 & 0 & 0 \\ 0 & 0 & 1 & 0 & 0 & 0 \\ 0 & 0 & 0 & -1/2 & 1/2 & 0 \\ 0 & 0 & 0 & 1/2 & 1/2 & 0 \\ 0 & 0 & 0 & 0 & 0 & 1 \end{pmatrix}$$

a1' = a1
a2' = a2
a3' = a3

a1 = a1'
a2 = a2'
a3 = a3'

a1*' = a1*
a2*' = a2*
a3*' = a3*

a1* = a1*'
a2* = a2*'
a3* = a3*'

q1' = -q1 + q2 = (0,b,g)
q2' = q1 + q2 = (0,-b,g)

q1 = -1/2 q1' + 1/2 q2' = (0,-b,0)
q2 = 1/2 q1' + 1/2 q2' = (0,0,g)

findssg   Pmnb(a,b,0)gm(double dot)g

Generators of the BSG setting of Lafond Sm2/3Cr2S4 have been entered into findssg.

Input setting

Centering
none
Operators
(-x+1/2,y,z,u+1/2,t+1/2); (x+1/2,-y+1/2,z+1/2,-u,-t); (x,y+1/2,-z+1/2,t+1/2,u+1/2); (x,y,z,t,u); (-x,-y+1/2,z+1/2,-t+1/2,-u+1/2); (-x+1/2,y+1/2,-z+1/2,u,t); (x+1/2,-y,-z,-u+1/2,-t+1/2); (-x,-y,-z,-t,-u)

Standard settings

Superspace group: 62.2.50.22 Pbnm(0,b,g)000(0,-b,g)000 [Y:2.1937]
Bravais class: 2.50 Pmmm(0,b,g)(0,-b,g) [JJdW:2.50]
Transformation to supercentered setting: A1=a1, A2=a2, A3=a3, A4=a4-a5, A5=a4+a5

BASIC SPACE GROUP SETTING
Modulation vectors: q1'=(0,b,g), q2'=(0,-b,g)
Centering: (0,0,0,0,0)
Non-lattice generators: (-x+1/2,y+1/2,z,t,u); (x+1/2,-y+1/2,z+1/2,u,t); (x,y,-z+1/2,-u,-t)
Non-lattice operators: (x,y,z,t,u); (-x+1/2,y+1/2,-z+1/2,-u,-t); (-x,-y,z+1/2,u,t); (x+1/2,-y+1/2,-z,-t,-u); (-x,-y,-z,-t,-u); (x+1/2,-y+1/2,z+1/2,u,t); (x,y,-z+1/2,-u,-t); (-x+1/2,y+1/2,z,t,u)

SUPERCENTERED SETTING
Modulation vectors: Q1'=(0,B,0), Q2'=(0,0,G), where B=b, G=g
Centering: (0,0,0,0,0); (0,0,0,1/2,1/2)
Non-lattice generators: (-X+1/2,Y+1/2,Z,T,U); (X+1/2,-Y+1/2,Z+1/2,-T,U); (X,Y,-Z+1/2,T,-U)
Non-lattice operators: (X,Y,Z,T,U); (-X+1/2,Y+1/2,-Z+1/2,T,-U); (-X,-Y,Z+1/2,-T,U); (X+1/2,-Y+1/2,-Z,-T,-U); (-X,-Y,-Z,-T,-U); (X+1/2,-Y+1/2,Z+1/2,-T,U); (X,Y,-Z+1/2,T,-U); (-X+1/2,Y+1/2,Z,T,U)
Reflection conditions: HKLMN:M+N=2n; 0KLMN:K=2n; H0L0N:H+L=2n

Affine transformation to standard basic space group setting

$S * g(input) * S^{-1} = g(standard)$,
where g is an augmented matrix for an operation in the superspace group.
Also, S * r(input) = r(standard),
where r is an augmented position vector, (x,y,z,t,u,1).

$$S = \begin{pmatrix} 0 & 0 & 1 & 0 & 0 & 0 \\ 0 & 1 & 0 & 0 & 0 & 0 \\ -1 & 0 & 0 & 0 & 0 & 0 \\ 0 & -1 & 0 & -1 & 0 & 1/2 \\ 0 & 1 & 0 & 0 & 1 & 0 \\ 0 & 0 & 0 & 0 & 0 & 1 \end{pmatrix} \quad S^{-1} = \begin{pmatrix} 0 & 0 & -1 & 0 & 0 & 0 \\ 0 & 1 & 0 & 0 & 0 & 0 \\ 1 & 0 & 0 & 0 & 0 & 0 \\ 0 & -1 & 0 & -1 & 0 & 1/2 \\ 0 & -1 & 0 & 0 & 1 & 0 \\ 0 & 0 & 0 & 0 & 0 & 1 \end{pmatrix}$$

a1' = a3
a2' = a2
a3' = -a1

a1 = -a3'
a2 = a2'
a3 = a1'

a1*' = a3*
a2*' = a2*
a3*' = -a1*

a1* = -a3*'
a2* = a2*'
a3* = a1*'

q1' = -q1 - a2* = (0,b,g)
q2' = q2 + a2* = (0,-b,g)

q1 = -q1' - a2*' = (g,-b-1,0)
q2 = q2' - a2*' = (-g,-b-1,0)

From previous version of findssg:
Affine transformation to standard supercentered setting

T * g(input) * T^{-1} = g(standard),
where g is an augmented matrix for an operation in the superspace group.
Also, T * r(input) = r(standard),
where r is an augmented position vector, (x,y,z,t,u,1) or (X,Y,Z,T,U,1).

$$T = \begin{pmatrix} 0 & 0 & 1 & 0 & 0 & 0 \\ 0 & -1 & 0 & 0 & 0 & 0 \\ 1 & 0 & 0 & 0 & 0 & 0 \\ 0 & -1 & 0 & -1/2 & -1/2 & 1/4 \\ 0 & 0 & 0 & -1/2 & 1/2 & 1/4 \\ 0 & 0 & 0 & 0 & 0 & 1 \end{pmatrix} \quad T^{-1} = \begin{pmatrix} 0 & 0 & 1 & 0 & 0 & 0 \\ 0 & -1 & 0 & 0 & 0 & 0 \\ 1 & 0 & 0 & 0 & 0 & 0 \\ 0 & 1 & 0 & -1 & -1 & 1/2 \\ 0 & 1 & 0 & -1 & 1 & 0 \\ 0 & 0 & 0 & 0 & 0 & 1 \end{pmatrix}$$

findssg Pmnb(ab0)s0s(-ab0)s0s

Input setting

Centering
none
Operators
(-x+1/2,y,z,u+1/2,t+1/2); (x,y+1/2,-z+1/2,t+1/2,u+1/2); (-x,-y,-z,-t,-u); (x,y,z,t,u); (-x+1/2,y+1/2,-z+1/2,u,t); (x+1/2,-y,-z,-u+1/2,-t+1/2); (-x,-y+1/2,z+1/2,-t+1/2,-u+1/2); (x+1/2,-y+1/2,z+1/2,-u,-t)

Standard settings

Superspace group: 62.2.50.22 Pbnm(0,b,g)000(0,-b,g)000 [Y:2.1937]
Bravais class: 2.50 Pmmm(0,b,g)(0,-b,g) [JJdW:2.50]
Transformation to supercentered setting: A1=a1, A2=a2, A3=a3, A4=a4-a5, A5=a4+a5

BASIC SPACE GROUP SETTING
Modulation vectors: q1'=(0,b,g), q2'=(0,-b,g)
Centering: (0,0,0,0,0)
Non-lattice generators: (-x+1/2,y+1/2,z,t,u); (x+1/2,-y+1/2,z+1/2,u,t); (x,y,-z+1/2,-u,-t)
Non-lattice operators: (x,y,z,t,u); (-x+1/2,y+1/2,-z+1/2,-u,-t); (-x,-y,z+1/2,u,t); (x+1/2,-y+1/2,-z,-t,-u); (-x,-y,-z,-t,-u); (x+1/2,-y+1/2,z+1/2,u,t); (x,y,-z+1/2,-u,-t); (-x+1/2,y+1/2,z,t,u)

SUPERCENTERED SETTING
Modulation vectors: Q1'=(0,B,0), Q2'=(0,0,G), where B=b, G=g
Centering: (0,0,0,0,0); (0,0,0,1/2,1/2)
Non-lattice generators: (-X+1/2,Y+1/2,Z,T,U); (X+1/2,-Y+1/2,Z+1/2,-T,U); (X,Y,-Z+1/2,T,-U)
Non-lattice operators: (X,Y,Z,T,U); (-X+1/2,Y+1/2,-Z+1/2,T,-U); (-X,-Y,Z+1/2,-T,U); (X+1/2,-Y+1/2,-Z,-T,-U); (-X,-Y,-Z,-T,-U); (X+1/2,-Y+1/2,Z+1/2,-T,U); (X,Y,-Z+1/2,T,-U); (-X+1/2,Y+1/2,Z,T,U)
Reflection conditions: HKLMN:M+N=2n; 0KLMN:K=2n; H0L0N:H+L=2n

Affine transformation to standard basic space group setting

$S * g(input) * S^{-1} = g(standard)$,
where g is an augmented matrix for an operation in the superspace group.
Also, S * r(input) = r(standard),
where r is an augmented position vector, (x,y,z,t,u,1).

$$S = \begin{matrix} 0 & 0 & 1 & 0 & 0 & 0 \\ 0 & 1 & 0 & 0 & 0 & 0 \\ -1 & 0 & 0 & 0 & 0 & 0 \\ 0 & -1 & 0 & -1 & 0 & 1/2 \\ 0 & 1 & 0 & 0 & 1 & 0 \\ 0 & 0 & 0 & 0 & 0 & 1 \end{matrix} \quad S^{-1} = \begin{matrix} 0 & 0 & -1 & 0 & 0 & 0 \\ 0 & 1 & 0 & 0 & 0 & 0 \\ 1 & 0 & 0 & 0 & 0 & 0 \\ 0 & -1 & 0 & -1 & 0 & 1/2 \\ 0 & -1 & 0 & 0 & 1 & 0 \\ 0 & 0 & 0 & 0 & 0 & 1 \end{matrix}$$

a1' = a3
a2' = a2
a3' = -a1

a1 = -a3'
a2 = a2'
a3 = a1'

a1*' = a3*
a2*' = a2*
a3*' = -a1*

a1* = -a3*'
a2* = a2*'
a3* = a1*'

q1' = -q1 - a2* = (0,b,g)
q2' = q2 + a2* = (0,-b,g)

q1 = -q1' - a2*' = (g,-b-1,0)
q2 = q2' - a2*' = (-g,-b-1,0)

From previous version of findssg:
Affine transformation to standard supercentered setting

$T * g(input) * T^{-1} = g(standard)$,
where g is an augmented matrix for an operation in the superspace group.
Also, T * r(input) = r(standard),
where r is an augmented position vector, (x,y,z,t,u,1) or (X,Y,Z,T,U,1).

$$T = \begin{pmatrix} 0 & 0 & 1 & 0 & 0 & 0 \\ 0 & -1 & 0 & 0 & 0 & 0 \\ 1 & 0 & 0 & 0 & 0 & 0 \\ 0 & -1 & 0 & -1/2 & -1/2 & 1/4 \\ 0 & 0 & 0 & -1/2 & 1/2 & 1/4 \\ 0 & 0 & 0 & 0 & 0 & 1 \end{pmatrix} \qquad T^{-1} = \begin{pmatrix} 0 & 0 & 1 & 0 & 0 & 0 \\ 0 & -1 & 0 & 0 & 0 & 0 \\ 1 & 0 & 0 & 0 & 0 & 0 \\ 0 & 1 & 0 & -1 & -1 & 1/2 \\ 0 & 1 & 0 & -1 & 1 & 0 \\ 0 & 0 & 0 & 0 & 0 & 1 \end{pmatrix}$$

62.2.50.20 Pmcn(0,b,g)000(0,-b,g)000

Superspace group: 62.2.50.20 Pmcn(0,b,g)000(0,-b,g)000 [Y:2.1929]
Bravais class: 2.50 Pmmm(0,b,g)(0,-b,g) [JJdW:2.50]
Transformation to supercentered setting: A1=a1, A2=a2, A3=a3, A4=a4-a5, A5=a4+a5

BASIC SPACE GROUP SETTING
Modulation vectors: q1=(0,b,g), q2=(0,-b,g)
Centering: (0,0,0,0,0)
Non-lattice generators: (-x+1/2,y,z,t,u); (x,-y+1/2,z+1/2,u,t); (x+1/2,y+1/2,-z+1/2,-u,-t)
Non-lattice operators: (x,y,z,t,u); (-x+1/2,-y+1/2,z+1/2,u,t); (x+1/2,-y,-z,-t,-u); (-x,y+1/2,-z+1/2,-u,-t); (-x,-y,-z,-t,-u); (x+1/2,y+1/2,-z+1/2,-u,-t); (-x+1/2,y,z,t,u); (x,-y+1/2,z+1/2,u,t)

SUPERCENTERED SETTING
Modulation vectors: Q1=(0,B,0), Q2=(0,0,G), where B=b, G=g
Centering: (0,0,0,0,0); (0,0,0,1/2,1/2)
Non-lattice generators: (-X+1/2,Y,Z,T,U); (X,-Y+1/2,Z+1/2,-T,U); (X+1/2,Y+1/2,-Z+1/2,T,-U)
Non-lattice operators: (X,Y,Z,T,U); (-X+1/2,-Y+1/2,Z+1/2,-T,U); (X+1/2,-Y,-Z,-T,-U); (-X,Y+1/2,-Z+1/2,T,-U); (-X,-Y,-Z,-T,-U); (X+1/2,Y+1/2,-Z+1/2,T,-U); (-X+1/2,Y,Z,T,U); (X,-Y+1/2,Z+1/2,-T,U)
Reflection conditions: HKLMN:M+N=2n; HK0M0:H+K=2n; H0L0N:L=2n

-----

There is one more SSG, differing in intrinsic translation along the superspace dimensions:

Superspace group: 62.2.50.21 Pmcn(0,b,g)000(0,-b,g)s00 [Y:2.1932]
Bravais class: 2.50 Pmmm(0,b,g)(0,-b,g) [JJdW:2.50]
Transformation to supercentered setting: A1=a1, A2=a2, A3=a3, A4=a4-a5, A5=a4+a5

BASIC SPACE GROUP SETTING
Modulation vectors: q1=(0,b,g), q2=(0,-b,g)
Centering: (0,0,0,0,0)
Non-lattice generators: (-x+1/2,y,z,t+1/2,u+1/2); (x,-y+1/2,z+1/2,u,t); (x+1/2,y+1/2,-z+1/2,-u+1/2,-t+1/2)
Non-lattice operators: (x,y,z,t,u); (-x+1/2,-y+1/2,z+1/2,u+1/2,t+1/2); (x+1/2,-y,-z,-t+1/2,-u+1/2); (-x,y+1/2,-z+1/2,-u,-t); (-x,-y,-z,-t,-u); (x+1/2,y+1/2,-z+1/2,-u+1/2,-t+1/2); (-x+1/2,y,z,t+1/2,u+1/2); (x,-y+1/2,z+1/2,u,t)

SUPERCENTERED SETTING
Modulation vectors: Q1=(0,B,0), Q2=(0,0,G), where B=b, G=g
Centering: (0,0,0,0,0); (0,0,0,1/2,1/2)
Non-lattice generators: (-X+1/2,Y,Z,T,U+1/2); (X,-Y+1/2,Z+1/2,-T,U); (X+1/2,Y+1/2,-Z+1/2,T,-U+1/2)
Non-lattice operators: (X,Y,Z,T,U); (-X+1/2,-Y+1/2,Z+1/2,-T,U+1/2); (X+1/2,-Y,-Z,-T,-U+1/2); (-X,Y+1/2,-Z+1/2,T,-U); (-X,-Y,-Z,-T,-U); (X+1/2,Y+1/2,-Z+1/2,T,-U+1/2); (-X+1/2,Y,Z,T,U+1/2); (X,-Y+1/2,Z+1/2,-T,U)
Reflection conditions: HKLMN:M+N=2n; 0KLMN:N=2n; HK0M0:H+K=2n; H0L0N:L=2n

-----

findssg Pmcn(0,b,g)000(0,-b,g)000

The standard BSG setting has been given as input to findssg.

findssg

Input setting

Centering
none
Operators
(-x+1/2,y,z,t,u); (x,-y+1/2,z+1/2,u,t); (x+1/2,y+1/2,-z+1/2,-u,-t); (x,y,z,t,u); (-x+1/2,-y+1/2,z+1/2,u,t); (-x,y+1/2,-z+1/2,-u,-t); (x+1/2,-y,-z,-t,-u); (-x,-y,-z,-t,-u)

Standard settings

Superspace group: 62.2.50.20 Pmcn(0,b,g)000(0,-b,g)000 [Y:2.1929]
Bravais class: 2.50 Pmmm(0,b,g)(0,-b,g) [JJdW:2.50]
Transformation to supercentered setting: A1=a1, A2=a2, A3=a3, A4=a4-a5, A5=a4+a5

BASIC SPACE GROUP SETTING
Modulation vectors: q1'=(0,b,g), q2'=(0,-b,g)
Centering: (0,0,0,0,0)
Non-lattice generators: (-x+1/2,y,z,t,u); (x,-y+1/2,z+1/2,u,t); (x+1/2,y+1/2,-z+1/2,-u,-t)
Non-lattice operators: (x,y,z,t,u); (-x+1/2,-y+1/2,z+1/2,u,t); (x+1/2,-y,-z,-t,-u); (-x,y+1/2,-z+1/2,-u,-t); (-x,-y,-z,-t,-u); (x+1/2,y+1/2,-z+1/2,-u,-t); (-x+1/2,y,z,t,u); (x,-y+1/2,z+1/2,u,t)

SUPERCENTERED SETTING
Modulation vectors: Q1'=(0,B,0), Q2'=(0,0,G), where B=b, G=g
Centering: (0,0,0,0,0); (0,0,0,1/2,1/2)
Non-lattice generators: (-X+1/2,Y,Z,T,U); (X,-Y+1/2,Z+1/2,-T,U); (X+1/2,Y+1/2,-Z+1/2,T,-U)
Non-lattice operators: (X,Y,Z,T,U); (-X+1/2,-Y+1/2,Z+1/2,-T,U); (X+1/2,-Y,-Z,-T,-U); (-X,Y+1/2,-Z+1/2,T,-U); (-X,-Y,-Z,-T,-U); (X+1/2,Y+1/2,-Z+1/2,T,-U); (-X+1/2,Y,Z,T,U); (X,-Y+1/2,Z+1/2,-T,U)
Reflection conditions: HKLMN:M+N=2n; HK0M0:H+K=2n; H0L0N:L=2n

Affine transformation to standard basic space group setting

$S * g(input) * S^{-1} = g(standard)$,
where g is an augmented matrix for an operation in the superspace group.
Also, S * r(input) = r(standard),
where r is an augmented position vector, (x,y,z,t,u,1).

$$S = \begin{pmatrix} 1 & 0 & 0 & 0 & 0 & 0 \\ 0 & 1 & 0 & 0 & 0 & 0 \\ 0 & 0 & 1 & 0 & 0 & 0 \\ 0 & 0 & 0 & 1 & 0 & 0 \\ 0 & 0 & 0 & 0 & 1 & 0 \\ 0 & 0 & 0 & 0 & 0 & 1 \end{pmatrix} \quad S^{-1} = \begin{pmatrix} 1 & 0 & 0 & 0 & 0 & 0 \\ 0 & 1 & 0 & 0 & 0 & 0 \\ 0 & 0 & 1 & 0 & 0 & 0 \\ 0 & 0 & 0 & 1 & 0 & 0 \\ 0 & 0 & 0 & 0 & 1 & 0 \\ 0 & 0 & 0 & 0 & 0 & 1 \end{pmatrix}$$

a1' = a1
a2' = a2
a3' = a3

a1 = a1'
a2 = a2'
a3 = a3'

a1*' = a1*
a2*' = a2*
a3*' = a3*

a1* = a1*'
a2* = a2*'
a3* = a3*'

q1' = q1 = (0,b,g)
q2' = q2 = (0,-b,g)

q1 = q1' = (0,b,g)
q2 = q2' = (0,-b,g)

From previous version of findssg:
Affine transformation to standard supercentered setting

$T * g(input) * T^{-1} = g(standard)$,
where g is an augmented matrix for an operation in the superspace group.
Also, T * r(input) = r(standard),
where r is an augmented position vector, (x,y,z,t,u,1) or (X,Y,Z,T,U,1).

$$T = \begin{pmatrix} 1 & 0 & 0 & 0 & 0 & 0 \\ 0 & 1 & 0 & 0 & 0 & 0 \\ 0 & 0 & 1 & 0 & 0 & 0 \\ 0 & 0 & 0 & 1/2 & -1/2 & 0 \\ 0 & 0 & 0 & 1/2 & 1/2 & 0 \\ 0 & 0 & 0 & 0 & 0 & 1 \end{pmatrix} \quad T^{-1} = \begin{pmatrix} 1 & 0 & 0 & 0 & 0 & 0 \\ 0 & 1 & 0 & 0 & 0 & 0 \\ 0 & 0 & 1 & 0 & 0 & 0 \\ 0 & 0 & 0 & 1 & 1 & 0 \\ 0 & 0 & 0 & -1 & 1 & 0 \\ 0 & 0 & 0 & 0 & 0 & 1 \end{pmatrix}$$
