# Supplementary material for: Equivalence of superspace groups
Source: Acta Crystallogr A. 2012 Nov 14;69(Pt 1):75–90. doi: 10.1107/S0108767312041657 (PMC3553647; doi:10.1107/S0108767312041657)
Supplement: Supplementary file 1 [file a-69-00075-sup1.zip › ssg2d_trigonal_yamamoto.pdf]

Selection of trigonal superspace groups according to Yamamoto.

3168 R3(qp0,-pr0)0
(00000;2/31/31/300;1/32/32/300)
x,y,z,t,u; -y,x-y,z,-u,t-u; -x+y,-x,z,-t+u,-t;
hklmn:2h+k+l=3n hklmn:h+2k+2l=3n

3179 R-3(qp0,-pr0)0
(00000;2/31/31/300;1/32/32/300)
x,y,z,t,u; -y,x-y,z,-u,t-u; -x+y,-x,z,-t+u,-t; -x,-y,-z,-t,-u; y,-x+y,-z,u,-t+u; x-y,x,-z,t-u,t;
hklmn:2h+k+l=3n hklmn:h+2k+2l=3n

3186 R32(-p 2p 0,-2p p 0)0m
(00000;2/31/31/300;1/32/32/300)
x,y,z,t,u; -y,x-y,z,-u,t-u; -x+y,-x,z,-t+u,-t; y,x,-z,-u,-t; x-y,-y,-z,-t+u,u; -x,-x+y,-z,t,t-u;
hklmn:2h+k+l=3n hklmn:h+2k+2l=3n

3175 P3(r-q0,qp0)0
x,y,z,t,u; -y,x-y,z,-u,t-u; -x+y,-x,z,-t+u,-t;

3182 P-3(r-q0,qp0)0
x,y,z,t,u; -y,x-y,z,-u,t-u; -x+y,-x,z,-t+u,-t; -x,-y,-z,-t,-u; y,-x+y,-z,u,-t+u; x-y,x,-z,t-u,t;

3196 P312(-p2 p 0,-2p p 0)00m
x,y,z,t,u; -y,x-y,z,-u,t-u; -x+y,-x,z,-t+u,-t; -y,-x,-z,u,t; -x+y,y,-z,t-u,-u; x,x-y,-z,-t,-t+u;

3202 P321(-p 2p 0,-2p p 0)0m0
x,y,z,t,u; -y,x-y,z,-u,t-u; -x+y,-x,z,-t+u,-t; y,x,-z,-u,-t; x-y,-y,-z,-t+u,u; -x,-x+y,-z,t,t-u;

-----

findssg P3(r –q 0, q p 0)

Generator of 3175 P3(r-q0,qp0)0 [Yamamoto] entered into findssg.

Input setting

Centering
none
Operators
(x,y,z,t,u); (-y,x-y,z,-u,t-u); (-x+y,-x,z,-t+u,-t)

Standard settings

Superspace group: 143.2.80.4 P3(a,b,0)0(-a-b,a,0)0 [Y:2.3175]
Bravais class: 2.80 P6/m(a,b,0)(-a-b,a,0) [JJdW:2.80]
Transformation to supercentered setting: none
Modulation vectors: q1'=(a,b,0), q2'=(-a-b,a,0)
Centering: (0,0,0,0,0)
Non-lattice generators: (-y,x-y,z,-t-u,t)
Non-lattice operators: (x,y,z,t,u); (-y,x-y,z,-t-u,t); (-x+y,-x,z,u,-t-u)
Reflection conditions: none

Affine transformation to standard basic space group setting

$S * g(input) * S^{-1} = g(standard)$,
where g is an augmented matrix for an operation in the superspace group.
Also, S * r(input) = r(standard),
where r is an augmented position vector, (x,y,z,t,u,1).

$$S = \begin{pmatrix} 0 & 1 & 0 & 0 & 0 & 0 \\ 1 & 0 & 0 & 0 & 0 & 0 \\ 0 & 0 & -1 & 0 & 0 & 0 \\ 0 & 0 & 0 & -1 & 0 & 0 \\ 0 & 0 & 0 & 0 & 1 & 0 \\ 0 & 0 & 0 & 0 & 0 & 1 \end{pmatrix} \quad S^{-1} = \begin{pmatrix} 0 & 1 & 0 & 0 & 0 & 0 \\ 1 & 0 & 0 & 0 & 0 & 0 \\ 0 & 0 & -1 & 0 & 0 & 0 \\ 0 & 0 & 0 & -1 & 0 & 0 \\ 0 & 0 & 0 & 0 & 1 & 0 \\ 0 & 0 & 0 & 0 & 0 & 1 \end{pmatrix}$$

a1' = a2		a3* = -a3*'
a2' = a1	a1*' = a2*	
a3' = -a3	a2*' = a1*	q1' = -q1 = (a,b,0)
	a3*' = -a3*	q2' = q2 = (-a-b,a,0)
a1 = a2'		
a2 = a1'	a1* = a2*'	q1 = -q1' = (-b,-a,0)
a3 = -a3'	a2* = a1*'	q2 = q2' = (a,-a-b,0)

findssg P-3(r –q 0, q p 0)

Generator of 3182 P-3(r-q0,qp0)0 [Yamamoto] entered into findssg.

Input setting

Centering
none
Operators
(-y,x-y,z,-u,t-u); (-x+y,-x,z,-t+u,-t); (-x,-y,-z,-t,-u); (y,-x+y,-z,u,-t+u); (x,y,z,t,u); (x-y,x,-z,t-u,t)

Standard settings

Superspace group: 147.2.80.3 P-3(a,b,0)0(-a-b,a,0)0 [Y:2.3182]
Bravais class: 2.80 P6/m(a,b,0)(-a-b,a,0) [JJdW:2.80]
Transformation to supercentered setting: none
Modulation vectors: q1'=(a,b,0), q2'=(-a-b,a,0)
Centering: (0,0,0,0,0)
Non-lattice generators: (y,-x+y,-z,t+u,-t)
Non-lattice operators: (x,y,z,t,u); (-y,x-y,z,-t-u,t); (-x+y,-x,z,u,-t-u); (-x,-y,-z,-t,-u); (y,-x+y,-z,t+u,-t); (x-y,x,-z,-u,t+u)
Reflection conditions: none

Affine transformation to standard basic space group setting

S * g(input) * S $^{-1}$ = g(standard),
where g is an augmented matrix for an operation in the superspace group.
Also, S * r(input) = r(standard),
where r is an augmented position vector, (x,y,z,t,u,1).

$$S = \begin{pmatrix} 0 & 1 & 0 & 0 & 0 & 0 \\ 1 & 0 & 0 & 0 & 0 & 0 \\ 0 & 0 & -1 & 0 & 0 & 0 \\ 0 & 0 & 0 & -1 & 0 & 0 \\ 0 & 0 & 0 & 0 & 1 & 0 \\ 0 & 0 & 0 & 0 & 0 & 1 \end{pmatrix} \quad S^{-1} = \begin{pmatrix} 0 & 1 & 0 & 0 & 0 & 0 \\ 1 & 0 & 0 & 0 & 0 & 0 \\ 0 & 0 & -1 & 0 & 0 & 0 \\ 0 & 0 & 0 & -1 & 0 & 0 \\ 0 & 0 & 0 & 0 & 1 & 0 \\ 0 & 0 & 0 & 0 & 0 & 1 \end{pmatrix}$$

a1' = a2		a3* = -a3*'
a2' = a1	a1*' = a2*	
a3' = -a3	a2*' = a1*	q1' = -q1 = (a,b,0)
	a3*' = -a3*	q2' = q2 = (-a-b,a,0)
a1 = a2'		
a2 = a1'	a1* = a2*'	q1 = -q1' = (-b,-a,0)
a3 = -a3'	a2* = a1*'	q2 = q2' = (a,-a-b,0)

findssg P312(-p 2p 0,-2p p 0)00m

Generator of 3196 P312(-p2p0,-2pp0)00m [Yamamoto] entered into findssg.

Input setting

Centering
none
Operators
(-y,x-y,z,-u,t-u); (-y,-x,-z,u,t); (-x+y,-x,z,-t+u,-t); (x,x-y,-z,-t,-t+u); (x,y,z,t,u); (-x+y,y,-z,t-u,-u)

Standard settings

Superspace group: 149.2.83.7 P312(a,a,0)000(-2a,a,0)000 [Y:2.3196]
Bravais class: 2.83 P6/mmm(a,a,0)(-2a,a,0) [JJdW:2.83]
Transformation to supercentered setting: none
Modulation vectors: q1'=(a,a,0), q2'=(-2a,a,0)
Centering: (0,0,0,0,0)
Non-lattice generators: (-y,x-y,z,-t-u,t); (x,y,z,t,u); (-y,-x,-z,-t,t+u)
Non-lattice operators: (x,y,z,t,u); (-y,x-y,z,-t-u,t); (-x+y,-x,z,u,-t-u); (x,x-y,-z,-u,-t); (-x+y,y,-z,t+u,-u); (-y,-x,-z,-t,t+u)
Reflection conditions: none

Affine transformation to standard basic space group setting

$S * g(input) * S^{-1} = g(standard)$,
where g is an augmented matrix for an operation in the superspace group.
Also, S * r(input) = r(standard),
where r is an augmented position vector, (x,y,z,t,u,1).

$$S = \begin{pmatrix} 1&0&0&0&0&0\\ 0&1&0&0&0&0\\ 0&0&1&0&0&0\\ 0&0&0&1&-1&0\\ 0&0&0&0&1&0\\ 0&0&0&0&0&1 \end{pmatrix} \quad S^{-1} = \begin{pmatrix} 1&0&0&0&0&0\\ 0&1&0&0&0&0\\ 0&0&1&0&0&0\\ 0&0&0&1&1&0\\ 0&0&0&0&1&0\\ 0&0&0&0&0&1 \end{pmatrix}$$

a1' = a1
a2' = a2
a3' = a3

a1 = a1'
a2 = a2'
a3 = a3'

a1*' = a1*
a2*' = a2*
a3*' = a3*

a1* = a1*'
a2* = a2*'
a3* = a3*'

q1' = q1 - q2 = (a,a,0)
q2' = q2 = (-2a,a,0)

q1 = q1' + q2' = (-a,2a,0)
q2 = q2' = (-2a,a,0)

findssg P321(-p 2p 0,-2p p 0)0m0

Generator of 3202 P321(-p 2p 0,-2p p 0)0m0 [Yamamoto] entered into findssg.

Input setting

Centering
none
Operators
(-y,x-y,z,-u,t-u); (-x+y,-x,z,-t+u,-t); (y,x,-z,-u,-t); (x-y,-y,-z,-t+u,u); (x,y,z,t,u); (-x,-x+y,-z,t,t-u)

Standard settings

Superspace group: 150.2.83.5 P321(a,a,0)000(-2a,a,0)000 [Y:2.3202]
Bravais class: 2.83 P6/mmm(a,a,0)(-2a,a,0) [JJdW:2.83]
Transformation to supercentered setting: none
Modulation vectors: q1'=(a,a,0), q2'=(-2a,a,0)
Centering: (0,0,0,0,0)
Non-lattice generators: (-y,x-y,z,-t-u,t); (-x,-x+y,-z,u,t); (x,y,z,t,u)
Non-lattice operators: (x,y,z,t,u); (-y,x-y,z,-t-u,t); (-x+y,-x,z,u,-t-u); (x-y,-y,-z,-t-u,u); (y,x,-z,t,-t-u); (-x,-x+y,-z,u,t)
Reflection conditions: none

Affine transformation to standard basic space group setting

$S * g(input) * S^{-1} = g(standard)$,
where g is an augmented matrix for an operation in the superspace group.
Also, S * r(input) = r(standard),
where r is an augmented position vector, (x,y,z,t,u,1).

$$S = \begin{pmatrix} 1 & 0 & 0 & 0 & 0 & 0 \\ 0 & 1 & 0 & 0 & 0 & 0 \\ 0 & 0 & 1 & 0 & 0 & 0 \\ 0 & 0 & 0 & 1 & -1 & 0 \\ 0 & 0 & 0 & 0 & 1 & 0 \\ 0 & 0 & 0 & 0 & 0 & 1 \end{pmatrix} \quad S^{-1} = \begin{pmatrix} 1 & 0 & 0 & 0 & 0 & 0 \\ 0 & 1 & 0 & 0 & 0 & 0 \\ 0 & 0 & 1 & 0 & 0 & 0 \\ 0 & 0 & 0 & 1 & 1 & 0 \\ 0 & 0 & 0 & 0 & 1 & 0 \\ 0 & 0 & 0 & 0 & 0 & 1 \end{pmatrix}$$

a1' = a1
a2' = a2
a3' = a3

a1 = a1'
a2 = a2'
a3 = a3'

a1*' = a1*
a2*' = a2*
a3*' = a3*

a1* = a1*'
a2* = a2*'
a3* = a3*'

q1' = q1 - q2 = (a,a,0)
q2' = q2 = (-2a,a,0)

q1 = q1' + q2' = (-a,2a,0)
q2 = q2' = (-2a,a,0)
