# Supplementary material for: Equivalence of superspace groups
Source: Acta Crystallogr A. 2012 Nov 14;69(Pt 1):75–90. doi: 10.1107/S0108767312041657 (PMC3553647; doi:10.1107/S0108767312041657)
Supplement: Supplementary file 1 [file a-69-00075-sup1.zip › ssg3d_fm3m_0bb_qsq_example.pdf]


225.3.212.6 Fm-3m(0,b,b)00q(b,0,b)s0q(b,b,0)00q

Superspace group: 225.3.212.6 Fm-3m(0,b,b)00q(b,0,b)s0q(b,b,0)00q [Y:none]
Bravais class: 3.212 Fm-3m(0,b,b)(b,0,b)(b,b,0) [JJdW:3.214]
Transformation to supercentered setting: A1=a1, A2=a2, A3=a3, A4=a5+a6, A5=a4+a6, A6=a4+a5

BASIC SPACE GROUP SETTING
Modulation vectors: q1=(0,b,b), q2=(b,0,b), q3=(b,b,0)
Centering: (0,0,0,0,0,0); (0,1/2,1/2,0,0,0); (1/2,0,1/2,0,0,0); (1/2,1/2,0,0,0,0)
Non-lattice generators: (x,y,-z,-u+v+1,-t+v+1/2,v+1/2); (-z,-x,-y,-v,-t,-u); (y,x,z,u+1/2,t+1/2,v+1/2)
Non-lattice operators: (x,y,z,t,u,v); (x,-y,-z,-t+1/2,-t+v,-t+u+1/2); (-x,y,-z,-u+v+1/2,-u+1/2,t-u); (-x,-y,z,u-v,t-v+1/2,-v+1/2); (y,z,x,u,v,t); (y,-z,-x,-u+1/2,t-u,-u+v+1/2); (-y,z,-x,t-v+1/2,-v+1/2,u-v); (-y,-z,x,-t+v,-t+u+1/2,-t+1/2); (z,x,y,v,t,u); (z,-x,-y,-v+1/2,u-v,t-v+1/2); (-z,x,-y,-t+u+1/2,-t+1/2,-t+v); (-z,-x,y,t-u,-u+v+1/2,-u+1/2); (-y,-x,-z,-u+1/2,-t+1/2,-v+1/2); (-y,x,z,u,u-v,-t+u+1/2); (y,-x,z,t-v+1/2,t,t-u); (y,x,-z,-t+v,-u+v+1/2,v); (-x,-z,-y,-t+1/2,-v+1/2,-u+1/2); (-x,z,y,t,t-u,t-v+1/2); (x,-z,y,-u+v+1/2,v,-t+v); (x,z,-y,u-v,-t+u+1/2,u); (-z,-y,-x,-v+1/2,-u+1/2,-t+1/2); (-z,y,x,v,-t+v,-u+v+1/2); (z,-y,x,-t+u+1/2,u,u-v); (z,y,-x,t-u,t-v+1/2,t); (-x,-y,-z,-t,-u,-v); (-x,y,z,t+1/2,t-v,t-u+1/2); (x,-y,z,u-v+1/2,u+1/2,-t+u); (x,y,-z,-u+v,-t+v+1/2,v+1/2); (-y,-z,-x,-u,-v,-t); (-y,z,x,u+1/2,-t+u,u-v+1/2); (y,-z,x,-t+v+1/2,v+1/2,-u+v); (y,z,-x,t-v,t-u+1/2,t+1/2); (-z,-x,-y,-v,-t,-u); (-z,x,y,v+1/2,-u+v,-t+v+1/2); (z,-x,y,t-u+1/2,t+1/2,t-v); (z,x,-y,-t+u,u-v+1/2,u+1/2); (y,x,z,u+1/2,t+1/2,v+1/2); (y,-x,-z,-u,-u+v,t-u+1/2); (-y,x,-z,-t+v+1/2,-t,-t+u); (-y,-x,z,t-v,u-v+1/2,-v); (x,z,y,t+1/2,v+1/2,u+1/2); (x,-z,-y,-t,-t+u,-t+v+1/2); (-x,z,-y,u-v+1/2,-v,t-v); (-x,-z,y,-u+v,t-u+1/2,-u); (z,y,x,v+1/2,u+1/2,t+1/2); (z,-y,-x,-v,t-v,u-v+1/2); (-z,y,-x,t-u+1/2,-u,-u+v); (-z,-y,x,-t+u,-t+v+1/2,-t)

SUPERCENTERED SETTING
Modulation vectors: Q1=(B,0,0), Q2=(0,B,0), Q3=(0,0,B), where B=b
Centering: (0,0,0,0,0,0); (0,1/2,1/2,0,0,0); (1/2,0,1/2,0,0,0); (1/2,1/2,0,0,0,0); (0,0,0,1/2,1/2,1/2); (0,1/2,1/2,1/2,1/2,1/2); (1/2,0,1/2,1/2,1/2,1/2); (1/2,1/2,0,1/2,1/2,1/2)
Non-lattice generators: (X,Y,-Z,T,U+1/2,-V+1/2); (-Z,-X,-Y,-V,-T,-U); (Y,X,Z,U+1/4,T+1/4,V+1/4)
Non-lattice operators: (X,Y,Z,T,U,V); (X,-Y,-Z,T,-U+1/2,-V); (-X,Y,-Z,-T,U,-V+1/2); (-X,-Y,Z,-T+1/2,-U,V); (Y,Z,X,U,V,T); (Y,-Z,-X,U,-V+1/2,-T); (-Y,Z,-X,-U,V,-T+1/2); (-Y,-Z,X,-U+1/2,-V,T); (Z,X,Y,V,T,U); (Z,-X,-Y,V,-T+1/2,-U); (-Z,X,-Y,-V,T,-U+1/2); (-Z,-X,Y,-V+1/2,-T,U); (-Y,-X,-Z,-U+1/4,-T+1/4,-V+1/4); (-Y,X,Z,-U+1/4,T+1/4,V+3/4); (Y,-X,Z,U+1/4,-T+3/4,V+3/4); (Y,X,-Z,U+1/4,T+3/4,-V+1/4); (-X,-Z,-Y,-T+1/4,-V+1/4,-U+1/4); (-X,Z,Y,-T+1/4,V+1/4,U+3/4); (X,-Z,Y,T+1/4,-V+3/4,U+3/4); (X,Z,-Y,T+1/4,V+3/4,-U+1/4); (-Z,-Y,-X,-V+1/4,-U+1/4,-T+1/4); (-Z,Y,X,-V+1/4,U+1/4,T+3/4); (Z,-Y,X,V+1/4,-U+3/4,T+3/4); (Z,Y,-X,V+1/4,U+3/4,-T+1/4); (-X,-Y,-Z,-T,-U,-V); (-X,Y,Z,-T,U+1/2,V); (X,-Y,Z,T,-U,V+1/2); (X,Y,-Z,T+1/2,U,-V); (-Y,-Z,-X,-U,-V,-T); (-Y,Z,X,-U,V+1/2,T); (Y,-Z,X,U,-V,T+1/2); (Y,Z,-X,U+1/2,V,-T); (-Z,-X,-Y,-V,-T,-U); (-Z,X,Y,-V,T+1/2,U); (Z,-X,Y,V,-T,U+1/2); (Z,X,-Y,V+1/2,T,-U); (Y,X,Z,U+1/4,T+1/4,V+1/4); (Y,-X,-Z,U+1/4,-T+1/4,-V+3/4); (-Y,X,-Z,-U+1/4,T+3/4,-V+3/4); (-Y,-X,Z,-U+1/4,-T+3/4,V+1/4); (X,Z,Y,T+1/4,V+1/4,U+1/4); (X,-Z,-Y,T+1/4,-V+1/4,-U+3/4); (-X,Z,-Y,-T+1/4,V+3/4,-U+3/4); (-X,-Z,Y,-T+1/4,-V+3/4,U+1/4); (Z,Y,X,V+1/4,U+1/4,T+1/4); (Z,-Y,-X,V+1/4,-U+1/4,-T+3/4); (-Z,Y,-X,-V+1/4,U+3/4,-T+3/4); (-Z,-Y,X,-V+1/4,-U+3/4,T+1/4)
Reflection conditions: HKLMNP:H+K=2n; HKLMNP:H+L=2n; HKLMNP:M+N+P=2n;

HHLMMP:2M+P=4n; H-HLM-MP:2M+P=4n; HKHMNM:2M+N=4n; HK-HMN-M:2M-N=4n; HKKMNN:M+2N=4n; HK-KMN-N:M+2N=4n; HK0MN0:M=2n; H0LM0P:P=2n; 0KL0NP:N=2n

-----

Two SSG exist with q-vectors forming a I-centered lattice and different intrinsic translational components:

225.3.212.5 Fm-3m(0,b,b)000(b,0,b)000(b,b,0)000
225.3.212.6 Fm-3m(0,b,b)00q(b,0,b)s0q(b,b,0)00q

-----

findssg Fm-3m(0,b,b)00q(b,0,b)s0q(b,b,0)00q

Generators of the standard BSG setting have been entered into findssg.

Input setting

Centering
(0,0,0,0,0,0); (1/2,1/2,0,0,0,0); (1/2,0,1/2,0,0,0); (0,1/2,1/2,0,0,0)
Operators
(x,y,-z,-u+v,-t+v+1/2,v+1/2); (-z,-x,-y,-v,-t,-u); (y,x,z,u+1/2,t+1/2,v+1/2); (x,y,z,t,u,v); (-z,-x,y,t-u,-u+v+1/2,-u+1/2); (y,x,-z,-t+v,-u+v+1/2,v); (z,-x,-y,-v+1/2,u-v,t-v+1/2); (y,z,x,u,v,t); (-z,-y,-x,-v+1/2,-u+1/2,-t+1/2); (-y,z,x,u+1/2,-t+u,u-v+1/2); (z,-y,-x,-v,t-v,u-v+1/2); (-x,-z,-y,-t+1/2,-v+1/2,-u+1/2); (-x,-z,y,-u+v,t-u+1/2,-u); (-x,z,-y,u-v+1/2,-v,t-v); (z,y,x,v+1/2,u+1/2,t+1/2); (-y,-z,-x,-u,-v,-t); (z,-y,x,-t+u+1/2,u,u-v); (-y,z,-x,t-v+1/2,-v+1/2,u-v); (z,-x,y,t-u+1/2,t+1/2,t-v); (y,z,-x,t-v,t-u+1/2,t+1/2); (-z,-y,x,-t+u,-t+v+1/2,-t); (-x,z,y,t,t-u,t-v+1/2); (z,y,-x,t-u,t-v+1/2,t); (-y,-z,x,-t+v,-t+u+1/2,-t+1/2); (y,-z,x,-t+v+1/2,v+1/2,-u+v); (-x,-y,-z,-t,-u,-v); (x,z,y,t+1/2,v+1/2,u+1/2); (-x,y,-z,-u+v+1/2,-u+1/2,t-u); (x,-z,y,-u+v+1/2,v,-t+v); (-y,x,z,u,u-v,-t+u+1/2); (x,-y,-z,-t+1/2,-t+v,-t+u+1/2); (-y,x,-z,-t+v+1/2,-t,-t+u); (x,-z,-y,-t,-t+u,-t+v+1/2); (-x,y,z,t+1/2,t-v,t-u+1/2); (-z,y,x,v,-t+v,-u+v+1/2); (-y,-x,-z,-u+1/2,-t+1/2,-v+1/2); (z,x,y,v,t,u); (y,-x,-z,-u,-u+v,t-u+1/2); (-z,x,y,v+1/2,-u+v,-t+v+1/2); (x,-y,z,u-v+1/2,u+1/2,-t+u); (-z,x,-y,-t+u+1/2,-t+1/2,-t+v); (y,-x,z,t-v+1/2,t,t-u); (y,-z,-x,-u+1/2,t-u,-u+v+1/2); (-x,-y,z,u-v,t-v+1/2,-v+1/2); (x,z,-y,u-v,-t+u+1/2,u); (z,x,-y,-t+u,u-v+1/2,u+1/2); (-y,-x,z,t-v,u-v+1/2,-v); (-z,y,-x,t-u+1/2,-u,-u+v)

Standard settings

Superspace group: 225.3.212.6 Fm-3m(0,b,b)00q(b,0,b)s0q(b,b,0)00q [Y:none]
Bravais class: 3.212 Fm-3m(0,b,b)(b,0,b)(b,b,0) [JJdW:3.214]
Transformation to supercentered setting: A1=a1, A2=a2, A3=a3, A4=a5+a6, A5=a4+a6, A6=a4+a5

BASIC SPACE GROUP SETTING
Modulation vectors: q1'=(0,b,b), q2'=(b,0,b), q3'=(b,b,0)
Centering: (0,0,0,0,0,0); (0,1/2,1/2,0,0,0); (1/2,0,1/2,0,0,0); (1/2,1/2,0,0,0,0)
Non-lattice generators: (x,y,-z,-u+v+1,-t+v+1/2,v+1/2); (-z,-x,-y,-v,-t,-u); (y,x,z,u+1/2,t+1/2,v+1/2)
Non-lattice operators: (x,y,z,t,u,v); (x,-y,-z,-t+1/2,-t+v,-t+u+1/2); (-x,y,-z,-u+v+1/2,-u+1/2,t-u); (-x,-y,z,u-v,t-v+1/2,-v+1/2); (y,z,x,u,v,t); (y,-z,-x,-u+1/2,t-u,-u+v+1/2); (-y,z,-x,t-v+1/2,-v+1/2,u-v); (-y,-z,x,-t+v,-t+u+1/2,-t+1/2); (z,x,y,v,t,u); (z,-x,-y,-v+1/2,u-v,t-v+1/2); (-z,x,-y,-t+u+1/2,-t+1/2,-t+v); (-z,-x,y,t-u,-u+v+1/2,-u+1/2); (-y,-x,-z,-u+1/2,-t+1/2,-v+1/2); (-y,x,z,u,u-v,-t+u+1/2); (y,-x,z,t-v+1/2,t,t-u); (y,x,-z,-t+v,-u+v+1/2,v); (-x,-z,-y,-t+1/2,-v+1/2,-u+1/2); (-x,z,y,t,t-u,t-v+1/2); (x,-z,y,-u+v+1/2,v,-t+v); (x,z,-y,u-v,-t+u+1/2,u); (-z,-y,-x,-v+1/2,-u+1/2,-t+1/2); (-z,y,x,v,-t+v,-u+v+1/2); (z,-y,x,-t+u+1/2,u,u-v); (z,y,-x,t-u,t-v+1/2,t); (-x,-y,-z,-t,-u,-v); (-x,y,z,t+1/2,t-v,t-u+1/2); (x,-y,z,u-v+1/2,u+1/2,-t+u); (x,y,-z,-u+v,-t+v+1/2,v+1/2); (-y,-z,-x,-u,-v,-t); (-y,z,x,u+1/2,-t+u,u-v+1/2); (y,-z,x,-t+v+1/2,v+1/2,-u+v); (y,z,-x,t-v,t-u+1/2,t+1/2); (-z,-x,-y,-v,-t,-u); (-z,x,y,v+1/2,-u+v,-t+v+1/2); (z,-x,y,t-u+1/2,t+1/2,t-v); (z,x,-y,-t+u,u-v+1/2,u+1/2); (y,x,z,u+1/2,t+1/2,v+1/2); (y,-x,-z,-u,-u+v,t-u+1/2); (-y,x,-z,-t+v+1/2,-t,-t+u); (-y,-x,z,t-v,u-v+1/2,-v); (x,z,y,t+1/2,v+1/2,u+1/2); (x,-z,-y,-t,-t+u,-t+v+1/2); (-x,z,-y,u-v+1/2,-v,t-v); (-x,-z,y,-u+v,t-u+1/2,-u); (z,y,x,v+1/2,u+1/2,t+1/2); (z,-y,-x,-v,t-v,u-v+1/2); (-z,y,-x,t-u+1/2,-u,-u+v); (-z,-y,x,-t+u,-t+v+1/2,-t)

SUPERCENTERED SETTING
Modulation vectors: Q1'=(B,0,0), Q2'=(0,B,0), Q3'=(0,0,B), where B=b
Centering: (0,0,0,0,0,0); (0,1/2,1/2,0,0,0); (1/2,0,1/2,0,0,0); (1/2,1/2,0,0,0,0); (0,0,0,1/2,1/2,1/2); (0,1/2,1/2,1/2,1/2,1/2); (1/2,0,1/2,1/2,1/2,1/2); (1/2,1/2,0,1/2,1/2,1/2)
Non-lattice generators: (X,Y,-Z,T,U+1/2,-V+1/2); (-Z,-X,-Y,-V,-T,-U); (Y,X,Z,U+1/4,T+1/4,V+1/4)
Non-lattice operators: (X,Y,Z,T,U,V); (X,-Y,-Z,T,-U+1/2,-V); (-X,Y,-Z,-T,U,-V+1/2); (-X,-Y,Z,-T+1/2,-U,V); (Y,Z,X,U,V,T); (Y,-Z,-X,U,-V+1/2,-T); (-Y,Z,-X,-U,V,-T+1/2); (-Y,-Z,X,-U+1/2,-V,T); (Z,X,Y,V,T,U); (Z,-X,-Y,V,-T+1/2,-U); (-Z,X,-Y,-V,T,-U+1/2); (-Z,-X,Y,-V+1/2,-T,U); (-Y,-X,-Z,-U+1/4,-T+1/4,-V+1/4); (-Y,X,Z,-U+1/4,T+1/4,V+3/4); (Y,-X,Z,U+1/4,-T+3/4,V+3/4); (Y,X,-Z,U+1/4,T+3/4,-V+1/4); (-X,-Z,-Y,-T+1/4,-V+1/4,-U+1/4); (-X,Z,Y,-T+1/4,V+1/4,U+3/4); (X,-Z,Y,T+1/4,-V+3/4,U+3/4); (X,Z,-Y,T+1/4,V+3/4,-U+1/4); (-Z,-Y,-X,-V+1/4,-U+1/4,-T+1/4); (-Z,Y,X,-V+1/4,U+1/4,T+3/4); (Z,-Y,X,V+1/4,-U+3/4,T+3/4); (Z,Y,-X,V+1/4,U+3/4,-T+1/4); (-X,-Y,-Z,-T,-U,-V); (-X,Y,Z,-T,U+1/2,V); (X,-Y,Z,T,-U,V+1/2); (X,Y,-Z,T+1/2,U,-V); (-Y,-Z,-X,-U,-V,-T); (-Y,Z,X,-U,V+1/2,T); (Y,-Z,X,U,-V,T+1/2); (Y,Z,-X,U+1/2,V,-T); (-Z,-X,-Y,-V,-T,-U); (-Z,X,Y,-V,T+1/2,U); (Z,-X,Y,V,-T,U+1/2); (Z,X,-Y,V+1/2,T,-U); (Y,X,Z,U+1/4,T+1/4,V+1/4); (Y,-X,-Z,U+1/4,-T+1/4,-V+3/4); (-Y,X,-Z,-U+1/4,T+3/4,-V+3/4); (-Y,-X,Z,-U+1/4,-T+3/4,V+1/4); (X,Z,Y,T+1/4,V+1/4,U+1/4); (X,-Z,-Y,T+1/4,-V+1/4,-U+3/4); (-X,Z,-Y,-T+1/4,V+3/4,-U+3/4); (-X,-Z,Y,-T+1/4,-V+3/4,U+1/4); (Z,Y,X,V+1/4,U+1/4,T+1/4); (Z,-Y,-X,V+1/4,-U+1/4,-T+3/4); (-Z,Y,-X,-V+1/4,U+3/4,-T+3/4); (-Z,-Y,X,-V+1/4,-U+3/4,T+1/4)
Reflection conditions: HKLMNP:H+K=2n; HKLMNP:H+L=2n; HKLMNP:M+N+P=2n; HHLMMP:2M+P=4n; H-HLM-MP:2M+P=4n; HKHMNM:2M+N=4n; HK-HMN-M:2M-N=4n; HKKMNN:M+2N=4n; HK-KMN-N:M+2N=4n; HK0MN0:M=2n; H0LM0P:P=2n; 0KL0NP:N=2n

Affine transformation to standard basic space group setting

S * g(input) * S $^{-1}$ = g(standard),
where g is an augmented matrix for an operation in the superspace group.
Also, S * r(input) = r(standard),
where r is an augmented position vector, (x,y,z,t,u,v,1).

$$S = \begin{matrix} 1&0&0&0&0&0&0 \\ 0&1&0&0&0&0&0 \\ 0&0&1&0&0&0&0 \\ 0&0&0&1&0&0&0 \\ 0&0&0&0&1&0&0 \\ 0&0&0&0&0&1&0 \\ 0&0&0&0&0&0&1 \end{matrix} \quad S^{-1} = \begin{matrix} 1&0&0&0&0&0&0 \\ 0&1&0&0&0&0&0 \\ 0&0&1&0&0&0&0 \\ 0&0&0&1&0&0&0 \\ 0&0&0&0&1&0&0 \\ 0&0&0&0&0&1&0 \\ 0&0&0&0&0&0&1 \end{matrix}$$

a1' = a1	a1*' = a1*	q1' = q1 = (0,b,b)
a2' = a2	a2*' = a2*	q2' = q2 = (b,0,b)
a3' = a3	a3*' = a3*	q3' = q3 = (b,b,0)

a1 = a1'	a1* = a1*'	q1 = q1' = (0,b,b)
a2 = a2'	a2* = a2*'	q2 = q2' = (b,0,b)
a3 = a3'	a3* = a3*'	q3 = q3' = (b,b,0)

findssg Fm-3m(0,b,b)00q(b,0,b)s0q(b,b,0)00q

Generators of the standard supercentered setting have been entered into findssg.

Input setting

Centering
(0,0,0,0,0,0); (1/2,1/2,0,0,0,0); (1/2,0,1/2,0,0,0); (0,1/2,1/2,0,0,0); (0,0,0,1/2,1/2,1/2); (1/2,1/2,0,1/2,1/2,1/2); (1/2,0,1/2,1/2,1/2,1/2); (0,1/2,1/2,1/2,1/2,1/2)
Operators
(x,y,-z,t,u+1/2,-v+1/2); (-z,-x,-y,-v,-t,-u); (y,x,z,u+1/4,t+1/4,v+1/4); (x,y,z,t,u,v); (-z,-x,y,-v,-t+1/2,u+1/2); (y,x,-z,u+1/4,t+3/4,-v+1/4); (z,-x,-y,v+1/2,-t,-u+1/2); (y,z,x,u,v,t); (-z,-y,-x,-v+3/4,-u+3/4,-t+3/4); (-y,z,x,-u+1/2,v,t+1/2); (z,-y,-x,v+3/4,-u+3/4,-t+1/4); (-x,-z,-y,-t+1/4,-v+1/4,-u+1/4); (-x,-z,y,-t+3/4,-v+1/4,u+3/4); (-x,z,-y,-t+1/4,v+3/4,-u+3/4); (z,y,x,v+1/4,u+1/4,t+1/4); (-y,-z,-x,-u,-v,-t); (z,-y,x,v+1/4,-u+3/4,t+3/4); (-y,z,-x,-u,v,-t+1/2); (z,-x,y,v+1/2,-t+1/2,u); (y,z,-x,u,v+1/2,-t+1/2); (-z,-y,x,-v+3/4,-u+1/4,t+3/4); (-x,z,y,-t+1/4,v+1/4,u+3/4); (z,y,-x,v+1/4,u+3/4,-t+1/4); (-y,-z,x,-u,-v+1/2,t+1/2); (y,-z,x,u+1/2,-v+1/2,t); (-x,-y,-z,-t,-u,-v); (x,z,y,t+1/4,v+1/4,u+1/4); (-x,y,-z,-t+1/2,u+1/2,-v); (x,-z,y,t+3/4,-v+1/4,u+1/4); (-y,x,z,-u+1/4,t+1/4,v+3/4); (x,-y,-z,t+1/2,-u,-v+1/2); (-y,x,-z,-u+1/4,t+3/4,-v+3/4); (x,-z,-y,t+3/4,-v+3/4,-u+1/4); (-x,y,z,-t+1/2,u,v+1/2); (-z,y,x,-v+3/4,u+3/4,t+1/4); (-y,-x,-z,-u+1/4,-t+1/4,-v+1/4); (z,x,y,v+1/2,t+1/2,u+1/2); (y,-x,-z,u+3/4,-t+3/4,-v+1/4); (-z,x,y,-v+1/2,t,u+1/2); (x,-y,z,t+1/2,-u+1/2,v); (-z,x,-y,-v,t,-u+1/2); (y,-x,z,u+1/4,-t+3/4,v+3/4); (y,-z,-x,u+1/2,-v,-t+1/2); (-x,-y,z,-t,-u+1/2,v+1/2); (x,z,-y,t+1/4,v+3/4,-u+1/4); (z,x,-y,v,t+1/2,-u+1/2); (-y,-x,z,-u+3/4,-t+1/4,v+3/4); (-z,y,-x,-v+3/4,u+1/4,-t+1/4)

Standard settings

Superspace group: 225.3.212.6 Fm-3m(0,b,b)00q(b,0,b)s0q(b,b,0)00q [Y:none]
Bravais class: 3.212 Fm-3m(0,b,b)(b,0,b)(b,b,0) [JJdW:3.214]
Transformation to supercentered setting: A1=a1, A2=a2, A3=a3, A4=a5+a6, A5=a4+a6, A6=a4+a5

BASIC SPACE GROUP SETTING
Modulation vectors: q1'=(0,b,b), q2'=(b,0,b), q3'=(b,b,0)
Centering: (0,0,0,0,0,0); (0,1/2,1/2,0,0,0); (1/2,0,1/2,0,0,0); (1/2,1/2,0,0,0,0)
Non-lattice generators: (x,y,-z,-u+v+1,-t+v+1/2,v+1/2); (-z,-x,-y,-v,-t,-u); (y,x,z,u+1/2,t+1/2,v+1/2)
Non-lattice operators: (x,y,z,t,u,v); (x,-y,-z,-t+1/2,-t+v,-t+u+1/2); (-x,y,-z,-u+v+1/2,-u+1/2,t-u); (-x,-y,z,u-v,t-v+1/2,-v+1/2); (y,z,x,u,v,t); (y,-z,-x,-u+1/2,t-u,-u+v+1/2); (-y,z,-x,t-v+1/2,-v+1/2,u-v); (-y,-z,x,-t+v,-t+u+1/2,-t+1/2); (z,x,y,v,t,u); (z,-x,-y,-v+1/2,u-v,t-v+1/2); (-z,x,-y,-t+u+1/2,-t+1/2,-t+v); (-z,-x,y,t-u,-u+v+1/2,-u+1/2); (-y,-x,-z,-u+1/2,-t+1/2,-v+1/2); (-y,x,z,u,u-v,-t+u+1/2); (y,-x,z,t-v+1/2,t,t-u); (y,x,-z,-t+v,-u+v+1/2,v); (-x,-z,-y,-t+1/2,-v+1/2,-u+1/2); (-x,z,y,t,t-u,t-v+1/2); (x,-z,y,-u+v+1/2,v,-t+v); (x,z,-y,u-v,-t+u+1/2,u); (-z,-y,-x,-v+1/2,-u+1/2,-t+1/2); (-z,y,x,v,-t+v,-u+v+1/2); (z,-y,x,-t+u+1/2,u,u-v); (z,y,-x,t-u,t-v+1/2,t); (-x,-y,-z,-t,-u,-v); (-x,y,z,t+1/2,t-v,t-u+1/2); (x,-y,z,u-v+1/2,u+1/2,-t+u); (x,y,-z,-u+v,-t+v+1/2,v+1/2); (-y,-z,-x,-u,-v,-t); (-y,z,x,u+1/2,-t+u,u-v+1/2); (y,-z,x,-t+v+1/2,v+1/2,-u+v); (y,z,-x,t-v,t-u+1/2,t+1/2); (-z,-x,-y,-v,-t,-u); (-z,x,y,v+1/2,-u+v,-t+v+1/2); (z,-x,y,t-u+1/2,t+1/2,t-v); (z,x,-y,-t+u,u-v+1/2,u+1/2); (y,x,z,u+1/2,t+1/2,v+1/2); (y,-x,-z,-u,-u+v,t-u+1/2); (-y,x,-z,-t+v+1/2,-t,-t+u); (-y,-x,z,t-v,u-v+1/2,-v); (x,z,y,t+1/2,v+1/2,u+1/2); (x,-z,-y,-t,-t+u,-t+v+1/2); (-x,z,-y,u-v+1/2,-v,t-v); (-x,-z,y,-u+v,t-u+1/2,-u); (z,y,x,v+1/2,u+1/2,t+1/2); (z,-y,-x,-v,t-v,u-v+1/2); (-z,y,-x,t-u+1/2,-u,-u+v); (-z,-y,x,-t+u,-t+v+1/2,-t)

SUPERCENTERED SETTING
Modulation vectors: Q1'=(B,0,0), Q2'=(0,B,0), Q3'=(0,0,B), where B=b
Centering: (0,0,0,0,0,0); (0,1/2,1/2,0,0,0); (1/2,0,1/2,0,0,0); (1/2,1/2,0,0,0,0); (0,0,0,1/2,1/2,1/2); (0,1/2,1/2,1/2,1/2,1/2); (1/2,0,1/2,1/2,1/2,1/2); (1/2,1/2,0,1/2,1/2,1/2)
Non-lattice generators: (X,Y,-Z,T,U+1/2,-V+1/2); (-Z,-X,-Y,-V,-T,-U); (Y,X,Z,U+1/4,T+1/4,V+1/4)
Non-lattice operators: (X,Y,Z,T,U,V); (X,-Y,-Z,T,-U+1/2,-V); (-X,Y,-Z,-T,U,-V+1/2); (-X,-Y,Z,-T+1/2,-U,V); (Y,Z,X,U,V,T); (Y,-Z,-X,U,-V+1/2,-T); (-Y,Z,-X,-U,V,-T+1/2); (-Y,-Z,X,-U+1/2,-V,T); (Z,X,Y,V,T,U); (Z,-X,-Y,V,-T+1/2,-U); (-Z,X,-Y,-V,T,-U+1/2); (-Z,-X,Y,-V+1/2,-T,U); (-Y,-X,-Z,-U+1/4,-T+1/4,-V+1/4); (-Y,X,Z,-U+1/4,T+1/4,V+3/4); (Y,-X,Z,U+1/4,-T+3/4,V+3/4); (Y,X,-Z,U+1/4,T+3/4,-V+1/4); (-X,-Z,-Y,-T+1/4,-V+1/4,-U+1/4); (-X,Z,Y,-T+1/4,V+1/4,U+3/4); (X,-Z,Y,T+1/4,-V+3/4,U+3/4); (X,Z,-Y,T+1/4,V+3/4,-U+1/4); (-Z,-Y,-X,-V+1/4,-U+1/4,-T+1/4); (-Z,Y,X,-V+1/4,U+1/4,T+3/4); (Z,-Y,X,V+1/4,-U+3/4,T+3/4); (Z,Y,-X,V+1/4,U+3/4,-T+1/4); (-X,-Y,-Z,-T,-U,-V); (-X,Y,Z,-T,U+1/2,V); (X,-Y,Z,T,-U,V+1/2); (X,Y,-Z,T+1/2,U,-V); (-Y,-Z,-X,-U,-V,-T); (-Y,Z,X,-U,V+1/2,T); (Y,-Z,X,U,-V,T+1/2); (Y,Z,-X,U+1/2,V,-T); (-Z,-X,-Y,-V,-T,-U); (-Z,X,Y,-V,T+1/2,U); (Z,-X,Y,V,-T,U+1/2); (Z,X,-Y,V+1/2,T,-U); (Y,X,Z,U+1/4,T+1/4,V+1/4); (Y,-X,-Z,U+1/4,-T+1/4,-V+3/4); (-Y,X,-Z,-U+1/4,T+3/4,-V+3/4); (-Y,-X,Z,-U+1/4,-T+3/4,V+1/4); (X,Z,Y,T+1/4,V+1/4,U+1/4); (X,-Z,-Y,T+1/4,-V+1/4,-U+3/4); (-X,Z,-Y,-T+1/4,V+3/4,-U+3/4); (-X,-Z,Y,-T+1/4,-V+3/4,U+1/4); (Z,Y,X,V+1/4,U+1/4,T+1/4); (Z,-Y,-X,V+1/4,-U+1/4,-T+3/4); (-Z,Y,-X,-V+1/4,U+3/4,-T+3/4); (-Z,-Y,X,-V+1/4,-U+3/4,T+1/4)
Reflection conditions: HKLMNP:H+K=2n; HKLMNP:H+L=2n; HKLMNP:M+N+P=2n; HHLMMP:2M+P=4n; H-HLM-MP:2M+P=4n; HKHMNM:2M+N=4n; HK-HMN-M:2M-N=4n; HKKMNN:M+2N=4n; HK-KMN-N:M+2N=4n; HK0MN0:M=2n; H0LM0P:P=2n; 0KL0NP:N=2n

Affine transformation to standard basic space group setting

$S * g(\text{input}) * S^{-1} = g(\text{standard})$,
where g is an augmented matrix for an operation in the superspace group.
Also, $S * r(\text{input}) = r(\text{standard})$,
where r is an augmented position vector, (x,y,z,t,u,v,1).

$$S = \begin{pmatrix} 0 & 0 & 1 & 0 & 0 & 0 & 0 \\ 1 & 0 & 0 & 0 & 0 & 0 & 0 \\ 0 & 1 & 0 & 0 & 0 & 0 & 0 \\ 0 & 0 & 0 & 1 & 1 & 0 & 0 \\ 0 & 0 & 0 & 0 & 1 & 1 & 0 \\ 0 & 0 & 0 & 1 & 0 & 1 & 0 \\ 0 & 0 & 0 & 0 & 0 & 0 & 1 \end{pmatrix} \quad S^{-1} = \begin{pmatrix} 0 & 1 & 0 & 0 & 0 & 0 & 0 \\ 0 & 0 & 1 & 0 & 0 & 0 & 0 \\ 1 & 0 & 0 & 0 & 0 & 0 & 0 \\ 0 & 0 & 0 & 1/2 & -1/2 & 1/2 & 0 \\ 0 & 0 & 0 & 1/2 & 1/2 & -1/2 & 0 \\ 0 & 0 & 0 & -1/2 & 1/2 & 1/2 & 0 \\ 0 & 0 & 0 & 0 & 0 & 0 & 1 \end{pmatrix}$$

a1' = a3	a1*' = a3*	q1' = q1 + q2 = (0,b,b)
a2' = a1	a2*' = a1*	q2' = q2 + q3 = (b,0,b)
a3' = a2	a3*' = a2*	q3' = q1 + q3 = (b,b,0)

a1 = a2'	a1* = a2*'	q1 = 1/2 q1' - 1/2 q2' + 1/2 q3' = (b,0,0)
a2 = a3'	a2* = a3*'	q2 = 1/2 q1' + 1/2 q2' - 1/2 q3' = (0,b,0)
a3 = a1'	a3* = a1*'	q3 = -1/2 q1' + 1/2 q2' + 1/2 q3' = (0,0,b)
