# Supplementary material for: Equivalence of superspace groups
Source: Acta Crystallogr A. 2012 Nov 14;69(Pt 1):75–90. doi: 10.1107/S0108767312041657 (PMC3553647; doi:10.1107/S0108767312041657)
Supplement: Supplementary file 1 [file a-69-00075-sup1.zip › ssg3d_fm3m_a00_fe1xo.pdf]

225.3.209.1 Fm-3m(a,0,0)000(0,a,0)000(0,0,a)000

Superspace group: 225.3.209.1 Fm-3m(a,0,0)000(0,a,0)000(0,0,a)000 [Y:3.11194, 3.11196]
Bravais class: 3.209 Fm-3m(a,0,0)(0,a,0)(0,0,a) [JJdW:3.211]
Transformation to supercentered setting: none
Modulation vectors: q1=(a,0,0), q2=(0,a,0), q3=(0,0,a)
Centering: (0,0,0,0,0,0); (0,1/2,1/2,0,0,0); (1/2,0,1/2,0,0,0); (1/2,1/2,0,0,0,0)
Non-lattice generators: (x,y,-z,t,u,-v); (-z,-x,-y,-v,-t,-u); (y,x,z,u,t,v)
Non-lattice operators: (x,y,z,t,u,v); (x,-y,-z,t,-u,-v); (-x,y,-z,-t,u,-v); (-x,-y,z,-t,-u,v); (y,z,x,u,v,t); (y,-z,-x,u,-v,-t); (-y,z,-x,-u,v,-t); (-y,-z,x,-u,-v,t); (z,x,y,v,t,u); (z,-x,-y,v,-t,-u); (-z,x,-y,-v,t,-u); (-z,-x,y,-v,-t,u); (-y,-x,-z,-u,-t,-v); (-y,x,z,-u,t,v); (y,-x,z,u,-t,v); (y,x,-z,u,t,-v); (-x,-z,-y,-t,-v,-u); (-x,z,y,-t,v,u); (x,-z,y,t,-v,u); (x,z,-y,t,v,-u); (-z,-y,-x,-v,-u,-t); (-z,y,x,-v,u,t); (z,-y,x,v,-u,t); (z,y,-x,v,u,-t); (-x,-y,-z,-t,-u,-v); (-x,y,z,-t,u,v); (x,-y,z,t,-u,v); (x,y,-z,t,u,-v); (-y,-z,-x,-u,-v,-t); (-y,z,x,-u,v,t); (y,-z,x,u,-v,t); (y,z,-x,u,v,-t); (-z,-x,-y,-v,-t,-u); (-z,x,y,-v,t,u); (z,-x,y,v,-t,u); (z,x,-y,v,t,-u); (y,x,z,u,t,v); (y,-x,-z,u,-t,-v); (-y,x,-z,-u,t,-v); (-y,-x,z,-u,-t,v); (x,z,y,t,v,u); (x,-z,-y,t,-v,-u); (-x,z,-y,-t,v,-u); (-x,-z,y,-t,-v,u); (z,y,x,v,u,t); (z,-y,-x,v,-u,-t); (-z,y,-x,-v,u,-t); (-z,-y,x,-v,-u,t)
Reflection conditions: hklmnp:h+k=2n; hklmnp:h+l=2n

This is the symmetry of Wustite $Fe_{1-x}O$. See A. Yamamoto, Acta Cryst B 38, 1451 (1982).

The F-center of the BSG is preserved as centring of the superspace lattice.

Four SSG exist with q-vectors forming a primitive lattice and different intrinsic translational components along the internal dimensions.

225.3.209.1 Fm-3m(a,0,0)000(0,a,0)000(0,0,a)000
225.3.209.2 Fm-3m(a,0,0)s00(0,a,0)s00(0,0,a)000
225.3.209.3 Fm-3m(a,0,0)000(0,a,0)000(0,0,a)00s
225.3.209.4 Fm-3m(a,0,0)s00(0,a,0)s00(0,0,a)00s

-----

findssg Fm-3m(a,0,0)000(0,a,0)000(0,0,a)000

Operators of the standard BSG setting have been inserted into findssg.

Input setting

Centering
(0,0,0,0,0,0); (0,1/2,1/2,0,0,0); (1/2,0,1/2,0,0,0); (1/2,1/2,0,0,0,0)
Operators
(x,y,-z,t,u,-v); (-z,-x,-y,-v,-t,-u); (y,x,z,u,t,v); (x,y,z,t,u,v); (-z,-x,y,-v,-t,u); (y,x,-z,u,t,-v); (z,-x,-y,v,-t,-u); (y,z,x,u,v,t); (-z,-y,-x,-v,-u,-t); (-y,z,x,-u,v,t); (z,-y,-x,v,-u,-t); (-x,-z,-y,-t,-v,-u); (-x,-z,y,-t,-v,u); (-x,z,-y,-t,v,-u); (z,y,x,v,u,t); (-y,-z,-x,-u,-v,-t); (z,-y,x,v,-u,t); (-y,z,-x,-u,v,-t); (z,-x,y,v,-t,u); (y,z,-x,u,v,-t); (-z,-y,x,-v,-u,t); (-x,z,y,-t,v,u); (z,y,-x,v,u,-t); (-y,-z,x,-u,-v,t); (y,-z,x,u,-v,t); (-x,-y,-z,-t,-u,-v); (x,z,y,t,v,u); (-x,y,-z,-t,u,-v); (x,-z,y,t,-v,u); (-y,x,z,-u,t,v); (x,-y,-z,t,-u,-v); (-y,x,-z,-u,t,-v); (x,-z,-y,t,-v,-u); (-x,y,z,-t,u,v); (-z,y,x,-v,u,t); (-y,-x,-z,-u,-t,-v); (z,x,y,v,t,u); (y,-x,-z,u,-t,-v); (-z,x,y,-v,t,u); (x,-y,z,t,-u,v); (-z,x,-y,-v,t,-u); (y,-x,z,u,-t,v); (y,-z,-x,u,-v,-t); (-x,-y,z,-t,-u,v); (x,z,-y,t,v,-u); (z,x,-y,v,t,-u); (-y,-x,z,-u,-t,v); (-z,y,-x,-v,u,-t)

Standard settings

Superspace group: 225.3.209.1 Fm-3m(a,0,0)000(0,a,0)000(0,0,a)000 [Y:3.11194, 3.11196]
Bravais class: 3.209 Fm-3m(a,0,0)(0,a,0)(0,0,a) [JJdW:3.211]
Transformation to supercentered setting: none
Modulation vectors: q1'=(a,0,0), q2'=(0,a,0), q3'=(0,0,a)
Centering: (0,0,0,0,0,0); (0,1/2,1/2,0,0,0); (1/2,0,1/2,0,0,0); (1/2,1/2,0,0,0,0)
Non-lattice generators: (x,y,-z,t,u,-v); (-z,-x,-y,-v,-t,-u); (y,x,z,u,t,v)
Non-lattice operators: (x,y,z,t,u,v); (x,-y,-z,t,-u,-v); (-x,y,-z,-t,u,-v); (-x,-y,z,-t,-u,v); (y,z,x,u,v,t); (y,-z,-x,u,-v,-t); (-y,z,-x,-u,v,-t); (-y,-z,x,-u,-v,t); (z,x,y,v,t,u); (z,-x,-y,v,-t,-u); (-z,x,-y,-v,t,-u); (-z,-x,y,-v,-t,u); (-y,-x,-z,-u,-t,-v); (-y,x,z,-u,t,v); (y,-x,z,u,-t,v); (y,x,-z,u,t,-v); (-x,-z,-y,-t,-v,-u); (-x,z,y,-t,v,u); (x,-z,y,t,-v,u); (x,z,-y,t,v,-u); (-z,-y,-x,-v,-u,-t); (-z,y,x,-v,u,t); (z,-y,x,v,-u,t); (z,y,-x,v,u,-t); (-x,-y,-z,-t,-u,-v); (-x,y,z,-t,u,v); (x,-y,z,t,-u,v); (x,y,-z,t,u,-v); (-y,-z,-x,-u,-v,-t); (-y,z,x,-u,v,t); (y,-z,x,u,-v,t); (y,z,-x,u,v,-t); (-z,-x,-y,-v,-t,-u); (-z,x,y,-v,t,u); (z,-x,y,v,-t,u); (z,x,-y,v,t,-u); (y,x,z,u,t,v); (y,-x,-z,u,-t,-v); (-y,x,-z,-u,t,-v); (-y,-x,z,-u,-t,v); (x,z,y,t,v,u); (x,-z,-y,t,-v,-u); (-x,z,-y,-t,v,-u); (-x,-z,y,-t,-v,u); (z,y,x,v,u,t); (z,-y,-x,v,-u,-t); (-z,y,-x,-v,u,-t); (-z,-y,x,-v,-u,t)
Reflection conditions: hklmnp:h+k=2n; hklmnp:h+l=2n

Affine transformation to standard basic space group setting

$S * g(input) * S^{-1} = g(standard)$,
where g is an augmented matrix for an operation in the superspace group.
Also, S * r(input) = r(standard),
where r is an augmented position vector, (x,y,z,t,u,v,1).

$$S = \begin{pmatrix} 1&0&0&0&0&0&0 \\ 0&1&0&0&0&0&0 \\ 0&0&1&0&0&0&0 \\ 0&0&0&1&0&0&0 \\ 0&0&0&0&1&0&0 \\ 0&0&0&0&0&1&0 \\ 0&0&0&0&0&0&1 \end{pmatrix} \quad S^{-1} = \begin{pmatrix} 1&0&0&0&0&0&0 \\ 0&1&0&0&0&0&0 \\ 0&0&1&0&0&0&0 \\ 0&0&0&1&0&0&0 \\ 0&0&0&0&1&0&0 \\ 0&0&0&0&0&1&0 \\ 0&0&0&0&0&0&1 \end{pmatrix}$$

a1' = a1
a2' = a2
a3' = a3

a1 = a1'
a2 = a2'
a3 = a3'

a1*' = a1*
a2*' = a2*
a3*' = a3*

a1* = a1*'
a2* = a2*'
a3* = a3*'

q1' = q1 = (a,0,0)
q2' = q2 = (0,a,0)
q3' = q3 = (0,0,a)

q1 = q1' = (a,0,0)
q2 = q2' = (0,a,0)
q3 = q3' = (0,0,a)

Superspace groups as given by Yamamoto for 225.3.209.1

11194   Fm3m(p00,0p0,00p)mtm

(000000;1/21/20000;1/201/2000;01/21/2000)

x,y,z,t,u,v; -x,-y,z,-t,-u,v; -x,y,-z,-t,u,-v; x,-y,-z,t,-u,-v; z,x,y,v,t,u; z,-x,-y,v,-t,-u; -z,-x,y,-v,-t,u; -z,x,-y,-v,t,-u; y,z,x,u,v,t; -y,z,-x,-u,v,-t; y,-z,-x,u,-v,-t; -y,-z,x,-u,-v,t; y,x,-z,u,t,-v; -y,-x,-z,-u,-t,-v; y,-x,z,u,-t,v; -y,x,z,-u,t,v; x,z,-y,t,v,-u; -x,z,y,-t,v,u; -x,-z,-y,-t,-v,-u; x,-z,y,t,-v,u; z,y,-x,v,u,-t; z,-y,x,v,-u,t; -z,y,x,-v,u,t; -z,-y,-x,-v,-u,-t; -x,-y,-z,-t,-u,-v; x,y,-z,t,u,-v; x,-y,z,t,-u,v; -x,y,z,-t,u,v; -z,-x,-y,-v,-t,-u; -z,x,y,-v,t,u; z,x,-y,v,t,-u; z,-x,y,v,-t,u; -y,-z,-x,-u,-v,-t; y,-z,x,u,-v,t; -y,z,x,-u,v,t; y,z,-x,u,v,-t; -y,-x,z,-u,-t,v; y,x,z,u,t,v; -y,x,-z,-u,t,-v; y,-x,-z,u,-t,-v; -x,-z,y,-t,-v,u; x,-z,-y,t,-v,-u; x,z,y,t,v,u; -x,z,-y,-t,v,-u; -z,-y,x,-v,-u,t; -z,y,-x,-v,u,-t; z,-y,-x,v,-u,-t; z,y,x,v,u,t;

hklmni:h+k=2n hklmni:h+l=2n hklmni:k+l=2n

11196   Fm3m(p00,0p0,00p)mtm

(000000;1/21/20000;1/201/2000;01/21/2000)

x,y,z,t,u,v; -x,-y,z,-t,-u,v; -x,y,-z,-t,u,-v; x,-y,-z,t,-u,-v; z,x,y,v,t,u; z,-x,-y,v,-t,-u; -z,-x,y,-v,-t,u; -z,x,-y,-v,t,-u; y,z,x,u,v,t; -y,z,-x,-u,v,-t; y,-z,-x,u,-v,-t; -y,-z,x,-u,-v,t; y,x,1/2-z,u,t,-v; -y,-x,1/2-z,-u,-t,-v; y,-x,1/2+z,u,-t,v; -y,x,1/2+z,-u,t,v; x,z,1/2-y,t,v,-u; -x,z,1/2+y,-t,v,u; -x,-z,1/2-y,-t,-v,-u; x,-z,1/2+y,t,-v,u; z,y,1/2-x,v,u,-t; z,-y,1/2+x,v,-u,t; -z,y,1/2+x,-v,u,t; -z,-y,1/2-x,-v,-u,-t; -x,-y,1/2-z,-t,-u,-v; x,y,1/2-z,t,u,-v; x,-y,1/2+z,t,-u,v; -x,y,1/2+z,-t,u,v; -z,-x,1/2-y,-v,-t,-u; -z,x,1/2+y,-v,t,u; z,x,1/2-y,v,t,-u; z,-x,1/2+y,v,-t,u; -y,-z,1/2-x,-u,-v,-t; y,-z,1/2+x,u,-v,t; -y,z,1/2+x,-u,v,t; y,z,1/2-x,u,v,-t; -y,-x,z,-u,-t,v; y,x,z,u,t,v; -y,x,-z,-u,t,-v; y,-x,-z,u,-t,-v; -x,-z,y,-t,-v,u; x,-z,-y,t,-v,-u; x,z,y,t,v,u; -x,z,-y,-t,v,-u; -z,-y,x,-v,-u,t; -z,y,-x,-v,u,-t; z,-y,-x,v,-u,-t; z,y,x,v,u,t;

hklmni:h+k=2n hklmni:h+l=2n hklmni:k+l=2n 00l00i:l=2n 00l00i:l=2n 0ll0ii:l+l=4n 0-ll0-ii:3l+l=4n l0li0i:l+l=4n h0-hm0-m:3h-h=4n h0lm0i:l=2n 0kl0ni:l=2n

-----

findssg Y:11194 Fm3m(p00,0p0,00p)mtm

Operators of Yamamoto: 11194 Fm3m(p00,0p0,00p)mtm are entered into findssg.

Input setting

Centering
(0,0,0,0,0,0); (1/2,1/2,0,0,0,0); (1/2,0,1/2,0,0,0); (0,1/2,1/2,0,0,0)
Operators
(-x,-y,z,-t,-u,v); (-x,y,-z,-t,u,-v); (x,-y,-z,t,-u,-v); (z,x,y,v,t,u); (z,-x,-y,v,-t,-u); (-z,-x,y,-v,-t,u); (-z,x,-y,-v,t,-u); (y,z,x,u,v,t); (-y,z,-x,-u,v,-t); (y,-z,-x,u,-v,-t); (-y,-z,x,-u,-v,t); (y,x,-z,u,t,-v); (-y,-x,-z,-u,-t,-v); (y,-x,z,u,-t,v); (-y,x,z,-u,t,v); (x,z,-y,t,v,-u); (-x,z,y,-t,v,u); (-x,-z,-y,-t,-v,-u); (x,-z,y,t,-v,u); (z,y,-x,v,u,-t); (z,-y,x,v,-u,t); (-z,y,x,-v,u,t); (-z,-y,-x,-v,-u,-t); (-x,-y,-z,-t,-u,-v); (x,y,-z,t,u,-v); (x,-y,z,t,-u,v); (-x,y,z,-t,u,v); (-z,-x,-y,-v,-t,-u); (-z,x,y,-v,t,u); (z,x,-y,v,t,-u); (z,-x,y,v,-t,u); (x,y,z,t,u,v); (-y,-z,-x,-u,-v,-t); (y,-z,x,u,-v,t); (-y,z,x,-u,v,t); (y,z,-x,u,v,-t); (-y,-x,z,-u,-t,v); (y,x,z,u,t,v); (-y,x,-z,-u,t,-v); (y,-x,-z,u,-t,-v); (-x,-z,y,-t,-v,u); (x,-z,-y,t,-v,-u); (x,z,y,t,v,u); (-x,z,-y,-t,v,-u); (-z,-y,x,-v,-u,t); (-z,y,-x,-v,u,-t); (z,-y,-x,v,-u,-t); (z,y,x,v,u,t)

Standard settings

Superspace group: 225.3.209.1 Fm-3m(a,0,0)000(0,a,0)000(0,0,a)000 [Y:3.11194, 3.11196]
Bravais class: 3.209 Fm-3m(a,0,0)(0,a,0)(0,0,a) [JJdW:3.211]
Transformation to supercentered setting: none
Modulation vectors: q1'=(a,0,0), q2'=(0,a,0), q3'=(0,0,a)
Centering: (0,0,0,0,0,0); (0,1/2,1/2,0,0,0); (1/2,0,1/2,0,0,0); (1/2,1/2,0,0,0,0)
Non-lattice generators: (x,y,-z,t,u,-v); (-z,-x,-y,-v,-t,-u); (y,x,z,u,t,v)
Non-lattice operators: (x,y,z,t,u,v); (x,-y,-z,t,-u,-v); (-x,y,-z,-t,u,-v); (-x,-y,z,-t,-u,v); (y,z,x,u,v,t); (y,-z,-x,u,-v,-t); (-y,z,-x,-u,v,-t); (-y,-z,x,-u,-v,t); (z,x,y,v,t,u); (z,-x,-y,v,-t,-u); (-z,x,-y,-v,t,-u); (-z,-x,y,-v,-t,u); (-y,-x,-z,-u,-t,-v); (-y,x,z,-u,t,v); (y,-x,z,u,-t,v); (y,x,-z,u,t,-v); (-x,-z,-y,-t,-v,-u); (-x,z,y,-t,v,u); (x,-z,y,t,-v,u); (x,z,-y,t,v,-u); (-z,-y,-x,-v,-u,-t); (-z,y,x,-v,u,t); (z,-y,x,v,-u,t); (z,y,-x,v,u,-t); (-x,-y,-z,-t,-u,-v); (-x,y,z,-t,u,v); (x,-y,z,t,-u,v); (x,y,-z,t,u,-v); (-y,-z,-x,-u,-v,-t); (-y,z,x,-u,v,t); (y,-z,x,u,-v,t); (y,z,-x,u,v,-t); (-z,-x,-y,-v,-t,-u); (-z,x,y,-v,t,u); (z,-x,y,v,-t,u); (z,x,-y,v,t,-u); (y,x,z,u,t,v); (y,-x,-z,u,-t,-v); (-y,x,-z,-u,t,-v); (-y,-x,z,-u,-t,v); (x,z,y,t,v,u); (x,-z,-y,t,-v,-u); (-x,z,-y,-t,v,-u); (-x,-z,y,-t,-v,u); (z,y,x,v,u,t); (z,-y,-x,v,-u,-t); (-z,y,-x,-v,u,-t); (-z,-y,x,-v,-u,t)
Reflection conditions: hklmnp:h+k=2n; hklmnp:h+l=2n

Affine transformation to standard basic space group setting

S * g(input) * S^{-1} = g(standard),
where g is an augmented matrix for an operation in the superspace group.
Also, S * r(input) = r(standard),
where r is an augmented position vector, (x,y,z,t,u,v,1).

$$S = \begin{matrix} 1 & 0 & 0 & 0 & 0 & 0 & 0 \\ 0 & 1 & 0 & 0 & 0 & 0 & 0 \\ 0 & 0 & 1 & 0 & 0 & 0 & 0 \\ 0 & 0 & 0 & 1 & 0 & 0 & 0 \\ 0 & 0 & 0 & 0 & 1 & 0 & 0 \\ 0 & 0 & 0 & 0 & 0 & 1 & 0 \\ 0 & 0 & 0 & 0 & 0 & 0 & 1 \end{matrix} \qquad S^{-1} = \begin{matrix} 1 & 0 & 0 & 0 & 0 & 0 & 0 \\ 0 & 1 & 0 & 0 & 0 & 0 & 0 \\ 0 & 0 & 1 & 0 & 0 & 0 & 0 \\ 0 & 0 & 0 & 1 & 0 & 0 & 0 \\ 0 & 0 & 0 & 0 & 1 & 0 & 0 \\ 0 & 0 & 0 & 0 & 0 & 1 & 0 \\ 0 & 0 & 0 & 0 & 0 & 0 & 1 \end{matrix}$$

a1' = a1
a2' = a2
a3' = a3

a1 = a1'
a2 = a2'
a3 = a3'

a1*' = a1*
a2*' = a2*
a3*' = a3*

a1* = a1*'
a2* = a2*'
a3* = a3*'

q1' = q1 = (a,0,0)
q2' = q2 = (0,a,0)
q3' = q3 = (0,0,a)

q1 = q1' = (a,0,0)
q2 = q2' = (0,a,0)
q3 = q3' = (0,0,a)

findssg Y: 11196 Fm3m(p00,0p0,00p)mtm

Operators of Yamamoto: 11194 Fm3m(p00,0p0,00p)mtm are entered into findssg.
Y: 11196 is the same SSG as Y: 11194. They are two settings that differ from each other by an origin shift.

Input setting

Centering
(0,0,0,0,0,0); (1/2,1/2,0,0,0,0); (1/2,0,1/2,0,0,0); (0,1/2,1/2,0,0,0)
Operators
(-x,-y,z,-t,-u,v); (-x,y,-z,-t,u,-v); (x,-y,-z,t,-u,-v); (z,x,y,v,t,u); (z,-x,-y,v,-t,-u); (-z,-x,y,-v,-t,u); (-z,x,-y,-v,t,-u); (y,z,x,u,v,t); (-y,z,-x,-u,v,-t); (y,-z,-x,u,-v,-t); (-y,-z,x,-u,-v,t); (y,x,-z+1/2,u,t,-v); (-y,-x,-z+1/2,-u,-t,-v); (y,-x,z+1/2,u,-t,v); (-y,x,z+1/2,-u,t,v); (x,z,-y+1/2,t,v,-u); (-x,z,y+1/2,-t,v,u); (-x,-z,-y+1/2,-t,-v,-u); (x,-z,y+1/2,t,-v,u); (z,y,-x+1/2,v,u,-t); (z,-y,x+1/2,v,-u,t); (-z,y,x+1/2,-v,u,t); (-z,-y,-x+1/2,-v,-u,-t); (-x,-y,-z+1/2,-t,-u,-v); (x,y,-z+1/2,t,u,-v); (x,-y,z+1/2,t,-u,v); (-x,y,z+1/2,-t,u,v); (-z,-x,-y+1/2,-v,-t,-u); (-z,x,y+1/2,-v,t,u); (z,x,-y+1/2,v,t,-u); (z,-x,y+1/2,v,-t,u); (-y,-z,-x+1/2,-u,-v,-t); (y,-z,x+1/2,u,-v,t); (-y,z,x+1/2,-u,v,t); (y,z,-x+1/2,u,v,-t); (-y,-x,z,-u,-t,v); (y,x,z,u,t,v); (x,y,z,t,u,v); (y,-x,-z,u,-t,-v); (-y,x,-z,-u,t,-v); (z,-y,-x,v,-u,-t); (z,y,x,v,u,t); (-z,y,-x,-v,u,-t); (-z,-y,x,-v,-u,t); (-x,-z,y,-t,-v,u); (x,-z,-y,t,-v,-u); (-x,z,-y,-t,v,-u); (x,z,y,t,v,u)

Standard settings

Superspace group: 225.3.209.1 Fm-3m(a,0,0)000(0,a,0)000(0,0,a)000 [Y:3.11194, 3.11196]
Bravais class: 3.209 Fm-3m(a,0,0)(0,a,0)(0,0,a) [JJdW:3.211]
Transformation to supercentered setting: none
Modulation vectors: q1'=(a,0,0), q2'=(0,a,0), q3'=(0,0,a)
Centering: (0,0,0,0,0,0); (0,1/2,1/2,0,0,0); (1/2,0,1/2,0,0,0); (1/2,1/2,0,0,0,0)
Non-lattice generators: (x,y,-z,t,u,-v); (-z,-x,-y,-v,-t,-u); (y,x,z,u,t,v)
Non-lattice operators: (x,y,z,t,u,v); (x,-y,-z,t,-u,-v); (-x,y,-z,-t,u,-v); (-x,-y,z,-t,-u,v); (y,z,x,u,v,t); (y,-z,-x,u,-v,-t); (-y,z,-x,-u,v,-t); (-y,-z,x,-u,-v,t); (z,x,y,v,t,u); (z,-x,-y,v,-t,-u); (-z,x,-y,-v,t,-u); (-z,-x,y,-v,-t,u); (-y,-x,-z,-u,-t,-v); (-y,x,z,-u,t,v); (y,-x,z,u,-t,v); (y,x,-z,u,t,-v); (-x,-z,-y,-t,-v,-u); (-x,z,y,-t,v,u); (x,-z,y,t,-v,u); (x,z,-y,t,v,-u); (-z,-y,-x,-v,-u,-t); (-z,y,x,-v,u,t); (z,-y,x,v,-u,t); (z,y,-x,v,u,-t); (-x,-y,-z,-t,-u,-v); (-x,y,z,-t,u,v); (x,-y,z,t,-u,v); (x,y,-z,t,u,-v); (-y,-z,-x,-u,-v,-t); (-y,z,x,-u,v,t); (y,-z,x,u,-v,t); (y,z,-x,u,v,-t); (-z,-x,-y,-v,-t,-u); (-z,x,y,-v,t,u); (z,-x,y,v,-t,u); (z,x,-y,v,t,-u); (y,x,z,u,t,v); (y,-x,-z,u,-t,-v); (-y,x,-z,-u,t,-v); (-y,-x,z,-u,-t,v); (x,z,y,t,v,u); (x,-z,-y,t,-v,-u); (-x,z,-y,-t,v,-u); (-x,-z,y,-t,-v,u); (z,y,x,v,u,t); (z,-y,-x,v,-u,-t); (-z,y,-x,-v,u,-t); (-z,-y,x,-v,-u,t)
Reflection conditions: hklmnp:h+k=2n; hklmnp:h+l=2n

Affine transformation to standard basic space group setting

S * g(input) * S^{-1} = g(standard),
where g is an augmented matrix for an operation in the superspace group.
Also, S * r(input) = r(standard),
where r is an augmented position vector, (x,y,z,t,u,v,1).

$$S = \begin{pmatrix} 1 & 0 & 0 & 0 & 0 & 0 & 3/4 \\ 0 & 1 & 0 & 0 & 0 & 0 & 3/4 \\ 0 & 0 & 1 & 0 & 0 & 0 & 3/4 \\ 0 & 0 & 0 & 1 & 0 & 0 & 0 \\ 0 & 0 & 0 & 0 & 1 & 0 & 0 \\ 0 & 0 & 0 & 0 & 0 & 1 & 0 \\ 0 & 0 & 0 & 0 & 0 & 0 & 1 \end{pmatrix} \quad S^{-1} = \begin{pmatrix} 1 & 0 & 0 & 0 & 0 & 0 & -3/4 \\ 0 & 1 & 0 & 0 & 0 & 0 & -3/4 \\ 0 & 0 & 1 & 0 & 0 & 0 & -3/4 \\ 0 & 0 & 0 & 1 & 0 & 0 & 0 \\ 0 & 0 & 0 & 0 & 1 & 0 & 0 \\ 0 & 0 & 0 & 0 & 0 & 1 & 0 \\ 0 & 0 & 0 & 0 & 0 & 0 & 1 \end{pmatrix}$$

a1' = a1
a2' = a2
a3' = a3

a1 = a1'
a2 = a2'
a3 = a3'

a1*' = a1*
a2*' = a2*
a3*' = a3*

a1* = a1*'
a2* = a2*'
a3* = a3*'

q1' = q1 = (a,0,0)
q2' = q2 = (0,a,0)
q3' = q3 = (0,0,a)

q1 = q1' = (a,0,0)
q2 = q2' = (0,a,0)
q3 = q3' = (0,0,a)
