# Supplementary material for: Equivalence of superspace groups
Source: Acta Crystallogr A. 2012 Nov 14;69(Pt 1):75–90. doi: 10.1107/S0108767312041657 (PMC3553647; doi:10.1107/S0108767312041657)
Supplement: Supplementary file 1 [file a-69-00075-sup1.zip › ssg3d_fm3m_aaa_bi85cr15o173.pdf]

225.3.215.7 Fm-3m(a,a,a)000(a,-a,-a)000(-a,a,-a)000

Superspace group: 225.3.215.7 Fm-3m(a,a,a)000(a,-a,-a)000(-a,a,-a)000 [Y:none]
Bravais class: 3.215 Fm-3m(a,a,a)(a,-a,-a)(-a,a,-a) [JJdW:3.217]
Transformation to supercentered setting: A1=a1, A2=a2, A3=a3, A4=a4+a5-a6, A5=a4-a5+a6, A6=a4-a5-a6

BASIC SPACE GROUP SETTING
Modulation vectors: q1=(a,a,a), q2=(a,-a,-a), q3=(-a,a,-a)
Centering: (0,0,0,0,0,0); (0,1/2,1/2,0,0,0); (1/2,0,1/2,0,0,0); (1/2,1/2,0,0,0,0)
Non-lattice generators: (x,y,-z,t+u+v,-v,-u); (-z,-x,-y,-t,t+u+v,-u); (y,x,z,t,v,u)
Non-lattice operators: (x,y,z,t,u,v); (x,-y,-z,u,t,-t-u-v); (-x,y,-z,v,-t-u-v,t); (-x,-y,z,-t-u-v,v,u); (y,z,x,t,v,-t-u-v); (y,-z,-x,v,t,u); (-y,z,-x,-t-u-v,u,t); (-y,-z,x,u,-t-u-v,v); (z,x,y,t,-t-u-v,u); (z,-x,-y,-t-u-v,t,v); (-z,x,-y,u,v,t); (-z,-x,y,v,u,-t-u-v); (-y,-x,-z,-t,-v,-u); (-y,x,z,-v,-t,t+u+v); (y,-x,z,-u,t+u+v,-t); (y,x,-z,t+u+v,-u,-v); (-x,-z,-y,-t,-u,t+u+v); (-x,z,y,-u,-t,-v); (x,-z,y,t+u+v,-v,-t); (x,z,-y,-v,t+u+v,-u); (-z,-y,-x,-t,t+u+v,-v); (-z,y,x,t+u+v,-t,-u); (z,-y,x,-v,-u,-t); (z,y,-x,-u,-v,t+u+v); (-x,-y,-z,-t,-u,-v); (-x,y,z,-u,-t,t+u+v); (x,-y,z,-v,t+u+v,-t); (x,y,-z,t+u+v,-v,-u); (-y,-z,-x,-t,-v,t+u+v); (-y,z,x,-v,-t,-u); (y,-z,x,t+u+v,-u,-t); (y,z,-x,-u,t+u+v,-v); (-z,-x,-y,-t,t+u+v,-u); (-z,x,y,t+u+v,-t,-v); (z,-x,y,-u,-v,-t); (z,x,-y,-v,-u,t+u+v); (y,x,z,t,v,u); (y,-x,-z,v,t,-t-u-v); (-y,x,-z,u,-t-u-v,t); (-y,-x,z,-t-u-v,u,v); (x,z,y,t,u,-t-u-v); (x,-z,-y,u,t,v); (-x,z,-y,-t-u-v,v,t); (-x,-z,y,v,-t-u-v,u); (z,y,x,t,-t-u-v,v); (z,-y,-x,-t-u-v,t,u); (-z,y,-x,v,u,t); (-z,-y,x,u,v,-t-u-v)

SUPERCENTERED SETTING
Modulation vectors: Q1=(A,0,0), Q2=(0,A,0), Q3=(0,0,A), where A=a
Centering: (0,0,0,0,0,0); (0,1/2,1/2,0,0,0); (1/2,0,1/2,0,0,0); (1/2,1/2,0,0,0,0); (0,0,0,1/2,1/2,0); (0,1/2,1/2,1/2,1/2,0); (1/2,0,1/2,1/2,1/2,0); (1/2,1/2,0,1/2,1/2,0); (0,0,0,1/2,0,1/2); (0,1/2,1/2,1/2,0,1/2); (1/2,0,1/2,1/2,0,1/2); (1/2,1/2,0,1/2,0,1/2); (0,0,0,0,1/2,1/2); (0,1/2,1/2,0,1/2,1/2); (1/2,0,1/2,0,1/2,1/2); (1/2,1/2,0,0,1/2,1/2)
Non-lattice generators: (X,Y,-Z,T,U,-V); (-Z,-X,-Y,-V,-T,-U); (Y,X,Z,U,T,V)
Non-lattice operators: (X,Y,Z,T,U,V); (X,-Y,-Z,T,-U,-V); (-X,Y,-Z,-T,U,-V); (-X,-Y,Z,-T,-U,V); (Y,Z,X,U,V,T); (Y,-Z,-X,U,-V,-T); (-Y,Z,-X,-U,V,-T); (-Y,-Z,X,-U,-V,T); (Z,X,Y,V,T,U); (Z,-X,-Y,V,-T,-U); (-Z,X,-Y,-V,T,-U); (-Z,-X,Y,-V,-T,U); (-Y,-X,-Z,-U,-T,-V); (-Y,X,Z,-U,T,V); (Y,-X,Z,U,-T,V); (Y,X,-Z,U,T,-V); (-X,-Z,-Y,-T,-V,-U); (-X,Z,Y,-T,V,U); (X,-Z,Y,T,-V,U); (X,Z,-Y,T,V,-U); (-Z,-Y,-X,-V,-U,-T); (-Z,Y,X,-V,U,T); (Z,-Y,X,V,-U,T); (Z,Y,-X,V,U,-T); (-X,-Y,-Z,-T,-U,-V); (-X,Y,Z,-T,U,V); (X,-Y,Z,T,-U,V); (X,Y,-Z,T,U,-V); (-Y,-Z,-X,-U,-V,-T); (-Y,Z,X,-U,V,T); (Y,-Z,X,U,-V,T); (Y,Z,-X,U,V,-T); (-Z,-X,-Y,-V,-T,-U); (-Z,X,Y,-V,T,U); (Z,-X,Y,V,-T,U); (Z,X,-Y,V,T,-U); (Y,X,Z,U,T,V); (Y,-X,-Z,U,-T,-V); (-Y,X,-Z,-U,T,-V); (-Y,-X,Z,-U,-T,V); (X,Z,Y,T,V,U); (X,-Z,-Y,T,-V,-U); (-X,Z,-Y,-T,V,-U); (-X,-Z,Y,-T,-V,U); (Z,Y,X,V,U,T); (Z,-Y,-X,V,-U,-T); (-Z,Y,-X,-V,U,-T); (-Z,-Y,X,-V,-U,T)
Reflection conditions: HKLMNP:H+K=2n; HKLMNP:H+L=2n; HKLMNP:M+N=2n; HKLMNP:M+P=2n

-----

Four SSG exist with q-vectors forming a F-centered lattice and different intrinsic translational components along the internal dimensions:
225.3.215.7 Fm-3m(a,a,a)000(a,-a,-a)000(-a,a,-a)000
225.3.215.8 Fm-3m(a,a,a)q00(a,-a,-a)q00(-a,a,-a)000
225.3.215.9 Fm-3m(a,a,a)000(a,-a,-a)000(-a,a,-a)00s
225.3.215.10 Fm-3m(a,a,a)q00(a,-a,-a)q00(-a,a,-a)00s

This is the symmetry of Bi0.85Cr0.15O1.73.
S. Esmaeilzadeh, S. Lundgren, U. Haslenius, and J. Grins,
J. Solid State Chem. 156, 168-180 (2001).
and several publications by R.L. Withers. E.g.
R.L. Withers et al., Z. Kristallographie 219, 701-710 (2004).
R.L. Withers et al., Z. Kristallogr. 214, 296-304 (1999).

Published symbol is P:Fm-3m:Fm-3m for 225.3.215.7
-----

findssg Fm-3m(a,a,a)000(a,-a,-a)000(-a,a,-a)000

Generators of the standard BSG setting have been entered into findssg.

Input setting

Centering
(0,0,0,0,0,0); (1/2,1/2,0,0,0,0); (1/2,0,1/2,0,0,0); (0,1/2,1/2,0,0,0)
Operators
(x,y,-z,t+u+v,-v,-u); (-z,-x,-y,-t,t+u+v,-u); (y,x,z,t,v,u); (x,y,z,t,u,v); (-z,-x,y,v,u,-t-u-v); (y,x,-z,t+u+v,-u,-v); (z,-x,-y,-t-u-v,t,v); (y,z,x,t,v,-t-u-v); (-z,-y,-x,-t,t+u+v,-v); (-y,z,x,-v,-t,-u); (z,-y,-x,-t-u-v,t,u); (-x,-z,-y,-t,-u,t+u+v); (-x,-z,y,v,-t-u-v,u); (-x,z,-y,-t-u-v,v,t); (z,y,x,t,-t-u-v,v); (-y,-z,-x,-t,-v,t+u+v); (z,-y,x,-v,-u,-t); (-y,z,-x,-t-u-v,u,t); (z,-x,y,-u,-v,-t); (y,z,-x,-u,t+u+v,-v); (-z,-y,x,u,v,-t-u-v); (-x,z,y,-u,-t,-v); (z,y,-x,-u,-v,t+u+v); (-y,-z,x,u,-t-u-v,v); (y,-z,x,t+u+v,-u,-t); (-x,-y,-z,-t,-u,-v); (x,z,y,t,u,-t-u-v); (-x,y,-z,v,-t-u-v,t); (x,-z,y,t+u+v,-v,-t); (-y,x,z,-v,-t,t+u+v); (x,-y,-z,u,t,-t-u-v); (-y,x,-z,u,-t-u-v,t); (x,-z,-y,u,t,v); (-x,y,z,-u,-t,t+u+v); (-z,y,x,t+u+v,-t,-u); (-y,-x,-z,-t,-v,-u); (z,x,y,t,-t-u-v,u); (y,-x,-z,v,t,-t-u-v); (-z,x,y,t+u+v,-t,-v); (x,-y,z,-v,t+u+v,-t); (-z,x,-y,u,v,t); (y,-x,z,-u,t+u+v,-t); (y,-z,-x,v,t,u); (-x,-y,z,-t-u-v,v,u); (x,z,-y,-v,t+u+v,-u); (z,x,-y,-v,-u,t+u+v); (-y,-x,z,-t-u-v,u,v); (-z,y,-x,v,u,t)

Standard settings

Superspace group: 225.3.215.7 Fm-3m(a,a,a)000(a,-a,-a)000(-a,a,-a)000 [Y:none]
Bravais class: 3.215 Fm-3m(a,a,a)(a,-a,-a)(-a,a,-a) [JJdW:3.217]
Transformation to supercentered setting: A1=a1, A2=a2, A3=a3, A4=a4+a5-a6, A5=a4-a5+a6, A6=a4-a5-a6

BASIC SPACE GROUP SETTING
Modulation vectors: q1'=(a,a,a), q2'=(a,-a,-a), q3'=(-a,a,-a)
Centering: (0,0,0,0,0,0); (0,1/2,1/2,0,0,0); (1/2,0,1/2,0,0,0); (1/2,1/2,0,0,0,0)
Non-lattice generators: (x,y,-z,t+u+v,-v,-u); (-z,-x,-y,-t,t+u+v,-u); (y,x,z,t,v,u)
Non-lattice operators: (x,y,z,t,u,v); (x,-y,-z,u,t,-t-u-v); (-x,y,-z,v,-t-u-v,t); (-x,-y,z,-t-u-v,v,u); (y,z,x,t,v,-t-u-v); (y,-z,-x,v,t,u); (-y,z,-x,-t-u-v,u,t); (-y,-z,x,u,-t-u-v,v); (z,x,y,t,-t-u-v,u); (z,-x,-y,-t-u-v,t,v); (-z,x,-y,u,v,t); (-z,-x,y,v,u,-t-u-v); (-y,-x,-z,-t,-v,-u); (-y,x,z,-v,-t,t+u+v); (y,-x,z,-u,t+u+v,-t); (y,x,-z,t+u+v,-u,-v); (-x,-z,-y,-t,-u,t+u+v); (-x,z,y,-u,-t,-v); (x,-z,y,t+u+v,-v,-t); (x,z,-y,-v,t+u+v,-u); (-z,-y,-x,-t,t+u+v,-v); (-z,y,x,t+u+v,-t,-u); (z,-y,x,-v,-u,-t); (z,y,-x,-u,-v,t+u+v); (-x,-y,-z,-t,-u,-v); (-x,y,z,-u,-t,t+u+v); (x,-y,z,-v,t+u+v,-t); (x,y,-z,t+u+v,-v,-u); (-y,-z,-x,-t,-v,t+u+v); (-y,z,x,-v,-t,-u); (y,-z,x,t+u+v,-u,-t); (y,z,-x,-u,t+u+v,-v); (-z,-x,-y,-t,t+u+v,-u); (-z,x,y,t+u+v,-t,-v); (z,-x,y,-u,-v,-t); (z,x,-y,-v,-u,t+u+v); (y,x,z,t,v,u); (y,-x,-z,v,t,-t-u-v); (-y,x,-z,u,-t-u-v,t); (-y,-x,z,-t-u-v,u,v); (x,z,y,t,u,-t-u-v); (x,-z,-y,u,t,v); (-x,z,-y,-t-u-v,v,t); (-x,-z,y,v,-t-u-v,u); (z,y,x,t,-t-u-v,v); (z,-y,-x,-t-u-v,t,u); (-z,y,-x,v,u,t); (-z,-y,x,u,v,-t-u-v)

SUPERCENTERED SETTING
Modulation vectors: Q1'=(A,0,0), Q2'=(0,A,0), Q3'=(0,0,A), where A=a
Centering: (0,0,0,0,0,0); (0,1/2,1/2,0,0,0); (1/2,0,1/2,0,0,0); (1/2,1/2,0,0,0,0); (0,0,0,1/2,1/2,0); (0,1/2,1/2,1/2,1/2,0); (1/2,0,1/2,1/2,1/2,0); (1/2,1/2,0,1/2,1/2,0); (0,0,0,1/2,0,1/2); (0,1/2,1/2,1/2,0,1/2); (1/2,0,1/2,1/2,0,1/2); (1/2,1/2,0,1/2,0,1/2); (0,0,0,0,1/2,1/2); (0,1/2,1/2,0,1/2,1/2); (1/2,0,1/2,0,1/2,1/2); (1/2,1/2,0,0,1/2,1/2)
Non-lattice generators: (X,Y,-Z,T,U,-V); (-Z,-X,-Y,-V,-T,-U); (Y,X,Z,U,T,V)

Non-lattice operators: (X,Y,Z,T,U,V); (X,-Y,-Z,T,-U,-V); (-X,Y,-Z,-T,U,-V); (-X,-Y,Z,-T,-U,V); (Y,Z,X,U,V,T); (Y,-Z,-X,U,-V,-T); (-Y,Z,-X,-U,V,-T); (-Y,-Z,X,-U,-V,T); (Z,X,Y,V,T,U); (Z,-X,-Y,V,-T,-U); (-Z,X,-Y,-V,T,-U); (-Z,-X,Y,-V,-T,U); (-Y,-X,-Z,-U,-T,-V); (-Y,X,Z,-U,T,V); (Y,-X,Z,U,-T,V); (Y,X,-Z,U,T,-V); (-X,-Z,-Y,-T,-V,-U); (-X,Z,Y,-T,V,U); (X,-Z,Y,T,-V,U); (X,Z,-Y,T,V,-U); (-Z,-Y,-X,-V,-U,-T); (-Z,Y,X,-V,U,T); (Z,-Y,X,V,-U,T); (Z,Y,-X,V,U,-T); (-X,-Y,-Z,-T,-U,-V); (-X,Y,Z,-T,U,V); (X,-Y,Z,T,-U,V); (X,Y,-Z,T,U,-V); (-Y,-Z,-X,-U,-V,-T); (-Y,Z,X,-U,V,T); (Y,-Z,X,U,-V,T); (Y,Z,-X,U,V,-T); (-Z,-X,-Y,-V,-T,-U); (-Z,X,Y,-V,T,U); (Z,-X,Y,V,-T,U); (Z,X,-Y,V,T,-U); (Y,X,Z,U,T,V); (Y,-X,-Z,U,-T,-V); (-Y,X,-Z,-U,T,-V); (-Y,-X,Z,-U,-T,V); (X,Z,Y,T,V,U); (X,-Z,-Y,T,-V,-U); (-X,Z,-Y,-T,V,-U); (-X,-Z,Y,-T,-V,U); (Z,Y,X,V,U,T); (Z,-Y,-X,V,-U,-T); (-Z,Y,-X,-V,U,-T); (-Z,-Y,X,-V,-U,T)
Reflection conditions: HKLMNP:H+K=2n; HKLMNP:H+L=2n; HKLMNP:M+N=2n; HKLMNP:M+P=2n

Affine transformation to standard basic space group setting

$S * g(input) * S^{-1} = g(standard)$,
where g is an augmented matrix for an operation in the superspace group.
Also, S * r(input) = r(standard),
where r is an augmented position vector, (x,y,z,t,u,v,1).

$$S = \begin{matrix} 1&0&0&0&0&0&0\\ 0&1&0&0&0&0&0\\ 0&0&1&0&0&0&0\\ 0&0&0&1&0&0&0\\ 0&0&0&0&1&0&0\\ 0&0&0&0&0&1&0\\ 0&0&0&0&0&0&1 \end{matrix} \qquad S^{-1} = \begin{matrix} 1&0&0&0&0&0&0\\ 0&1&0&0&0&0&0\\ 0&0&1&0&0&0&0\\ 0&0&0&1&0&0&0\\ 0&0&0&0&1&0&0\\ 0&0&0&0&0&1&0\\ 0&0&0&0&0&0&1 \end{matrix}$$

a1' = a1	a1*' = a1*	q1' = q1 = (a,a,a)
a2' = a2	a2*' = a2*	q2' = q2 = (a,-a,-a)
a3' = a3	a3*' = a3*	q3' = q3 = (-a,a,-a)

a1 = a1'	a1* = a1*'	q1 = q1' = (a,a,a)
a2 = a2'	a2* = a2*'	q2 = q2' = (a,-a,-a)
a3 = a3'	a3* = a3*'	q3 = q3' = (-a,a,-a)

findssg Xm-3m(a,0,0)000(0,a,0)000(0,0,a)000

Generators of the standard supercentered setting have been entered into findssg.

Input setting

Centering
(0,0,0,0,0,0); (1/2,1/2,0,0,0,0); (1/2,0,1/2,0,0,0); (0,1/2,1/2,0,0,0); (0,0,0,1/2,1/2,0); (0,1/2,1/2,1/2,1/2,0); (1/2,0,1/2,1/2,1/2,0); (1/2,1/2,0,1/2,1/2,0); (0,0,0,1/2,0,1/2); (0,1/2,1/2,1/2,0,1/2); (1/2,0,1/2,1/2,0,1/2); (1/2,1/2,0,1/2,0,1/2); (0,0,0,0,1/2,1/2); (0,1/2,1/2,0,1/2,1/2); (1/2,0,1/2,0,1/2,1/2); (1/2,1/2,0,0,1/2,1/2)
Operators
(x,y,-z,t,u,-v); (-z,-x,-y,-v,-t,-u); (y,x,z,u,t,v); (x,y,z,t,u,v); (-z,-x,y,-v,-t,u); (y,x,-z,u,t,-v); (z,-x,-y,v,-t,-u); (y,z,x,u,v,t); (-z,-y,-x,-v,-u,-t); (-y,z,x,-u,v,t); (z,-y,-x,v,-u,-t); (-x,-z,-y,-t,-v,-u); (-x,-z,y,-t,-v,u); (-x,z,-y,-t,v,-u); (z,y,x,v,u,t); (-y,-z,-x,-u,-v,-t); (z,-y,x,v,-u,t); (-y,z,-x,-u,v,-t); (z,-x,y,v,-t,u); (y,z,-x,u,v,-t); (-z,-y,x,-v,-u,t); (-x,z,y,-t,v,u); (z,y,-x,v,u,-t); (-y,-z,x,-u,-v,t); (y,-z,x,u,-v,t); (-x,-y,-z,-t,-u,-v); (x,z,y,t,v,u); (-x,y,-z,-t,u,-v); (x,-z,y,t,-v,u); (-y,x,z,-u,t,v); (x,-y,-z,t,-u,-v); (-y,x,-z,-u,t,-v); (x,-z,-y,t,-v,-u); (-x,y,z,-t,u,v); (-z,y,x,-v,u,t); (-y,-x,-z,-u,-t,-v); (z,x,y,v,t,u); (y,-x,-z,u,-t,-v); (-z,x,y,-v,t,u); (x,-y,z,t,-u,v); (-z,x,-y,-v,t,-u); (y,-x,z,u,-t,v); (y,-z,-x,u,-v,-t); (-x,-y,z,-t,-u,v); (x,z,-y,t,v,-u); (z,x,-y,v,t,-u); (-y,-x,z,-u,-t,v); (-z,y,-x,-v,u,-t)

Standard settings

Superspace group: 225.3.215.7 Fm-3m(a,a,a)000(a,-a,-a)000(-a,a,-a)000 [Y:none]
Bravais class: 3.215 Fm-3m(a,a,a)(a,-a,-a)(-a,a,-a) [JJdW:3.217]
Transformation to supercentered setting: A1=a1, A2=a2, A3=a3, A4=a4+a5-a6, A5=a4-a5+a6, A6=a4-a5-a6

BASIC SPACE GROUP SETTING
Modulation vectors: q1'=(a,a,a), q2'=(a,-a,-a), q3'=(-a,a,-a)
Centering: (0,0,0,0,0,0); (0,1/2,1/2,0,0,0); (1/2,0,1/2,0,0,0); (1/2,1/2,0,0,0,0)
Non-lattice generators: (x,y,-z,t+u+v,-v,-u); (-z,-x,-y,-t,t+u+v,-u); (y,x,z,t,v,u)
Non-lattice operators: (x,y,z,t,u,v); (x,-y,-z,u,t,-t-u-v); (-x,y,-z,v,-t-u-v,t); (-x,-y,z,-t-u-v,v,u); (y,z,x,t,v,-t-u-v); (y,-z,-x,v,t,u); (-y,z,-x,-t-u-v,u,t); (-y,-z,x,u,-t-u-v,v); (z,x,y,t,-t-u-v,u); (z,-x,-y,-t-u-v,t,v); (-z,x,-y,u,v,t); (-z,-x,y,v,u,-t-u-v); (-y,-x,-z,-t,-v,-u); (-y,x,z,-v,-t,t+u+v); (y,-x,z,-u,t+u+v,-t); (y,x,-z,t+u+v,-u,-v); (-x,-z,-y,-t,-u,t+u+v); (-x,z,y,-u,-t,-v); (x,-z,y,t+u+v,-v,-t); (x,z,-y,-v,t+u+v,-u); (-z,-y,-x,-t,t+u+v,-v); (-z,y,x,t+u+v,-t,-u); (z,-y,x,-v,-u,-t); (z,y,-x,-u,-v,t+u+v); (-x,-y,-z,-t,-u,-v); (-x,y,z,-u,-t,t+u+v); (x,-y,z,-v,t+u+v,-t); (x,y,-z,t+u+v,-v,-u); (-y,-z,-x,-t,-v,t+u+v); (-y,z,x,-v,-t,-u); (y,-z,x,t+u+v,-u,-t); (y,z,-x,-u,t+u+v,-v); (-z,-x,-y,-t,t+u+v,-u); (-z,x,y,t+u+v,-t,-v); (z,-x,y,-u,-v,-t); (z,x,-y,-v,-u,t+u+v); (y,x,z,t,v,u); (y,-x,-z,v,t,-t-u-v); (-y,x,-z,u,-t-u-v,t); (-y,-x,z,-t-u-v,u,v); (x,z,y,t,u,-t-u-v); (x,-z,-y,u,t,v); (-x,z,-y,-t-u-v,v,t); (-x,-z,y,v,-t-u-v,u); (z,y,x,t,-t-u-v,v); (z,-y,-x,-t-u-v,t,u); (-z,y,-x,v,u,t); (-z,-y,x,u,v,-t-u-v)

SUPERCENTERED SETTING
Modulation vectors: Q1'=(A,0,0), Q2'=(0,A,0), Q3'=(0,0,A), where A=a
Centering: (0,0,0,0,0,0); (0,1/2,1/2,0,0,0); (1/2,0,1/2,0,0,0); (1/2,1/2,0,0,0,0); (0,0,0,1/2,1/2,0); (0,1/2,1/2,1/2,1/2,0); (1/2,0,1/2,1/2,1/2,0); (1/2,1/2,0,1/2,1/2,0); (0,0,0,1/2,0,1/2); (0,1/2,1/2,1/2,0,1/2); (1/2,0,1/2,1/2,0,1/2); (1/2,1/2,0,1/2,0,1/2); (0,0,0,0,1/2,1/2); (0,1/2,1/2,0,1/2,1/2); (1/2,0,1/2,0,1/2,1/2); (1/2,1/2,0,0,1/2,1/2)

Non-lattice generators: (X,Y,-Z,T,U,-V); (-Z,-X,-Y,-V,-T,-U); (Y,X,Z,U,T,V)
Non-lattice operators: (X,Y,Z,T,U,V); (X,-Y,-Z,T,-U,-V); (-X,Y,-Z,-T,U,-V); (-X,-Y,Z,-T,-U,V); (Y,Z,X,U,V,T); (Y,-Z,-X,U,-V,-T); (-Y,Z,-X,-U,V,-T); (-Y,-Z,X,-U,-V,T); (Z,X,Y,V,T,U); (Z,-X,-Y,V,-T,-U); (-Z,X,-Y,-V,T,-U); (-Z,-X,Y,-V,-T,U); (-Y,-X,-Z,-U,-T,-V); (-Y,X,Z,-U,T,V); (Y,-X,Z,U,-T,V); (Y,X,-Z,U,T,-V); (-X,-Z,-Y,-T,-V,-U); (-X,Z,Y,-T,V,U); (X,-Z,Y,T,-V,U); (X,Z,-Y,T,V,-U); (-Z,-Y,-X,-V,-U,-T); (-Z,Y,X,-V,U,T); (Z,-Y,X,V,-U,T); (Z,Y,-X,V,U,-T); (-X,-Y,-Z,-T,-U,-V); (-X,Y,Z,-T,U,V); (X,-Y,Z,T,-U,V); (X,Y,-Z,T,U,-V); (-Y,-Z,-X,-U,-V,-T); (-Y,Z,X,-U,V,T); (Y,-Z,X,U,-V,T); (Y,Z,-X,U,V,-T); (-Z,-X,-Y,-V,-T,-U); (-Z,X,Y,-V,T,U); (Z,-X,Y,V,-T,U); (Z,X,-Y,V,T,-U); (Y,X,Z,U,T,V); (Y,-X,-Z,U,-T,-V); (-Y,X,-Z,-U,T,-V); (-Y,-X,Z,-U,-T,V); (X,Z,Y,T,V,U); (X,-Z,-Y,T,-V,-U); (-X,Z,-Y,-T,V,-U); (-X,-Z,Y,-T,-V,U); (Z,Y,X,V,U,T); (Z,-Y,-X,V,-U,-T); (-Z,Y,-X,-V,U,-T); (-Z,-Y,X,-V,-U,T)
Reflection conditions: HKLMNP:H+K=2n; HKLMNP:H+L=2n; HKLMNP:M+N=2n; HKLMNP:M+P=2n

Affine transformation to standard basic space group setting

$S * g(\text{input}) * S^{-1} = g(\text{standard})$,
where g is an augmented matrix for an operation in the superspace group.
Also, $S * r(\text{input}) = r(\text{standard})$,
where r is an augmented position vector, (x,y,z,t,u,v,1).

$$S = \begin{matrix} 1 & 0 & 0 & 0 & 0 & 0 & 0 \\ 0 & 0 & 1 & 0 & 0 & 0 & 0 \\ 0 & -1 & 0 & 0 & 0 & 0 & 0 \\ 0 & 0 & 0 & 1 & -1 & 1 & 0 \\ 0 & 0 & 0 & 1 & 1 & -1 & 0 \\ 0 & 0 & 0 & -1 & 1 & 1 & 0 \\ 0 & 0 & 0 & 0 & 0 & 0 & 1 \end{matrix} \qquad S^{-1} = \begin{matrix} 1 & 0 & 0 & 0 & 0 & 0 & 0 \\ 0 & 0 & -1 & 0 & 0 & 0 & 0 \\ 0 & 1 & 0 & 0 & 0 & 0 & 0 \\ 0 & 0 & 0 & 1/2 & 1/2 & 0 & 0 \\ 0 & 0 & 0 & 0 & 1/2 & 1/2 & 0 \\ 0 & 0 & 0 & 1/2 & 0 & 1/2 & 0 \\ 0 & 0 & 0 & 0 & 0 & 0 & 1 \end{matrix}$$

a1' = a1	a1*' = a1*	q1' = q1 - q2 + q3 = (a,a,a)
a2' = a3	a2*' = a3*	q2' = q1 + q2 - q3 = (a,-a,-a)
a3' = -a2	a3*' = -a2*	q3' = -q1 + q2 + q3 = (-a,a,-a)

a1 = a1'	a1* = a1*'	q1 = 1/2 q1' + 1/2 q2' = (a,0,0)
a2 = -a3'	a2* = -a3*'	q2 = 1/2 q2' + 1/2 q3' = (0,a,0)
a3 = a2'	a3* = a2*'	q3 = 1/2 q1' + 1/2 q3' = (0,0,a)
