# Supplementary material for: Equivalence of superspace groups
Source: Acta Crystallogr A. 2012 Nov 14;69(Pt 1):75–90. doi: 10.1107/S0108767312041657 (PMC3553647; doi:10.1107/S0108767312041657)
Supplement: Supplementary file 1 [file a-69-00075-sup1.zip › ssg3d_im3m_aaa_bi3bao55.pdf]

229.3.211.5 Im-3m(0,b,b)000(b,0,b)000(b,b,0)000

Superspace group: 229.3.211.5 Im-3m(0,b,b)000(b,0,b)000(b,b,0)000 [Y:3.11762]
Bravais class: 3.211 Im-3m(0,b,b)(b,0,b)(b,b,0) [JJdW:3.213]
Transformation to supercentered setting: A1=a1, A2=a2, A3=a3, A4=a5+a6, A5=a4+a6, A6=a4+a5

BASIC SPACE GROUP SETTING
Modulation vectors: q1=(0,b,b), q2=(b,0,b), q3=(b,b,0)
Centering: (0,0,0,0,0,0); (1/2,1/2,1/2,0,0,0)
Non-lattice generators: (x,y,-z,-u+v,-t+v,v); (-z,-x,-y,-v,-t,-u); (y,x,z,u,t,v)
Non-lattice operators: (x,y,z,t,u,v); (x,-y,-z,-t,-t+v,-t+u); (-x,y,-z,-u+v,-u,t-u); (-x,-y,z,u-v,t-v,-v); (y,z,x,u,v,t); (y,-z,-x,-u,t-u,-u+v); (-y,z,-x,t-v,-v,u-v); (-y,-z,x,-t+v,-t+u,-t); (z,x,y,v,t,u); (z,-x,-y,-v,u-v,t-v); (-z,x,-y,-t+u,-t,-t+v); (-z,-x,y,t-u,-u+v,-u); (-y,-x,-z,-u,-t,-v); (-y,x,z,u,u-v,-t+u); (y,-x,z,t-v,t,t-u); (y,x,-z,-t+v,-u+v,v); (-x,-z,-y,-t,-v,-u); (-x,z,y,t,t-u,t-v); (x,-z,y,-u+v,v,-t+v); (x,z,-y,u-v,-t+u,u); (-z,-y,-x,-v,-u,-t); (-z,y,x,v,-t+v,-u+v); (z,-y,x,-t+u,u,u-v); (z,y,-x,t-u,t-v,t); (-x,-y,-z,-t,-u,-v); (-x,y,z,t,t-v,t-u); (x,-y,z,u-v,u,-t+u); (x,y,-z,-u+v,-t+v,v); (-y,-z,-x,-u,-v,-t); (-y,z,x,u,-t+u,u-v); (y,-z,x,-t+v,v,-u+v); (y,z,-x,t-v,t-u,t); (-z,-x,-y,-v,-t,-u); (-z,x,y,v,-u+v,-t+v); (z,-x,y,t-u,t,t-v); (z,x,-y,-t+u,u-v,u); (y,x,z,u,t,v); (y,-x,-z,-u,-u+v,t-u); (-y,x,-z,-t+v,-t,-t+u); (-y,-x,z,t-v,u-v,-v); (x,z,y,t,v,u); (x,-z,-y,-t,-t+u,-t+v); (-x,z,-y,u-v,-v,t-v); (-x,-z,y,-u+v,t-u,-u); (z,y,x,v,u,t); (z,-y,-x,-v,t-v,u-v); (-z,y,-x,t-u,-u,-u+v); (-z,-y,x,-t+u,-t+v,-t)

SUPERCENTERED SETTING
Modulation vectors: Q1=(B,0,0), Q2=(0,B,0), Q3=(0,0,B), where B=b
Centering: (0,0,0,0,0,0); (1/2,1/2,1/2,0,0,0); (0,0,0,1/2,1/2,1/2); (1/2,1/2,1/2,1/2,1/2,1/2)
Non-lattice generators: (X,Y,-Z,T,U,-V); (-Z,-X,-Y,-V,-T,-U); (Y,X,Z,U,T,V)
Non-lattice operators: (X,Y,Z,T,U,V); (X,-Y,-Z,T,-U,-V); (-X,Y,-Z,-T,U,-V); (-X,-Y,Z,-T,-U,V); (Y,Z,X,U,V,T); (Y,-Z,-X,U,-V,-T); (-Y,Z,-X,-U,V,-T); (-Y,-Z,X,-U,-V,T); (Z,X,Y,V,T,U); (Z,-X,-Y,V,-T,-U); (-Z,X,-Y,-V,T,-U); (-Z,-X,Y,-V,-T,U); (-Y,-X,-Z,-U,-T,-V); (-Y,X,Z,-U,T,V); (Y,-X,Z,U,-T,V); (Y,X,-Z,U,T,-V); (-X,-Z,-Y,-T,-V,-U); (-X,Z,Y,-T,V,U); (X,-Z,Y,T,-V,U); (X,Z,-Y,T,V,-U); (-Z,-Y,-X,-V,-U,-T); (-Z,Y,X,-V,U,T); (Z,-Y,X,V,-U,T); (Z,Y,-X,V,U,-T); (-X,-Y,-Z,-T,-U,-V); (-X,Y,Z,-T,U,V); (X,-Y,Z,T,-U,V); (X,Y,-Z,T,U,-V); (-Y,-Z,-X,-U,-V,-T); (-Y,Z,X,-U,V,T); (Y,-Z,X,U,-V,T); (Y,Z,-X,U,V,-T); (-Z,-X,-Y,-V,-T,-U); (-Z,X,Y,-V,T,U); (Z,-X,Y,V,-T,U); (Z,X,-Y,V,T,-U); (Y,X,Z,U,T,V); (Y,-X,-Z,U,-T,-V); (-Y,X,-Z,-U,T,-V); (-Y,-X,Z,-U,-T,V); (X,Z,Y,T,V,U); (X,-Z,-Y,T,-V,-U); (-X,Z,-Y,-T,V,-U); (-X,-Z,Y,-T,-V,U); (Z,Y,X,V,U,T); (Z,-Y,-X,V,-U,-T); (-Z,Y,-X,-V,U,-T); (-Z,-Y,X,-V,-U,T)
Reflection conditions: HKLMNP:H+K+L=2n; HKLMNP:M+N+P=2n

Two SSG exists with the same BSG and Bravais class but with different intrinsic translational components:
229.3.211.5 Im-3m(0,b,b)000(b,0,b)000(b,b,0)000
229.3.211.6 Im-3m(0,b,b)00q(b,0,b)s0q(b,b,0)00q
Only No. 229.3.211.5 fulfills the reported reflection conditions for BaBi3O5.5 Esmaeilzadeh, JSSC 152, 435-440 (2000).

SSG from Yamamoto: 11762 Im3m(0pp, p0p, pp0)mtm

11762 Im3m(0pp,p0p,pp0)mtm

(000000;0001/21/21/2;1/21/21/2000;1/21/21/21/21/21/2)

x,y,z,t,u,v; -x,-y,z,-t,-u,v; -x,y,-z,-t,u,-v; x,-y,-z,t,-u,-v; z,x,y,v,t,u; z,-x,-y,v,-t,-u; -z,-x,y,-v,-t,u; -z,x,-y,-v,t,-u; y,z,x,u,v,t; -y,z,-x,-u,v,-t; y,-z,-x,u,-v,-t; -y,-z,x,-u,-v,t; y,x,-z,u,t,-v; -y,-x,-z,-u,-t,-v; y,-x,z,u,-t,v; -y,x,z,-u,t,v; x,z,-y,t,v,-u; -x,z,y,-t,v,u; -x,-z,-y,-t,-v,-u; x,-z,y,t,-v,u; z,y,-x,v,u,-t; z,-y,x,v,-u,t; -z,y,x,-v,u,t; -z,-y,-x,-v,-u,-t; -x,-y,-z,-t,-u,-v; x,y,-z,t,u,-v; x,-y,z,t,-u,v; -x,y,z,-t,u,v; -z,-x,-y,-v,-t,-u; -z,x,y,-v,t,u; z,x,-y,v,t,-u; z,-x,y,v,-t,u; -y,-z,-x,-u,-v,-t; y,-z,x,u,-v,t; -y,z,x,-u,v,t; y,z,-x,u,v,-t; -y,-x,z,-u,-t,v; y,x,z,u,t,v; -y,x,-z,-u,t,-v; y,-x,-z,u,-t,-v; -x,-z,y,-t,-v,u; x,-z,-y,t,-v,-u; x,z,y,t,v,u; -x,z,-y,-t,v,-u; -z,-y,x,-v,-u,t; -z,y,-x,-v,u,-t; z,-y,-x,v,-u,-t; z,y,x,v,u,t;

hklmni:m+n+i=2n hklmni:h+k+l=2n hklmni:h+k+l+m+n+i=2n

-----

findssg Im-3m(0,b,b)000(b,0,b)000(b,b,0)000

Generators of the standard BSG setting have been entered into findssg.

Input setting

Centering
(0,0,0,0,0,0); (1/2,1/2,1/2,0,0,0)
Operators
(x,y,-z,-u+v,-t+v,v); (-z,-x,-y,-v,-t,-u); (y,x,z,u,t,v); (x,y,z,t,u,v); (-z,-x,y,t-u,-u+v,-u); (y,x,-z,-t+v,-u+v,v); (z,-x,-y,-v,u-v,t-v); (y,z,x,u,v,t); (-z,-y,-x,-v,-u,-t); (-y,z,x,u,-t+u,u-v); (z,-y,-x,-v,t-v,u-v); (-x,-z,-y,-t,-v,-u); (-x,-z,y,-u+v,t-u,-u); (-x,z,-y,u-v,-v,t-v); (z,y,x,v,u,t); (-y,-z,-x,-u,-v,-t); (z,-y,x,-t+u,u,u-v); (-y,z,-x,t-v,-v,u-v); (z,-x,y,t-u,t,t-v); (y,z,-x,t-v,t-u,t); (-z,-y,x,-t+u,-t+v,-t); (-x,z,y,t,t-u,t-v); (z,y,-x,t-u,t-v,t); (-y,-z,x,-t+v,-t+u,-t); (y,-z,x,-t+v,v,-u+v); (-x,-y,-z,-t,-u,-v); (x,z,y,t,v,u); (-x,y,-z,-u+v,-u,t-u); (x,-z,y,-u+v,v,-t+v); (-y,x,z,u,u-v,-t+u); (x,-y,-z,-t,-t+v,-t+u); (-y,x,-z,-t+v,-t,-t+u); (x,-z,-y,-t,-t+u,-t+v); (-x,y,z,t,t-v,t-u); (-z,y,x,v,-t+v,-u+v); (-y,-x,-z,-u,-t,-v); (z,x,y,v,t,u); (y,-x,-z,-u,-u+v,t-u); (-z,x,y,v,-u+v,-t+v); (x,-y,z,u-v,u,-t+u); (-z,x,-y,-t+u,-t,-t+v); (y,-x,z,t-v,t,t-u); (y,-z,-x,-u,t-u,-u+v); (-x,-y,z,u-v,t-v,-v); (x,z,-y,u-v,-t+u,u); (z,x,-y,-t+u,u-v,u); (-y,-x,z,t-v,u-v,-v); (-z,y,-x,t-u,-u,-u+v)

Standard settings

Superspace group: 229.3.211.5 Im-3m(0,b,b)000(b,0,b)000(b,b,0)000 [Y:3.11762]
Bravais class: 3.211 Im-3m(0,b,b)(b,0,b)(b,b,0) [JJdW:3.213]
Transformation to supercentered setting: A1=a1, A2=a2, A3=a3, A4=a5+a6, A5=a4+a6, A6=a4+a5

BASIC SPACE GROUP SETTING
Modulation vectors: q1'=(0,b,b), q2'=(b,0,b), q3'=(b,b,0)
Centering: (0,0,0,0,0,0); (1/2,1/2,1/2,0,0,0)
Non-lattice generators: (x,y,-z,-u+v,-t+v,v); (-z,-x,-y,-v,-t,-u); (y,x,z,u,t,v)
Non-lattice operators: (x,y,z,t,u,v); (x,-y,-z,-t,-t+v,-t+u); (-x,y,-z,-u+v,-u,t-u); (-x,-y,z,u-v,t-v,-v); (y,z,x,u,v,t); (y,-z,-x,-u,t-u,-u+v); (-y,z,-x,t-v,-v,u-v); (-y,-z,x,-t+v,-t+u,-t); (z,x,y,v,t,u); (z,-x,-y,-v,u-v,t-v); (-z,x,-y,-t+u,-t,-t+v); (-z,-x,y,t-u,-u+v,-u); (-y,-x,-z,-u,-t,-v); (-y,x,z,u,u-v,-t+u); (y,-x,z,t-v,t,t-u); (y,x,-z,-t+v,-u+v,v); (-x,-z,-y,-t,-v,-u); (-x,z,y,t,t-u,t-v); (x,-z,y,-u+v,v,-t+v); (x,z,-y,u-v,-t+u,u); (-z,-y,-x,-v,-u,-t); (-z,y,x,v,-t+v,-u+v); (z,-y,x,-t+u,u,u-v); (z,y,-x,t-u,t-v,t); (-x,-y,-z,-t,-u,-v); (-x,y,z,t,t-v,t-u); (x,-y,z,u-v,u,-t+u); (x,y,-z,-u+v,-t+v,v); (-y,-z,-x,-u,-v,-t); (-y,z,x,u,-t+u,u-v); (y,-z,x,-t+v,v,-u+v); (y,z,-x,t-v,t-u,t); (-z,-x,-y,-v,-t,-u); (-z,x,y,v,-u+v,-t+v); (z,-x,y,t-u,t,t-v); (z,x,-y,-t+u,u-v,u); (y,x,z,u,t,v); (y,-x,-z,-u,-u+v,t-u); (-y,x,-z,-t+v,-t,-t+u); (-y,-x,z,t-v,u-v,-v); (x,z,y,t,v,u); (x,-z,-y,-t,-t+u,-t+v); (-x,z,-y,u-v,-v,t-v); (-x,-z,y,-u+v,t-u,-u); (z,y,x,v,u,t); (z,-y,-x,-v,t-v,u-v); (-z,y,-x,t-u,-u,-u+v); (-z,-y,x,-t+u,-t+v,-t)

SUPERCENTERED SETTING
Modulation vectors: Q1'=(B,0,0), Q2'=(0,B,0), Q3'=(0,0,B), where B=b
Centering: (0,0,0,0,0,0); (1/2,1/2,1/2,0,0,0); (0,0,0,1/2,1/2,1/2); (1/2,1/2,1/2,1/2,1/2,1/2)
Non-lattice generators: (X,Y,-Z,T,U,-V); (-Z,-X,-Y,-V,-T,-U); (Y,X,Z,U,T,V)
Non-lattice operators: (X,Y,Z,T,U,V); (X,-Y,-Z,T,-U,-V); (-X,Y,-Z,-T,U,-V); (-X,-Y,Z,-T,-U,V); (Y,Z,X,U,V,T); (Y,-Z,-X,U,-V,-T); (-Y,Z,-X,-U,V,-T); (-Y,-Z,X,-U,-V,T); (Z,X,Y,V,T,U); (Z,-X,-Y,V,-T,-U); (-Z,X,-Y,-V,T,-U); (-Z,-X,Y,-V,-T,U); (-Y,-X,-Z,-U,-T,-V); (-Y,X,Z,-U,T,V); (Y,-X,Z,U,-T,V); (Y,X,-Z,U,T,-V); (-X,-Z,-Y,-T,-V,-U); (-X,Z,Y,-T,V,U); (X,-Z,Y,T,-V,U); (X,Z,-Y,T,V,-U); (-Z,-Y,-X,-V,-U,-T); (-Z,Y,X,-V,U,T); (Z,-Y,X,V,-U,T); (Z,Y,-X,V,U,-T); (-X,-Y,-Z,-T,-U,-V); (-X,Y,Z,-T,U,V); (X,-Y,Z,T,-U,V); (X,Y,-Z,T,U,-V); (-Y,-Z,-X,-U,-V,-T); (-Y,Z,X,-U,V,T); (Y,-Z,X,U,-V,T); (Y,Z,-X,U,V,-T); (-Z,-X,-Y,-V,-T,-U); (-Z,X,Y,-V,T,U); (Z,-X,Y,V,-T,U); (Z,X,-Y,V,T,-U); (Y,X,Z,U,T,V); (Y,-X,-Z,U,-T,-V); (-Y,X,-Z,-U,T,-V); (-Y,-X,Z,-U,-T,V); (X,Z,Y,T,V,U); (X,-Z,-Y,T,-V,-U); (-X,Z,-Y,-T,V,-U); (-X,-Z,Y,-T,-V,U); (Z,Y,X,V,U,T); (Z,-Y,-X,V,-U,-T); (-Z,Y,-X,-V,U,-T); (-Z,-Y,X,-V,-U,T)
Reflection conditions: HKLMNP:H+K+L=2n; HKLMNP:M+N+P=2n

Affine transformation to standard basic space group setting

$S * g(\text{input}) * S^{-1} = g(\text{standard})$,
where g is an augmented matrix for an operation in the superspace group.
Also, S * r(input) = r(standard),
where r is an augmented position vector, (x,y,z,t,u,v,1).

$$S = \begin{pmatrix} 1&0&0&0&0&0&0\\ 0&1&0&0&0&0&0\\ 0&0&1&0&0&0&0\\ 0&0&0&1&0&0&0\\ 0&0&0&0&1&0&0\\ 0&0&0&0&0&1&0\\ 0&0&0&0&0&0&1 \end{pmatrix} \quad S^{-1} = \begin{pmatrix} 1&0&0&0&0&0&0\\ 0&1&0&0&0&0&0\\ 0&0&1&0&0&0&0\\ 0&0&0&1&0&0&0\\ 0&0&0&0&1&0&0\\ 0&0&0&0&0&1&0\\ 0&0&0&0&0&0&1 \end{pmatrix}$$

a1' = a1	a1*' = a1*	q1' = q1 = (0,b,b)
a2' = a2	a2*' = a2*	q2' = q2 = (b,0,b)
a3' = a3	a3*' = a3*	q3' = q3 = (b,b,0)

a1 = a1'	a1* = a1*'	q1 = q1' = (0,b,b)
a2 = a2'	a2* = a2*'	q2 = q2' = (b,0,b)
a3 = a3'	a3* = a3*'	q3 = q3' = (b,b,0)

findssg Xm-3m(b,0,0)000(0,b,0)000(0,0,b)000

Generators of the standard supercentred setting have been entered into findssg.

Input setting

Centering
(0,0,0,0,0,0); (1/2,1/2,1/2,0,0,0); (0,0,0,1/2,1/2,1/2); (1/2,1/2,1/2,1/2,1/2,1/2)
Operators
(x,y,-z,t,u,-v); (-z,-x,-y,-v,-t,-u); (y,x,z,u,t,v); (x,y,z,t,u,v); (-z,-x,y,-v,-t,u); (y,x,-z,u,t,-v); (z,-x,-y,v,-t,-u); (y,z,x,u,v,t); (-z,-y,-x,-v,-u,-t); (-y,z,x,-u,v,t); (z,-y,-x,v,-u,-t); (-x,-z,-y,-t,-v,-u); (-x,-z,y,-t,-v,u); (-x,z,-y,-t,v,-u); (z,y,x,v,u,t); (-y,-z,-x,-u,-v,-t); (z,-y,x,v,-u,t); (-y,z,-x,-u,v,-t); (z,-x,y,v,-t,u); (y,z,-x,u,v,-t); (-z,-y,x,-v,-u,t); (-x,z,y,-t,v,u); (z,y,-x,v,u,-t); (-y,-z,x,-u,-v,t); (y,-z,x,u,-v,t); (-x,-y,-z,-t,-u,-v); (x,z,y,t,v,u); (-x,y,-z,-t,u,-v); (x,-z,y,t,-v,u); (-y,x,z,-u,t,v); (x,-y,-z,t,-u,-v); (-y,x,-z,-u,t,-v); (x,-z,-y,t,-v,-u); (-x,y,z,-t,u,v); (-z,y,x,-v,u,t); (-y,-x,-z,-u,-t,-v); (z,x,y,v,t,u); (y,-x,-z,u,-t,-v); (-z,x,y,-v,t,u); (x,-y,z,t,-u,v); (-z,x,-y,-v,t,-u); (y,-x,z,u,-t,v); (y,-z,-x,u,-v,-t); (-x,-y,z,-t,-u,v); (x,z,-y,t,v,-u); (z,x,-y,v,t,-u); (-y,-x,z,-u,-t,v); (-z,y,-x,-v,u,-t)

Standard settings

Superspace group: 229.3.211.5 Im-3m(0,b,b)000(b,0,b)000(b,b,0)000 [Y:3.11762]
Bravais class: 3.211 Im-3m(0,b,b)(b,0,b)(b,b,0) [JJdW:3.213]
Transformation to supercentered setting: A1=a1, A2=a2, A3=a3, A4=a5+a6, A5=a4+a6, A6=a4+a5

BASIC SPACE GROUP SETTING
Modulation vectors: q1'=(0,b,b), q2'=(b,0,b), q3'=(b,b,0)
Centering: (0,0,0,0,0,0); (1/2,1/2,1/2,0,0,0)
Non-lattice generators: (x,y,-z,-u+v,-t+v,v); (-z,-x,-y,-v,-t,-u); (y,x,z,u,t,v)
Non-lattice operators: (x,y,z,t,u,v); (x,-y,-z,-t,-t+v,-t+u); (-x,y,-z,-u+v,-u,t-u); (-x,-y,z,u-v,t-v,-v); (y,z,x,u,v,t); (y,-z,-x,-u,t-u,-u+v); (-y,z,-x,t-v,-v,u-v); (-y,-z,x,-t+v,-t+u,-t); (z,x,y,v,t,u); (z,-x,-y,-v,u-v,t-v); (-z,x,-y,-t+u,-t,-t+v); (-z,-x,y,t-u,-u+v,-u); (-y,-x,-z,-u,-t,-v); (-y,x,z,u,u-v,-t+u); (y,-x,z,t-v,t,t-u); (y,x,-z,-t+v,-u+v,v); (-x,-z,-y,-t,-v,-u); (-x,z,y,t,t-u,t-v); (x,-z,y,-u+v,v,-t+v); (x,z,-y,u-v,-t+u,u); (-z,-y,-x,-v,-u,-t); (-z,y,x,v,-t+v,-u+v); (z,-y,x,-t+u,u,u-v); (z,y,-x,t-u,t-v,t); (-x,-y,-z,-t,-u,-v); (-x,y,z,t,t-v,t-u); (x,-y,z,u-v,u,-t+u); (x,y,-z,-u+v,-t+v,v); (-y,-z,-x,-u,-v,-t); (-y,z,x,u,-t+u,u-v); (y,-z,x,-t+v,v,-u+v); (y,z,-x,t-v,t-u,t); (-z,-x,-y,-v,-t,-u); (-z,x,y,v,-u+v,-t+v); (z,-x,y,t-u,t,t-v); (z,x,-y,-t+u,u-v,u); (y,x,z,u,t,v); (y,-x,-z,-u,-u+v,t-u); (-y,x,-z,-t+v,-t,-t+u); (-y,-x,z,t-v,u-v,-v); (x,z,y,t,v,u); (x,-z,-y,-t,-t+u,-t+v); (-x,z,-y,u-v,-v,t-v); (-x,-z,y,-u+v,t-u,-u); (z,y,x,v,u,t); (z,-y,-x,-v,t-v,u-v); (-z,y,-x,t-u,-u,-u+v); (-z,-y,x,-t+u,-t+v,-t)

SUPERCENTERED SETTING
Modulation vectors: Q1'=(B,0,0), Q2'=(0,B,0), Q3'=(0,0,B), where B=b
Centering: (0,0,0,0,0,0); (1/2,1/2,1/2,0,0,0); (0,0,0,1/2,1/2,1/2); (1/2,1/2,1/2,1/2,1/2,1/2)
Non-lattice generators: (X,Y,-Z,T,U,-V); (-Z,-X,-Y,-V,-T,-U); (Y,X,Z,U,T,V)

Non-lattice operators: (X,Y,Z,T,U,V); (X,-Y,-Z,T,-U,-V); (-X,Y,-Z,-T,U,-V); (-X,-Y,Z,-T,-U,V); (Y,Z,X,U,V,T); (Y,-Z,-X,U,-V,-T); (-Y,Z,-X,-U,V,-T); (-Y,-Z,X,-U,-V,T); (Z,X,Y,V,T,U); (Z,-X,-Y,V,-T,-U); (-Z,X,-Y,-V,T,-U); (-Z,-X,Y,-V,-T,U); (-Y,-X,-Z,-U,-T,-V); (-Y,X,Z,-U,T,V); (Y,-X,Z,U,-T,V); (Y,X,-Z,U,T,-V); (-X,-Z,-Y,-T,-V,-U); (-X,Z,Y,-T,V,U); (X,-Z,Y,T,-V,U); (X,Z,-Y,T,V,-U); (-Z,-Y,-X,-V,-U,-T); (-Z,Y,X,-V,U,T); (Z,-Y,X,V,-U,T); (Z,Y,-X,V,U,-T); (-X,-Y,-Z,-T,-U,-V); (-X,Y,Z,-T,U,V); (X,-Y,Z,T,-U,V); (X,Y,-Z,T,U,-V); (-Y,-Z,-X,-U,-V,-T); (-Y,Z,X,-U,V,T); (Y,-Z,X,U,-V,T); (Y,Z,-X,U,V,-T); (-Z,-X,-Y,-V,-T,-U); (-Z,X,Y,-V,T,U); (Z,-X,Y,V,-T,U); (Z,X,-Y,V,T,-U); (Y,X,Z,U,T,V); (Y,-X,-Z,U,-T,-V); (-Y,X,-Z,-U,T,-V); (-Y,-X,Z,-U,-T,V); (X,Z,Y,T,V,U); (X,-Z,-Y,T,-V,-U); (-X,Z,-Y,-T,V,-U); (-X,-Z,Y,-T,-V,U); (Z,Y,X,V,U,T); (Z,-Y,-X,V,-U,-T); (-Z,Y,-X,-V,U,-T); (-Z,-Y,X,-V,-U,T)
Reflection conditions: HKLMNP:H+K+L=2n; HKLMNP:M+N+P=2n

Affine transformation to standard basic space group setting

S * g(input) * S $^{-1}$ = g(standard),
where g is an augmented matrix for an operation in the superspace group.
Also, S * r(input) = r(standard),
where r is an augmented position vector, (x,y,z,t,u,v,1).

$$
S = \begin{matrix} 0 & 0 & 1 & 0 & 0 & 0 & 0 \\ 1 & 0 & 0 & 0 & 0 & 0 & 0 \\ 0 & 1 & 0 & 0 & 0 & 0 & 0 \\ 0 & 0 & 0 & 1 & 1 & 0 & 0 \\ 0 & 0 & 0 & 0 & 1 & 1 & 0 \\ 0 & 0 & 0 & 1 & 0 & 1 & 0 \\ 0 & 0 & 0 & 0 & 0 & 0 & 1 \end{matrix} \qquad S^{-1} = \begin{matrix} 0 & 1 & 0 & 0 & 0 & 0 & 0 \\ 0 & 0 & 1 & 0 & 0 & 0 & 0 \\ 1 & 0 & 0 & 0 & 0 & 0 & 0 \\ 0 & 0 & 0 & 1/2 & -1/2 & 1/2 & 0 \\ 0 & 0 & 0 & 1/2 & 1/2 & -1/2 & 0 \\ 0 & 0 & 0 & -1/2 & 1/2 & 1/2 & 0 \\ 0 & 0 & 0 & 0 & 0 & 0 & 1 \end{matrix}
$$

a1' = a3	a1*' = a3*	q1' = q1 + q2 = (0,b,b)
a2' = a1	a2*' = a1*	q2' = q2 + q3 = (b,0,b)
a3' = a2	a3*' = a2*	q3' = q1 + q3 = (b,b,0)

a1 = a2'	a1* = a2*'	q1 = 1/2 q1' - 1/2 q2' + 1/2 q3' = (b,0,0)
a2 = a3'	a2* = a3*'	q2 = 1/2 q1' + 1/2 q2' - 1/2 q3' = (0,b,0)
a3 = a1'	a3* = a1*'	q3 = -1/2 q1' + 1/2 q2' + 1/2 q3' = (0,0,b)

findssg Im3m(0pp,p0p,pp0)mtm

Operators of Yamamoto: 11762 Im3m(0pp,p0p,pp0)mtm have been entered into findssg.

Input setting

Centering
(0,0,0,0,0,0); (1/2,1/2,1/2,0,0,0); (0,0,0,1/2,1/2,1/2); (1/2,1/2,1/2,1/2,1/2,1/2)
Operators
(-x,-y,z,-t,-u,v); (-x,y,-z,-t,u,-v); (x,-y,-z,t,-u,-v); (z,x,y,v,t,u); (z,-x,-y,v,-t,-u); (-z,-x,y,-v,-t,u); (-z,x,-y,-v,t,-u); (y,z,x,u,v,t); (-y,z,-x,-u,v,-t); (y,-z,-x,u,-v,-t); (-y,-z,x,-u,-v,t); (y,x,-z,u,t,-v); (-y,-x,-z,-u,-t,-v); (y,-x,z,u,-t,v); (-y,x,z,-u,t,v); (x,z,-y,t,v,-u); (-x,z,y,-t,v,u); (-x,-z,-y,-t,-v,-u); (x,-z,y,t,-v,u); (z,y,-x,v,u,-t); (z,-y,x,v,-u,t); (-z,y,x,-v,u,t); (-z,-y,-x,-v,-u,-t); (-x,-y,-z,-t,-u,-v); (x,y,-z,t,u,-v); (x,-y,z,t,-u,v); (-x,y,z,-t,u,v); (-z,-x,-y,-v,-t,-u); (-z,x,y,-v,t,u); (z,x,-y,v,t,-u); (z,-x,y,v,-t,u); (-y,-z,-x,-u,-v,-t); (y,-z,x,u,-v,t); (-y,z,x,-u,v,t); (y,z,-x,u,v,-t); (-y,-x,z,-u,-t,v); (y,x,z,u,t,v); (-y,x,-z,-u,t,-v); (y,-x,-z,u,-t,-v); (x,y,z,t,u,v); (z,-y,-x,v,-u,-t); (z,y,x,v,u,t); (-z,-y,x,-v,-u,t); (-z,y,-x,-v,u,-t); (-x,-z,y,-t,-v,u); (x,-z,-y,t,-v,-u); (x,z,y,t,v,u); (-x,z,-y,-t,v,-u)

Standard settings

Superspace group: 229.3.211.5 Im-3m(0,b,b)000(b,0,b)000(b,b,0)000 [Y:3.11762]
Bravais class: 3.211 Im-3m(0,b,b)(b,0,b)(b,b,0) [JJdW:3.213]
Transformation to supercentered setting: A1=a1, A2=a2, A3=a3, A4=a5+a6, A5=a4+a6, A6=a4+a5

BASIC SPACE GROUP SETTING
Modulation vectors: q1'=(0,b,b), q2'=(b,0,b), q3'=(b,b,0)
Centering: (0,0,0,0,0,0); (1/2,1/2,1/2,0,0,0)
Non-lattice generators: (x,y,-z,-u+v,-t+v,v); (-z,-x,-y,-v,-t,-u); (y,x,z,u,t,v)
Non-lattice operators: (x,y,z,t,u,v); (x,-y,-z,-t,-t+v,-t+u); (-x,y,-z,-u+v,-u,t-u); (-x,-y,z,u-v,t-v,-v); (y,z,x,u,v,t); (y,-z,-x,-u,t-u,-u+v); (-y,z,-x,t-v,-v,u-v); (-y,-z,x,-t+v,-t+u,-t); (z,x,y,v,t,u); (z,-x,-y,-v,u-v,t-v); (-z,x,-y,-t+u,-t,-t+v); (-z,-x,y,t-u,-u+v,-u); (-y,-x,-z,-u,-t,-v); (-y,x,z,u,u-v,-t+u); (y,-x,z,t-v,t,t-u); (y,x,-z,-t+v,-u+v,v); (-x,-z,-y,-t,-v,-u); (-x,z,y,t,t-u,t-v); (x,-z,y,-u+v,v,-t+v); (x,z,-y,u-v,-t+u,u); (-z,-y,-x,-v,-u,-t); (-z,y,x,v,-t+v,-u+v); (z,-y,x,-t+u,u,u-v); (z,y,-x,t-u,t-v,t); (-x,-y,-z,-t,-u,-v); (-x,y,z,t,t-v,t-u); (x,-y,z,u-v,u,-t+u); (x,y,-z,-u+v,-t+v,v); (-y,-z,-x,-u,-v,-t); (-y,z,x,u,-t+u,u-v); (y,-z,x,-t+v,v,-u+v); (y,z,-x,t-v,t-u,t); (-z,-x,-y,-v,-t,-u); (-z,x,y,v,-u+v,-t+v); (z,-x,y,t-u,t,t-v); (z,x,-y,-t+u,u-v,u); (y,x,z,u,t,v); (y,-x,-z,-u,-u+v,t-u); (-y,x,-z,-t+v,-t,-t+u); (-y,-x,z,t-v,u-v,-v); (x,z,y,t,v,u); (x,-z,-y,-t,-t+u,-t+v); (-x,z,-y,u-v,-v,t-v); (-x,-z,y,-u+v,t-u,-u); (z,y,x,v,u,t); (z,-y,-x,-v,t-v,u-v); (-z,y,-x,t-u,-u,-u+v); (-z,-y,x,-t+u,-t+v,-t)

SUPERCENTERED SETTING
Modulation vectors: Q1'=(B,0,0), Q2'=(0,B,0), Q3'=(0,0,B), where B=b
Centering: (0,0,0,0,0,0); (1/2,1/2,1/2,0,0,0); (0,0,0,1/2,1/2,1/2); (1/2,1/2,1/2,1/2,1/2,1/2)
Non-lattice generators: (X,Y,-Z,T,U,-V); (-Z,-X,-Y,-V,-T,-U); (Y,X,Z,U,T,V)

Non-lattice operators: (X,Y,Z,T,U,V); (X,-Y,-Z,T,-U,-V); (-X,Y,-Z,-T,U,-V); (-X,-Y,Z,-T,-U,V); (Y,Z,X,U,V,T); (Y,-Z,-X,U,-V,-T); (-Y,Z,-X,-U,V,-T); (-Y,-Z,X,-U,-V,T); (Z,X,Y,V,T,U); (Z,-X,-Y,V,-T,-U); (-Z,X,-Y,-V,T,-U); (-Z,-X,Y,-V,-T,U); (-Y,-X,-Z,-U,-T,-V); (-Y,X,Z,-U,T,V); (Y,-X,Z,U,-T,V); (Y,X,-Z,U,T,-V); (-X,-Z,-Y,-T,-V,-U); (-X,Z,Y,-T,V,U); (X,-Z,Y,T,-V,U); (X,Z,-Y,T,V,-U); (-Z,-Y,-X,-V,-U,-T); (-Z,Y,X,-V,U,T); (Z,-Y,X,V,-U,T); (Z,Y,-X,V,U,-T); (-X,-Y,-Z,-T,-U,-V); (-X,Y,Z,-T,U,V); (X,-Y,Z,T,-U,V); (X,Y,-Z,T,U,-V); (-Y,-Z,-X,-U,-V,-T); (-Y,Z,X,-U,V,T); (Y,-Z,X,U,-V,T); (Y,Z,-X,U,V,-T); (-Z,-X,-Y,-V,-T,-U); (-Z,X,Y,-V,T,U); (Z,-X,Y,V,-T,U); (Z,X,-Y,V,T,-U); (Y,X,Z,U,T,V); (Y,-X,-Z,U,-T,-V); (-Y,X,-Z,-U,T,-V); (-Y,-X,Z,-U,-T,V); (X,Z,Y,T,V,U); (X,-Z,-Y,T,-V,-U); (-X,Z,-Y,-T,V,-U); (-X,-Z,Y,-T,-V,U); (Z,Y,X,V,U,T); (Z,-Y,-X,V,-U,-T); (-Z,Y,-X,-V,U,-T); (-Z,-Y,X,-V,-U,T)
Reflection conditions: HKLMNP:H+K+L=2n; HKLMNP:M+N+P=2n

Affine transformation to standard basic space group setting

$S * g(\text{input}) * S^{-1} = g(\text{standard})$,
where g is an augmented matrix for an operation in the superspace group.
Also, S * r(input) = r(standard),
where r is an augmented position vector, (x,y,z,t,u,v,1).

$$S = \begin{pmatrix} 1&0&0&0&0&0&0 \\ 0&1&0&0&0&0&0 \\ 0&0&1&0&0&0&0 \\ 0&0&0&0&1&1&0 \\ 0&0&0&1&0&1&0 \\ 0&0&0&1&1&0&0 \\ 0&0&0&0&0&0&1 \end{pmatrix} \quad S^{-1} = \begin{pmatrix} 1&0&0&0&0&0&0 \\ 0&1&0&0&0&0&0 \\ 0&0&1&0&0&0&0 \\ 0&0&0&-1/2&1/2&1/2&0 \\ 0&0&0&1/2&-1/2&1/2&0 \\ 0&0&0&1/2&1/2&-1/2&0 \\ 0&0&0&0&0&0&1 \end{pmatrix}$$

a1' = a1	a1*' = a1*	q1' = q2 + q3 = (0,b,b)
a2' = a2	a2*' = a2*	q2' = q1 + q3 = (b,0,b)
a3' = a3	a3*' = a3*	q3' = q1 + q2 = (b,b,0)

a1 = a1'	a1* = a1*'	q1 = -1/2 q1' + 1/2 q2' + 1/2 q3' = (b,0,0)
a2 = a2'	a2* = a2*'	q2 = 1/2 q1' - 1/2 q2' + 1/2 q3' = (0,b,0)
a3 = a3'	a3* = a3*'	q3 = 1/2 q1' + 1/2 q2' - 1/2 q3' = (0,0,b)

229.3.211.6 Im-3m(0,b,b)00q(b,0,b)s0q(b,b,0)00q

Generators of the standard BSG setting have been entered into findssg.

Input setting

Centering
(0,0,0,0,0,0); (1/2,1/2,1/2,0,0,0)
Operators
(x,y,-z,-u+v,-t+v+1/2,v+1/2); (-z,-x,-y,-v,-t,-u); (y,x,z,u+1/2,t+1/2,v+1/2); (x,y,z,t,u,v); (-z,-x,y,t-u,-u+v+1/2,-u+1/2); (y,x,-z,-t+v,-u+v+1/2,v); (z,-x,-y,-v+1/2,u-v,t-v+1/2); (y,z,x,u,v,t); (-z,-y,-x,-v+1/2,-u+1/2,-t+1/2); (-y,z,x,u+1/2,-t+u,u-v+1/2); (z,-y,-x,-v,t-v,u-v+1/2); (-x,-z,-y,-t+1/2,-v+1/2,-u+1/2); (-x,-z,y,-u+v,t-u+1/2,-u); (-x,z,-y,u-v+1/2,-v,t-v); (z,y,x,v+1/2,u+1/2,t+1/2); (-y,-z,-x,-u,-v,-t); (z,-y,x,-t+u+1/2,u,u-v); (-y,z,-x,t-v+1/2,-v+1/2,u-v); (z,-x,y,t-u+1/2,t+1/2,t-v); (y,z,-x,t-v,t-u+1/2,t+1/2); (-z,-y,x,-t+u,-t+v+1/2,-t); (-x,z,y,t,t-u,t-v+1/2); (z,y,-x,t-u,t-v+1/2,t); (-y,-z,x,-t+v,-t+u+1/2,-t+1/2); (y,-z,x,-t+v+1/2,v+1/2,-u+v); (-x,-y,-z,-t,-u,-v); (x,z,y,t+1/2,v+1/2,u+1/2); (-x,y,-z,-u+v+1/2,-u+1/2,t-u); (x,-z,y,-u+v+1/2,v,-t+v); (-y,x,z,u,u-v,-t+u+1/2); (x,-y,-z,-t+1/2,-t+v,-t+u+1/2); (-y,x,-z,-t+v+1/2,-t,-t+u); (x,-z,-y,-t,-t+u,-t+v+1/2); (-x,y,z,t+1/2,t-v,t-u+1/2); (-z,y,x,v,-t+v,-u+v+1/2); (-y,-x,-z,-u+1/2,-t+1/2,-v+1/2); (z,x,y,v,t,u); (y,-x,-z,-u,-u+v,t-u+1/2); (-z,x,y,v+1/2,-u+v,-t+v+1/2); (x,-y,z,u-v+1/2,u+1/2,-t+u); (-z,x,-y,-t+u+1/2,-t+1/2,-t+v); (y,-x,z,t-v+1/2,t,t-u); (y,-z,-x,-u+1/2,t-u,-u+v+1/2); (-x,-y,z,u-v,t-v+1/2,-v+1/2); (x,z,-y,u-v,-t+u+1/2,u); (z,x,-y,-t+u,u-v+1/2,u+1/2); (-y,-x,z,t-v,u-v+1/2,-v); (-z,y,-x,t-u+1/2,-u,-u+v)

Standard settings

Superspace group: 229.3.211.6 Im-3m(0,b,b)00q(b,0,b)s0q(b,b,0)00q [Y:none]
Bravais class: 3.211 Im-3m(0,b,b)(b,0,b)(b,b,0)   [JJdW:3.213]
Transformation to supercentered setting: A1=a1, A2=a2, A3=a3, A4=a5+a6, A5=a4+a6, A6=a4+a5

BASIC SPACE GROUP SETTING
Modulation vectors: q1'=(0,b,b), q2'=(b,0,b), q3'=(b,b,0)
Centering: (0,0,0,0,0,0); (1/2,1/2,1/2,0,0,0)
Non-lattice generators: (x,y,-z,-u+v+1,-t+v+1/2,v+1/2); (-z,-x,-y,-v,-t,-u); (y,x,z,u+1/2,t+1/2,v+1/2)
Non-lattice operators: (x,y,z,t,u,v); (x,-y,-z,-t+1/2,-t+v,-t+u+1/2); (-x,y,-z,-u+v+1/2,-u+1/2,t-u); (-x,-y,z,u-v,t-v+1/2,-v+1/2); (y,z,x,u,v,t); (y,-z,-x,-u+1/2,t-u,-u+v+1/2); (-y,z,-x,t-v+1/2,-v+1/2,u-v); (-y,-z,x,-t+v,-t+u+1/2,-t+1/2); (z,x,y,v,t,u); (z,-x,-y,-v+1/2,u-v,t-v+1/2); (-z,x,-y,-t+u+1/2,-t+1/2,-t+v); (-z,-x,y,t-u,-u+v+1/2,-u+1/2); (-y,-x,-z,-u+1/2,-t+1/2,-v+1/2); (-y,x,z,u,u-v,-t+u+1/2); (y,-x,z,t-v+1/2,t,t-u); (y,x,-z,-t+v,-u+v+1/2,v); (-x,-z,-y,-t+1/2,-v+1/2,-u+1/2); (-x,z,y,t,t-u,t-v+1/2); (x,-z,y,-u+v+1/2,v,-t+v); (x,z,-y,u-v,-t+u+1/2,u); (-z,-y,-x,-v+1/2,-u+1/2,-t+1/2); (-z,y,x,v,-t+v,-u+v+1/2); (z,-y,x,-t+u+1/2,u,u-v); (z,y,-x,t-u,t-v+1/2,t); (-x,-y,-z,-t,-u,-v); (-x,y,z,t+1/2,t-v,t-u+1/2); (x,-y,z,u-v+1/2,u+1/2,-t+u); (x,y,-z,-u+v,-t+v+1/2,v+1/2); (-y,-z,-x,-u,-v,-t); (-y,z,x,u+1/2,-t+u,u-v+1/2); (y,-z,x,-t+v+1/2,v+1/2,-u+v); (y,z,-x,t-v,t-u+1/2,t+1/2); (-z,-x,-y,-v,-t,-u); (-z,x,y,v+1/2,-u+v,-t+v+1/2); (z,-x,y,t-u+1/2,t+1/2,t-v); (z,x,-y,-t+u,u-v+1/2,u+1/2); (y,x,z,u+1/2,t+1/2,v+1/2); (y,-x,-z,-u,-u+v,t-u+1/2); (-y,x,-z,-t+v+1/2,-t,-t+u); (-y,-x,z,t-v,u-v+1/2,-v); (x,z,y,t+1/2,v+1/2,u+1/2); (x,-z,-y,-t,-t+u,-t+v+1/2); (-x,z,-y,u-v+1/2,-v,t-v); (-x,-z,y,-u+v,t-u+1/2,-u); (z,y,x,v+1/2,u+1/2,t+1/2); (z,-y,-x,-v,t-v,u-v+1/2); (-z,y,-x,t-u+1/2,-u,-u+v); (-z,-y,x,-t+u,-t+v+1/2,-t)

SUPERCENTERED SETTING
Modulation vectors: Q1'=(B,0,0), Q2'=(0,B,0), Q3'=(0,0,B), where B=b
Centering: (0,0,0,0,0,0); (1/2,1/2,1/2,0,0,0); (0,0,0,1/2,1/2,1/2); (1/2,1/2,1/2,1/2,1/2,1/2)
Non-lattice generators: (X,Y,-Z,T,U+1/2,-V+1/2); (-Z,-X,-Y,-V,-T,-U); (Y,X,Z,U+1/4,T+1/4,V+1/4)
Non-lattice operators: (X,Y,Z,T,U,V); (X,-Y,-Z,T,-U+1/2,-V); (-X,Y,-Z,-T,U,-V+1/2); (-X,-Y,Z,-T+1/2,-U,V); (Y,Z,X,U,V,T); (Y,-Z,-X,U,-V+1/2,-T); (-Y,Z,-X,-U,V,-T+1/2); (-Y,-Z,X,-U+1/2,-V,T); (Z,X,Y,V,T,U); (Z,-X,-Y,V,-T+1/2,-U); (-Z,X,-Y,-V,T,-U+1/2); (-Z,-X,Y,-V+1/2,-T,U); (-Y,-X,-Z,-U+1/4,-T+1/4,-V+1/4); (-Y,X,Z,-U+1/4,T+1/4,V+3/4); (Y,-X,Z,U+1/4,-T+3/4,V+3/4); (Y,X,-Z,U+1/4,T+3/4,-V+1/4); (-X,-Z,-Y,-T+1/4,-V+1/4,-U+1/4); (-X,Z,Y,-T+1/4,V+1/4,U+3/4); (X,-Z,Y,T+1/4,-V+3/4,U+3/4); (X,Z,-Y,T+1/4,V+3/4,-U+1/4); (-Z,-Y,-X,-V+1/4,-U+1/4,-T+1/4); (-Z,Y,X,-V+1/4,U+1/4,T+3/4); (Z,-Y,X,V+1/4,-U+3/4,T+3/4); (Z,Y,-X,V+1/4,U+3/4,-T+1/4); (-X,-Y,-Z,-T,-U,-V); (-X,Y,Z,-T,U+1/2,V); (X,-Y,Z,T,-U,V+1/2); (X,Y,-Z,T+1/2,U,-V); (-Y,-Z,-X,-U,-V,-T); (-Y,Z,X,-U,V+1/2,T); (Y,-Z,X,U,-V,T+1/2); (Y,Z,-X,U+1/2,V,-T); (-Z,-X,-Y,-V,-T,-U); (-Z,X,Y,-V,T+1/2,U); (Z,-X,Y,V,-T,U+1/2); (Z,X,-Y,V+1/2,T,-U); (Y,X,Z,U+1/4,T+1/4,V+1/4); (Y,-X,-Z,U+1/4,-T+1/4,-V+3/4); (-Y,X,-Z,-U+1/4,T+3/4,-V+3/4); (-Y,-X,Z,-U+1/4,-T+3/4,V+1/4); (X,Z,Y,T+1/4,V+1/4,U+1/4); (X,-Z,-Y,T+1/4,-V+1/4,-U+3/4); (-X,Z,-Y,-T+1/4,V+3/4,-U+3/4); (-X,-Z,Y,-T+1/4,-V+3/4,U+1/4); (Z,Y,X,V+1/4,U+1/4,T+1/4); (Z,-Y,-X,V+1/4,-U+1/4,-T+3/4); (-Z,Y,-X,-V+1/4,U+3/4,-T+3/4); (-Z,-Y,X,-V+1/4,-U+3/4,T+1/4)
Reflection conditions: HKLMNP:H+K+L=2n; HKLMNP:M+N+P=2n; HHLMMP:2M+P=4n; H-HLM-MP:2M+P=4n; HKHMNM:2M+N=4n; HK-HMN-M:2M-N=4n; HKKMNN:M+2N=4n; HK-KMN-N:M+2N=4n; HK0MN0:M=2n; H0LM0P:P=2n; 0KL0NP:N=2n

Affine transformation to standard basic space group setting

$S * g(\text{input}) * S^{-1} = g(\text{standard})$,
where g is an augmented matrix for an operation in the superspace group.
Also, S * r(input) = r(standard),
where r is an augmented position vector, (x,y,z,t,u,v,1).

$$S = \begin{pmatrix} 1&0&0&0&0&0&0\\ 0&1&0&0&0&0&0\\ 0&0&1&0&0&0&0\\ 0&0&0&1&0&0&0\\ 0&0&0&0&1&0&0\\ 0&0&0&0&0&1&0\\ 0&0&0&0&0&0&1 \end{pmatrix} \quad S^{-1} = \begin{pmatrix} 1&0&0&0&0&0&0\\ 0&1&0&0&0&0&0\\ 0&0&1&0&0&0&0\\ 0&0&0&1&0&0&0\\ 0&0&0&0&1&0&0\\ 0&0&0&0&0&1&0\\ 0&0&0&0&0&0&1 \end{pmatrix}$$

a1' = a1	a1*' = a1*	q1' = q1 = (0,b,b)
a2' = a2	a2*' = a2*	q2' = q2 = (b,0,b)
a3' = a3	a3*' = a3*	q3' = q3 = (b,b,0)

a1 = a1'	a1* = a1*'	q1 = q1' = (0,b,b)
a2 = a2'	a2* = a2*'	q2 = q2' = (b,0,b)
a3 = a3'	a3* = a3*'	q3 = q3' = (b,b,0)

findssg Xm-3m(b,0,0)00q(0,b,0)s0q(0,0,b)00q

Generators of the standard supercentered setting have been entered into findssg.

Input setting

Centering
(0,0,0,0,0,0); (1/2,1/2,1/2,0,0,0); (0,0,0,1/2,1/2,1/2); (1/2,1/2,1/2,1/2,1/2,1/2)
Operators
(x,y,-z,t,u+1/2,-v+1/2); (-z,-x,-y,-v,-t,-u); (y,x,z,u+1/4,t+1/4,v+1/4); (x,y,z,t,u,v); (-z,-x,y,-v,-t+1/2,u+1/2); (y,x,-z,u+1/4,t+3/4,-v+1/4); (z,-x,-y,v+1/2,-t,-u+1/2); (y,z,x,u,v,t); (-z,-y,-x,-v+3/4,-u+3/4,-t+3/4); (-y,z,x,-u+1/2,v,t+1/2); (z,-y,-x,v+3/4,-u+3/4,-t+1/4); (-x,-z,-y,-t+1/4,-v+1/4,-u+1/4); (-x,-z,y,-t+3/4,-v+1/4,u+3/4); (-x,z,-y,-t+1/4,v+3/4,-u+3/4); (z,y,x,v+1/4,u+1/4,t+1/4); (-y,-z,-x,-u,-v,-t); (z,-y,x,v+1/4,-u+3/4,t+3/4); (-y,z,-x,-u,v,-t+1/2); (z,-x,y,v+1/2,-t+1/2,u); (y,z,-x,u,v+1/2,-t+1/2); (-z,-y,x,-v+3/4,-u+1/4,t+3/4); (-x,z,y,-t+1/4,v+1/4,u+3/4); (z,y,-x,v+1/4,u+3/4,-t+1/4); (-y,-z,x,-u,-v+1/2,t+1/2); (y,-z,x,u+1/2,-v+1/2,t); (-x,-y,-z,-t,-u,-v); (x,z,y,t+1/4,v+1/4,u+1/4); (-x,y,-z,-t+1/2,u+1/2,-v); (x,-z,y,t+3/4,-v+1/4,u+1/4); (-y,x,z,-u+1/4,t+1/4,v+3/4); (x,-y,-z,t+1/2,-u,-v+1/2); (-y,x,-z,-u+1/4,t+3/4,-v+3/4); (x,-z,-y,t+3/4,-v+3/4,-u+1/4); (-x,y,z,-t+1/2,u,v+1/2); (-z,y,x,-v+3/4,u+3/4,t+1/4); (-y,-x,-z,-u+1/4,-t+1/4,-v+1/4); (z,x,y,v+1/2,t+1/2,u+1/2); (y,-x,-z,u+3/4,-t+3/4,-v+1/4); (-z,x,y,-v+1/2,t,u+1/2); (x,-y,z,t+1/2,-u+1/2,v); (-z,x,-y,-v,t,-u+1/2); (y,-x,z,u+1/4,-t+3/4,v+3/4); (y,-z,-x,u+1/2,-v,-t+1/2); (-x,-y,z,-t,-u+1/2,v+1/2); (x,z,-y,t+1/4,v+3/4,-u+1/4); (z,x,-y,v,t+1/2,-u+1/2); (-y,-x,z,-u+3/4,-t+1/4,v+3/4); (-z,y,-x,-v+3/4,u+1/4,-t+1/4)

Standard settings

Superspace group: 229.3.211.6 Im-3m(0,b,b)00q(b,0,b)s0q(b,b,0)00q [Y:none]
Bravais class: 3.211 Im-3m(0,b,b)(b,0,b)(b,b,0)   [JJdW:3.213]
Transformation to supercentered setting: A1=a1, A2=a2, A3=a3, A4=a5+a6, A5=a4+a6, A6=a4+a5

BASIC SPACE GROUP SETTING
Modulation vectors: q1'=(0,b,b), q2'=(b,0,b), q3'=(b,b,0)
Centering: (0,0,0,0,0,0); (1/2,1/2,1/2,0,0,0)
Non-lattice generators: (x,y,-z,-u+v+1,-t+v+1/2,v+1/2); (-z,-x,-y,-v,-t,-u); (y,x,z,u+1/2,t+1/2,v+1/2)
Non-lattice operators: (x,y,z,t,u,v); (x,-y,-z,-t+1/2,-t+v,-t+u+1/2); (-x,y,-z,-u+v+1/2,-u+1/2,t-u); (-x,-y,z,u-v,t-v+1/2,-v+1/2); (y,z,x,u,v,t); (y,-z,-x,-u+1/2,t-u,-u+v+1/2); (-y,z,-x,t-v+1/2,-v+1/2,u-v); (-y,-z,x,-t+v,-t+u+1/2,-t+1/2); (z,x,y,v,t,u); (z,-x,-y,-v+1/2,u-v,t-v+1/2); (-z,x,-y,-t+u+1/2,-t+1/2,-t+v); (-z,-x,y,t-u,-u+v+1/2,-u+1/2); (-y,-x,-z,-u+1/2,-t+1/2,-v+1/2); (-y,x,z,u,u-v,-t+u+1/2); (y,-x,z,t-v+1/2,t,t-u); (y,x,-z,-t+v,-u+v+1/2,v); (-x,-z,-y,-t+1/2,-v+1/2,-u+1/2); (-x,z,y,t,t-u,t-v+1/2); (x,-z,y,-u+v+1/2,v,-t+v); (x,z,-y,u-v,-t+u+1/2,u); (-z,-y,-x,-v+1/2,-u+1/2,-t+1/2); (-z,y,x,v,-t+v,-u+v+1/2); (z,-y,x,-t+u+1/2,u,u-v); (z,y,-x,t-u,t-v+1/2,t); (-x,-y,-z,-t,-u,-v); (-x,y,z,t+1/2,t-v,t-u+1/2); (x,-y,z,u-v+1/2,u+1/2,-t+u); (x,y,-z,-u+v,-t+v+1/2,v+1/2); (-y,-z,-x,-u,-v,-t); (-y,z,x,u+1/2,-t+u,u-v+1/2); (y,-z,x,-t+v+1/2,v+1/2,-u+v); (y,z,-x,t-v,t-u+1/2,t+1/2); (-z,-x,-y,-v,-t,-u); (-z,x,y,v+1/2,-u+v,-t+v+1/2); (z,-x,y,t-u+1/2,t+1/2,t-v); (z,x,-y,-t+u,u-v+1/2,u+1/2); (y,x,z,u+1/2,t+1/2,v+1/2); (y,-x,-z,-u,-u+v,t-u+1/2); (-y,x,-z,-t+v+1/2,-t,-t+u); (-y,-x,z,t-v,u-v+1/2,-v); (x,z,y,t+1/2,v+1/2,u+1/2); (x,-z,-y,-t,-t+u,-t+v+1/2); (-x,z,-y,u-v+1/2,-v,t-v); (-x,-z,y,-u+v,t-u+1/2,-u); (z,y,x,v+1/2,u+1/2,t+1/2); (z,-y,-x,-v,t-v,u-v+1/2); (-z,y,-x,t-u+1/2,-u,-u+v); (-z,-y,x,-t+u,-t+v+1/2,-t)

SUPERCENTERED SETTING
Modulation vectors: Q1'=(B,0,0), Q2'=(0,B,0), Q3'=(0,0,B), where B=b
Centering: (0,0,0,0,0,0); (1/2,1/2,1/2,0,0,0); (0,0,0,1/2,1/2,1/2); (1/2,1/2,1/2,1/2,1/2,1/2)
Non-lattice generators: (X,Y,-Z,T,U+1/2,-V+1/2); (-Z,-X,-Y,-V,-T,-U); (Y,X,Z,U+1/4,T+1/4,V+1/4)
Non-lattice operators: (X,Y,Z,T,U,V); (X,-Y,-Z,T,-U+1/2,-V); (-X,Y,-Z,-T,U,-V+1/2); (-X,-Y,Z,-T+1/2,-U,V); (Y,Z,X,U,V,T); (Y,-Z,-X,U,-V+1/2,-T); (-Y,Z,-X,-U,V,-T+1/2); (-Y,-Z,X,-U+1/2,-V,T); (Z,X,Y,V,T,U); (Z,-X,-Y,V,-T+1/2,-U); (-Z,X,-Y,-V,T,-U+1/2); (-Z,-X,Y,-V+1/2,-T,U); (-Y,-X,-Z,-U+1/4,-T+1/4,-V+1/4); (-Y,X,Z,-U+1/4,T+1/4,V+3/4); (Y,-X,Z,U+1/4,-T+3/4,V+3/4); (Y,X,-Z,U+1/4,T+3/4,-V+1/4); (-X,-Z,-Y,-T+1/4,-V+1/4,-U+1/4); (-X,Z,Y,-T+1/4,V+1/4,U+3/4); (X,-Z,Y,T+1/4,-V+3/4,U+3/4); (X,Z,-Y,T+1/4,V+3/4,-U+1/4); (-Z,-Y,-X,-V+1/4,-U+1/4,-T+1/4); (-Z,Y,X,-V+1/4,U+1/4,T+3/4); (Z,-Y,X,V+1/4,-U+3/4,T+3/4); (Z,Y,-X,V+1/4,U+3/4,-T+1/4); (-X,-Y,-Z,-T,-U,-V); (-X,Y,Z,-T,U+1/2,V); (X,-Y,Z,T,-U,V+1/2); (X,Y,-Z,T+1/2,U,-V); (-Y,-Z,-X,-U,-V,-T); (-Y,Z,X,-U,V+1/2,T); (Y,-Z,X,U,-V,T+1/2); (Y,Z,-X,U+1/2,V,-T); (-Z,-X,-Y,-V,-T,-U); (-Z,X,Y,-V,T+1/2,U); (Z,-X,Y,V,-T,U+1/2); (Z,X,-Y,V+1/2,T,-U); (Y,X,Z,U+1/4,T+1/4,V+1/4); (Y,-X,-Z,U+1/4,-T+1/4,-V+3/4); (-Y,X,-Z,-U+1/4,T+3/4,-V+3/4); (-Y,-X,Z,-U+1/4,-T+3/4,V+1/4); (X,Z,Y,T+1/4,V+1/4,U+1/4); (X,-Z,-Y,T+1/4,-V+1/4,-U+3/4); (-X,Z,-Y,-T+1/4,V+3/4,-U+3/4); (-X,-Z,Y,-T+1/4,-V+3/4,U+1/4); (Z,Y,X,V+1/4,U+1/4,T+1/4); (Z,-Y,-X,V+1/4,-U+1/4,-T+3/4); (-Z,Y,-X,-V+1/4,U+3/4,-T+3/4); (-Z,-Y,X,-V+1/4,-U+3/4,T+1/4)
Reflection conditions: HKLMNP:H+K+L=2n; HKLMNP:M+N+P=2n; HHLMMP:2M+P=4n; H-HLM-MP:2M+P=4n; HKHMNM:2M+N=4n; HK-HMN-M:2M-N=4n; HKKMNN:M+2N=4n; HK-KMN-N:M+2N=4n; HK0MN0:M=2n; H0LM0P:P=2n; 0KL0NP:N=2n

Affine transformation to standard basic space group setting

S * g(input) * S $^{-1}$ = g(standard),
where g is an augmented matrix for an operation in the superspace group.
Also, S * r(input) = r(standard),
where r is an augmented position vector, (x,y,z,t,u,v,1).

$$S = \begin{array}{ccccccc} 0 & 0 & 1 & 0 & 0 & 0 & 0 \\ 1 & 0 & 0 & 0 & 0 & 0 & 0 \\ 0 & 1 & 0 & 0 & 0 & 0 & 0 \\ 0 & 0 & 0 & 1 & 1 & 0 & 0 \\ 0 & 0 & 0 & 0 & 1 & 1 & 0 \\ 0 & 0 & 0 & 1 & 0 & 1 & 0 \\ 0 & 0 & 0 & 0 & 0 & 0 & 1 \end{array} \qquad S^{-1} = \begin{array}{ccccccc} 0 & 1 & 0 & 0 & 0 & 0 & 0 \\ 0 & 0 & 1 & 0 & 0 & 0 & 0 \\ 1 & 0 & 0 & 0 & 0 & 0 & 0 \\ 0 & 0 & 0 & 1/2 & -1/2 & 1/2 & 0 \\ 0 & 0 & 0 & 1/2 & 1/2 & -1/2 & 0 \\ 0 & 0 & 0 & -1/2 & 1/2 & 1/2 & 0 \\ 0 & 0 & 0 & 0 & 0 & 0 & 1 \end{array}$$

a1' = a3	a1*' = a3*	q1' = q1 + q2 = (0,b,b)
a2' = a1	a2*' = a1*	q2' = q2 + q3 = (b,0,b)
a3' = a2	a3*' = a2*	q3' = q1 + q3 = (b,b,0)

a1 = a2'	a1* = a2*'	q1 = 1/2 q1' - 1/2 q2' + 1/2 q3' = (b,0,0)
a2 = a3'	a2* = a3*'	q2 = 1/2 q1' + 1/2 q2' - 1/2 q3' = (0,b,0)
a3 = a1'	a3* = a1*'	q3 = -1/2 q1' + 1/2 q2' + 1/2 q3' = (0,0,b)
