# Supplementary material for: Equivalence of superspace groups
Source: Acta Crystallogr A. 2012 Nov 14;69(Pt 1):75–90. doi: 10.1107/S0108767312041657 (PMC3553647; doi:10.1107/S0108767312041657)
Supplement: Supplementary file 1 [file a-69-00075-sup1.zip › ssg3d_im3m_aaa_v6ni16si7.pdf]

229.3.214.8 Im-3m(a,a,a)q00(a,-a,-a)q00(-a,a,-a)000

Superspace group: 229.3.214.8 Im-3m(a,a,a)q00(a,-a,-a)q00(-a,a,-a)000 [Y:3.11733]
Bravais class: 3.214 Im-3m(a,a,a)(a,-a,-a)(-a,a,-a) [JJdW:3.216]
Transformation to supercentered setting: A1=a1, A2=a2, A3=a3, A4=a4+a5-a6, A5=a4-a5+a6, A6=a4-a5-a6

BASIC SPACE GROUP SETTING
Modulation vectors: q1=(a,a,a), q2=(a,-a,-a), q3=(-a,a,-a)
Centering: (0,0,0,0,0,0); (1/2,1/2,1/2,0,0,0)
Non-lattice generators: (x,y,-z,t+u+v+1,-v-1/2,-u-1/2); (-z,-x,-y,-t+1,t+u+v+1/2,-u-1/2); (y,x,z,t,v+1/2,u-1/2)
Non-lattice operators: (x,y,z,t,u,v); (x,-y,-z,u+1/2,t+1/2,-t-u-v); (-x,y,-z,v,-t-u-v+1/2,t); (-x,-y,z,-t-u-v,v+1/2,u+1/2); (y,z,x,t,v+1/2,-t-u-v); (y,-z,-x,v,t+1/2,u+1/2); (-y,z,-x,-t-u-v,u,t); (-y,-z,x,u+1/2,-t-u-v+1/2,v); (z,x,y,t,-t-u-v+1/2,u+1/2); (z,-x,-y,-t-u-v,t+1/2,v); (-z,x,-y,u+1/2,v+1/2,t); (-z,-x,y,v,u,-t-u-v); (-y,-x,-z,-t,-v+1/2,-u+1/2); (-y,x,z,-v,-t+1/2,t+u+v); (y,-x,z,-u+1/2,t+u+v+1/2,-t); (y,x,-z,t+u+v,-u,-v); (-x,-z,-y,-t,-u,t+u+v); (-x,z,y,-u+1/2,-t+1/2,-v); (x,-z,y,t+u+v,-v+1/2,-t); (x,z,-y,-v,t+u+v+1/2,-u+1/2); (-z,-y,-x,-t,t+u+v+1/2,-v); (-z,y,x,t+u+v,-t+1/2,-u+1/2); (z,-y,x,-v,-u,-t); (z,y,-x,-u+1/2,-v+1/2,t+u+v); (-x,-y,-z,-t,-u,-v); (-x,y,z,-u+1/2,-t+1/2,t+u+v); (x,-y,z,-v,t+u+v+1/2,-t); (x,y,-z,t+u+v,-v+1/2,-u+1/2); (-y,-z,-x,-t,-v+1/2,t+u+v); (-y,z,x,-v,-t+1/2,-u+1/2); (y,-z,x,t+u+v,-u,-t); (y,z,-x,-u+1/2,t+u+v+1/2,-v); (-z,-x,-y,-t,t+u+v+1/2,-u+1/2); (-z,x,y,t+u+v,-t+1/2,-v); (z,-x,y,-u+1/2,-v+1/2,-t); (z,x,-y,-v,-u,t+u+v); (y,x,z,t,v+1/2,u+1/2); (y,-x,-z,v,t+1/2,-t-u-v); (-y,x,-z,u+1/2,-t-u-v+1/2,t); (-y,-x,z,-t-u-v,u,v); (x,z,y,t,u,-t-u-v); (x,-z,-y,u+1/2,t+1/2,v); (-x,z,-y,-t-u-v,v+1/2,t); (-x,-z,y,v,-t-u-v+1/2,u+1/2); (z,y,x,t,-t-u-v+1/2,v); (z,-y,-x,-t-u-v,t+1/2,u+1/2); (-z,y,-x,v,u,t); (-z,-y,x,u+1/2,v+1/2,-t-u-v)

SUPERCENTERED SETTING
Modulation vectors: Q1=(A,0,0), Q2=(0,A,0), Q3=(0,0,A), where A=a
Centering: (0,0,0,0,0,0); (1/2,1/2,1/2,0,0,0); (0,0,0,1/2,1/2,0); (1/2,1/2,1/2,1/2,1/2,0); (0,0,0,1/2,0,1/2); (1/2,1/2,1/2,1/2,0,1/2); (0,0,0,0,1/2,1/2); (1/2,1/2,1/2,0,1/2,1/2)
Non-lattice generators: (X,Y,-Z,T+1/4,U+1/4,-V+1/2); (-Z,-X,-Y,-V+3/4,-T+1/4,-U); (Y,X,Z,U+1/4,T-1/4,V)
Non-lattice operators: (X,Y,Z,T,U,V); (X,-Y,-Z,T,-U+1/4,-V+1/4); (-X,Y,-Z,-T+1/4,U,-V+3/4); (-X,-Y,Z,-T+1/4,-U+3/4,V); (Y,Z,X,U+1/4,V,T+3/4); (Y,-Z,-X,U+1/4,-V+3/4,-T); (-Y,Z,-X,-U,V,-T); (-Y,-Z,X,-U,-V+1/4,T+1/4); (Z,X,Y,V+1/4,T+3/4,U); (Z,-X,-Y,V+1/4,-T,-U+3/4); (-Z,X,-Y,-V,T+1/4,-U+1/4); (-Z,-X,Y,-V,-T,U); (-Y,-X,-Z,-U+1/4,-T+3/4,-V); (-Y,X,Z,-U+1/4,T,V+3/4); (Y,-X,Z,U,-T+1/4,V+1/4); (Y,X,-Z,U,T,-V); (-X,-Z,-Y,-T,-V,-U); (-X,Z,Y,-T,V+1/4,U+1/4); (X,-Z,Y,T+1/4,-V,U+3/4); (X,Z,-Y,T+1/4,V+3/4,-U); (-Z,-Y,-X,-V+1/4,-U,-T+3/4); (-Z,Y,X,-V+1/4,U+3/4,T); (Z,-Y,X,V,-U,T); (Z,Y,-X,V,U+1/4,-T+1/4); (-X,-Y,-Z,-T,-U,-V); (-X,Y,Z,-T,U+1/4,V+1/4); (X,-Y,Z,T+1/4,-U,V+3/4); (X,Y,-Z,T+1/4,U+3/4,-V); (-Y,-Z,-X,-U+1/4,-V,-T+3/4); (-Y,Z,X,-U+1/4,V+3/4,T); (Y,-Z,X,U,-V,T); (Y,Z,-X,U,V+1/4,-T+1/4); (-Z,-X,-Y,-V+1/4,-T+3/4,-U); (-Z,X,Y,-V+1/4,T,U+3/4); (Z,-X,Y,V,-T+1/4,U+1/4); (Z,X,-Y,V,T,-U); (Y,X,Z,U+1/4,T+3/4,V); (Y,-X,-Z,U+1/4,-T,-V+3/4); (-Y,X,-Z,-U,T+1/4,-V+1/4); (-Y,-X,Z,-U,-T,V); (X,Z,Y,T,V,U); (X,-Z,-Y,T,-V+1/4,-U+1/4); (-X,Z,-Y,-T+1/4,V,-U+3/4); (-X,-Z,Y,-T+1/4,-V+3/4,U); (Z,Y,X,V+1/4,U,T+3/4); (Z,-Y,-X,V+1/4,-U+3/4,-T); (-Z,Y,-X,-V,U,-T); (-Z,-Y,X,-V,-U+1/4,T+1/4)
Reflection conditions: HKLMNP:M+N=2n; HKLMNP:M+P=2n; HKLMNP:H+K+L=2n; HK0MN0:M-N=4n; H0LM0P:M-P=4n; 0KL0NP:N+P=4n

-----

Four SSG exists with the same BSG and Bravais class but with different intrinsic translational components:
229.3.214.7 Im-3m(a,a,a)000(a,-a,-a)000(-a,a,-a)000
229.3.214.8 Im-3m(a,a,a)q00(a,-a,-a)q00(-a,a,-a)000
229.3.214.9 Im-3m(a,a,a)000(a,-a,-a)000(-a,a,-a)00s
229.3.214.10 Im-3m(a,a,a)q00(a,-a,-a)q00(-a,a,-a)00s
Only No. 229.3.214.8 fulfills the reported reflection conditions for $V_6Ni_{16}Si_7$,
R.L. Withers, Y.C. Feng & G.H. Lu, JPCM 2, 3187-3200 (1990).

SSG from Yamamoto:11733 Im3m(p-p-p,-pp-p,-p-pp)dtd

11733 Im3m(p-p-p,-pp-p,-p-pp)dtd equivalent group: Im3m(p-p-p,-pp-p,-p-pp)dtd

(000000;0001/21/20;0001/201/2;00001/21/2;1/21/21/2000;1/21/21/21/21/20;
1/21/21/21/201/2;1/21/21/201/21/2)

x,y,z,t,u,v; -x,-y,z,-t,-u,v; -x,y,-z,-t,u,-v; x,-y,-z,t,-u,-v; z,x,y,v,t,u; z,-x,-y,v,-t,-u; -z,-x,y,-v,-t,u; -z,x,-y,-v,t,-u; y,z,x,u,v,t; -y,z,-x,-u,v,-t; y,-z,-x,u,-v,-t; -y,-z,x,-u,-v,t; y,x,-z,1/4+u,3/4+t,3/4-v; -y,-x,-z,1/4-u,3/4-t,3/4-v; y,-x,z,1/4+u,3/4-t,3/4+v; -y,x,z,1/4-u,3/4+t,3/4+v; x,z,-y,1/4+t,3/4+v,3/4-u; -x,z,y,1/4-t,3/4+v,3/4+u; -x,-z,-y,1/4-t,3/4-v,3/4-u; x,-z,y,1/4+t,3/4-v,3/4+u; z,y,-x,1/4+v,3/4+u,3/4-t; z,-y,x,1/4+v,3/4-u,3/4+t; -z,y,x,1/4-v,3/4+u,3/4+t; -z,-y,-x,1/4-v,3/4-u,3/4-t; -x,-y,-z,1/4-t,1/4-u,1/4-v; x,y,-z,1/4+t,1/4+u,1/4-v; x,-y,z,1/4+t,1/4-u,1/4+v; -x,y,z,1/4-t,1/4+u,1/4+v; -z,-x,-y,1/4-v,1/4-t,1/4-u; -z,x,y,1/4-v,1/4+t,1/4+u; z,x,-y,1/4+v,1/4+t,1/4-u; z,-x,y,1/4+v,1/4-t,1/4+u; -y,-z,-x,1/4-u,1/4-v,1/4-t; y,-z,x,1/4+u,1/4-v,1/4+t; -y,z,x,1/4-u,1/4+v,1/4+t; y,z,-x,1/4+u,1/4+v,1/4-t; -y,-x,z,-u,1/2-t,1/2+v; y,x,z,u,1/2+t,1/2+v; -y,x,-z,-u,1/2+t,1/2-v; y,-x,-z,u,1/2-t,1/2-v; -x,-z,y,-t,1/2-v,1/2+u; x,-z,-y,t,1/2-v,1/2-u; x,z,y,t,1/2+v,1/2+u; -x,z,-y,-t,1/2+v,1/2-u; -z,-y,x,-v,1/2-u,1/2+t; -z,y,-x,-v,1/2+u,1/2-t; z,-y,-x,v,1/2-u,1/2-t; z,y,x,v,1/2+u,1/2+t;

hklmni:m+n=2n hklmni:m+i=2n hklmni:n+i=2n hklmni:h+k+l=2n hklmni:h+k+l+m+n=2n hklmni:h+k+l+m+i=2n hklmni:h+k+l+n+i=2n hh0nn0:n+n=2n h-h0-nn0:3n+n=4n 00l00i:3i=4n 00l00i:3i=4n h00m00:m=4n 0ll0ii:3i+3i=4n h00m00:m=4n 0k00n0:3n=4n l0li0i:i+i=2n 0k00n0:3n=4n h0-hm0-m:3m-m=4n hk0mn0:m+n=4n h0lm0i:m+i=4n 0kl0ni:n+i=4n h-hl-nni:3n+n+2i=4n kklnni:n+n+2i=4n hllmii:i+i=2n -lklmn-m:3m+2n-m=4n lklini:i+2n+i=4n

-----

findssg Im-3m(a,a,a)q00(a,-a,-a)q00(-a,a,-a)000

Generators of the standard BSG setting have been entered into findssg.

Input setting

Centering
(0,0,0,0,0,0); (1/2,1/2,1/2,0,0,0)
Operators
(x,y,-z,t+u+v,-v+1/2,-u+1/2); (-z,-x,-y,-t,t+u+v+1/2,-u+1/2); (y,x,z,t,v+1/2,u+1/2); (x,y,z,t,u,v); (-z,-x,y,v,u,-t-u-v); (y,x,-z,t+u+v,-u,-v); (z,-x,-y,-t-u-v,t+1/2,v); (y,z,x,t,v+1/2,-t-u-v); (-z,-y,-x,-t,t+u+v+1/2,-v); (-y,z,x,-v,-t+1/2,-u+1/2); (z,-y,-x,-t-u-v,t+1/2,u+1/2); (-x,-z,-y,-t,-u,t+u+v); (-x,-z,y,v,-t-u-v+1/2,u+1/2); (-x,z,-y,-t-u-v,v+1/2,t); (z,y,x,t,-t-u-v+1/2,v); (-y,-z,-x,-t,-v+1/2,t+u+v); (z,-y,x,-v,-u,-t); (-y,z,-x,-t-u-v,u,t); (z,-x,y,-u+1/2,-v+1/2,-t); (y,z,-x,-u+1/2,t+u+v+1/2,-v); (-z,-y,x,u+1/2,v+1/2,-t-u-v); (-x,z,y,-u+1/2,-t+1/2,-v); (z,y,-x,-u+1/2,-v+1/2,t+u+v); (-y,-z,x,u+1/2,-t-u-v+1/2,v); (y,-z,x,t+u+v,-u,-t); (-x,-y,-z,-t,-u,-v); (x,z,y,t,u,-t-u-v); (-x,y,-z,v,-t-u-v+1/2,t); (x,-z,y,t+u+v,-v+1/2,-t); (-y,x,z,-v,-t+1/2,t+u+v); (x,-y,-z,u+1/2,t+1/2,-t-u-v); (-y,x,-z,u+1/2,-t-u-v+1/2,t); (x,-z,-y,u+1/2,t+1/2,v); (-x,y,z,-u+1/2,-t+1/2,t+u+v); (-z,y,x,t+u+v,-t+1/2,-u+1/2); (-y,-x,-z,-t,-v+1/2,-u+1/2); (z,x,y,t,-t-u-v+1/2,u+1/2); (y,-x,-z,v,t+1/2,-t-u-v); (-z,x,y,t+u+v,-t+1/2,-v); (x,-y,z,-v,t+u+v+1/2,-t); (-z,x,-y,u+1/2,v+1/2,t); (y,-x,z,-u+1/2,t+u+v+1/2,-t); (y,-z,-x,v,t+1/2,u+1/2); (-x,-y,z,-t-u-v,v+1/2,u+1/2); (x,z,-y,-v,t+u+v+1/2,-u+1/2); (z,x,-y,-v,-u,t+u+v); (-y,-x,z,-t-u-v,u,v); (-z,y,-x,v,u,t)

Standard settings

Superspace group: 229.3.214.8 Im-3m(a,a,a)q00(a,-a,-a)q00(-a,a,-a)000 [Y:3.11733]
Bravais class: 3.214 Im-3m(a,a,a)(a,-a,-a)(-a,a,-a) [JJdW:3.216]
Transformation to supercentered setting: A1=a1, A2=a2, A3=a3, A4=a4+a5-a6, A5=a4-a5+a6, A6=a4-a5-a6

BASIC SPACE GROUP SETTING
Modulation vectors: q1'=(a,a,a), q2'=(a,-a,-a), q3'=(-a,a,-a)
Centering: (0,0,0,0,0,0); (1/2,1/2,1/2,0,0,0)
Non-lattice generators: (x,y,-z,t+u+v+1,-v-1/2,-u-1/2); (-z,-x,-y,-t+1,t+u+v+1/2,-u-1/2); (y,x,z,t,v+1/2,u-1/2)
Non-lattice operators: (x,y,z,t,u,v); (x,-y,-z,u+1/2,t+1/2,-t-u-v); (-x,y,-z,v,-t-u-v+1/2,t); (-x,-y,z,-t-u-v,v+1/2,u+1/2); (y,z,x,t,v+1/2,-t-u-v); (y,-z,-x,v,t+1/2,u+1/2); (-y,z,-x,-t-u-v,u,t); (-y,-z,x,u+1/2,-t-u-v+1/2,v); (z,x,y,t,-t-u-v+1/2,u+1/2); (z,-x,-y,-t-u-v,t+1/2,v); (-z,x,-y,u+1/2,v+1/2,t); (-z,-x,y,v,u,-t-u-v); (-y,-x,-z,-t,-v+1/2,-u+1/2); (-y,x,z,-v,-t+1/2,t+u+v); (y,-x,z,-u+1/2,t+u+v+1/2,-t); (y,x,-z,t+u+v,-u,-v); (-x,-z,-y,-t,-u,t+u+v); (-x,z,y,-u+1/2,-t+1/2,-v); (x,-z,y,t+u+v,-v+1/2,-t); (x,z,-y,-v,t+u+v+1/2,-u+1/2); (-z,-y,-x,-t,t+u+v+1/2,-v); (-z,y,x,t+u+v,-t+1/2,-u+1/2); (z,-y,x,-v,-u,-t); (z,y,-x,-u+1/2,-v+1/2,t+u+v); (-x,-y,-z,-t,-u,-v); (-x,y,z,-u+1/2,-t+1/2,t+u+v); (x,-y,z,-v,t+u+v+1/2,-t); (x,y,-z,t+u+v,-v+1/2,-u+1/2); (-y,-z,-x,-t,-v+1/2,t+u+v); (-y,z,x,-v,-t+1/2,-u+1/2); (y,-z,x,t+u+v,-u,-t); (y,z,-x,-u+1/2,t+u+v+1/2,-v); (-z,-x,-y,-t,t+u+v+1/2,-u+1/2); (-z,x,y,t+u+v,-t+1/2,-v); (z,-x,y,-u+1/2,-v+1/2,-t); (z,x,-y,-v,-u,t+u+v); (y,x,z,t,v+1/2,u+1/2); (y,-x,-z,v,t+1/2,-t-u-v); (-y,x,-z,u+1/2,-t-u-v+1/2,t); (-y,-x,z,-t-u-v,u,v); (x,z,y,t,u,-t-u-v); (x,-z,-y,u+1/2,t+1/2,v); (-x,z,-y,-t-u-v,v+1/2,t); (-x,-z,y,v,-t-u-v+1/2,u+1/2); (z,y,x,t,-t-u-v+1/2,v); (z,-y,-x,-t-u-v,t+1/2,u+1/2); (-z,y,-x,v,u,t); (-z,-y,x,u+1/2,v+1/2,-t-u-v)

SUPERCENTERED SETTING
Modulation vectors: Q1'=(A,0,0), Q2'=(0,A,0), Q3'=(0,0,A), where A=a
Centering: (0,0,0,0,0,0); (1/2,1/2,1/2,0,0,0); (0,0,0,1/2,1/2,0); (1/2,1/2,1/2,1/2,1/2,0); (0,0,0,1/2,0,1/2); (1/2,1/2,1/2,1/2,0,1/2); (0,0,0,0,1/2,1/2); (1/2,1/2,1/2,0,1/2,1/2)
Non-lattice generators: (X,Y,-Z,T+1/4,U+1/4,-V+1/2); (-Z,-X,-Y,-V+3/4,-T+1/4,-U); (Y,X,Z,U+1/4,T-1/4,V)
Non-lattice operators: (X,Y,Z,T,U,V); (X,-Y,-Z,T,-U+1/4,-V+1/4); (-X,Y,-Z,-T+1/4,U,-V+3/4); (-X,-Y,Z,-T+1/4,-U+3/4,V); (Y,Z,X,U+1/4,V,T+3/4); (Y,-Z,-X,U+1/4,-V+3/4,-T); (-Y,Z,-X,-U,V,-T); (-Y,-Z,X,-U,-V+1/4,T+1/4); (Z,X,Y,V+1/4,T+3/4,U); (Z,-X,-Y,V+1/4,-T,-U+3/4); (-Z,X,-Y,-V,T+1/4,-U+1/4); (-Z,-X,Y,-V,-T,U); (-Y,-X,-Z,-U+1/4,-T+3/4,-V); (-Y,X,Z,-U+1/4,T,V+3/4); (Y,-X,Z,U,-T+1/4,V+1/4); (Y,X,-Z,U,T,-V); (-X,-Z,-Y,-T,-V,-U); (-X,Z,Y,-T,V+1/4,U+1/4); (X,-Z,Y,T+1/4,-V,U+3/4); (X,Z,-Y,T+1/4,V+3/4,-U); (-Z,-Y,-X,-V+1/4,-U,-T+3/4); (-Z,Y,X,-V+1/4,U+3/4,T); (Z,-Y,X,V,-U,T); (Z,Y,-X,V,U+1/4,-T+1/4); (-X,-Y,-Z,-T,-U,-V); (-X,Y,Z,-T,U+1/4,V+1/4); (X,-Y,Z,T+1/4,-U,V+3/4); (X,Y,-Z,T+1/4,U+3/4,-V); (-Y,-Z,-X,-U+1/4,-V,-T+3/4); (-Y,Z,X,-U+1/4,V+3/4,T); (Y,-Z,X,U,-V,T); (Y,Z,-X,U,V+1/4,-T+1/4); (-Z,-X,-Y,-V+1/4,-T+3/4,-U); (-Z,X,Y,-V+1/4,T,U+3/4); (Z,-X,Y,V,-T+1/4,U+1/4); (Z,X,-Y,V,T,-U); (Y,X,Z,U+1/4,T+3/4,V); (Y,-X,-Z,U+1/4,-T,-V+3/4); (-Y,X,-Z,-U,T+1/4,-V+1/4); (-Y,-X,Z,-U,-T,V); (X,Z,Y,T,V,U); (X,-Z,-Y,T,-V+1/4,-U+1/4); (-X,Z,-Y,-T+1/4,V,-U+3/4); (-X,-Z,Y,-T+1/4,-V+3/4,U); (Z,Y,X,V+1/4,U,T+3/4); (Z,-Y,-X,V+1/4,-U+3/4,-T); (-Z,Y,-X,-V,U,-T); (-Z,-Y,X,-V,-U+1/4,T+1/4)
Reflection conditions: HKLMNP:M+N=2n; HKLMNP:M+P=2n; HKLMNP:H+K+L=2n; HK0MN0:M-N=4n; H0LM0P:M-P=4n; 0KL0NP:N+P=4n

Affine transformation to standard basic space group setting

S * g(input) * S $^{-1}$ = g(standard),
where g is an augmented matrix for an operation in the superspace group.
Also, S * r(input) = r(standard),
where r is an augmented position vector, (x,y,z,t,u,v,1).

$$S = \begin{matrix} 1 & 0 & 0 & 0 & 0 & 0 & 0 \\ 0 & 1 & 0 & 0 & 0 & 0 & 0 \\ 0 & 0 & 1 & 0 & 0 & 0 & 0 \\ 0 & 0 & 0 & 1 & 0 & 0 & 0 \\ 0 & 0 & 0 & 0 & 1 & 0 & 0 \\ 0 & 0 & 0 & 0 & 0 & 1 & 0 \\ 0 & 0 & 0 & 0 & 0 & 0 & 1 \end{matrix} \qquad S^{-1} = \begin{matrix} 1 & 0 & 0 & 0 & 0 & 0 & 0 \\ 0 & 1 & 0 & 0 & 0 & 0 & 0 \\ 0 & 0 & 1 & 0 & 0 & 0 & 0 \\ 0 & 0 & 0 & 1 & 0 & 0 & 0 \\ 0 & 0 & 0 & 0 & 1 & 0 & 0 \\ 0 & 0 & 0 & 0 & 0 & 1 & 0 \\ 0 & 0 & 0 & 0 & 0 & 0 & 1 \end{matrix}$$

a1' = a1	a1*' = a1*	q1' = q1 = (a,a,a)
a2' = a2	a2*' = a2*	q2' = q2 = (a,-a,-a)
a3' = a3	a3*' = a3*	q3' = q3 = (-a,a,-a)

a1 = a1'	a1* = a1*'	q1 = q1' = (a,a,a)
a2 = a2'	a2* = a2*'	q2 = q2' = (a,-a,-a)
a3 = a3'	a3* = a3*'	q3 = q3' = (-a,a,-a)

findssg Xm-3m(a,0,0)q00(0,0,a)q00(0,0,a)000

Generators of the standard supercentered setting have been entered into findssg.

Input setting

Centering
(0,0,0,0,0,0); (1/2,1/2,1/2,0,0,0); (0,0,0,1/2,1/2,0); (0,0,0,1/2,0,1/2); (0,0,0,0,1/2,1/2); (1/2,1/2,1/2,1/2,1/2,0); (1/2,1/2,1/2,1/2,0,1/2); (1/2,1/2,1/2,0,1/2,1/2)
Operators
(x,y,-z,t+1/4,u+1/4,-v+1/2); (-z,-x,-y,-v+3/4,-t+1/4,-u); (y,x,z,u+1/4,t+3/4,v); (x,y,z,t+1/2,u+1/2,v); (-z,-x,y,-v,-t+1/2,u+1/2); (y,x,-z,u+1/2,t,-v+1/2); (z,-x,-y,v+1/4,-t,-u+3/4); (y,z,x,u+3/4,v+1/2,t+3/4); (-z,-y,-x,-v+3/4,-u,-t+1/4); (-y,z,x,-u+1/4,v+1/4,t+1/2); (z,-y,-x,v+1/4,-u+3/4,-t); (-x,-z,-y,-t+1/2,-v+1/2,-u); (-x,-z,y,-t+3/4,-v+3/4,u+1/2); (-x,z,-y,-t+1/4,v,-u+3/4); (z,y,x,v+3/4,u+1/2,t+3/4); (-y,-z,-x,-u+1/4,-v+1/2,-t+1/4); (z,-y,x,v+1/2,-u,t+1/2); (-y,z,-x,-u,v,-t); (z,-x,y,v+1/2,-t+1/4,u+3/4); (y,z,-x,u,v+3/4,-t+3/4); (-z,-y,x,-v,-u+1/4,t+1/4); (-x,z,y,-t+1/2,v+1/4,u+3/4); (z,y,-x,v,u+3/4,-t+3/4); (-y,-z,x,-u+1/2,-v+3/4,t+1/4); (y,-z,x,u,-v,t); (-x,-y,-z,-t,-u+1/2,-v+1/2); (x,z,y,t+1/2,v+1/2,u); (-x,y,-z,-t+1/4,u,-v+3/4); (x,-z,y,t+3/4,-v,u+1/4); (-y,x,z,-u+1/4,t+1/2,v+1/4); (x,-y,-z,t,-u+1/4,-v+1/4); (-y,x,-z,-u,t+3/4,-v+3/4); (x,-z,-y,t,-v+1/4,-u+1/4); (-x,y,z,-t+1/2,u+3/4,v+1/4); (-z,y,x,-v+1/4,u+3/4,t); (-y,-x,-z,-u+3/4,-t+3/4,-v+1/2); (z,x,y,v+3/4,t+1/4,u); (y,-x,-z,u+1/4,-t,-v+3/4); (-z,x,y,-v+1/4,t+1/2,u+1/4); (x,-y,z,t+3/4,-u,v+1/4); (-z,x,-y,-v+1/2,t+3/4,-u+1/4); (y,-x,z,u,-t+1/4,v+1/4); (y,-z,-x,u+1/4,-v+1/4,-t+1/2); (-x,-y,z,-t+1/4,-u+3/4,v); (x,z,-y,t+3/4,v+3/4,-u+1/2); (z,x,-y,v+1/2,t+1/2,-u); (-y,-x,z,-u,-t,v); (-z,y,-x,-v+1/2,u,-t+1/2)

Standard settings

Superspace group: 229.3.214.8 Im-3m(a,a,a)q00(a,-a,-a)q00(-a,a,-a)000 [Y:3.11733]
Bravais class: 3.214 Im-3m(a,a,a)(a,-a,-a)(-a,a,-a) [JJdW:3.216]
Transformation to supercentered setting: A1=a1, A2=a2, A3=a3, A4=a4+a5-a6, A5=a4-a5+a6, A6=a4-a5-a6

BASIC SPACE GROUP SETTING
Modulation vectors: q1'=(a,a,a), q2'=(a,-a,-a), q3'=(-a,a,-a)
Centering: (0,0,0,0,0,0); (1/2,1/2,1/2,0,0,0)
Non-lattice generators: (x,y,-z,t+u+v+1,-v-1/2,-u-1/2); (-z,-x,-y,-t+1,t+u+v+1/2,-u-1/2); (y,x,z,t,v+1/2,u-1/2)
Non-lattice operators: (x,y,z,t,u,v); (x,-y,-z,u+1/2,t+1/2,-t-u-v); (-x,y,-z,v,-t-u-v+1/2,t); (-x,-y,z,-t-u-v,v+1/2,u+1/2); (y,z,x,t,v+1/2,-t-u-v); (y,-z,-x,v,t+1/2,u+1/2); (-y,z,-x,-t-u-v,u,t); (-y,-z,x,u+1/2,-t-u-v+1/2,v); (z,x,y,t,-t-u-v+1/2,u+1/2); (z,-x,-y,-t-u-v,t+1/2,v); (-z,x,-y,u+1/2,v+1/2,t); (-z,-x,y,v,u,-t-u-v); (-y,-x,-z,-t,-v+1/2,-u+1/2); (-y,x,z,-v,-t+1/2,t+u+v); (y,-x,z,-u+1/2,t+u+v+1/2,-t); (y,x,-z,t+u+v,-u,-v); (-x,-z,-y,-t,-u,t+u+v); (-x,z,y,-u+1/2,-t+1/2,-v); (x,-z,y,t+u+v,-v+1/2,-t); (x,z,-y,-v,t+u+v+1/2,-u+1/2); (-z,-y,-x,-t,t+u+v+1/2,-v); (-z,y,x,t+u+v,-t+1/2,-u+1/2); (z,-y,x,-v,-u,-t); (z,y,-x,-u+1/2,-v+1/2,t+u+v); (-x,-y,-z,-t,-u,-v); (-x,y,z,-u+1/2,-t+1/2,t+u+v); (x,-y,z,-v,t+u+v+1/2,-t); (x,y,-z,t+u+v,-v+1/2,-u+1/2); (-y,-z,-x,-t,-v+1/2,t+u+v); (-y,z,x,-v,-t+1/2,-u+1/2); (y,-z,x,t+u+v,-u,-t); (y,z,-x,-u+1/2,t+u+v+1/2,-v); (-z,-x,-y,-t,t+u+v+1/2,-u+1/2); (-z,x,y,t+u+v,-t+1/2,-v); (z,-x,y,-u+1/2,-v+1/2,-t); (z,x,-y,-v,-u,t+u+v); (y,x,z,t,v+1/2,u+1/2); (y,-x,-z,v,t+1/2,-t-u-v); (-y,x,-z,u+1/2,-t-u-v+1/2,t); (-y,-x,z,-t-u-v,u,v); (x,z,y,t,u,-t-u-v); (x,-z,-y,u+1/2,t+1/2,v); (-x,z,-y,-t-u-v,v+1/2,t); (-x,-z,y,v,-t-u-v+1/2,u+1/2); (z,y,x,t,-t-u-v+1/2,v); (z,-y,-x,-t-u-v,t+1/2,u+1/2); (-z,y,-x,v,u,t); (-z,-y,x,u+1/2,v+1/2,-t-u-v)

SUPERCENTERED SETTING
Modulation vectors: Q1'=(A,0,0), Q2'=(0,A,0), Q3'=(0,0,A), where A=a
Centering: (0,0,0,0,0,0); (1/2,1/2,1/2,0,0,0); (0,0,0,1/2,1/2,0); (1/2,1/2,1/2,1/2,1/2,0); (0,0,0,1/2,0,1/2); (1/2,1/2,1/2,1/2,0,1/2); (0,0,0,0,1/2,1/2); (1/2,1/2,1/2,0,1/2,1/2)
Non-lattice generators: (X,Y,-Z,T+1/4,U+1/4,-V+1/2); (-Z,-X,-Y,-V+3/4,-T+1/4,-U); (Y,X,Z,U+1/4,T-1/4,V)
Non-lattice operators: (X,Y,Z,T,U,V); (X,-Y,-Z,T,-U+1/4,-V+1/4); (-X,Y,-Z,-T+1/4,U,-V+3/4); (-X,-Y,Z,-T+1/4,-U+3/4,V); (Y,Z,X,U+1/4,V,T+3/4); (Y,-Z,-X,U+1/4,-V+3/4,-T); (-Y,Z,-X,-U,V,-T); (-Y,-Z,X,-U,-V+1/4,T+1/4); (Z,X,Y,V+1/4,T+3/4,U); (Z,-X,-Y,V+1/4,-T,-U+3/4); (-Z,X,-Y,-V,T+1/4,-U+1/4); (-Z,-X,Y,-V,-T,U); (-Y,-X,-Z,-U+1/4,-T+3/4,-V); (-Y,X,Z,-U+1/4,T,V+3/4); (Y,-X,Z,U,-T+1/4,V+1/4); (Y,X,-Z,U,T,-V); (-X,-Z,-Y,-T,-V,-U); (-X,Z,Y,-T,V+1/4,U+1/4); (X,-Z,Y,T+1/4,-V,U+3/4); (X,Z,-Y,T+1/4,V+3/4,-U); (-Z,-Y,-X,-V+1/4,-U,-T+3/4); (-Z,Y,X,-V+1/4,U+3/4,T); (Z,-Y,X,V,-U,T); (Z,Y,-X,V,U+1/4,-T+1/4); (-X,-Y,-Z,-T,-U,-V); (-X,Y,Z,-T,U+1/4,V+1/4); (X,-Y,Z,T+1/4,-U,V+3/4); (X,Y,-Z,T+1/4,U+3/4,-V); (-Y,-Z,-X,-U+1/4,-V,-T+3/4); (-Y,Z,X,-U+1/4,V+3/4,T); (Y,-Z,X,U,-V,T); (Y,Z,-X,U,V+1/4,-T+1/4); (-Z,-X,-Y,-V+1/4,-T+3/4,-U); (-Z,X,Y,-V+1/4,T,U+3/4); (Z,-X,Y,V,-T+1/4,U+1/4); (Z,X,-Y,V,T,-U); (Y,X,Z,U+1/4,T+3/4,V); (Y,-X,-Z,U+1/4,-T,-V+3/4); (-Y,X,-Z,-U,T+1/4,-V+1/4); (-Y,-X,Z,-U,-T,V); (X,Z,Y,T,V,U); (X,-Z,-Y,T,-V+1/4,-U+1/4); (-X,Z,-Y,-T+1/4,V,-U+3/4); (-X,-Z,Y,-T+1/4,-V+3/4,U); (Z,Y,X,V+1/4,U,T+3/4); (Z,-Y,-X,V+1/4,-U+3/4,-T); (-Z,Y,-X,-V,U,-T); (-Z,-Y,X,-V,-U+1/4,T+1/4)
Reflection conditions: HKLMNP:M+N=2n; HKLMNP:M+P=2n; HKLMNP:H+K+L=2n; HK0MN0:M-N=4n; H0LM0P:M-P=4n; 0KL0NP:N+P=4n

Affine transformation to standard basic space group setting

$S * g(\text{input}) * S^{-1} = g(\text{standard})$,
where g is an augmented matrix for an operation in the superspace group.
Also, $S * r(\text{input}) = r(\text{standard})$,
where r is an augmented position vector, (x,y,z,t,u,v,1).

$$S = \begin{pmatrix} 1 & 0 & 0 & 0 & 0 & 0 & 0 \\ 0 & 1 & 0 & 0 & 0 & 0 & 0 \\ 0 & 0 & 1 & 0 & 0 & 0 & 0 \\ 0 & 0 & 0 & 1 & 1 & 1 & 0 \\ 0 & 0 & 0 & 1 & -1 & -1 & 0 \\ 0 & 0 & 0 & -1 & 1 & -1 & 0 \\ 0 & 0 & 0 & 0 & 0 & 0 & 1 \end{pmatrix} \quad S^{-1} = \begin{pmatrix} 1 & 0 & 0 & 0 & 0 & 0 & 0 \\ 0 & 1 & 0 & 0 & 0 & 0 & 0 \\ 0 & 0 & 1 & 0 & 0 & 0 & 0 \\ 0 & 0 & 0 & 1/2 & 1/2 & 0 & 0 \\ 0 & 0 & 0 & 1/2 & 0 & 1/2 & 0 \\ 0 & 0 & 0 & 0 & -1/2 & -1/2 & 0 \\ 0 & 0 & 0 & 0 & 0 & 0 & 1 \end{pmatrix}$$

a1' = a1	a1*' = a1*	q1' = q1 + q2 + q3 = (a,a,a)
a2' = a2	a2*' = a2*	q2' = q1 - q2 - q3 = (a,-a,-a)
a3' = a3	a3*' = a3*	q3' = -q1 + q2 - q3 = (-a,a,-a)

a1 = a1'	a1* = a1*'	q1 = 1/2 q1' + 1/2 q2' = (a,0,0)
a2 = a2'	a2* = a2*'	q2 = 1/2 q1' + 1/2 q3' = (0,a,0)
a3 = a3'	a3* = a3*'	q3 = -1/2 q2' - 1/2 q3' = (0,0,a)

findssg   Im3m(p-p-p,-pp-p,-p-pp)dtd

Operators of Yamamoto: 11733 have been entered into findssg.

Input setting

Centering
(0,0,0,0,0,0); (1/2,1/2,1/2,0,0,0); (0,0,0,1/2,1/2,0); (0,0,0,1/2,0,1/2); (0,0,0,0,1/2,1/2); (1/2,1/2,1/2,1/2,1/2,0); (1/2,1/2,1/2,1/2,0,1/2); (1/2,1/2,1/2,0,1/2,1/2)
Operators
(-x,-y,z,-t,-u,v); (-x,y,-z,-t,u,-v); (x,-y,-z,t,-u,-v); (z,x,y,v,t,u); (z,-x,-y,v,-t,-u); (-z,-x,y,-v,-t,u); (-z,x,-y,-v,t,-u); (y,z,x,u,v,t); (-y,z,-x,-u,v,-t); (y,-z,-x,u,-v,-t); (-y,-z,x,-u,-v,t); (y,x,-z,u+1/4,t+3/4,-v+3/4); (-y,-x,-z,-u+1/4,-t+3/4,-v+3/4); (y,-x,z,u+1/4,-t+3/4,v+3/4); (-y,x,z,-u+1/4,t+3/4,v+3/4); (x,z,-y,t+1/4,v+3/4,-u+3/4); (-x,z,y,-t+1/4,v+3/4,u+3/4); (-x,-z,-y,-t+1/4,-v+3/4,-u+3/4); (x,-z,y,t+1/4,-v+3/4,u+3/4); (z,y,-x,v+1/4,u+3/4,-t+3/4); (z,-y,x,v+1/4,-u+3/4,t+3/4); (-z,y,x,-v+1/4,u+3/4,t+3/4); (-z,-y,-x,-v+1/4,-u+3/4,-t+3/4); (-x,-y,-z,-t+1/4,-u+1/4,-v+1/4); (x,y,-z,t+1/4,u+1/4,-v+1/4); (x,-y,z,t+1/4,-u+1/4,v+1/4); (-x,y,z,-t+1/4,u+1/4,v+1/4); (-z,-x,-y,-v+1/4,-t+1/4,-u+1/4); (-z,x,y,-v+1/4,t+1/4,u+1/4); (z,x,-y,v+1/4,t+1/4,-u+1/4); (z,-x,y,v+1/4,-t+1/4,u+1/4); (-y,-z,-x,-u+1/4,-v+1/4,-t+1/4); (y,-z,x,u+1/4,-v+1/4,t+1/4); (-y,z,x,-u+1/4,v+1/4,t+1/4); (y,z,-x,u+1/4,v+1/4,-t+1/4); (-y,-x,z,-u,-t+1/2,v+1/2); (y,x,z,u,t+1/2,v+1/2); (-y,x,-z,-u,t+1/2,-v+1/2); (x,y,z,t,u,v); (y,-x,-z,u,-t+1/2,-v+1/2); (z,-y,-x,v+1/2,-u,-t+1/2); (z,y,x,v+1/2,u,t+1/2); (-z,-y,x,-v+1/2,-u,t+1/2); (-z,y,-x,-v+1/2,u,-t+1/2); (-x,-z,y,-t+1/2,-v+1/2,u); (x,-z,-y,t+1/2,-v+1/2,-u); (x,z,y,t+1/2,v+1/2,u); (-x,z,-y,-t+1/2,v+1/2,-u)

Standard settings

Superspace group: 229.3.214.8 Im-3m(a,a,a)q00(a,-a,-a)q00(-a,a,-a)000 [Y:3.11733]
Bravais class: 3.214 Im-3m(a,a,a)(a,-a,-a)(-a,a,-a)   [JJdW:3.216]
Transformation to supercentered setting: A1=a1, A2=a2, A3=a3, A4=a4+a5-a6, A5=a4-a5+a6, A6=a4-a5-a6

BASIC SPACE GROUP SETTING
Modulation vectors: q1'=(a,a,a), q2'=(a,-a,-a), q3'=(-a,a,-a)
Centering: (0,0,0,0,0,0); (1/2,1/2,1/2,0,0,0)
Non-lattice generators: (x,y,-z,t+u+v+1,-v-1/2,-u-1/2); (-z,-x,-y,-t+1,t+u+v+1/2,-u-1/2); (y,x,z,t,v+1/2,u-1/2)
Non-lattice operators: (x,y,z,t,u,v); (x,-y,-z,u+1/2,t+1/2,-t-u-v); (-x,y,-z,v,-t-u-v+1/2,t); (-x,-y,z,-t-u-v,v+1/2,u+1/2); (y,z,x,t,v+1/2,-t-u-v); (y,-z,-x,v,t+1/2,u+1/2); (-y,z,-x,-t-u-v,u,t); (-y,-z,x,u+1/2,-t-u-v+1/2,v); (z,x,y,t,-t-u-v+1/2,u+1/2); (z,-x,-y,-t-u-v,t+1/2,v); (-z,x,-y,u+1/2,v+1/2,t); (-z,-x,y,v,u,-t-u-v); (-y,-x,-z,-t,-v+1/2,-u+1/2); (-y,x,z,-v,-t+1/2,t+u+v); (y,-x,z,-u+1/2,t+u+v+1/2,-t); (y,x,-z,t+u+v,-u,-v); (-x,-z,-y,-t,-u,t+u+v); (-x,z,y,-u+1/2,-t+1/2,-v); (x,-z,y,t+u+v,-v+1/2,-t); (x,z,-y,-v,t+u+v+1/2,-u+1/2); (-z,-y,-x,-t,t+u+v+1/2,-v); (-z,y,x,t+u+v,-t+1/2,-u+1/2); (z,-y,x,-v,-u,-t); (z,y,-x,-u+1/2,-v+1/2,t+u+v); (-x,-y,-z,-t,-u,-v); (-x,y,z,-u+1/2,-t+1/2,t+u+v); (x,-y,z,-v,t+u+v+1/2,-t); (x,y,-z,t+u+v,-v+1/2,-u+1/2); (-y,-z,-x,-t,-v+1/2,t+u+v); (-y,z,x,-v,-t+1/2,-u+1/2); (y,-z,x,t+u+v,-u,-t); (y,z,-x,-u+1/2,t+u+v+1/2,-v); (-z,-x,-y,-t,t+u+v+1/2,-u+1/2); (-z,x,y,t+u+v,-t+1/2,-v); (z,-x,y,-u+1/2,-v+1/2,-t); (z,x,-y,-v,-u,t+u+v); (y,x,z,t,v+1/2,u+1/2); (y,-x,-z,v,t+1/2,-t-u-v); (-y,x,-z,u+1/2,-t-u-v+1/2,t); (-y,-x,z,-t-u-v,u,v); (x,z,y,t,u,-t-u-v); (x,-z,-y,u+1/2,t+1/2,v); (-x,z,-y,-t-u-v,v+1/2,t); (-x,-z,y,v,-t-u-v+1/2,u+1/2); (z,y,x,t,-t-u-v+1/2,v); (z,-y,-x,-t-u-v,t+1/2,u+1/2); (-z,y,-x,v,u,t); (-z,-y,x,u+1/2,v+1/2,-t-u-v)

SUPERCENTERED SETTING
Modulation vectors: Q1'=(A,0,0), Q2'=(0,A,0), Q3'=(0,0,A), where A=a
Centering: (0,0,0,0,0,0); (1/2,1/2,1/2,0,0,0); (0,0,0,1/2,1/2,0); (1/2,1/2,1/2,1/2,1/2,0); (0,0,0,1/2,0,1/2); (1/2,1/2,1/2,1/2,0,1/2); (0,0,0,0,1/2,1/2); (1/2,1/2,1/2,0,1/2,1/2)
Non-lattice generators: (X,Y,-Z,T+1/4,U+1/4,-V+1/2); (-Z,-X,-Y,-V+3/4,-T+1/4,-U); (Y,X,Z,U+1/4,T-1/4,V)
Non-lattice operators: (X,Y,Z,T,U,V); (X,-Y,-Z,T,-U+1/4,-V+1/4); (-X,Y,-Z,-T+1/4,U,-V+3/4); (-X,-Y,Z,-T+1/4,-U+3/4,V); (Y,Z,X,U+1/4,V,T+3/4); (Y,-Z,-X,U+1/4,-V+3/4,-T); (-Y,Z,-X,-U,V,-T); (-Y,-Z,X,-U,-V+1/4,T+1/4); (Z,X,Y,V+1/4,T+3/4,U); (Z,-X,-Y,V+1/4,-T,-U+3/4); (-Z,X,-Y,-V,T+1/4,-U+1/4); (-Z,-X,Y,-V,-T,U); (-Y,-X,-Z,-U+1/4,-T+3/4,-V); (-Y,X,Z,-U+1/4,T,V+3/4); (Y,-X,Z,U,-T+1/4,V+1/4); (Y,X,-Z,U,T,-V); (-X,-Z,-Y,-T,-V,-U); (-X,Z,Y,-T,V+1/4,U+1/4); (X,-Z,Y,T+1/4,-V,U+3/4); (X,Z,-Y,T+1/4,V+3/4,-U); (-Z,-Y,-X,-V+1/4,-U,-T+3/4); (-Z,Y,X,-V+1/4,U+3/4,T); (Z,-Y,X,V,-U,T); (Z,Y,-X,V,U+1/4,-T+1/4); (-X,-Y,-Z,-T,-U,-V); (-X,Y,Z,-T,U+1/4,V+1/4); (X,-Y,Z,T+1/4,-U,V+3/4); (X,Y,-Z,T+1/4,U+3/4,-V); (-Y,-Z,-X,-U+1/4,-V,-T+3/4); (-Y,Z,X,-U+1/4,V+3/4,T); (Y,-Z,X,U,-V,T); (Y,Z,-X,U,V+1/4,-T+1/4); (-Z,-X,-Y,-V+1/4,-T+3/4,-U); (-Z,X,Y,-V+1/4,T,U+3/4); (Z,-X,Y,V,-T+1/4,U+1/4); (Z,X,-Y,V,T,-U); (Y,X,Z,U+1/4,T+3/4,V); (Y,-X,-Z,U+1/4,-T,-V+3/4); (-Y,X,-Z,-U,T+1/4,-V+1/4); (-Y,-X,Z,-U,-T,V); (X,Z,Y,T,V,U); (X,-Z,-Y,T,-V+1/4,-U+1/4); (-X,Z,-Y,-T+1/4,V,-U+3/4); (-X,-Z,Y,-T+1/4,-V+3/4,U); (Z,Y,X,V+1/4,U,T+3/4); (Z,-Y,-X,V+1/4,-U+3/4,-T); (-Z,Y,-X,-V,U,-T); (-Z,-Y,X,-V,-U+1/4,T+1/4)
Reflection conditions: HKLMNP:M+N=2n; HKLMNP:M+P=2n; HKLMNP:H+K+L=2n; HK0MN0:M-N=4n; H0LM0P:M-P=4n; 0KL0NP:N+P=4n

Affine transformation to standard basic space group setting

$S * g(\text{input}) * S^{-1} = g(\text{standard})$,
where g is an augmented matrix for an operation in the superspace group.
Also, S * r(input) = r(standard),
where r is an augmented position vector, (x,y,z,t,u,v,1).

$$S = \begin{pmatrix} 1&0&0&0&0&0&0\\ 0&1&0&0&0&0&0\\ 0&0&1&0&0&0&0\\ 0&0&0&1&1&1&5/8\\ 0&0&0&1&-1&-1&1/8\\ 0&0&0&-1&1&-1&5/8\\ 0&0&0&0&0&0&1 \end{pmatrix} \quad S^{-1} = \begin{pmatrix} 1&0&0&0&0&0&0\\ 0&1&0&0&0&0&0\\ 0&0&1&0&0&0&0\\ 0&0&0&1/2&1/2&0&-3/8\\ 0&0&0&1/2&0&1/2&-5/8\\ 0&0&0&0&-1/2&-1/2&3/8\\ 0&0&0&0&0&0&1 \end{pmatrix}$$

a1' = a1	a1*' = a1*	q1' = q1 + q2 + q3 = (a,a,a)
a2' = a2	a2*' = a2*	q2' = q1 - q2 - q3 = (a,-a,-a)
a3' = a3	a3*' = a3*	q3' = -q1 + q2 - q3 = (-a,a,-a)

a1 = a1'	a1* = a1*'	q1 = 1/2 q1' + 1/2 q2' = (a,0,0)
a2 = a2'	a2* = a2*'	q2 = 1/2 q1' + 1/2 q3' = (0,a,0)
a3 = a3'	a3* = a3*'	q3 = -1/2 q2' - 1/2 q3' = (0,0,a)

229.3.214.7 Im-3m(a,a,a)000(a,-a,-a)000(-a,a,-a)000

Superspace group: 229.3.214.7 Im-3m(a,a,a)000(a,-a,-a)000(-a,a,-a)000 [Y:3.11732, 3.11734]
Bravais class: 3.214 Im-3m(a,a,a)(a,-a,-a)(-a,a,-a) [JJdW:3.216]
Transformation to supercentered setting: A1=a1, A2=a2, A3=a3, A4=a4+a5-a6, A5=a4-a5+a6, A6=a4-a5-a6

BASIC SPACE GROUP SETTING
Modulation vectors: q1=(a,a,a), q2=(a,-a,-a), q3=(-a,a,-a)
Centering: (0,0,0,0,0,0); (1/2,1/2,1/2,0,0,0)
Non-lattice generators: (x,y,-z,t+u+v,-v,-u); (-z,-x,-y,-t,t+u+v,-u); (y,x,z,t,v,u)
Non-lattice operators: (x,y,z,t,u,v); (x,-y,-z,u,t,-t-u-v); (-x,y,-z,v,-t-u-v,t); (-x,-y,z,-t-u-v,v,u); (y,z,x,t,v,-t-u-v); (y,-z,-x,v,t,u); (-y,z,-x,-t-u-v,u,t); (-y,-z,x,u,-t-u-v,v); (z,x,y,t,-t-u-v,u); (z,-x,-y,-t-u-v,t,v); (-z,x,-y,u,v,t); (-z,-x,y,v,u,-t-u-v); (-y,-x,-z,-t,-v,-u); (-y,x,z,-v,-t,t+u+v); (y,-x,z,-u,t+u+v,-t); (y,x,-z,t+u+v,-u,-v); (-x,-z,-y,-t,-u,t+u+v); (-x,z,y,-u,-t,-v); (x,-z,y,t+u+v,-v,-t); (x,z,-y,-v,t+u+v,-u); (-z,-y,-x,-t,t+u+v,-v); (-z,y,x,t+u+v,-t,-u); (z,-y,x,-v,-u,-t); (z,y,-x,-u,-v,t+u+v); (-x,-y,-z,-t,-u,-v); (-x,y,z,-u,-t,t+u+v); (x,-y,z,-v,t+u+v,-t); (x,y,-z,t+u+v,-v,-u); (-y,-z,-x,-t,-v,t+u+v); (-y,z,x,-v,-t,-u); (y,-z,x,t+u+v,-u,-t); (y,z,-x,-u,t+u+v,-v); (-z,-x,-y,-t,t+u+v,-u); (-z,x,y,t+u+v,-t,-v); (z,-x,y,-u,-v,-t); (z,x,-y,-v,-u,t+u+v); (y,x,z,t,v,u); (y,-x,-z,v,t,-t-u-v); (-y,x,-z,u,-t-u-v,t); (-y,-x,z,-t-u-v,u,v); (x,z,y,t,u,-t-u-v); (x,-z,-y,u,t,v); (-x,z,-y,-t-u-v,v,t); (-x,-z,y,v,-t-u-v,u); (z,y,x,t,-t-u-v,v); (z,-y,-x,-t-u-v,t,u); (-z,y,-x,v,u,t); (-z,-y,x,u,v,-t-u-v)

SUPERCENTERED SETTING
Modulation vectors: Q1=(A,0,0), Q2=(0,A,0), Q3=(0,0,A), where A=a
Centering: (0,0,0,0,0,0); (1/2,1/2,1/2,0,0,0); (0,0,0,1/2,1/2,0); (1/2,1/2,1/2,1/2,1/2,0); (0,0,0,1/2,0,1/2); (1/2,1/2,1/2,1/2,0,1/2); (0,0,0,0,1/2,1/2); (1/2,1/2,1/2,0,1/2,1/2)
Non-lattice generators: (X,Y,-Z,T,U,-V); (-Z,-X,-Y,-V,-T,-U); (Y,X,Z,U,T,V)
Non-lattice operators: (X,Y,Z,T,U,V); (X,-Y,-Z,T,-U,-V); (-X,Y,-Z,-T,U,-V); (-X,-Y,Z,-T,-U,V); (Y,Z,X,U,V,T); (Y,-Z,-X,U,-V,-T); (-Y,Z,-X,-U,V,-T); (-Y,-Z,X,-U,-V,T); (Z,X,Y,V,T,U); (Z,-X,-Y,V,-T,-U); (-Z,X,-Y,-V,T,-U); (-Z,-X,Y,-V,-T,U); (-Y,-X,-Z,-U,-T,-V); (-Y,X,Z,-U,T,V); (Y,-X,Z,U,-T,V); (Y,X,-Z,U,T,-V); (-X,-Z,-Y,-T,-V,-U); (-X,Z,Y,-T,V,U); (X,-Z,Y,T,-V,U); (X,Z,-Y,T,V,-U); (-Z,-Y,-X,-V,-U,-T); (-Z,Y,X,-V,U,T); (Z,-Y,X,V,-U,T); (Z,Y,-X,V,U,-T); (-X,-Y,-Z,-T,-U,-V); (-X,Y,Z,-T,U,V); (X,-Y,Z,T,-U,V); (X,Y,-Z,T,U,-V); (-Y,-Z,-X,-U,-V,-T); (-Y,Z,X,-U,V,T); (Y,-Z,X,U,-V,T); (Y,Z,-X,U,V,-T); (-Z,-X,-Y,-V,-T,-U); (-Z,X,Y,-V,T,U); (Z,-X,Y,V,-T,U); (Z,X,-Y,V,T,-U); (Y,X,Z,U,T,V); (Y,-X,-Z,U,-T,-V); (-Y,X,-Z,-U,T,-V); (-Y,-X,Z,-U,-T,V); (X,Z,Y,T,V,U); (X,-Z,-Y,T,-V,-U); (-X,Z,-Y,-T,V,-U); (-X,-Z,Y,-T,-V,U); (Z,Y,X,V,U,T); (Z,-Y,-X,V,-U,-T); (-Z,Y,-X,-V,U,-T); (-Z,-Y,X,-V,-U,T)
Reflection conditions: HKLMNP:M+N=2n; HKLMNP:M+P=2n; HKLMNP:H+K+L=2n

-----

229.3.214.9 Im-3m(a,a,a)000(a,-a,-a)000(-a,a,-a)00s

Superspace group: 229.3.214.9 Im-3m(a,a,a)000(a,-a,-a)000(-a,a,-a)00s [Y:3.11735, 3.11737]
Bravais class: 3.214 Im-3m(a,a,a)(a,-a,-a)(-a,a,-a) [JJdW:3.216]
Transformation to supercentered setting: A1=a1, A2=a2, A3=a3, A4=a4+a5-a6, A5=a4-a5+a6, A6=a4-a5-a6

BASIC SPACE GROUP SETTING
Modulation vectors: q1=(a,a,a), q2=(a,-a,-a), q3=(-a,a,-a)
Centering: (0,0,0,0,0,0); (1/2,1/2,1/2,0,0,0)
Non-lattice generators: (x,y,-z,t+u+v+1/2,-v-1/2,-u-1/2); (-z,-x,-y,-t+1,t+u+v-1,-u-1/2); (y,x,z,t+1/2,v,u-1)
Non-lattice operators: (x,y,z,t,u,v); (x,-y,-z,u+1/2,t+1/2,-t-u-v+1/2); (-x,y,-z,v,-t-u-v,t); (-x,-y,z,-t-u-v+1/2,v+1/2,u+1/2); (y,z,x,t,v+1/2,-t-u-v+1/2); (y,-z,-x,v,t+1/2,u+1/2); (-y,z,-x,-t-u-v+1/2,u,t); (-y,-z,x,u+1/2,-t-u-v,v); (z,x,y,t,-t-u-v,u+1/2); (z,-x,-y,-t-u-v+1/2,t+1/2,v); (-z,x,-y,u+1/2,v+1/2,t); (-z,-x,y,v,u,-t-u-v+1/2); (-y,-x,-z,-t+1/2,-v,-u); (-y,x,z,-v+1/2,-t,t+u+v); (y,-x,z,-u,t+u+v+1/2,-t+1/2); (y,x,-z,t+u+v,-u+1/2,-v+1/2); (-x,-z,-y,-t+1/2,-u+1/2,t+u+v); (-x,z,y,-u,-t,-v+1/2); (x,-z,y,t+u+v,-v,-t+1/2); (x,z,-y,-v+1/2,t+u+v+1/2,-u); (-z,-y,-x,-t+1/2,t+u+v+1/2,-v+1/2); (-z,y,x,t+u+v,-t,-u); (z,-y,x,-v+1/2,-u+1/2,-t+1/2); (z,y,-x,-u,-v,t+u+v); (-x,-y,-z,-t,-u,-v); (-x,y,z,-u+1/2,-t+1/2,t+u+v+1/2); (x,-y,z,-v,t+u+v,-t); (x,y,-z,t+u+v+1/2,-v+1/2,-u+1/2); (-y,-z,-x,-t,-v+1/2,t+u+v+1/2); (-y,z,x,-v,-t+1/2,-u+1/2); (y,-z,x,t+u+v+1/2,-u,-t); (y,z,-x,-u+1/2,t+u+v,-v); (-z,-x,-y,-t,t+u+v,-u+1/2); (-z,x,y,t+u+v+1/2,-t+1/2,-v); (z,-x,y,-u+1/2,-v+1/2,-t); (z,x,-y,-v,-u,t+u+v+1/2); (y,x,z,t+1/2,v,u); (y,-x,-z,v+1/2,t,-t-u-v); (-y,x,-z,u,-t-u-v+1/2,t+1/2); (-y,-x,z,-t-u-v,u+1/2,v+1/2); (x,z,y,t+1/2,u+1/2,-t-u-v); (x,-z,-y,u,t,v+1/2); (-x,z,-y,-t-u-v,v,t+1/2); (-x,-z,y,v+1/2,-t-u-v+1/2,u); (z,y,x,t+1/2,-t-u-v+1/2,v+1/2); (z,-y,-x,-t-u-v,t,u); (-z,y,-x,v+1/2,u+1/2,t+1/2); (-z,-y,x,u,v,-t-u-v)

SUPERCENTERED SETTING
Modulation vectors: Q1=(A,0,0), Q2=(0,A,0), Q3=(0,0,A), where A=a
Centering: (0,0,0,0,0,0); (1/2,1/2,1/2,0,0,0); (0,0,0,1/2,1/2,0); (1/2,1/2,1/2,1/2,1/2,0); (0,0,0,1/2,0,1/2); (1/2,1/2,1/2,1/2,0,1/2); (0,0,0,0,1/2,1/2); (1/2,1/2,1/2,0,1/2,1/2)
Non-lattice generators: (X,Y,-Z,T,U,-V+1/2); (-Z,-X,-Y,-V,-T+1/4,-U+3/4); (Y,X,Z,U+1/4,T-1/4,V+1/2)
Non-lattice operators: (X,Y,Z,T,U,V); (X,-Y,-Z,T,-U,-V+1/2); (-X,Y,-Z,-T,U,-V); (-X,-Y,Z,-T,-U,V+1/2); (Y,Z,X,U+1/4,V+3/4,T); (Y,-Z,-X,U+1/4,-V+3/4,-T); (-Y,Z,-X,-U+1/4,V+1/4,-T); (-Y,-Z,X,-U+1/4,-V+1/4,T); (Z,X,Y,V,T+1/4,U+3/4); (Z,-X,-Y,V,-T+1/4,-U+1/4); (-Z,X,-Y,-V,T+1/4,-U+1/4); (-Z,-X,Y,-V,-T+1/4,U+3/4); (-Y,-X,-Z,-U+1/4,-T+1/4,-V); (-Y,X,Z,-U+1/4,T+1/4,V); (Y,-X,Z,U+1/4,-T+3/4,V); (Y,X,-Z,U+1/4,T+3/4,-V); (-X,-Z,-Y,-T,-V+1/4,-U+1/4); (-X,Z,Y,-T,V+1/4,U+3/4); (X,-Z,Y,T,-V+1/4,U+3/4); (X,Z,-Y,T,V+1/4,-U+1/4); (-Z,-Y,-X,-V,-U,-T+1/2); (-Z,Y,X,-V,U,T); (Z,-Y,X,V,-U,T+1/2); (Z,Y,-X,V,U,-T); (-X,-Y,-Z,-T,-U,-V); (-X,Y,Z,-T,U,V+1/2); (X,-Y,Z,T,-U,V); (X,Y,-Z,T,U,-V+1/2); (-Y,-Z,-X,-U+1/4,-V+3/4,-T); (-Y,Z,X,-U+1/4,V+3/4,T); (Y,-Z,X,U+1/4,-V+1/4,T); (Y,Z,-X,U+1/4,V+1/4,-T); (-Z,-X,-Y,-V,-T+1/4,-U+3/4); (-Z,X,Y,-V,T+1/4,U+1/4); (Z,-X,Y,V,-T+1/4,U+1/4); (Z,X,-Y,V,T+1/4,-U+3/4); (Y,X,Z,U+1/4,T+1/4,V); (Y,-X,-Z,U+1/4,-T+1/4,-V); (-Y,X,-Z,-U+1/4,T+3/4,-V); (-Y,-X,Z,-U+1/4,-T+3/4,V); (X,Z,Y,T,V+1/4,U+1/4); (X,-Z,-Y,T,-V+1/4,-U+3/4); (-X,Z,-Y,-T,V+1/4,-U+3/4); (-X,-Z,Y,-T,-V+1/4,U+1/4); (Z,Y,X,V,U,T+1/2); (Z,-Y,-X,V,-U,-T); (-Z,Y,-X,-V,U,-T+1/2); (-Z,-Y,X,-V,-U,T)
Reflection conditions: HKLMNP:M+N=2n; HKLMNP:M+P=2n; HKLMNP:H+K+L=2n;

HHLMMP:M=2n; H-HLM-MP:M=2n; HKHMNM:M=2n; HK-HMN-M:M=2n; HKKMNN:N=2n; HK-KMN-N:N=2n

-----

229.3.214.10 Im-3m(a,a,a)q00(a,-a,-a)q00(-a,a,-a)00s

Superspace group: 229.3.214.10 Im-3m(a,a,a)q00(a,-a,-a)q00(-a,a,-a)00s [Y:3.11736]
Bravais class: 3.214 Im-3m(a,a,a)(a,-a,-a)(-a,a,-a) [JJdW:3.216]
Transformation to supercentered setting: A1=a1, A2=a2, A3=a3, A4=a4+a5-a6, A5=a4-a5+a6, A6=a4-a5-a6

BASIC SPACE GROUP SETTING
Modulation vectors: q1=(a,a,a), q2=(a,-a,-a), q3=(-a,a,-a)
Centering: (0,0,0,0,0,0); (1/2,1/2,1/2,0,0,0)
Non-lattice generators: (x,y,-z,t+u+v+1/2,-v,-u); (-z,-x,-y,-t+1,t+u+v+1/2,-u-1); (y,x,z,t+1/2,v-1/2,u-1/2)
Non-lattice operators: (x,y,z,t,u,v); (x,-y,-z,u,t,-t-u-v+1/2); (-x,y,-z,v,-t-u-v+1/2,t); (-x,-y,z,-t-u-v+1/2,v,u); (y,z,x,t,v,-t-u-v+1/2); (y,-z,-x,v,t,u); (-y,z,-x,-t-u-v+1/2,u,t); (-y,-z,x,u,-t-u-v+1/2,v); (z,x,y,t,-t-u-v+1/2,u); (z,-x,-y,-t-u-v+1/2,t,v); (-z,x,-y,u,v,t); (-z,-x,y,v,u,-t-u-v+1/2); (-y,-x,-z,-t+1/2,-v+1/2,-u+1/2); (-y,x,z,-v+1/2,-t+1/2,t+u+v); (y,-x,z,-u+1/2,t+u+v,-t+1/2); (y,x,-z,t+u+v,-u+1/2,-v+1/2); (-x,-z,-y,-t+1/2,-u+1/2,t+u+v); (-x,z,y,-u+1/2,-t+1/2,-v+1/2); (x,-z,y,t+u+v,-v+1/2,-t+1/2); (x,z,-y,-v+1/2,t+u+v,-u+1/2); (-z,-y,-x,-t+1/2,t+u+v,-v+1/2); (-z,y,x,t+u+v,-t+1/2,-u+1/2); (z,-y,x,-v+1/2,-u+1/2,-t+1/2); (z,y,-x,-u+1/2,-v+1/2,t+u+v); (-x,-y,-z,-t,-u,-v); (-x,y,z,-u,-t,t+u+v+1/2); (x,-y,z,-v,t+u+v+1/2,-t); (x,y,-z,t+u+v+1/2,-v,-u); (-y,-z,-x,-t,-v,t+u+v+1/2); (-y,z,x,-v,-t,-u); (y,-z,x,t+u+v+1/2,-u,-t); (y,z,-x,-u,t+u+v+1/2,-v); (-z,-x,-y,-t,t+u+v+1/2,-u); (-z,x,y,t+u+v+1/2,-t,-v); (z,-x,y,-u,-v,-t); (z,x,-y,-v,-u,t+u+v+1/2); (y,x,z,t+1/2,v+1/2,u+1/2); (y,-x,-z,v+1/2,t+1/2,-t-u-v); (-y,x,-z,u+1/2,-t-u-v,t+1/2); (-y,-x,z,-t-u-v,u+1/2,v+1/2); (x,z,y,t+1/2,u+1/2,-t-u-v); (x,-z,-y,u+1/2,t+1/2,v+1/2); (-x,z,-y,-t-u-v,v+1/2,t+1/2); (-x,-z,y,v+1/2,-t-u-v,u+1/2); (z,y,x,t+1/2,-t-u-v,v+1/2); (z,-y,-x,-t-u-v,t+1/2,u+1/2); (-z,y,-x,v+1/2,u+1/2,t+1/2); (-z,-y,x,u+1/2,v+1/2,-t-u-v)

SUPERCENTERED SETTING
Modulation vectors: Q1=(A,0,0), Q2=(0,A,0), Q3=(0,0,A), where A=a
Centering: (0,0,0,0,0,0); (1/2,1/2,1/2,0,0,0); (0,0,0,1/2,1/2,0); (1/2,1/2,1/2,1/2,1/2,0); (0,0,0,1/2,0,1/2); (1/2,1/2,1/2,1/2,0,1/2); (0,0,0,0,1/2,1/2); (1/2,1/2,1/2,0,1/2,1/2)
Non-lattice generators: (X,Y,-Z,T+1/4,U+1/4,-V); (-Z,-X,-Y,-V+3/4,-T,-U+1/4); (Y,X,Z,U,T,V+1/2)
Non-lattice operators: (X,Y,Z,T,U,V); (X,-Y,-Z,T,-U+1/4,-V+3/4); (-X,Y,-Z,-T+1/4,U,-V+3/4); (-X,-Y,Z,-T+1/4,-U+1/4,V); (Y,Z,X,U,V+1/4,T+3/4); (Y,-Z,-X,U,-V,-T); (-Y,Z,-X,-U+1/4,V+1/4,-T); (-Y,-Z,X,-U+1/4,-V,T+3/4); (Z,X,Y,V+1/4,T,U+3/4); (Z,-X,-Y,V+1/4,-T+1/4,-U); (-Z,X,-Y,-V,T,-U); (-Z,-X,Y,-V,-T+1/4,U+3/4); (-Y,-X,-Z,-U,-T,-V+1/2); (-Y,X,Z,-U,T+1/4,V+1/4); (Y,-X,Z,U+1/4,-T,V+1/4); (Y,X,-Z,U+1/4,T+3/4,-V); (-X,-Z,-Y,-T,-V+1/4,-U+1/4); (-X,Z,Y,-T,V,U+1/2); (X,-Z,Y,T+1/4,-V+3/4,U); (X,Z,-Y,T+1/4,V,-U+1/4); (-Z,-Y,-X,-V+1/4,-U,-T+1/4); (-Z,Y,X,-V+1/4,U+3/4,T); (Z,-Y,X,V,-U,T+1/2); (Z,Y,-X,V,U+1/4,-T+1/4); (-X,-Y,-Z,-T,-U,-V); (-X,Y,Z,-T,U+1/4,V+3/4); (X,-Y,Z,T+1/4,-U,V+3/4); (X,Y,-Z,T+1/4,U+1/4,-V); (-Y,-Z,-X,-U,-V+1/4,-T+3/4); (-Y,Z,X,-U,V,T); (Y,-Z,X,U+1/4,-V+1/4,T); (Y,Z,-X,U+1/4,V,-T+3/4); (-Z,-X,-Y,-V+1/4,-T,-U+3/4); (-Z,X,Y,-V+1/4,T+1/4,U); (Z,-X,Y,V,-T,U); (Z,X,-Y,V,T+1/4,-U+3/4); (Y,X,Z,U,T,V+1/2); (Y,-X,-Z,U,-T+1/4,-V+1/4); (-Y,X,-Z,-U+1/4,T,-V+1/4); (-Y,-X,Z,-U+1/4,-T+3/4,V); (X,Z,Y,T,V+1/4,U+1/4); (X,-Z,-Y,T,-V,-U+1/2); (-X,Z,-Y,-T+1/4,V+3/4,-U); (-X,-Z,Y,-T+1/4,-V,U+1/4); (Z,Y,X,V+1/4,U,T+1/4); (Z,-Y,-X,V+1/4,-U+3/4,-T); (-Z,Y,-X,-V,U,-T+1/2); (-Z,-Y,X,-V,-U+1/4,T+1/4)
Reflection conditions: HKLMNP:M+N=2n; HKLMNP:M+P=2n; HKLMNP:H+K+L=2n; HHLMMP:P=2n; H-HLM-MP:M=2n; HKHMNM:M=2n; HK-HMN-M:M=2n;

HKKMNN:N=2n; HK-KMN-N:N=2n; HK0MN0:M+N=4n; H0LM0P:M-P=4n; 0KL0NP:N-P=4n
-----
