# Supplementary material for: Equivalence of superspace groups
Source: Acta Crystallogr A. 2012 Nov 14;69(Pt 1):75–90. doi: 10.1107/S0108767312041657 (PMC3553647; doi:10.1107/S0108767312041657)
Supplement: Supplementary file 1 [file a-69-00075-sup1.zip › ssg3d_p422_aag_tase4i.pdf]

97.3.179.24 I422(a,a,g)000(-a,a,g)000(a,-a,g)000

Superspace group: 97.3.179.24 I422(a,a,g)000(-a,a,g)000(a,-a,g)000 [Y:3.7043]
Bravais class: 3.179 I4/mmm(a,a,g)(-a,a,g)(a,-a,g) [JJdW:3.181]
Transformation to supercentered setting: A1=a1, A2=a2, A3=a3, A4=a4, A5=a5-a6, A6=a4+a5+a6

BASIC SPACE GROUP SETTING
Modulation vectors: q1=(a,a,g), q2=(-a,a,g), q3=(a,-a,g)
Centering: (0,0,0,0,0,0); (1/2,1/2,1/2,0,0,0)
Non-lattice generators: (-y,x,z,v,t,-t+u+v); (x,-y,-z,-u,-t,t-u-v); (-y,-x,-z,-t,-v,-u)
Non-lattice operators: (x,y,z,t,u,v); (x,-y,-z,-u,-t,t-u-v); (-x,y,-z,-v,t-u-v,-t); (-x,-y,z,-t+u+v,v,u); (-y,-x,-z,-t,-v,-u); (-y,x,z,v,t,-t+u+v); (y,-x,z,u,-t+u+v,t); (y,x,-z,t-u-v,-u,-v)

SUPERCENTERED SETTING
Modulation vectors: Q1=(A,A,0), Q2=(-A,A,0), Q3=(0,0,G), where A=a, G=g
Centering: (0,0,0,0,0,0); (1/2,1/2,1/2,0,0,0); (0,0,0,1/2,1/2,1/2); (1/2,1/2,1/2,1/2,1/2,1/2)
Non-lattice generators: (-Y,X,Z,-U,T,V); (X,-Y,-Z,-U,-T,-V); (-Y,-X,-Z,-T,U,-V)
Non-lattice operators: (X,Y,Z,T,U,V); (X,-Y,-Z,-U,-T,-V); (-X,Y,-Z,U,T,-V); (-X,-Y,Z,-T,-U,V); (-Y,-X,-Z,-T,U,-V); (-Y,X,Z,-U,T,V); (Y,-X,Z,U,-T,V); (Y,X,-Z,T,-U,-V)
Reflection conditions: HKLMNP:H+K+L=2n; HKLMNP:M+N+P=2n

One other SSG exists, with different intrinsic translational components:

Superspace group: 97.3.179.25 I422(a,a,g)000(-a,a,g)000(a,-a,g)q00 [Y:3.7045]
Bravais class: 3.179 I4/mmm(a,a,g)(-a,a,g)(a,-a,g) [JJdW:3.181]
Transformation to supercentered setting: A1=a1, A2=a2, A3=a3, A4=a4, A5=a5-a6, A6=a4+a5+a6

BASIC SPACE GROUP SETTING
Modulation vectors: q1=(a,a,g), q2=(-a,a,g), q3=(a,-a,g)
Centering: (0,0,0,0,0,0); (1/2,1/2,1/2,0,0,0)
Non-lattice generators: (-y,x,z,v+3/4,t+1/4,-t+u+v+1/4); (x,-y,-z,-u,-t,t-u-v); (-y,-x,-z,-t+3/4,-v+1/4,-u+1/4)
Non-lattice operators: (x,y,z,t,u,v); (x,-y,-z,-u,-t,t-u-v); (-x,y,-z,-v,t-u-v,-t); (-x,-y,z,-t+u+v,v,u); (-y,-x,-z,-t+3/4,-v+1/4,-u+1/4); (-y,x,z,v+3/4,t+1/4,-t+u+v+1/4); (y,-x,z,u+3/4,-t+u+v+1/4,t+1/4); (y,x,-z,t-u-v+3/4,-u+1/4,-v+1/4)

SUPERCENTERED SETTING
Modulation vectors: Q1=(A,A,0), Q2=(-A,A,0), Q3=(0,0,G), where A=a, G=g
Centering: (0,0,0,0,0,0); (1/2,1/2,1/2,0,0,0); (0,0,0,1/2,1/2,1/2); (1/2,1/2,1/2,1/2,1/2,1/2)
Non-lattice generators: (-Y,X,Z,-U+1/2,T,V+1/4); (X,-Y,-Z,-U,-T,-V); (-Y,-X,-Z,-T+1/2,U,-V+1/4)
Non-lattice operators: (X,Y,Z,T,U,V); (X,-Y,-Z,-U,-T,-V); (-X,Y,-Z,U,T,-V); (-X,-Y,Z,-T,-U,V); (-Y,-X,-Z,-T+1/2,U,-V+1/4); (-Y,X,Z,-U+1/2,T,V+1/4); (Y,-X,Z,U+1/2,-T,V+1/4); (Y,X,-Z,T+1/2,-U,-V+1/4)
Reflection conditions: HKLMNP:H+K+L=2n; HKLMNP:M+N+P=2n; 00L00P:P=4n
-----

findssg I422(a,a,g)000(-a,a,g)000(a,-a,g)000

Operators of the standard BSG setting have been inserted into findssg.

Input setting

Centering
(0,0,0,0,0,0); (1/2,1/2,1/2,0,0,0)
Operators
(-y,x,z,v,t,-t+u+v); (x,-y,-z,-u,-t,t-u-v); (-y,-x,-z,-t,-v,-u); (-x,-y,z,-t+u+v,v,u); (y,x,-z,t-u-v,-u,-v); (x,y,z,t,u,v); (-x,y,-z,-v,t-u-v,-t); (y,-x,z,u,-t+u+v,t)

Standard settings

Superspace group: 97.3.179.24 I422(a,a,g)000(-a,a,g)000(a,-a,g)000 [Y:3.7043]
Bravais class: 3.179 I4/mmm(a,a,g)(-a,a,g)(a,-a,g) [JJdW:3.181]
Transformation to supercentered setting: A1=a1, A2=a2, A3=a3, A4=a4, A5=a5-a6, A6=a4+a5+a6

BASIC SPACE GROUP SETTING
Modulation vectors: q1'=(a,a,g), q2'=(-a,a,g), q3'=(a,-a,g)
Centering: (0,0,0,0,0,0); (1/2,1/2,1/2,0,0,0)
Non-lattice generators: (-y,x,z,v,t,-t+u+v); (x,-y,-z,-u,-t,t-u-v); (-y,-x,-z,-t,-v,-u)
Non-lattice operators: (x,y,z,t,u,v); (x,-y,-z,-u,-t,t-u-v); (-x,y,-z,-v,t-u-v,-t); (-x,-y,z,-t+u+v,v,u); (-y,-x,-z,-t,-v,-u); (-y,x,z,v,t,-t+u+v); (y,-x,z,u,-t+u+v,t); (y,x,-z,t-u-v,-u,-v)

SUPERCENTERED SETTING
Modulation vectors: Q1'=(A,A,0), Q2'=(-A,A,0), Q3'=(0,0,G), where A=a, G=g
Centering: (0,0,0,0,0,0); (1/2,1/2,1/2,0,0,0); (0,0,0,1/2,1/2,1/2); (1/2,1/2,1/2,1/2,1/2,1/2)
Non-lattice generators: (-Y,X,Z,-U,T,V); (X,-Y,-Z,-U,-T,-V); (-Y,-X,-Z,-T,U,-V)
Non-lattice operators: (X,Y,Z,T,U,V); (X,-Y,-Z,-U,-T,-V); (-X,Y,-Z,U,T,-V); (-X,-Y,Z,-T,-U,V); (-Y,-X,-Z,-T,U,-V); (-Y,X,Z,-U,T,V); (Y,-X,Z,U,-T,V); (Y,X,-Z,T,-U,-V)
Reflection conditions: HKLMNP:H+K+L=2n; HKLMNP:M+N+P=2n

Affine transformation to standard basic space group setting

$S * g(input) * S^{-1} = g(standard)$,
where g is an augmented matrix for an operation in the superspace group.
Also, S * r(input) = r(standard),
where r is an augmented position vector, (x,y,z,t,u,v,1).

$$S = \begin{pmatrix} 1&0&0&0&0&0&0\\ 0&1&0&0&0&0&0\\ 0&0&1&0&0&0&0\\ 0&0&0&1&0&0&0\\ 0&0&0&0&1&0&0\\ 0&0&0&0&0&1&0\\ 0&0&0&0&0&0&1 \end{pmatrix} \quad S^{-1} = \begin{pmatrix} 1&0&0&0&0&0&0\\ 0&1&0&0&0&0&0\\ 0&0&1&0&0&0&0\\ 0&0&0&1&0&0&0\\ 0&0&0&0&1&0&0\\ 0&0&0&0&0&1&0\\ 0&0&0&0&0&0&1 \end{pmatrix}$$

a1' = a1
a2' = a2
a3' = a3

a1 = a1'
a2 = a2'
a3 = a3'

a1*' = a1*
a2*' = a2*
a3*' = a3*

a1* = a1*'
a2* = a2*'
a3* = a3*'

q1' = q1 = (a,a,g)
q2' = q2 = (-a,a,g)
q3' = q3 = (a,-a,g)

q1 = q1' = (a,a,g)
q2 = q2' = (-a,a,g)
q3 = q3' = (a,-a,g)

findssg X422(a,a,g)000(-a,a,g)000(a,-a,g)000

Generators of standard supercentered setting given to findssg.

Input setting

Centering
(0,0,0,0,0,0); (1/2,1/2,1/2,0,0,0); (0,0,0,1/2,1/2,1/2); (1/2,1/2,1/2,1/2,1/2,1/2)
Operators
(-y,x,z,-u,t,v); (x,-y,-z,-u,-t,-v); (-y,-x,-z,-t,u,-v); (-x,-y,z,-t,-u,v); (y,x,-z,t,-u,-v); (x,y,z,t,u,v); (-x,y,-z,u,t,-v); (y,-x,z,u,-t,v)

Standard settings

Superspace group: 97.3.179.24 I422(a,a,g)000(-a,a,g)000(a,-a,g)000 [Y:3.7043]
Bravais class: 3.179 I4/mmm(a,a,g)(-a,a,g)(a,-a,g) [JJdW:3.181]
Transformation to supercentered setting: A1=a1, A2=a2, A3=a3, A4=a4, A5=a5-a6, A6=a4+a5+a6

BASIC SPACE GROUP SETTING
Modulation vectors: q1'=(a,a,g), q2'=(-a,a,g), q3'=(a,-a,g)
Centering: (0,0,0,0,0,0); (1/2,1/2,1/2,0,0,0)
Non-lattice generators: (-y,x,z,v,t,-t+u+v); (x,-y,-z,-u,-t,t-u-v); (-y,-x,-z,-t,-v,-u)
Non-lattice operators: (x,y,z,t,u,v); (x,-y,-z,-u,-t,t-u-v); (-x,y,-z,-v,t-u-v,-t); (-x,-y,z,-t+u+v,v,u); (-y,-x,-z,-t,-v,-u); (-y,x,z,v,t,-t+u+v); (y,-x,z,u,-t+u+v,t); (y,x,-z,t-u-v,-u,-v)

SUPERCENTERED SETTING
Modulation vectors: Q1'=(A,A,0), Q2'=(-A,A,0), Q3'=(0,0,G), where A=a, G=g
Centering: (0,0,0,0,0,0); (1/2,1/2,1/2,0,0,0); (0,0,0,1/2,1/2,1/2); (1/2,1/2,1/2,1/2,1/2,1/2)
Non-lattice generators: (-Y,X,Z,-U,T,V); (X,-Y,-Z,-U,-T,-V); (-Y,-X,-Z,-T,U,-V)
Non-lattice operators: (X,Y,Z,T,U,V); (X,-Y,-Z,-U,-T,-V); (-X,Y,-Z,U,T,-V); (-X,-Y,Z,-T,-U,V); (-Y,-X,-Z,-T,U,-V); (-Y,X,Z,-U,T,V); (Y,-X,Z,U,-T,V); (Y,X,-Z,T,-U,-V)
Reflection conditions: HKLMNP:H+K+L=2n; HKLMNP:M+N+P=2n

Affine transformation to standard basic space group setting

$S * g(input) * S^{-1} = g(standard)$,
where g is an augmented matrix for an operation in the superspace group.
Also, S * r(input) = r(standard),
where r is an augmented position vector, (x,y,z,t,u,v,1).

$$S = \begin{pmatrix} 1&0&0&0&0&0&0\\ 0&1&0&0&0&0&0\\ 0&0&1&0&0&0&0\\ 0&0&0&-1&0&1&0\\ 0&0&0&0&-1&1&0\\ 0&0&0&0&1&1&0\\ 0&0&0&0&0&0&1 \end{pmatrix} \quad S^{-1} = \begin{pmatrix} 1&0&0&0&0&0&0\\ 0&1&0&0&0&0&0\\ 0&0&1&0&0&0&0\\ 0&0&0&-1&1/2&1/2&0\\ 0&0&0&0&-1/2&1/2&0\\ 0&0&0&0&1/2&1/2&0\\ 0&0&0&0&0&0&1 \end{pmatrix}$$

a1' = a1
a2' = a2
a3' = a3

a1 = a1'
a2 = a2'
a3 = a3'

a1*' = a1*
a2*' = a2*
a3*' = a3*

a1* = a1*'
a2* = a2*'
a3* = a3*'

q1' = -q1 + q3 = (a,a,g)
q2' = -q2 + q3 = (-a,a,g)
q3' = q2 + q3 = (a,-a,g)

q1 = -q1' + 1/2 q2' + 1/2 q3' = (-a,-a,0)
q2 = -1/2 q2' + 1/2 q3' = (a,-a,0)
q3 = 1/2 q2' + 1/2 q3' = (0,0,g)
