# Supplementary material for: Equivalence of superspace groups
Source: Acta Crystallogr A. 2012 Nov 14;69(Pt 1):75–90. doi: 10.1107/S0108767312041657 (PMC3553647; doi:10.1107/S0108767312041657)
Supplement: Supplementary file 2 [file a-69-00075-sup2.pdf]

Supplementary Material
for the article
Equivalence of superspace groups

Sander van Smaalen,[a]* Branton J. Campbell[b] and Harold T. Stokes[b]

[a] *Laboratory of Crystallography, University of Bayreuth, Bayreuth, Germany, and*
[b] *Department of Physics and Astronomy, Brigham Young University, Provo, Utah 84602, USA. E-mail: smash@uni-bayreuth.de*

(Received 0 XXXXXXX 0000; accepted 0 XXXXXXX 0000)

Abstract

A detailed discussion is provided of the algorithm for determination of the transformation S between two settings of $(3+d)$-dimensional superspace groups ($d = 1, 2, 3$).

1. Algorithm for determination of the transformation S.

Equivalence of two superspace groups is established by finding the transformation S between them as defined in Eq. (9) of the manuscript:

$$A(g^1) = S\,A(g^2)\,S^{-1}\,. \tag{9}$$

Failure to find such a transformation is considered proof that two superspace groups are inequivalent.

As a general strategy, we will select an appropriate range limit N_k for each of the independent variables $\tilde{S}_{sk}$ in Eq. (15),

$$\tilde{S}_{sj} = -\sum_{k=j+1}^{(3+d)^2-3d} \frac{B_{ik}}{B_{ij}}\,\tilde{S}_{sk}\,, \tag{15}$$

and try all possible combinations of independent integers such that $-N_k \le \tilde{S}_{sk} \le N_k$. For each trial set, we then calculate each of the dependent variables $\tilde{S}_{sj}$, check they are all integers, check that $\det(S_R) = 1$ and $\det(S_\epsilon) = \pm 1$, and finally check if there is a solution to Eq. (12)

$$S_{vi} - \sum_{j=1}^{3+d} R_{sij}^{1l}\,S_{vj} = \mathbf{v}_{si}^{1l} - \sum_{j=1}^{3+d} S_{sij}\,\mathbf{v}_{sj}^{2l}\ (\mathrm{mod}\ 1)\,. \tag{12}$$

It is important that each of the N_k be sufficiently large that a solution to Eqs. (12) and (15) will be found whenever one exists. The following are our rules for determining the values of N_k.

Rule 1. The value of N_k must be greater than or equal to 1 for all independent variables capable of affecting the search for a solution (not all variables matter).

Rule 2. For a given j in Eq. (15), we can ensure that the dependent component $\tilde{S}_{sj}$ has the opportunity to take on non-zero integer values by requiring that each of the N_k be greater than or equal to the least common denominator of all of the coefficients B'_{ik}/B'_{ij}, some of which may be rational fractions.

Rule 3. The last term in Eq. (12) can be written as:

$$\sum_{\substack{k=1 \\ \text{independent}}}^{(3+d)^2-3d} C_{lik}\tilde{S}_{sk} \tag{S1}$$

where the sum is only over the independent components of $\tilde{S}_s$. For a given l and i, let m_{li} be the least common denominator of the nonzero coefficients $C_{li1}, C_{1i2}, \ldots$, and let m be the least common multiple of all of the m_{li}. Since Eq. (12) only needs to be satisfied to within an integer (mod 1), we can allow the full range of possible solutions by ensuring that its last term is able to take on nonzero integer values. This is accomplished by requiring that all of the N_k be greater than or equal to $\frac{1}{2}m$.

Rule 4. Before performing an equivalence test, we always begin by transforming the 3-dimensional (*i.e.* basic space group) parts of the two superspace groups into a common simple setting. This means that both groups have the same set of R matrices and $\mathbf{v}_3$ translations, so that the identity transformation ($S_R = 1$ and $S_v = 0$) relates their basic space groups. However, the two superspace groups may still differ in ϵ, M, and $\mathbf{v}_d$, such that there exists no overall solution for S with $S_R = 1$. In such a case, a solution with $S_R \neq 1$ may still exist, which involves a simple permutation or mixing of the external lattice vectors. Due to this preprocessing of the operators, we can use $N_k = 1$ for each independent variable in S_R, even if the previous rules say otherwise.

Rule 5. Let m_ϵ be the maximum value of N_k among the independent variables in S_ϵ. In order to allow solutions for which $\det(S_\epsilon) = \pm 1$, we require that all values of N_k for the independent variables in S_ϵ must greater than or equal to $m_\epsilon - 1$.

Based on the independent-variable range limits described above, the total number of independent-variable trials will be

$$\prod_{\substack{k=1 \\ \text{independent}}}^{(3+d)^2-3d} (2N_k + 1). \tag{S2}$$

For large numbers of independent variables and/or for large values of N_k, it is often impractical to conduct so many trials. We must therefore increase the efficiency of the search by recognizing situations in which the values of certain higher-priority variables fail a critical test, making it unnecessary to explore the values of any other variables until the higher-priority variables have been changed.

To facilitate this approach, we first classify the independent variables as follows:

X_R: The independent variables in $\tilde{S}_R$.

X_ϵ: The independent variables in $\tilde{S}_\epsilon$.

X_M: The independent variables in $\tilde{S}_M$.

X_I: The independent variables of $\tilde{S}_s$ that have non-integer rational fractions for coefficients in Eq. (15). The values of these independent variables can cause the dependent variables to have non-integer values.

X_T: The independent variables in $\tilde{S}_s$ that have nonzero coefficients in Eq. (S1). The values of these independent variables might make the last term in Eq. (12) too small to permit (mod 1) equivalence.

$\overline{X}_I$: The independent variables of $\tilde{S}_s$ that are not included in X_I.

$\overline{X}_T$: The independent variables of $\tilde{S}_s$ that are not included in X_T.

Consider that in Eq. (13),

$$\tilde{S}_s = \begin{pmatrix} \tilde{S}_M \\ \tilde{S}_R \\ \tilde{S}_\epsilon \end{pmatrix}, \tag{13}$$

we put $\tilde{S}_R$ and $\tilde{S}_\epsilon$ below $\tilde{S}_M$. When we bring B to row echelon form, this arrangement guarantees that dependent variables in S_ϵ will only depend on independent variables in X_ϵ. And because of the zeros that occur by definition in the upper right corner of

S_s, it also guarantees that dependent variables in S_R will only depend on independent variables in X_R.

Intersections with X_T and X_I split the variables of the X_R, X_ϵ and X_M classes into four subclasses each.

$$\begin{array}{lll} X_R \cap X_T \cap X_I & X_\epsilon \cap X_T \cap X_I & X_M \cap X_T \cap X_I \\ X_R \cap X_T \cap \overline{X}_I & X_\varepsilon \cap X_T \cap \overline{X}_I & X_M \cap X_T \cap \overline{X}_I \\ X_R \cap \overline{X}_T \cap X_I & X_\varepsilon \cap \overline{X}_T \cap X_I & X_M \cap \overline{X}_T \cap X_I \\ X_R \cap \overline{X}_T \cap \overline{X}_I & X_\varepsilon \cap \overline{X}_T \cap \overline{X}_I & X_M \cap \overline{X}_T \cap \overline{X}_I \end{array} \tag{S3}$$

The simplest way to construct an algorithm would be to have 12 successively nested loops to iterate the variables of each these subclasses (X_R subclasses first, X_ϵ subclasses next, and X_M subclasses last), such that we perform tests in each loop that have the potential to skip all inner loops as soon as a failure is detected. For example, a dependent-variable test (must be integers) can be performed on $\tilde{S}_R$ as soon as values have been assigned to all variables in $X_R \cap X_I$, and a determinant test can be performed as soon as values have been assigned to all variables in X_R.

In practice, we find it convenient to group some of the subclasses together, while keeping others separate. See the algorithm pseudo-code at the end of the appendix for details. In the outermost loop (#1), we systematically iterate over all variables in $X_R \cap X_T$. For each outer-loop trial, loops #2 and #3 iterate over the X_R variables that don't affect the translation test, and accumulate a list of candidates that pass the dependent-variable and determinant tests (called the $R\overline{T}$ list). Note that for each candidate in $X_R \cap \overline{X}_T \cap X_I$ that passes the independent variable test, we only need one candidate in $X_R \cap \overline{X}_T \cap \overline{X}_I$ that passes the determinant test—all candidates of this type are equivalent with respect to the tests that remain to be performed.

Provided that at least one successful candidate turns up in the $R\overline{T}$ list, we nest inward with a loop (#4) that iterates over $X_\epsilon \cap X_T$, which contains additional loops (#5 and #6) that accumulate a comparable list of successful $\epsilon\overline{T}$ candidates.

Provided that at least one successful candidate turns up in the $\epsilon\overline{T}$ list, we run

another loop (#7) that iterates over $X_M \cap X_T$. These trials each complete the assignment of variables in X_T, so that we are able to perform the translation test of Eq. (12). Each successful candidate is accumulated in the MT list. Note that the variables in X_M are not subject to a determinant test.

Nested loops #8, #9 and #10 now iterate over the accumulated lists of successful candidates from previous tests on specific variables. Taking $X_R \cap X_T$ and $X_\epsilon \cap X_T$ values from the loop #1 and loop #4 iterators, together with $X_R \cap \overline{X}_T$ values from the $R\overline{T}$ list, $X_\epsilon \cap \overline{X}_T$ values from the $\epsilon\overline{T}$ list, and $X_M \cap \overline{X}_T$ values from the $M\overline{T}$ list, we finally iterate over the $X_M \cap \overline{X}_T \cap X_I$ variables, and perform the last test: the dependent-variable on $\tilde{S}_M$. Only one success within this innermost loop proves the two groups to be equivalent. Observe that we never explore the variables in $X_M \cap \overline{X}_T \cap \overline{X}_I$ because they don't impact the outcomes of any of the tests performed—we simply set these variables to zero.

The accumulation of the $R\overline{T}$, $\epsilon\overline{T}$ and $M\overline{T}$ lists was convenient but not necessary. Their creation did not eliminate nested loops, but merely delayed the nesting until the last step of the algorithm (loops #8-#11). We accumulated these lists because the overhead associated with the tests relevant to each list were expensive. In the current algorithm, we get many valid candidates for a specific variable subclass after paying this expense only once.

Our algorithm is still a brute force exploratory approach to establishing superspace-group equivalence based on two sets of group operators. But it is also robust and highly efficient. Efficiency was enhanced in at least four ways: (1) separating dependent and independent variables, (2) keeping variable dependencies isolated within $\tilde{S}_R$ and $\tilde{S}_\epsilon$, (3) aggressively performing outer-loop tests that bypassed large numbers of inner-loop iterations, (4) and keeping the independent-variable ranges as small as possible without precluding viable solutions.

2. Algorithm pseudo-code

Set all values in the intersection $X_M \cap \overline{X}_T \cap \overline{X}_I$ equal to zero, since they have no influence on the solution.

Loop (1): Iterate over $X_R \cap X_T$.

  Clear the $R\overline{T}$ list.

  Loop (2): Iterate over $X_R \cap \overline{X}_T \cap X_I$.

    Calculate the dependent variables in S_R.

    If any of them are not integers, cycle Loop (2).

    Loop (3): Iterate over $X_R \cap \overline{X}_T \cap \overline{X}_I$.

      Calculate the dependent variables in S_R.

      If $\det(S_R) = 1$, store the values for $X_R \cap \overline{X}_T$ in

      the $R\overline{T}$ list and cycle Loop (2).

    End Loop (3)

  End Loop (2)

  If the $R\overline{T}$ list is empty, cycle Loop (1).

  Loop (4): Iterate over $X_\varepsilon \cap X_T$.

    Clear the $\varepsilon\overline{T}$ list.

    Loop (5): Iterate over $X_\epsilon \cap \overline{X}_T \cap X_I$.

      Calculate the dependent variables in S_ϵ.

      If any of them are not integers, cycle Loop (5).

      Loop (6): Iterate over $X_\epsilon \cap \overline{X}_T \cap \overline{X}_I$.

        Calculate the dependent variables in S_ε.

        If $\det(S_\epsilon) = \pm 1$, store the values for

        $X_\epsilon \cap \overline{X}_T$ in the $\epsilon\overline{T}$ list and cycle Loop (5).

      End Loop (6)

    End Loop (5)

If the $\epsilon\overline{T}$ list is empty, cycle Loop (4).

Clear the MT list.

Loop (7): Iterate over $X_M \cap X_T$.

If a solution for S_t exists, store the values for $X_M \cap X_T$ in the MT list.

End Loop (7)

If the MT list is empty, cycle Loop (4).

Loop (8): Iterate over entries in the $R\overline{T}$ list.

Loop (9): Iterate over entries in the $\epsilon\overline{T}$ list.

Loop (10): Iterate over entries in MT list.

Loop (11): Iterate over $X_M \cap \overline{X}_T \cap X_I$.

Calculate the dependent variables in S_M. If any of them are not integers, cycle Loop (11).

Else we have found a solution. The two groups are equivalent.

Exit algorithm.

End Loop (11).

End Loop (10).

End Loop (9).

End Loop (8).

End Loop (4).

End Loop (1).

There is no solution. The two groups are not equivalent.

Exit algorithm.
